# Reasoning Language Model as Rule Finder: A Case Study on C–H Bond Activation Using 2D Metal–Organic Frameworks

**DOI:** 10.1021/acscentsci.5c00561

**Published:** 2025-06-13

**Authors:** He Lin, Xiaoqi Cui, Binglin Dai, Jiawei Chen, Pengkun Su, Zhaomin Su, Huihui Hu, Yibin Jiang, Cheng Wang

**Affiliations:** † iChem, State Key Laboratory of Physical Chemistry of Solid Surfaces, College of Chemistry and Chemical Engineering, 12466 Xiamen University, Xiamen 361005, P. R. China; ‡ Innovation Laboratory for Sciences and Technologies of Energy Materials of Fujian Province (IKKEM), Xiamen 361005, P. R. China; § Jiangsu Key Laboratory for Science and Applications of Molecular Ferroelectrics, 12579 Southeast University, Nanjing 211189, P. R. China; ∥ Independent researcher, 15 Church Street, Glasgow G11 5JP, United Kingdom

## Abstract

Unraveling the structure–activity relationship in catalysis requires interpretable models that can extract governing principles from complex data sets. This study explores reasoning large language models (LLMs) as rule-finders for predicting C­(sp^3^)–H activation outcomes catalyzed by 2D Fe-terpyridine MOFs. Surface modifications with molecular modifiers systematically modulate the catalytic microenvironment, but linking modifier structure to activity remains challenging. While traditional descriptors offer high predictive accuracy, LLM-derived rules provide interpretable insights. Integrating LLM reasoning with experimental features (e.g., Fe-loading, modifier ratios) identified para-substituted benzoates with electron-withdrawing or coordinating groups as performance boosters. Validated by machine learning, this rule achieved 82.6% prediction accuracy. Notably, the coordinating group can become electron-withdrawing upon Fe^3+^ coordination or protonation. The LLM revealed that modifiers tune the catalyst’s electronic state rather than directly interacting with intermediates/transition states, bridging data-driven predictions with mechanistic understanding. This highlights LLM’s potential to derive chemically meaningful rules in catalysis.

## Graphical abstract

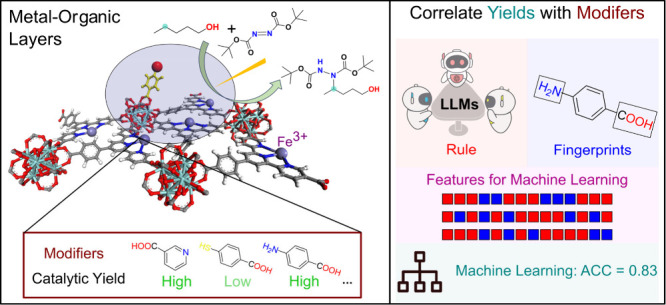

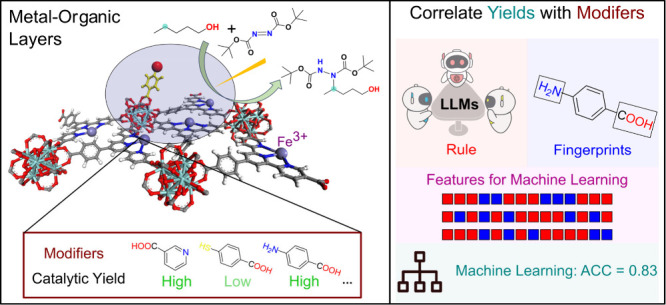

## Introduction

Metal–organic layers (MOLs),^1^ a subclass of ultrathin two-dimensional metal–organic frameworks (MOFs) with monolayer thickness, have attracted interest in catalysis studies, due to their tunable structures, ability to host catalytic centers, and readiness for surface modification that systematically tailors their catalytic microenvironments.^2^ However, understanding how surface modifications influence catalysis remains challenging. Molecular functionalization can simultaneously affect multiple aspects, including substrate transport, electronic environment, and transition-state stabilization, making it difficult to disentangle their individual contributions. Additionally, the high-dimensional design space and structural diversity of molecular modifiers require a robust analytical framework beyond manual inspection to extract meaningful insights.

Machine learning (ML) has emerged as a powerful tool for exploring complex chemical systems by uncovering structure–activity relationships.^3^ Many ML models rely on molecular descriptors^4^—quantitative representations of chemical structures and features—to link molecular properties with catalytic performance. Traditional descriptors, including string-based representation,^5^ molecular fingerprints,^6^ physicochemical properties,^7^ theoretical calculation-derived descriptors,^8^ graph-based representations,^9^ and pretrained embeddings,^10^ often achieve high predictive accuracy. However, their reliance on predefined features can limit interpretability, requiring extensive domain expertise and feature engineering.

Large language models (LLMs)^11,12^ offer an alternative approach by processing and interpreting complex chemical information using integrated prior chemical knowledge.^13,14^ LLMs have been applied in various chemical domains, including chemical information mining,^14^ robotic control,^15^ and the design^16^ and optimization^17^ of experimental workflows. Recent studies^18^ have demonstrated that GPT-3 ^12^ and GPT-4 ^19^ can correlate molecular SMILES with chemical properties through fine-tuning and in-context learning, making them promising tools for feature extraction and small-data prediction tasks. However, LLMs can generate erroneous outputs, known as hallucinations, necessitating iterative validation to ensure their reliability and relevance.

In this study, we explore the potential of reasoning language models as rule finders in catalyst design, forcing them to explicitly produce understandable and testable rules to address both the hallucination and the interpretability issue. The rules are then used as input features in a machine learning workflow to strictly test their reliability. These models are integrated into a multiagent collaboration framework to analyze MOL-catalyzed C–H bond activation in long-chain alcohols^20^ (Figure 1). The MOLs were constructed from Hf_6_(μ_3_-O)_4_(μ_3_-OH)_4_(HCO_2_)_6_ clusters as the secondary building units (SBUs) and terpyridine-tricarboxylate (TPY) as ligands, with Fe centers anchored to the TPY sites.^21^ These Fe centers catalyze sp^3^ C–H bond activation through photon-driven ligand-to-metal charge transfer (LMCT) process.^20^ By systematically modifying the MOL surfaces with a data set of carboxylic acid modifiers, we investigate how their structures influence catalytic performance (Figure 1) and found that the electronic effect of the modifiers, instead of their secondary interaction with the intermediate/transition state, tunes the activity of the MOL catalyst.

**Figure 1:**
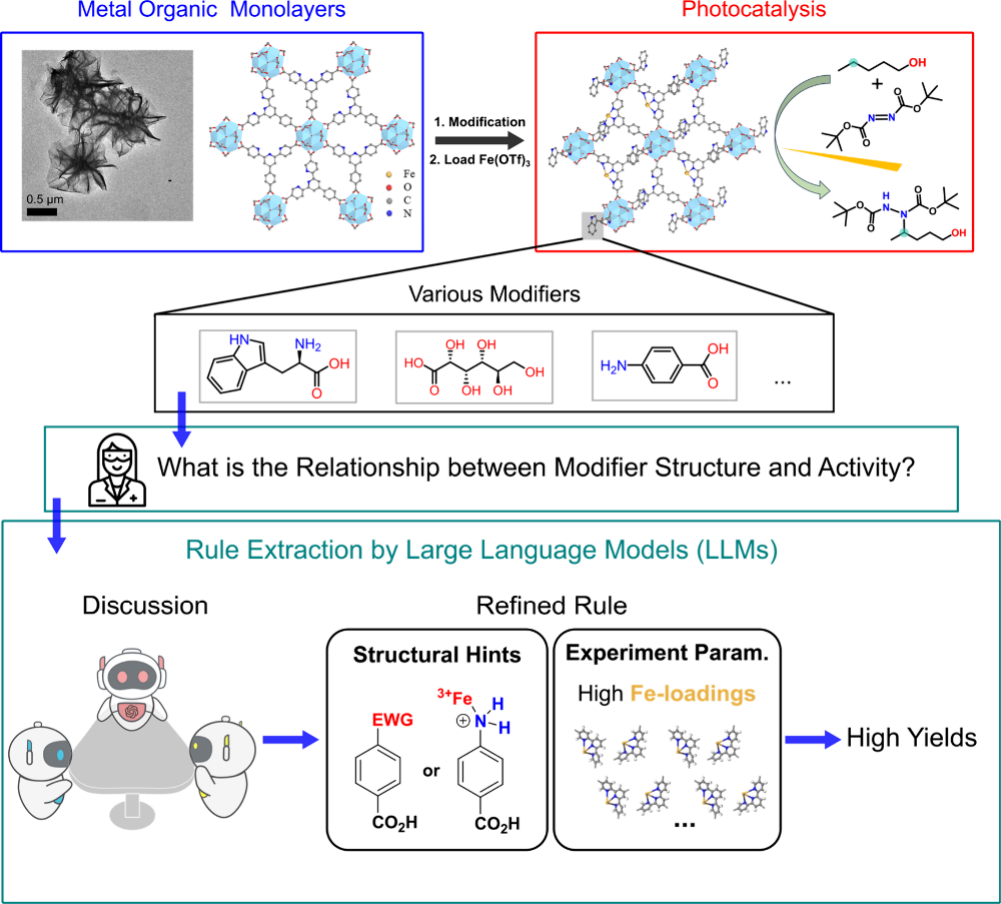
Overview of the workflow employed in this study. The study explores the δ-C­(sp^3^)–H bond functionalization of alcohols using Fe-loaded Hf-TPY-MOL modified with various molecular modifiers. The top panel illustrates the design and synthesis of the Hf-TPY-MOL framework (left) and its application in photocatalysis (right) to drive selective functionalization reactions. The iterative workflow employs a rule extraction pipeline, automated by large language models (LLMs), to uncover structure–activity relationships (bottom panel). By analyzing the interaction between structural features of modifiers and experimental outcomes, the approach identifies benzoic acid derivatives with para-substituted electron-withdrawing groups (EWGs) or coordinated amino groups as optimal modifiers, which, in combination with Fe-loadings, lead to a high yield. “Experiment Param.” stands for experimental parameters.

In the multiagent framework for the analysis, one agent generates hypotheses based on structure–property correlations, another critically evaluates these hypotheses through objective analysis, and a third one provides refinement suggestions to the first one in iterative cycles. The refined rules after several rounds of iterations are then passed to additional agents responsible for converting them into feature matrices and validating them against experimental data using traditional machine-learning models. The validation results are further analyzed by yet another agent, which provides structured feedback to the rule-generation group, enabling further iterative refinement. A finally refined rule was obtained, stating that para-substituting benzoate with an electron-withdrawing group or coordinating group can enhance activity. By integrating experimental data with reasoning-driven rule extraction, we establish a scalable framework for catalyst design, demonstrating the power of AI-driven reasoning in chemical research (Figure 1).

## Results and Discussion

### Photocatalytic C–H Amination of 1-Pentanol Using Hf-TPY-MOL

Hf-TPY-MOL, Hf_6_(μ_3_-O)_4_(μ_3_-OH)_4_(HCO_2_)_6_(TPY)_2_, was employed as a support for Fe^3+^ ions to study the heterogeneous photocatalytic δ-C­(sp^3^)–H amination of 1-pentanol.^20^ The Hf-TPY-MOL was synthesized following a previously published procedure,^21^ and its structure and morphology were confirmed using powder X-ray diffraction (PXRD) and transmission electron microscopy (TEM). To load Fe^3+^, Hf-TPY-MOL was dispersed in acetonitrile (CH_3_CN) containing 1.05 equivalents of Fe­(OTf)_3_ relative to TPY ligands and stirred at room temperature for 24 h. The resulting Fe^3+^-loaded Hf-TPY-MOL (Hf-TPY-MOL-Fe) exhibited a blue-violet color and was washed extensively with CH_3_CN to remove uncoordinated Fe^3+^, as confirmed by UV–vis spectroscopy. Inductively coupled plasma optical emission spectrometer (ICP-OES) indicated 102% metalation of Fe^3+^ onto the TPY ligands.

The selection of Fe­(OTf)_3_ as the Fe^3+^ source was based on the prior study,^20^ which compared various Fe^3+^ and Fe^2+^ salts. FeCl_3_ (99.99%) and Fe­(OTf)_3_ showed the highest catalytic activity with nearly equivalent performance. However, Fe­(OTf)_3_ was chosen to avoid potential interference from Cl^–^ in ligand-to-metal charge-transfer (LMCT) processes, allowing the separate study of Fe^3+^ and Cl^–^ roles (with Cl^–^ introduced via TBACl).

The catalytic activity of Hf-TPY-MOL-Fe was tested in the photocatalytic amination of 1-pentanol with di-tert-butyl azodicarboxylate (DBAD). A mixture of Hf-TPY-MOL-Fe (2 mg), DBAD (1 equivalent), 1-pentanol (3 equivalents), and tetrabutylammonium chloride (TBACl, 1.25 mol%) was irradiated with 393 nm LED light under a nitrogen atmosphere at room temperature for 12 h. Reaction yields were quantified using proton nuclear magnetic resonance (^1^H NMR) with CH_2_Br_2_ as an internal standard, yielding a moderate product conversion of 9% relative to DBAD, corresponding to a turnover number (TON) of 41 on Fe.

To confirm the heterogeneous nature of the catalysis and rule out the contribution of leached Fe^3+^ ions, we supplemented our analysis of heterogeneity through multiple substrates in the same process using the same catalyst. After 12 h of successful reaction on 1-pentanol, the supernatant was extracted and tested by UV–vis absorption spectroscopy. Then the activity of the supernatant was tested with the reaction to n-hexane. The UV–vis spectroscopy showed no detectable Fe^3+^ signals (Figure S1), and the result showed no conversion of n-hexane, confirming that the supernatant possessed no catalytic activity, supporting the conclusion that the catalysis is heterogeneous. In comparison, the heterogeneous catalyst Hf-TPY-MOL-Fe has high activity (TON of 233, yield of 35% after irradiation of 12 h) for converting *n*-hexane to a mixture of substitution at C-1 (di-tert-butyl 1-hexylhydrazine-1,2-dicarboxylate), substitution at C-2, and substitution at C-3 (1.0:2.8:3.0, normalized with respect to the number of Hs in C1, C2 and C3 ^22^). Furthermore, ICP-OES analysis verified that the Fe/Hf ratio remained unchanged before and after the reaction, confirming that Fe^3+^ ions remained bound to the MOL throughout the reaction.

To elucidate the reaction mechanism, free radical trapping agents were employed. The addition of radical quenchers, such as butylated hydroxytoluene (BHT) or 2,2,6,6-tetramethylpiperidine-1-oxyl (TEMPO), completely inhibited the reaction, supporting the proposed radical pathway. This finding aligns with prior research.^20^ Control experiments without Fe^3+^ showed no conversion, confirming that Fe^3+^ is essential for catalysis. Similarly, as shown in Table 1, reactions conducted in the dark yielded no conversion, demonstrating the photodriven nature of the reaction. Additionally, removing TBACl from the reaction system resulted in zero conversion, highlighting the critical of Cl^–^ in forming a Fe^3+^–Cl bond. This bond facilitates ligand-to-metal charge transfer (LMCT), generating Cl• radicals that initiate hydrogen atom transfer (HAT) with 1-pentanol. Substrate scope experiments (Figure S3) further validated the importance of the hydroxyl groups for δ-position selectivity via the [1,5] HAT pathway.

**Table 1:** Conditions for the Amination Reaction of n-Pentanol

Entry	Catalyst	Yield	TON
1	Hf-TPY-MOL (Fe)	9%	41
2	Hf-TPY-MOL (Fe)^a^	0	0
3	Hf-TPY-MOL (Fe)^b^	0	0
4	Fe(OTf)_3_	57%	229
5	Fe(OTf)_3_ ^c^	0	0
6	Hf-BTB-MOL (Fe)^a^	0	0
7	Hf-BTB-MOL (Fe)	0	0
8	Hf-BTB-MOL-l-aspartate (Fe)	80.4%	323
9	-	0	0
10	Hf-TPY-MOL-d-tryptophan (Fe)^d^	0	0
11	Hf-TPY-MOL-d-tryptophan (Fe)^e^	0	0

a Dark reaction.

b Dark reaction and 55 °C heated.

c No TBACl.

d 3 eq. BHT.

e 1.5 eq. TEMPO.

### Tuning Activity by Surface Modification of the MOLs

The Hf_6_(μ_3_-O)_4_(μ_3_-OH)_4_ SBU in the Hf-TPY-MOL contains six formate-capped sites that can be replaced by other carboxylates, enabling postsynthetic modification of the MOLs. This straightforward replacement chemistry allows systematic tuning of the surface properties (Figure 2a). Surface modification not only affects the interaction between the terpyridine-Fe^3+^-Cl active site and the substrate but also alters the surface solvent structure, thereby influencing catalytic activity. To investigate these effects, we systematically studied how different molecular modifiers impact the catalytic performance of Fe^3+^-loaded MOLs.

**Figure 2:**
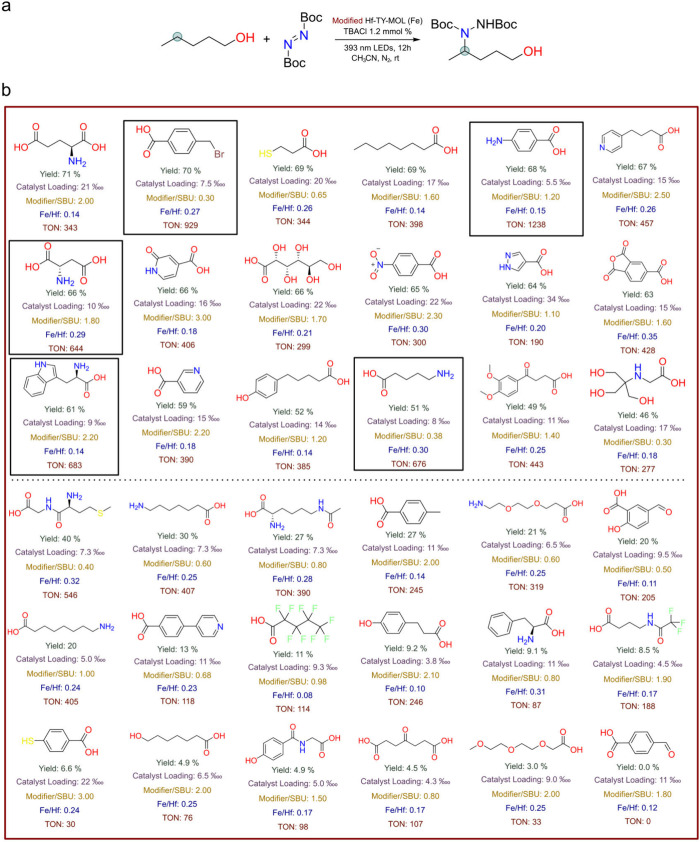
(a) The sp^3^ C–H activation reaction catalyzed by Hf-TPY-MOL-Fe-(modifier). (b) The reaction data corresponding to different modifiers on the Fe-loaded Hf-TPY-MOL. The yield of the catalysis reaction (%), catalyst (Fe) loading on the MOL 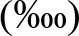, modifier/SBU ratio, and Fe/Hf ratio for each modified Hf-TPY-MOL are shown below each modifier. The modifiers are arranged in descending order of yield. Modifiers positioned above the dashed line are classified as high-yield, whereas those below are considered low-yield. The modifiers displayed in the black border are those with a TON larger than 600, indicating the necessity of an amine group for a high TON.

For each modifier, Hf-TPY-MOL was incubated with a 0.2 M carboxylic acid modifier solution in CH_3_CN, H_2_O, DMF, or THF, chosen based on solubility, at 55 °C for 24 h. Structural integrity after modification was confirmed by PXRD and TEM (Figure S2e,f). The amount of substituted modifier was quantified by proton nuclear magnetic resonance (^1^H NMR) after digesting the MOL with K_3_PO_4_/D_2_O, expressed as the Modifier/SBU ratio. Subsequently, Fe^3+^ was loaded onto the modified Hf-TPY-MOL by reacting with Fe­(OTf)_3_, and the resulting materials were tested as catalysts.

Significant variations in activity and yield were observed among different modifications (Figure 2b). Most carboxylic acid modifications enhanced activity, with several achieving a turnover number (TON) exceeding 600, indicating substantial improvement. However, certain modifiers, such as 2-(2-(2-methoxyethoxy)­ethoxy)­acetic acid, 4-mercaptobenzoic acid, and 4-formylbenzoic acid, inhibited the reaction, resulting in reduced catalytic activity.

Fe^3+^ ions can coordinate with either the TPY ligands or the molecular modifiers on the SBUs in Hf-TPY-MOL. To differentiate the roles of Fe^3+^ ions at these two sites, we designed a control catalyst, Hf-BTB-MOL, using 1,3,5-tri (4-carboxyphenyl) benzene (H_3_BTB) as the ligand, which lacks TPY sites for Fe coordination (Figure S2a). The synthesized Hf-BTB-MOL was characterized by PXRD and TEM (Figure S2b–d). Upon loading Fe^3+^ via reaction with Fe­(OTf)_3_ (1.05 equivalents) in CH_3_CN, yellow MOLs were obtained. Under standard catalytic conditions (Table 1), Hf-BTB-MOL (Fe) exhibited no activity (0% yield). However, when modified with l-aspartate, the catalyst achieved a significantly higher yield of 80.4%, indicating that both Fe^3+^ ions on TPY sites and Fe^3+^ ions interacting with modifiers contribute to the catalytic activity.

Additionally, a range of different alcohol substrates showed selective C–H conversion at the δ-position with respect to the hydroxyl group, while C–H conversion of alkanes without the hydroxyl group showed a distribution of site selectivity (Figure S3).

### Machine Learning Benchmark Using Traditional Descriptors

To study the system using conventional machine learning as a baseline (raw data shown in Table S7, Table S8, Figures S39–74), we constructed a traditional descriptor set by combining LoFFi^23^ molecular descriptors and additional descriptors. LoFFi incorporates RDKit descriptors,^24^ modified functional group fingerprints, and pooled properties at both atomic and molecular subsystem levels. Considering the carboxylate as the connecting point for the modifier, the modifier was fragmented starting from the carboxylic group at three levels (Figure 3a). For each level, the average values of the Marsilli–Gasteiger’s partial equalization of orbital electronegativities (PEOE) charge,^25^ logarithm of the partition coefficient (LogP),^26^ molar refractivity (MR),^26^ Labute’s approximate surface area (LabuteASA),^27^ and topological polar surface area (TPSA)^28^ were calculated to obtain fragment-level descriptors. In addition, MORFEUS descriptor^29^ and electrostatic potential descriptors calculated using Multiwfn^30^ from structures optimized at B3LYP/def2-SVP^31^ level were included. To minimize redundancy among all the descriptors, highly correlated features were identified and removed manually. Details of each descriptor are provided in Table S1.

**Figure 3:**
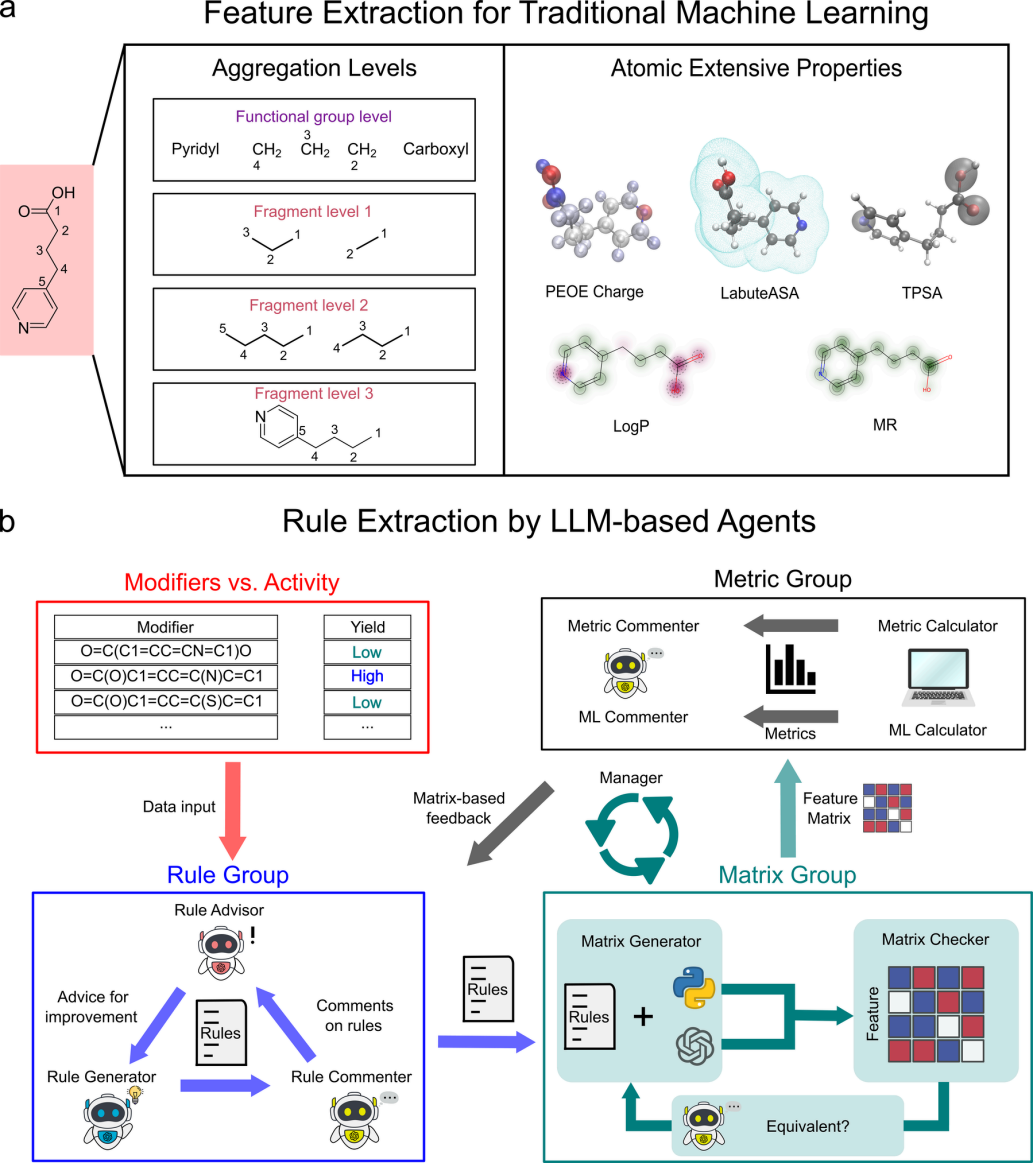
(a) Pooling process for generating molecular descriptors. The LoFFi descriptor combines RDKit properties, such as Marsilli–Gasteiger’s partial equalization of orbital electronegativities (PEOE) charge, logarithm of the partition coefficient (LogP), molar refractivity (MR), Labute’s approximate surface area (LabuteASA), and topological polar surface area (TPSA), using pooled values at different function group levels. A new series of descriptors was developed by pooling these properties at fragment levels, defined by molecular structure and fragment length (Fragment levels 1–3). (b) The process begins with the data input of SMILES structures and target values. In the Rule Group, the Rule Generator creates rules describing substructure combinations, which are reviewed by the Rule Commenter and refined with guidance from the Rule Advisor. Refined rules are converted into a feature matrix by the Matrix Group, where the Matrix Generator encodes the rules into a Python function, and the Matrix Checker validates accuracy. The resulting matrix is assessed by the Metric Group using statistical metrics (e.g., support, confidence, lift, leverage), with further machine learning metrics (5-fold validation accuracy and SHAP values) evaluated by the ML Calculator. Feedback loops between groups, coordinated by the Manager, ensure iterative refinement of rules and matrices until optimal performance is achieved.

The yields of the reactions were assigned to “high” or “low” based on whether they were above the median value. Classification using a random forest model was tested. To ensure robust model performance and avoid overfitting, Leave-P-Out splitting was used to generate 50 train-test sets (P = 4 with 2 “high” yield + 2 “low” yield for the test set), and leave-one-out (LOO) evaluation was performed on each train set for forward feature selection. Each training was performed with nine random seeds to obtain statistically reliable results. The frequency of each feature appearing among the top three features in the selected feature sets of the 50 splits was counted to identify the optimal feature combination.

The best ML result showed a yield prediction accuracy of 0.812, suggesting that:

(1) Fe-loading is the most critical feature to consider—the higher the loading, the higher the yield, which is quite expected.
(2) A high solvent-accessible surface area (SASA) of surface modifiers was associated with lower yields.

### Large Language Models Extracting Structural Rules

In parallel with the traditional descriptor approach, large language models (LLMs) were explored to automate the generation and refinement of structural rules for molecular modifiers, aiming to capture broader characteristics than traditional fingerprints. SMILES (Simplified Molecular Input Line Entry System), a text-based representation of chemical structures, was used as input for the rule generation. The workflow, illustrated in Figure 3b and Figure S4, involves iterative collaboration of multiple agents.

### “Rule Generator”

LLMs first generate linguistic rules from SMILES and experimental data sets through the “Rule Generator”. This agent receives background information on the catalytic reaction, key chemistry, and its task of generating rules, tapping into the integrated knowledge of the LLM. The “Rule Generator” is also notified that it is working collaboratively with other agents who will evaluate and provide feedback on these rules. Seven guidelines are set for the “Rule Generator”:

(1) Rules should illustrate direct combinations of substructures (function groups) in the modifiers. Combining multiple substructures is encouraged.
(2) Consider underlying physical-chemical properties while generating rules.
(3) Each rule should clearly predict whether the target value is high or low for any SMILES structure that fits its description.
(4) Prioritize rules that cover a broader range of the data set.
(5) Generate 5 to 15 rules.
(6) Maintain a suitable balance between simple rules with higher coverage and complex rules with lower coverage.
(7) Discard ineffective rules and create new ones as needed.

Strict output formats are enforced to maintain consistency.

### “Rule Commenter”

The generated rules are then reviewed by a “Rule Commenter”, who assesses them based on:

(1) Clarity: whether the rule unambiguously predicts high or low target values.
(2) Property Insight: the extent of physical-chemical reasoning in generating the rule.
(3) Complexity: whether the rule incorporates multiple substructures (functional groups).
(4) Coverage: ensuring each rule is supported by at least two data points.
(5) Balance: evaluating trade-offs between rule complexity and data set coverage.

### “Rule Advisor”

A “Rule Advisor” takes in the outputs from the “Rule Commenter” and provides revision guidance to the “Rule Generator”. The iterative process continues until the “Rule Advisor” deems a rule sufficiently refined or determines it should be discarded. While both the “Rule Generator” and “Rule Commenter” focus only on the current round of the rule revision, the “Rule Advisor” can oversee the history of the whole discussion to make a more literal judgment. Once finalized, the rules proceed to the matrix generation stage.

### “Matrix Generator” and “Matrix Checker”

Rules are converted into numerical feature matrices for machine learning evaluation. A “Matrix Generator” assigns matrix elements as follows:

(1) **1**: The rule applies and predicts a positive effect on the target (e.g., yield).
(2) **–1**: The rule applies and predicts a negative effect.
(3) **0**: The rule is irrelevant to the molecule.

The LLM rule extraction operates in two distinct modes:

(1) Linguistic mode: In this approach, the LLM directly processes chemical structures (provided as SMILES strings) and proposed rules using its pretrained knowledge, generating a feature matrix through natural language processing. This mode leverages the model’s inherent chemical understanding without explicit programming steps, producing an output matrix of dimensions (number of SMILES) × (number of rules).
(2) Code mode: Here, the LLM first translates generated rules into executable Python functions. These programmatic rules are then systematically applied to the SMILES data through automated script execution, generating the feature matrix computationally. This mode provides more transparent and reproducible rule implementation while maintaining the LLM’s interpretative advantages.

In the “code” mode, the “Matrix Generator” writes a tailored “rule2matrix­(rule, smiles)” function, applying each rule to each sample. The written function contains proper commentary descriptions, package imports, and error handling. The way of representing a rule by code is quite flexible (Figure 4a,b). Packages like the chemical informatics module “RDkit” are implemented to examine complex chemical structure relationships or calculate some chemical properties of a molecule for judgment. In addition, SMARTS (SMiles ARbitrary Target Specification) representations are adopted, which use placeholders and logical operators to allow dynamic adaptation to complex and variable structural motifs. Empirical testing showed that the “code” mode achieved higher accuracy in matrix generation compared to the “linguistic” mode (89.7% vs 56.4%, implemented by o1, OpenAI). The matrix generation accuracies were calculated on the top 10 significant rules (Table S2), compared to the human-generated matrix.

**Figure 4:**
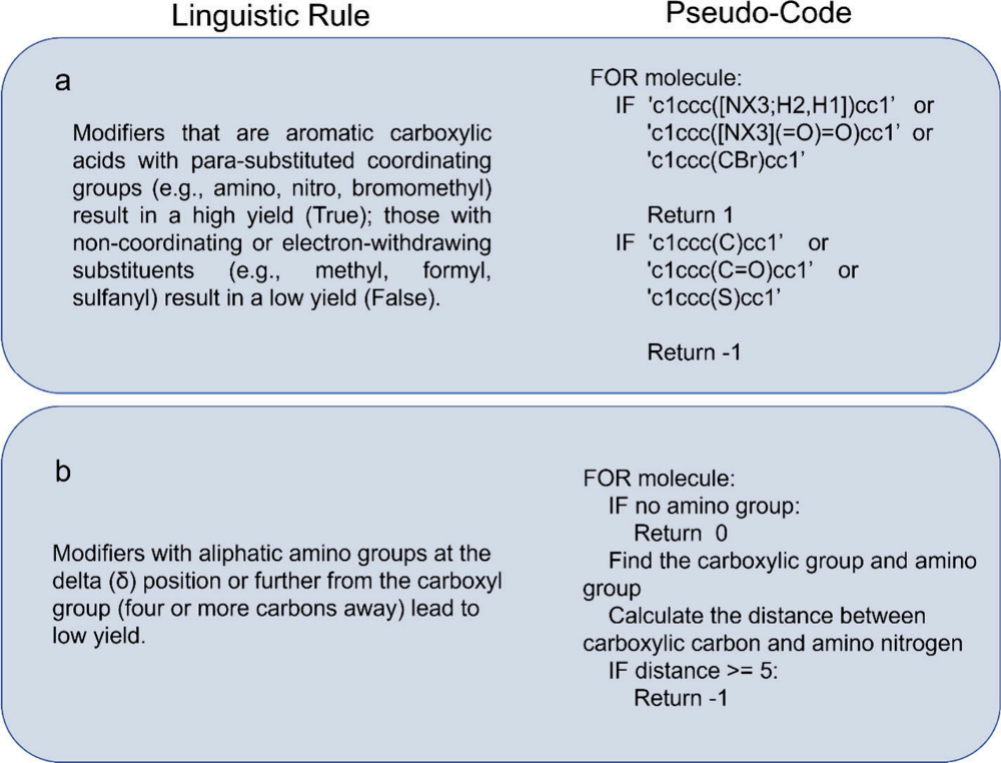
Representative examples of o1-generated rules and their corresponding code implementations. (a) An example of code implementation based on the combination of multiple SMILES strings. Modifiers that are aromatic carboxylic acids with para-substituted coordinating groups are predicted to result in a high yield. Modifiers with noncoordinating or electron-withdrawing substituents are expected to result in a low yield. (b) An example of code implementation based on logical structure recognition between molecular groups. Modifiers with aliphatic amino groups at the delta (δ) position or further from the carboxyl group are predicted to result in a low yield. The feature matrix uses values of −1, 0, and 1, where 0 indicates the rule does not apply, 1 predicts a high yield, and −1 predicts a low yield.

A “Matrix Checker” validates matrix assignment and ensures the overall matrix structure is reasonable. Feedback is provided to refine code implementation.

### “Metric Calculator” and “Metric Commenter”

A “Metric Calculator” uses preset codes to assess rule effectiveness using four statistical metrics:

(1) Support: percentage of samples that agree with the rule.
(2) Confidence: percentage of rule-supporting samples that align with experimental results.
(3) Lift: ratio of confidence to baseline prediction success.
(4) Leverage: additional rule support over random expectation.

A “Metric Commenter” analyzes these metrics across iterations, comparing past and current results to track improvement and suggest refinements. The comments are then given to the “Rule Generator” in a new iteration.

### “ML Calculator” and “ML Commenter”

The rule matrix is then used in an “ML Calculator” that applies traditional machine learning models. A 5-fold cross-validation on an extra-trees classifier was adopted for the “ML Calculator”, and accuracy and SHAP (SHapley Additive exPlanations) values are calculated as metrics.

An “ML Commenter” interprets results, identifying overfitting or underfitting, rule effectiveness, and insights from SHAP analysis, providing further revision guidance to the “Rule Generator”.

### “Project Manager”

A “Project Manager” oversees the iterative process, reviewing logs to determine when refinements should stop. It employs a leave-one-out (LOO) evaluation scheme to assess the performance of the final rule matrix, ensuring robust and meaningful rule development.

### Evaluation of LLM-Generated Rules in Predicting Catalytic Performance

Fe-loading, defined as the amount of Fe relative to the substrate, is critical, as models without this descriptor all showed low LOO accuracy below the baseline of using “Fe-loading” alone with the random forest model (0.736 ± 0.014). This trend was observed across models utilizing traditional machine learning descriptors (0.618 ± 0.016) as well as from LLM-generated rules (GPT-4o: linguistic 0.333, code 0.222; o1: linguistic 0.430, code 0.611) (Figure 5a). Notably, these tests revealed that the reasoning model o1 has a much-improved performance than GPT-4o.

**Figure 5:**
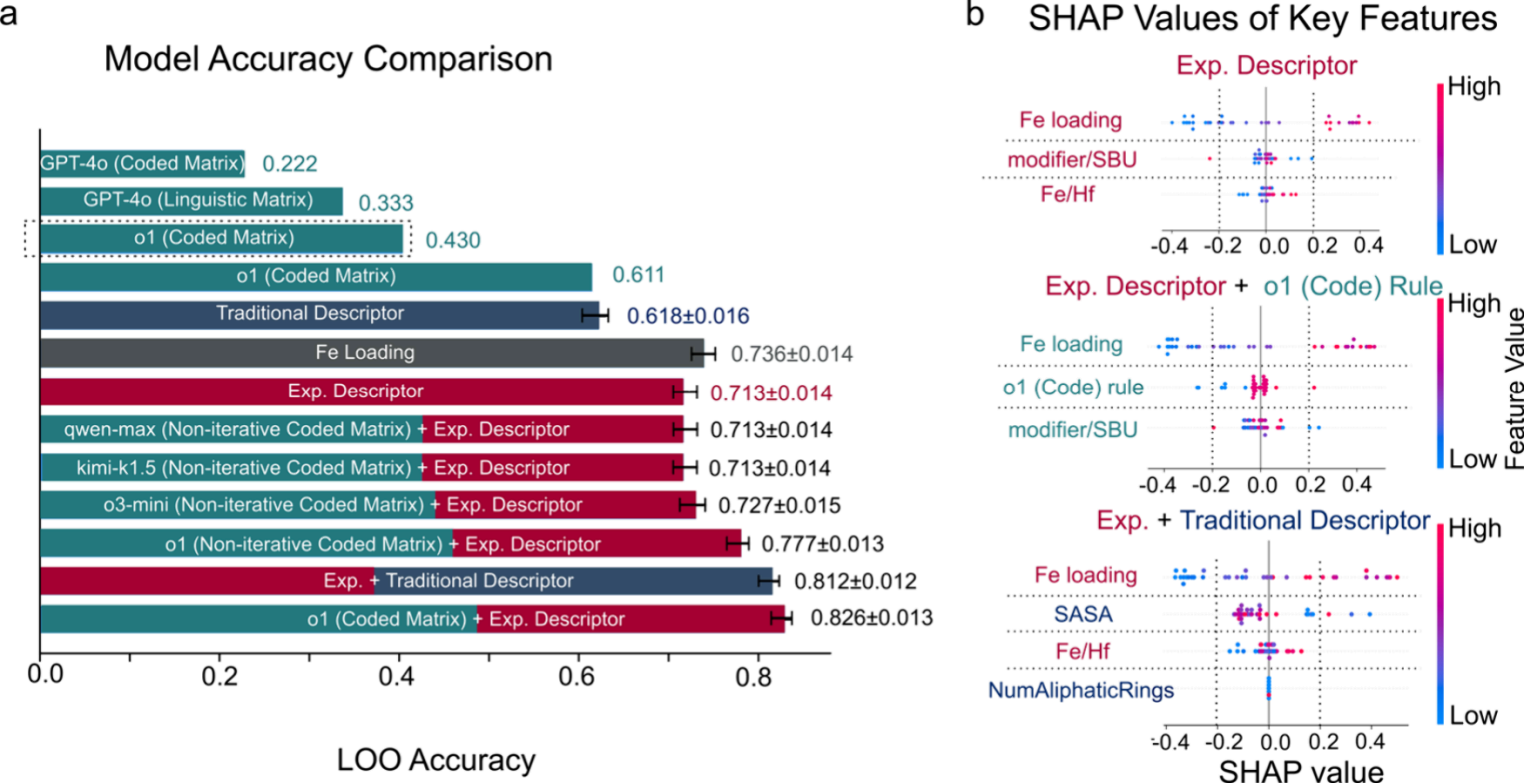
(a) Model accuracy for different descriptor sets, including experimental descriptors, traditional descriptors, and LLM-generated rules. LLMs were prompted to generate the feature matrix with the help of LLM-generated code or by explicitly reading the rules. The term ‘Coded Matrix’ refers to an iterative rule-generating pipeline that includes code generation, while ‘Linguistic Matrix’ refers to an iterative rule-generating pipeline without code generation. ‘Noniterative’ indicates that LLMs generated rules and code only once, without iteration. Various LLMs, including GPT-4o, o1, o3-mini, qwen-max, and kimi-k1.5, were utilized in this study. Error bars represent ‘mean ± standard deviation’ for accuracies calculated using a random forest model with varying random states. For all methods except the four LLM-only approaches, the leave-one-out (LOO) accuracy was evaluated using a single feature matrix across 36 train-test splits. In the case of the four LLM-only methods, accuracies were derived using 36 individual feature matrices for each train-test split during the LOO iteration. However, due to the consistently low accuracy of these four methods, random-seed standard deviations were not calculated for them. Additionally, the o1 (‘Linguistic’) accuracy was only evaluated on the first five train-test splits due to cost constraints, as indicated by the dashed block. (b) SHAP values illustrating feature importance for models using experimental descriptors (top), experimental descriptors + o1 (Code) rule (middle), and experimental + traditional descriptors (bottom). Higher SHAP values indicate stronger feature contributions to yield predictions. SASA means the solvent-accessible surface area of the modifier. Fun_LogP_Max is the max LogP value among the functional groups in a modifier.

As a result, experimental descriptors including Fe-loading, Modifier/SBU, and Fe/Hf, along with the reaction results, were provided to multiple reasoning LLMs, including o1 (OpenAI), o3-mini (OpenAI), qwen-max,^32^ and kimi-k1.5.^33^ These models were instructed to directly generate rules, code, and the corresponding feature matrix without iterations to save expense (“Non-iterative” in Figure 5a, see Table S3 for the rules). After recursive feature elimination with cross-validation (REFCV), the rules generated by qwen-max, kimi-k1.5, and o3-mini achieved accuracies of 0.713 ± 0.014, 0.713 ± 0.014, and 0.727 ± 0.015 respectively—showing no clear improvement over Fe-loading alone. In contrast, o1 outperformed the Fe-loading-only model, achieving an accuracy of 0.777 ± 0.013. Accuracies, recalls, precisions, F1 scores, and ROC-AUC scores of these models are listed in Table S4. Furthermore, iteratively refined o1’s rule set alongside the experimental descriptors improved its accuracy to 0.826 ± 0.013. Ultimately, only the most significant rule identified via SHAP analysis was retained in the final model (Table S2):

### Para-Substituted Benzoic Acids with Electron-Withdrawing, Metal-Coordinating Groups Give High Yields, While Electron-Donating or Noncoordinating Substitution Groups Give Low Yields

Considering the importance of the existence of Cl^–^, we provide the following explanation:

The electronic effects of para-substituents on the modifiers exert precise control over the hydrogen atom transfer (HAT) efficiency by modulating the Fe–Cl ligand-to-metal charge transfer (LMCT) process. Our studies reveal a clear structure–activity relationship: para-substituted benzoates with electron-withdrawing groups (EWGs) or metal-coordinating groups consistently yield superior activity, while electron-donating groups (EDGs) or noncoordinating substituents underperform. This trend originates from the substituents’ ability to tune the electron density at the Fe^3+^ center through the conjugated Hf-carboxylate framework.

EWGs enhance catalytic performance through two synergistic mechanisms: (1) they increase the Fe^3+^ center’s electron deficiency, lowering the LMCT energy barrier and promoting Cl• radical generation, and (2) they strengthen Fe–Cl bond polarization, facilitating homolytic cleavage. Conversely, EDGs diminish these effects, resulting in slower LMCT kinetics. Notably, metal-coordinating groups. Notably, the metal-coordinating group, like amines, can coordinate to dissociative Fe^3+^ or get protonated, thereby transforming into electron-withdrawing groups.

Structural analysis confirms this electronic property: the para-substituents positioning ensures optimal electronic coupling through π-conjugation while avoiding steric conflicts with the active site. Crucially, the modifiers’ carboxylates and TPY ligands coordinate to shared Hf^4+^ nodes, creating an efficient pathway for electronic effects while physically separating substituents from the Fe center (Figure 6). This arrangement excludes direct interaction between substituents and reaction intermediates/transition states, confirming the primacy of electronic over steric effects in governing activity.

**Figure 6:**
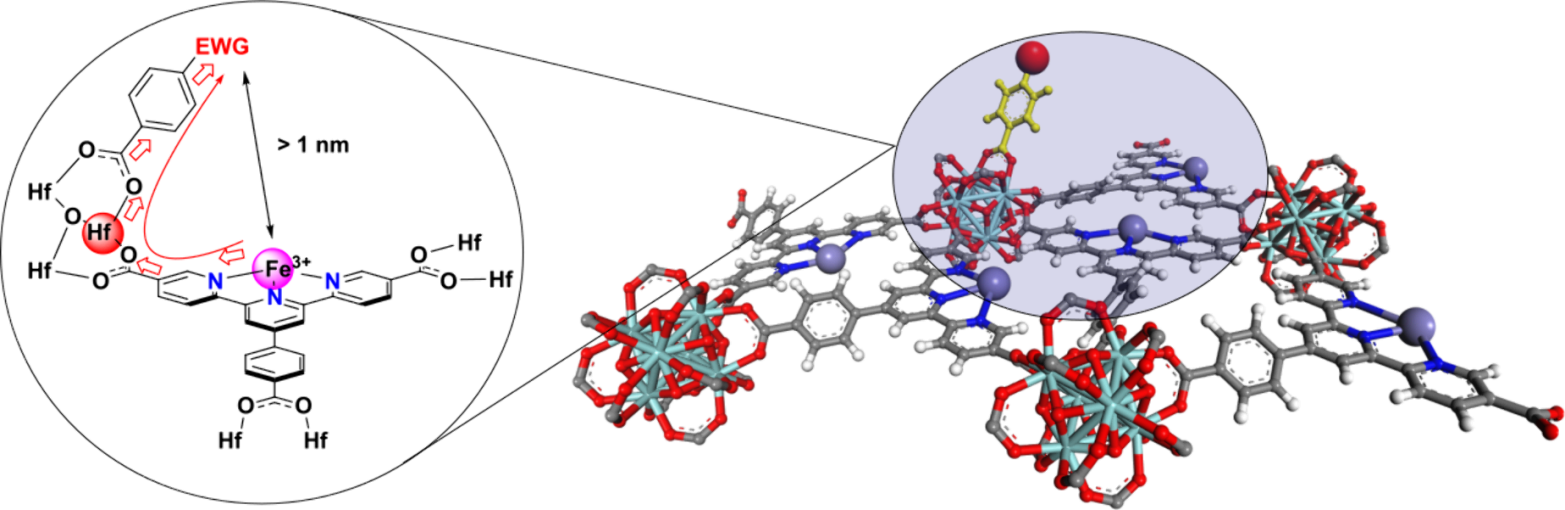
Local structure of the modifier and the Fe^3+^ active center showing the transduction path of the electron-withdrawing effect.

As a result, these analyses emphasized the importance of the LMCT process between Fe^3+^ and Cl^–^, providing guidance for improving the yield of the original sp^3^ C–H bond activation.

At the same time, the thermodynamic preference of Hf^4+^ for carboxylate binding, the sequential experimental preparation method ensuring precoordination to Hf nodes, and steric hindrance from the terpyridine plane collectively reduce the possibility of secondary coordination with Fe^3+^ centers. These factors indicate that the electronic effects from para-substituents on benzoates are primarily transmitted through the Hf-carboxylate framework, other than any secondary coordination.

### Analysis of the Cooperative Rule Generation and Refinement Process

We can track the generation and refinement process of the above rule (Table 2, original prompts and outputs are shown in Figures S5–35). The “Rule Generator” initially proposed that electron-withdrawing groups (EWGs) give high catalytic performance, while electron-donating groups (EDGs) and aldehydes produce low yields. The “Rule Commenter” found this clear but soon discovered contradictions, such as classifying amino (an EDG) as high yield.

**Table 2:** Iterations in Refining a Rule

Steps	Agent	Response Summary	Current Rule or SMARTS
1	Rule Generator	Set the initial rule and give supportive examples of the rules:	**Rule 2**: **para-substituted benzoic acids with electron-withdrawing groups yield high**
		*Examples:*	
		High Yield:	
		4-(bromomethyl)benzoic acid	
		4-nitrobenzoic acid	
		Low Yield:	
		4-methylbenzoic acid	
		4-formylbenzoic acid	
2	Rule Commenter	Found Rule 2 clear and in line with the data	-
3	Rule Advisor	Suggested broader coverage or merging with similar rules	-
4	Rule Generator	Added EDG/bulky group references	**Para-substituted benzoic acids with electron-withdrawing groups yield high; electron-donating or bulky groups yield low**
		*Examples:*	
		High Yield:	
		4-nitrobenzoic acid	
		4-(bromomethyl)benzoic acid	
		4-aminobenzoic acid	
		Low Yield:	
		4-methylbenzoic acid	
		4-formylbenzoic acid	
		4-pyridin-4-ylbenzoic acid	
5	Rule Commenter	Flagged inconsistency: amino (an EDG) contradicts the EWG requirement	-
6	Rule Advisor	Recommended focusing on metal-coordination and combining steric/electronic effects rather than the EDGs and EWGs	-
7	Rule Generator	Revised the rule to prioritize metal-coordinating groups (e.g., NH_2_, NO_2_, BrCH_2_) at para positions	**Rule 2**: **para-substituted benzoic acids with metal-coordinating groups yield high; noncoordinating groups yield low**
		*Examples:*	
		**High Yield:**	
		4-aminobenzoic acid	
		4-nitrobenzoic acid	
		4-(bromomethyl)benzoic acid	
		**Low Yield:**	
		4-methylbenzoic acid	
		4-formylbenzoic acid	
8	Rule Commenter	Approved updated Rule 2	Sent the rule to Matrix Generator
9	Matrix Generator	Identified amino, nitro, and bromoethyl groups as coordinating groups and methyl as a noncoordinating group	'c1ccc(cc1[NH2])C(=O)[OH]', #amino
			'c1ccc(cc1[N+](=O)[O-])C(=O)[OH]', #nitro
			'c1ccc(cc1CBr)C(=O)[OH]', #bromomethyl
			('c1ccc(cc1C)C(=O)[OH]'), #methyl
10	Matrix Checker	Pointed out the problem with the para position and failure in identifying some substitution groups	-
11	Matrix Generator	Added the aldehyde group to the set of noncoordinating groups	'c1ccc(cc1[NH2])C(=O)[OH]', #amino
			'c1ccc(cc1[N+](=O)[O-])C(=O)[OH]', #nitro
			'c1ccc(cc1CBr)C(=O)[OH]', #bromomethyl
			'c1ccc(cc1C)C(=O)[OH]', #methyl
			'c1ccc(cc1C=O)C(=O)[OH]', #formyl
12	Matrix Checker	Pointed out that hydroxyl and sulfhydryl groups should be recognized as coordinating groups	Matrix Generator encounters an error when running the code for rule2matrix
13	Rule Advisor	Proposed merging Rule 2 with Rule 9 for metal-coordination and conjugation	-
14	Rule Generator	Combine the two rules	**Rule 2**: **para-substituted benzoic acids with electron-withdrawing, metal-coordinating groups yield high; electron-donating or noncoordinating groups yield low**
		*Examples:*	
		**High Yield:**	
		4-aminobenzoic acid	
		4-nitrobenzoic acid	
		4-(bromomethyl)benzoic acid	
		**Low Yield:**	
		4-methylbenzoic acid	
		4-formylbenzoic acid	
		4-sulfanylbenzoic acid	
15	Rule Commenter	Approved final Rule 2	-
16	Rule Advisor	Recommended to clarify and correct Rule 2 regarding metal coordination	The rule is revised over three times (threshold) with limited improvement in the last round of revision; pass to the next stage
17	Matrix Generator	Identified positive yield predictions for amino, nitro, and bromomethyl groups and negative predictions for other para-substituents of benzoic acid	'[NX3;H2,H1]', #primary or secondary amine
			'[NX3](=O)=O', #nitro group
			'CBr', #bromomethyl group
18	Matrix Checker	Ensured correct identification of para-substituted benzoic acids with coordinating groups	Sent to metrics calculations
19	Metric Calculator	Calculated support, confidence, lift, and leverage for the entire rule set	-
20	Metric Commenter	Provided overview; no specific mention of Rule 2	-
21	ML Calculator	Checked Rule 2’s SHAP values for consistent yield trends	-
22	ML Commenter	Identify potential underestimation and overestimation cases	-
		Clarify electron-withdrawing groups, differentiate between substituents, and consider the effect of substituents at positions other than para to fix the inconsistency between Rule 2 and some samples	
23	Project Manager	Noted para vs meta/ortho concerns; asked for final improvements	
24	Rule Advisor	Concluded limited further gains; accepted current Rule 2	**Final Rule 2**: **para-substituted benzoic acids with electron-withdrawing, metal-coordinating groups yield high; electron-donating or noncoordinating groups yield low**

The critical moment of the refinement comes at the sixth step when the “Rule Advisor” realized that it might not be appropriate to consider EWGs or EDGs in this case, but worth exploring a shifted focus on coordination.

Once the rule shifted to highlight metal-coordinating modifiers, the “Matrix Generator” converted it into SMARTS patterns, which the “Matrix Checker” refined to account for a variety of functional groups (e.g., – OH, −SH, and aldehydes). Along the way, the “Metric Calculator” and “ML Calculator” tested the rule’s predictive accuracy via confidence, lift, support, machine learning performance, and SHAP analysis. In the final steps, the “Project Manager” and “Rule Advisor” concluded that further changes yielded diminishing returns, and the rule was accepted in its current form, faithfully capturing the reactivity trend.

## Conclusion

We investigated the potential of large language models (LLMs) for quantitative structure–activity relationship (QSAR) analysis in a small data set modifying the surfaces of metal–organic layers (MOLs) to enhance their catalytic activity in C–H bond activation. An LLM-assisted rule-based machine learning (ML) pipeline was compared with traditional descriptor-based approaches. The LLM-generated rules exhibited versatility and interpretability—being low-dimensional, problem-specific, and capable of iterative refinement—enhancing prediction accuracy when combined with experimental descriptors. Only one rule, in combination with experimental features such as Fe-loading, is sufficient for the prediction. The rule showed that the electronic effect of the modifier, instead of its direct interaction with the intermediate/transition state, is critical: benzoate para-substituted by the electron-withdrawing group is a booster for catalytic performance, while the electron-donating group disfavors the activity.

Considering the transferability of our LLM-based framework, the approach demonstrates notable versatility across diverse catalytic systems due to its foundation on SMILES representations. The system’s adaptability primarily requires two modifications: (1) customization of prompts to reflect new reaction systems, and (2) incorporation of domain-specific details about the target reaction process. This flexibility is particularly advantageous for research scenarios involving moderate-sized data sets (∼100 samples) where conventional structure–activity relationships can be elusive.

Despite these strengths, several limitations remain. First, the refinement of LLM-generated rules lacks a rigorous mathematical foundation, unlike gradient-based methods such as gradient descent. As a result, iterative updates do not consistently lead to improvement. Second, most LLMs rely on textual input and struggle with heterogeneous data formats, limiting their ability to process complex experimental data sets. This limitation is further compounded by token constraints (e.g., 200,000 for o1), which restrict the amount of data or contextual information that can be handled in the iterative process. While the development of multimodal models or AI agents equipped with specialized tools offers a promising path forward, further research and validation are needed.

This synergy between LLMs and traditional ML tools represents a promising pathway for enhancing the predictive capabilities and mechanistic understanding of intricate chemical systems, particularly in the field of heterogeneous catalysis. Looking ahead, future research should explore the scalability of this hybrid approach to larger data sets and more diverse chemical systems. The integration of domain knowledge, deductive reasoning, and the creative potential of LLMs with the robust validation of traditional ML techniques offers significant promise for advancing predictive power in complex systems. Further progress could be achieved by improving LLM protocols, refining feature extraction methods, and enhancing the interoperability of LLM-generated rules with other ML frameworks. We expect significant progress using this approach as the reasoning models and agentic AI continue to improve, revolutionizing chemical research in the near future.

## Supplementary Material





## Data Availability

The codes used in this study are publicly available under the MIT License in the GitHub repository: https://github.com/Wang-Group/Reasoning-Language-Model-as-Rule-Finder. Experimental data supporting the findings in this paper are available at Figshare through DOI: 10.6084/m9.figshare.28596839.

## Notes

Additional experimental details, materials, feature sets, machine learning results, and H^1^ NMR spectra for all the experiments (PDF)
Transparent Peer Review report available (PDF)
