# Supplementary material for: Reasoning Language Model as Rule Finder: A Case Study on C–H Bond Activation Using 2D Metal–Organic Frameworks
Source: ACS Cent Sci. 2025 Jun 13;11(7):1135–46. doi: 10.1021/acscentsci.5c00561 (PMC12291106; doi:10.1021/acscentsci.5c00561)
Supplement: Supplementary file 1 [file oc5c00561_si_001.pdf]

Supporting Information

Reasoning Language Model as Rule Finder: A Case Study on C–H Bond Activation Using 2D Metal-Organic Frameworks

He Lin[1†], Xiaoqi Cui[1†], Binglin Dai[1], Jiawei Chen[1], Pengkun Su[1], Zhaomin Su[1], Huihui Hu[3]*, Yibin Jiang[1,2,4]*, Cheng Wang[1,2]*

[1]iChem, State Key Laboratory of Physical Chemistry of Solid Surfaces, College of Chemistry and Chemical Engineering, Xiamen University, Xiamen 361005, P. R. China.

[2]Innovation Laboratory for Sciences and Technologies of Energy Materials of Fujian Province (IKKEM), Xiamen 361005, P. R. China.

[3]Jiangsu Key Laboratory for Science and Applications of Molecular Ferroelectrics, Southeast University, Nanjing 211189, P. R. China.

[4]15 Church Street, Glasgow G11 5JP, the United Kingdom.

[†]These authors contributed equally.

*Corresponding author email: huhuihui@seu.edu.cn, yibin_jiang@outlook.com, wangchengxmu@xmu.edu.cn.

Supplementary information

1. Reagents and instruments.......2

2. Mechanism Studies.......4

3. Substrate expansion.......6

4. Feature sets.......7

5. Discussion about o1-generated rules for yield.......8

6. Rules generated by other LLMs for yields.......11

7. Classification scores of different methods.......13

8. Hyperparameters for machine learning modules.......13

9. Prompts of the iterative LLM pipeline.......15

10. Outputs of the iteration of the selected rule.......23

11. Traditional and LLM results on Fe/Hf.......65

12. Two distinctive fingerprints from MACCS for Fe/Hf.......67

13. Dataset.......68

14. ^{1}H-NMR and GC-MS data.......72

1. Reagents and instruments

Chemicals: Hafnium(IV) chloride (99.9%, $HfCl_4$), 1,3,5-Tris(4-carboxyphenyl)benzene (H_3BTB) and formic acid (HCO_2H, 99%) were purchased from J&K Scientific Co. and Ltd. N, N'-dimethylformamide (DMF), acetone, acetonitrile, tetrahydrofuran, dibromo methane, methanol was purchased from Sinopharm Chemical Reagent Co. N_2 (99.999%) was purchased from Fuzhou Xinhang Industrial gases Co., Ltd. Reagents were commercially available and used without further purification unless otherwise stated.

Characterization: X-ray Powder Diffraction (PXRD) was performed using a Rigaku IV XRD system equipped with Cu-Kα radiation at a wavelength of 1.54 Å. Gas Chromatography-Mass Spectrometry (GC-MS) analysis was conducted on a Shimadzu GCMS-QP2010 Plus instrument under a nitrogen atmosphere, with a temperature range of 40 to 290 °C. Transmission Electron Microscopy (TEM) examination was carried out using a JEM1400 microscope. Sample preparation involved wet dispersion in ethanol, ultrasonication for 20 minutes, deposition onto a copper grid, drying by evaporation on filter paper, followed by testing. Hydrogen Nuclear Magnetic Resonance Spectroscopy (1H-NMR) spectra were recorded using Bruker Avance II 400 M and 500 M instruments. Thermogravimetric Analysis (TGA) was performed on a Netzsch TG209F1 analyzer under an air atmosphere, using an Al2O3 crucible, with temperatures ranging from 40 to 600 °C. Prior to Inductively Coupled Plasma Optical Emission Spectroscopy (ICP-OES) analysis, samples were digested and diluted using equipment from Agilent Technologies. Ultraviolet-visible spectrophotometry (UV-Vis) measurements were taken using an Agilent Carry 60 UV-Vis Spectrophotometer.

2. Mechanism Studies

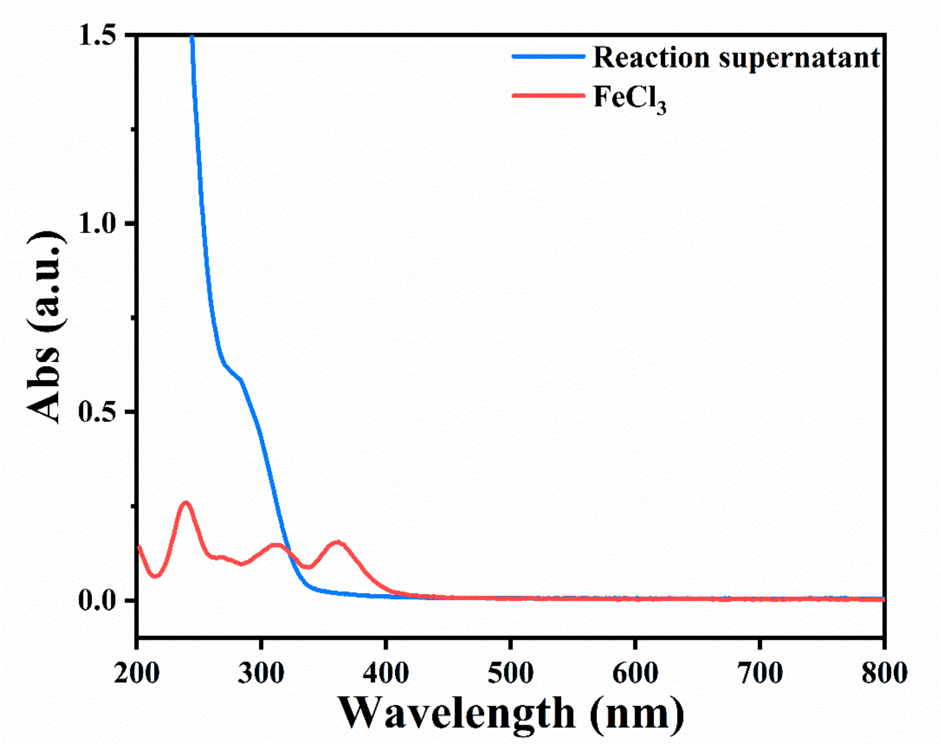

Figure S1. UV-Vis spectra of the supernatant after reaction (blue) and $FeCl_3$ (red). The fact that no observable Fe^{3+} signals in the reaction supernatant excludes the influence of free Fe^{3+}.

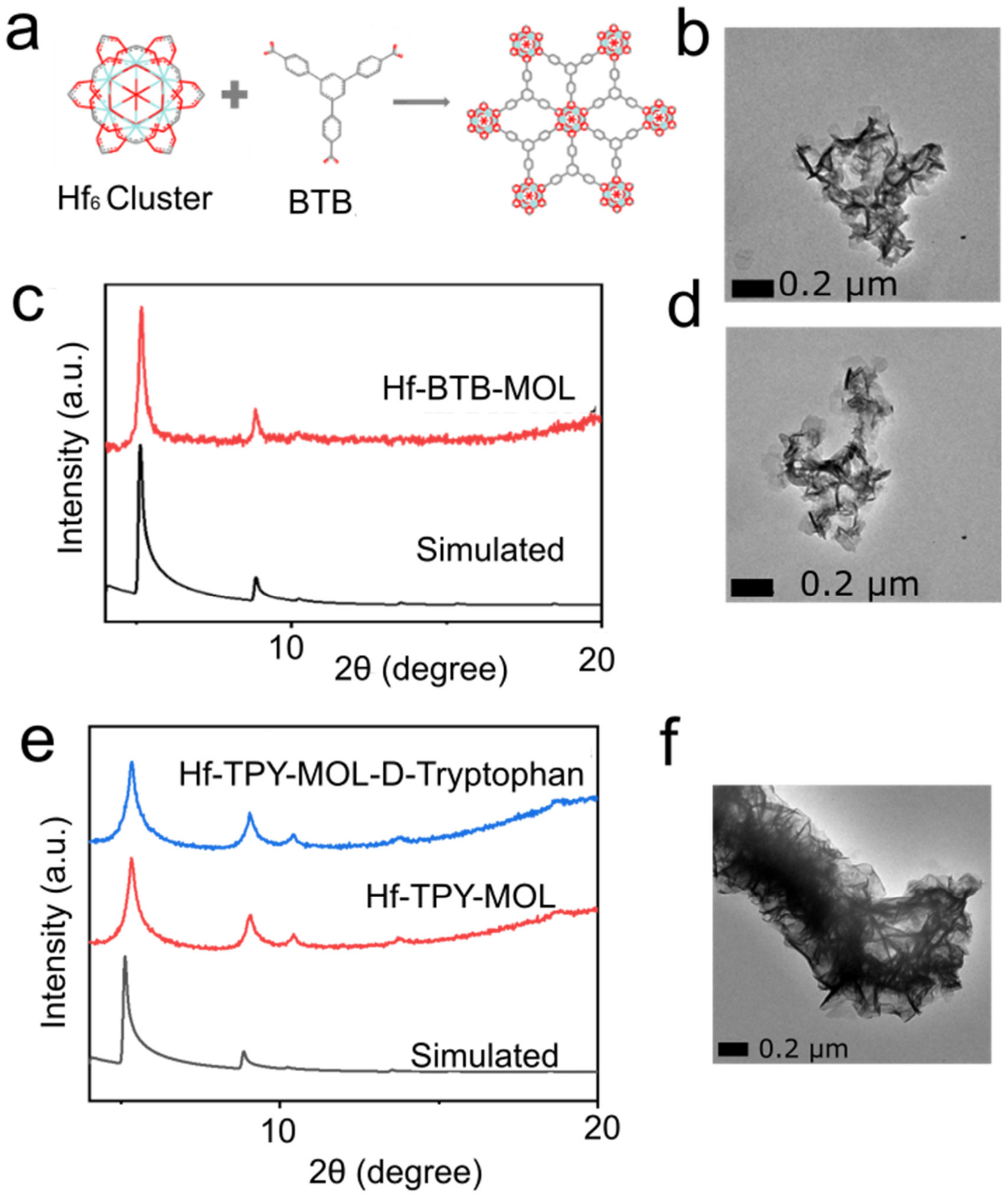

Figure S2. **(a)** Structure of Hf-BTB-MOL. **(b)** Simulated (black) and experimental (red) PXRD patterns of Hf-TPY-MOL. **(c, d)** The TEM pattern of Hf-BTB-MOL. **(e)** Simulated and experimental PXRD patterns of Hf-TPY-MOL and Hf-TPY-MOL-D-Tryptophan. **(f)** TEM image of Hf-TPY-MOL-D-Tryptophan.

3. Substrate expansion

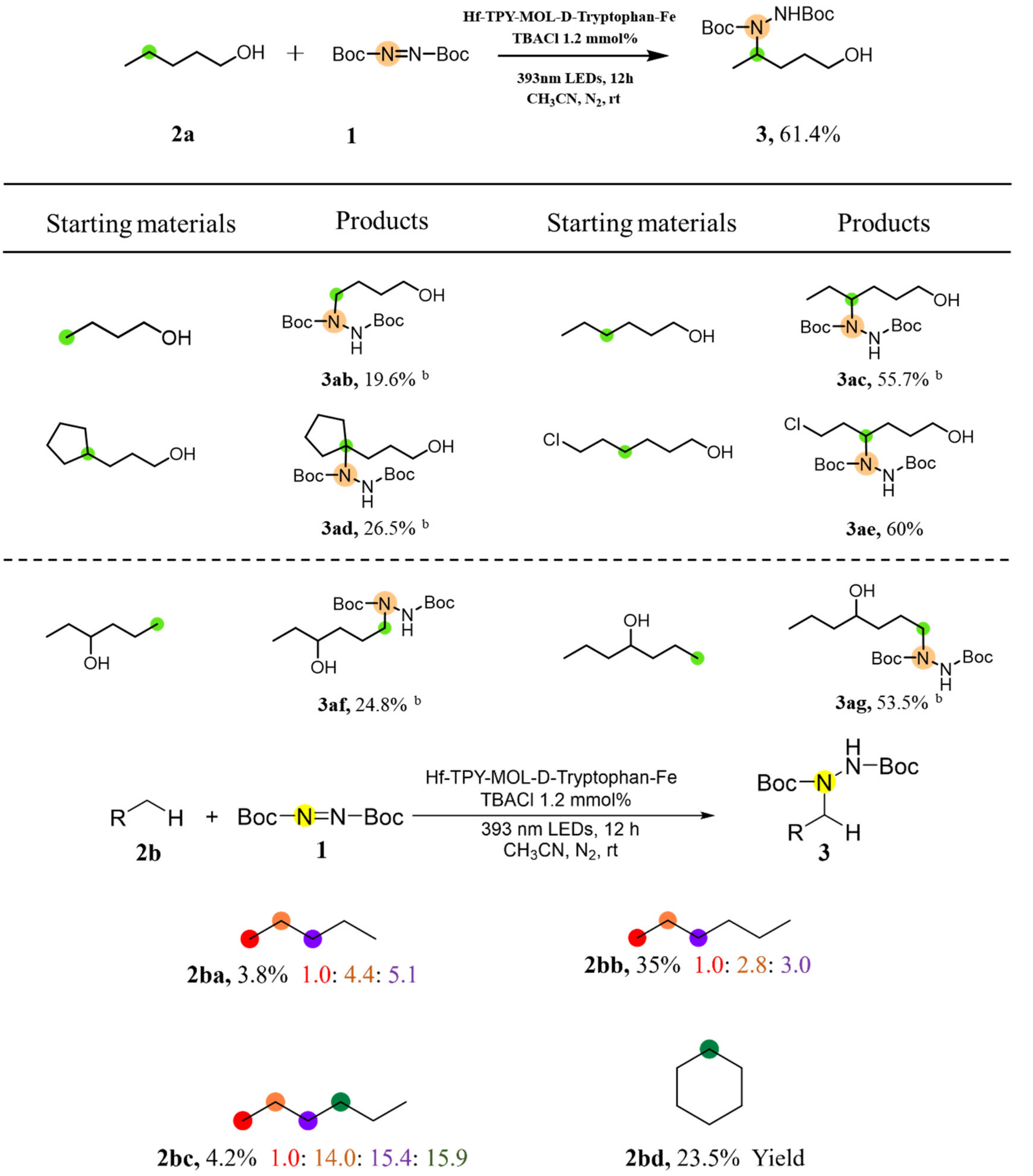

Figure S3. Substrate expansion for C–H bond activation reaction.

In addition to 1-pentanol, we also explored the C–H bond activation reaction with other alcoholic substrates such as 1-butanol, 3-cyclopentyl-1-propanol, and 3-hexanol. The reactions utilized 0.6 mg of Hf-TPY-MOL-D-Tryptophan-Fe as the photocatalyst, along with DBAD (1 eq.), the alcoholic substrate (3.0 eq.), and 5 μmol of TBACl as an additive. Reactions were conducted under a nitrogen atmosphere with 393 nm LED illumination at room temperature for 12 hours, except for 1-butanol, which required 24 hours. Yields were characterized relative to DBAD using

^{1}H-NMR spectroscopy. The yields for 1-hexanol, 6-chloro-1-hexanol, and 4-heptanol were 55.7%, 60%, and 53.5%, respectively, comparable to the 61.4% yield obtained with 1-pentanol. However, the yields for 1-butanol, 3-cyclopentyl-1-propanol, and 3-hexanol were significantly lower, at 19.6%, 26.5%, and 24.8%, respectively.

While the selectivity for the δ position was nearly 100% for the alcohol substrates mentioned, no such selectivity was observed for alkane substrates. The yields for the alkane substrates were also low, with pentane, hexane, heptane, and cyclohexane yielding 3.8%, 35%, 4.2%, and 23.5%, respectively. The reaction conditions for the alkane substrates were identical to those used for the alcohol substrates, except that the reaction time was extended to 24 hours.

These substrate expansion experiments underscore the importance of the hydroxyl group for achieving δ-position selectivity in these reactions.

4. Feature sets

Table S1. The definition of different descriptors.

**Feature Name**	**Meaning**
MaxEstateIndex – MolMR	24 RDkit features
Atom_PEOE-Charge_Mean – Conju_LogP_Min	The 45 LoFFi pooling features are calculated using RDKit. They include PEOE charge, LogP, and molecular refractivity (MR) values, aggregated through pooling methods (max, min, mean, sum, standard deviation) across various levels: atom, functional group, connected functional groups, and conjugated systems.
frag_level_0_PEOE-Charge_Mean<br>frag_level_0_LogP_Mean<br>frag_level_0_MR_Mean<br>frag_level_0_TPSA_Mean<br>frag_level_0 _LabuteASA_Mean<br>frag_level_1_PEOE-Charge_Mean<br>frag_level_1_LogP_Mean<br>frag_level_1_MR_Mean<br>frag_level_1_TPSA_Mean<br>frag_level_1_LabuteASA_Mean<br>frag_level_2_PEOE-Charge_Mean<br>frag_level_2_LogP_Mean	All fragments in a molecule are extracted, with their lengths defined by the number of bonds starting from the carbon in the carboxylic group. Based on these lengths, fragments are categorized into three levels. The mean values of PEOE charge, LogP, MR, topological polar surface area (TPSA), and Labute ASA are then calculated for each level.

frag_level_2_MR_Mean<br>frag_level_2_TPSA_Mean<br>frag_level_2_LabuteASA_Mean	
Longest_fragment_length (No H)	The longest length of all the fragments, ignoring H.
Surface_area – sterimol_B5	10 Morfeus features
HO_Estateidx	Estate index on the hydroxyl oxygen in the carboxylic group.
HO_PEOE	PEOE charge on the hydroxyl oxygen in the carboxylic group.
HO_phi (0.001 au)	Mean electrostatic potential on the van der Waals surface of the hydroxyl oxygen in the carboxylic group.
O_phi (0.001 au)	Mean electrostatic potential on the van der Waals surface of the ketonic oxygen in the carboxylic group.

The feature file is provided on https://github.com/Wang-Group/Reasoning-Language-Model-as-Rule-Finder/tree/main/agent/data/del_features.csv.

5. Discussion about o1-generated rules for yield

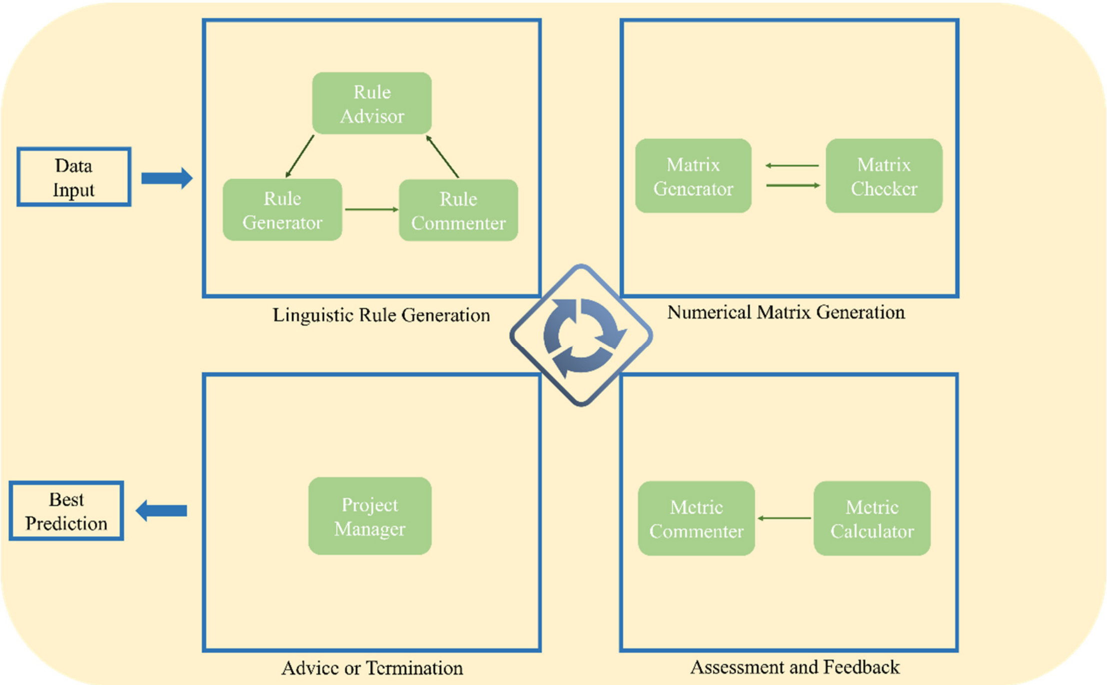

Figure S4. Four main components of the LLM pipeline. The Rule Generator, Rule Commenter, and Rule Advisor work collaboratively to develop and refine linguistic rules. Subsequently, the Matrix Generator produces feature matrices, which are then evaluated for effectiveness by the Matrix Checker. Performance metrics are calculated by the Metric Calculator and assessed by the

Metric Commenter to evaluate overall performance. The Project Manager collects feedback to either improve the rules or finalize the most accurate prediction. If feedback is provided, the Rule Advisor incorporates it and offers further guidance. Note that for o1, it cannot fix the format error raised by the matrix generation through code. Instead, it regenerates the rules whenever a format error occurs, rather than addressing the code issue.

In this section, we explore the flexibility and constraints involved in generating a feature matrix through a pathway comprising three distinct processes using OpenAI o1. This pathway begins with the generation of linguistic rules, which serve as the foundational framework for subsequent steps. These linguistic rules are then utilized to produce SMARTS-based code, which, in turn, facilitates the transformation of molecular SMILES representations into a comprehensive feature matrix. By examining the details of these rules and their corresponding code, we can gain a deeper understanding of the performance of OpenAI o1 within this pipeline.

This highlights the remarkable ability of large language models (LLMs) to effectively extract and utilize structural information from the dataset. However, for more complex rules, the generated code may fail to fully capture the rules accurately, and the underlying chemical insights might not always be apparent. The rules with the top 20 significant SHAP values are presented in Table S2.

Table S2. SHAP value of top LLM rules for yield.

o1-generated Rules	SHAP value
Para-substituted benzoic acids with electron-withdrawing, metal-coordinating groups yield high; electron-donating or non-coordinating groups yield low.	-0.027
Modifiers that are **aromatic carboxylic acids with electron-donating groups at the para position** (excluding amino groups) result in a **low yield**. Electron-donating groups decrease the acidity of the carboxylate, weakening coordination with the metal center.	-0.023
Aromatic carboxylic acids with electron-withdrawing substituents at the para position (excluding aldehydes) lead to high yield.	-0.019
Modifiers containing **thiol groups on aromatic rings** result in **low yield** due to steric hindrance and reduced thiol accessibility.	0.015
Modifiers that are aliphatic carboxylic acids with polar functional groups (e.g.,	0.014

hydroxyl, amino) on longer chains (six carbons or more) lead to low yield.	
Modifiers that **contain amide bonds involving the amino group or near the carboxyl group** predict a **low yield**.	0.014
Modifiers containing **thiol groups** lead to varying yields depending on their position: **thiol groups on aliphatic chains** result in **high yield**, while **thiol groups on aromatic rings** result in **low yield**.	0.014
Aromatic carboxylic acids with an amino group (-NH_2) at the para position**, such as **4-aminobenzoic acid**, tend to result in **high yield**	-0.014
Modifiers that include sulfur-containing groups, such as sulfanyl or thioether groups (e.g., 4-sulfanylbenzoic acid), generally produce a **low yield**. Sulfur atoms may bind strongly to the Fe catalyst, potentially deactivating it.	0.014
Modifiers that are aromatic amino acids with electron-rich heterocyclic side chains (e.g., indole ring) lead to high yield, while those with simple aromatic side chains lead to low yield.	-0.013
Modifiers containing a **sulfhydryl group (–SH) on a short aliphatic chain (three carbons or fewer)** are associated with **high yield**, possibly due to the thiol group enhancing metal coordination. However, when the sulfhydryl group is **attached directly to an aromatic ring**, they are associated with **low yield**, potentially due to steric hindrance or electronic effects.	0.013
Modifiers that are **carboxylic acids with electron-withdrawing groups (e.g., formyl (-CHO)) directly attached to the aromatic ring** will lead to **low yield**.	0.013
Modifiers that are aromatic carboxylic acids with electron-withdrawing substituents at the para position tend to result in _high_ yield.	0.013
Modifiers that are **aromatic carboxylic acids with aldehyde groups attached to the ring** predict a **low yield**.	0.012
Modifiers that are aromatic carboxylic acids with electron-donating substituents at the para position tend to result in _low_ yield.	0.012
Modifiers that are **aromatic carboxylic acids with para-substituted amino (-	-0.012

NH_2) or hydroxyl (-OH) groups** will lead to **high yield**.	
Modifiers containing a **carboxylic acid group** and an **aldehyde group** tend to give a **low yield**.	0.012
Modifiers containing **aldehyde (formyl)** functional groups on an aromatic ring lead to a **low yield**.	0.011
Modifiers that are aromatic carboxylic acids substituted with electron-donating groups at positions that do not enhance metal coordination tend to result in low yield.	-0.011
Modifiers that are aromatic carboxylic acids with aldehyde (formyl) groups lead to low yield.	0.010

The most significant SHAP value is -0.043 (Fe loading), and the least significant SHAP value is 0.000. These rules do not produce duplicate matrix columns due to subtle differences in code implementation.

6. Rules generated by other LLMs for yields

We also explore the direct generation of rules and codes by LLMs without employing an iterative process. Given the significant classification capability of Fe loading in relation to yield, we test these rules in combination with Fe loading (Table S3)

Table S3. Rules generated by various LLMs without the iteration process.

Rules	LLM
High Yield: Modifiers containing para-substituted benzoic acids with substituents –Br, $–NO_2$, $–NH_2$ at the para position.	o1
Low Yield: Modifiers containing para-substituted benzoic acids with substituents –CHO, –SH at the para position.	o1
High Yield: Simple aliphatic carboxylic acids without additional polar functional groups near the metal site.	o1
High Yield: Modifiers with thiol groups (-SH) far from the carboxyl group (e.g., in aliphatic chain).	o1
Low Yield: Modifiers with multiple polar functional groups or strongly	o1

coordinating moieties (e.g., PEG-like chains).	
High Yield: Modifiers are amino acids or related molecules (e.g., aspartic acid).	o1
Low Yield: Modifiers that directly coordinate Fe (e.g., phenolate, –SH on aromatic ring, –CHO).	o1
Low yield (-1): Modifiers containing a thiol group (-SH).	o3-mini-high
Low yield (-1): Modifiers containing an aldehyde group (-CHO).	o3-mini-high
Low yield (-1): Modifiers containing polyether-type structures (chains of ethoxy units).	o3-mini-high
High yield (+1): Modifiers that are simple carboxylic acids, whether aliphatic or aromatic with modest substituents (e.g., halogen or nitro groups).	o3-mini-high
High yield (+1): Modifiers that are amino acid derivatives (contain both amine and carboxylic acid groups).	o3-mini-high
High yield (+1): Modifiers containing aromatic rings with electron-withdrawing groups attached and connected to a carboxylic acid group.	kimi-k1.5
High yield (+1): Modifiers containing more than one carboxylic acid group.	kimi-k1.5
Low yield (-1): Modifiers containing electron-donating groups connected to aromatic rings along with a carboxylic acid group.	kimi-k1.5
Low yield (-1): Modifiers that are bulky or highly branched structures (e.g., having more than one ring).	kimi-k1.5
Low yield (-1): Modifiers containing an aldehyde group along with the carboxylic acid group.	kimi-k1.5
High Yield: Aromatic ring present.	qwen-max
High Yield: Hydroxyl group present.	qwen-max
High Yield: Alkoxy group present.	qwen-max
High Yield: Amino group present.	qwen-max
Low Yield: Nitro group present.	qwen-max
Low Yield: Halogen group present.	qwen-max

7. Classification scores of different methods

Table S4. Classification scores of different features.

Features	Accuracy	Recall	Precision	F1 score	ROC-AUC score
Traditional Descriptor	0.618± 0.016	0.44	0.62	0.52	0.52
Fe loading	0.736± 0.014	0.67	0.75	0.71	0.78
Exp. Descriptor	0.713± 0.018	0.67	0.75	0.71	0.77
qwen-max (Non-iterative Coded Matrix) + Exp. Descriptor	0.713± 0.018	0.67	0.75	0.71	0.77
kimi-k1.5 (Non-iterative Coded Matrix) + Exp. Descriptor	0.713± 0.018	0.67	0.75	0.71	0.77
o3-mini (Non-iterative Coded Matrix) + Exp. Descriptor	0.727± 0.015	0.67	0.75	0.71	0.76
o1 (Non-iterative Coded Matrix) + Exp. Descriptor	0.777± 0.013	0.72	0.76	0.74	0.80
o1 (Non-iterative Coded Matrix) + Exp. Descriptor	0.826± 0.013	0.72	0.93	0.81	0.82
Exp. + Traditional Descriptor	0.867± 0.011	0.78	0.93	0.85	0.90

The standard deviation of accuracies is calculated via 50 different random states of the RandomForestClassifier. For other classification scores, the only random state is 42.

8. Hyperparameters for machine learning modules

Table S5. Hyperparameters and models for different methods.

Method	Model (Classifier)	Hyperparameters
4o (Coded Matrix)	Extra Trees	n_estimators=500, random_state=42
4o (Linguistic Matrix)	Extra Trees	n_estimators=500, random_state=42
o1 (Coded Matrix)	Extra Trees	n_estimators=500, random_state=42
o1 (Linguistic Matrix)	Extra Trees	n_estimators=500, random_state=42
Traditional Descriptor	Random Forest	n_estimators=500,max_depth=4, max_features=None
Fe Loading	Random Forest	n_estimators=500,max_depth=1, max_features=None
Exp. Descriptor	Random Forest	n_estimators=500,max_depth=4, max_features=None
qwen-max (Non-iterative Coded Matrix) + Exp. Descriptor	Random Forest	n_estimators=500,max_depth=3, max_features=None
kimi-k1.5 (Non-iterative Coded Matrix) + Exp. Descriptor	Random Forest	n_estimators=500,max_depth=3, max_features=None
o3-mini (Non-iterative Coded Matrix) + Exp. Descriptor	Random Forest	n_estimators=500,max_depth=4, max_features=None
o1 (Non-iterative Coded Matrix) + Exp. Descriptor	Random Forest	n_estimators=500,max_depth=4, max_features=None
o1 (Non-iterative Coded Matrix) + Exp. Descriptor	Random Forest	n_estimators=500,max_depth=3, max_features=None
Exp. + Traditional Descriptor	Random Forest	n_estimators=500,max_depth=4, max_features=None

9. Prompts of the iterative LLM pipeline

```
Prompt for Rule Generator

** System Prompt **
You are collaborating with other agents on a research program focused on the aforementioned problem. Utilizing
your chemical insights, please analyze the dataset provided below and generate rules that describe the
relationship between molecular modifiers (depicted as SMILES strings) and the relative high or low value of
{target_name}.

    If any advice is given, take the advice into consideration.
------------------------------------------------------------------------------------------------------------

**Reaction Background**
    {reaction_background}
------------------------------------------------------------------------------------------------------------

**DataSet**
    {train_set}
------------------------------------------------------------------------------------------------------------

**Requirements for Generated Rules**
    1. Rules should illustrate direct combinations of sub-structures (function groups) features in the modifiers. Combining
multiple sub-structures (function groups) is recommended.
    2. When generating rules, consider the underlying physical-chemical properties in your mind.
    3. Each rule should clearly predict whether the target value is high or low for any SMILES structure that fits its description.
    4. Prioritize rules that cover a broader range of the dataset.
    5. Generate between 5 and 15 rules.
    6. Maintain a suitable balance between simple rules with higher coverage and complex rules with lower coverage.
    7. If you think some of the current rules are terrible, abandon them and write new ones.
---------------------------------------------------------------------------------------------

**Format of Rules**
    **Start of Rules
    - **Rule 1**:...
    - **Rule 2**:...
    - ...
    **End of Rules
------------------------------------------------------------------------------------------------------------

    {current_rules}
    {rule_advice}
# if  not the first generation
------------------------------------------------------------------------------------------------------------

Please generate rules! You're suggested to polish the current rule set
```

Figure S5. Prompt of Rule Generator.

LLMs first generate linguistic rules from SMILES and experimental datasets through the "Rule Generator". This agent receives background information of the catalytic reaction, key chemistry, and its task of generating rules, tapping into the integrated knowledge of the LLM. The "Rule Generator" is also notified that it is working collaboratively with other agents who will evaluate and provide feedback on these rules. Seven guidelines are set for the "Rule Generator":

(1) Rules should illustrate direct combinations of sub-structures (function groups) in the modifiers. Combining multiple sub-structures is encouraged.

(2) Consider underlying physical-chemical properties while generating rules.

(3) Each rule should clearly predict whether the target value is high or low for any SMILES structure that fits its description.

(4) Prioritize rules that cover a broader range of the dataset.

(5) Generate 5 to 15 rules.

(6) Maintain a suitable balance between simple rules with higher coverage and complex rules with lower coverage.

(7) Discard ineffective rules and create new ones as needed.

Prompt for Rule Commenter

```
**System Prompt**
You are collaborating with other agents on a research program focused on the catalytical problem.
Your target is give comment to rules for advance rules and judge if the rules is enough for latter tasks.
------------------------------------------------------------------------------------------

**Reaction Background**
   {reaction_background}
------------------------------------------------------------------------------------------------------------

**DataSet**
   {train_set}
------------------------------------------------------------------------------------------------------------

**Scoring Criteria for Rules**
 # Importance in descending order:
   1. **Clarity**: Determine if you can clearly tell whether the target value is high or low when a modifier matches the
structural description of the rule.
   2. **Property Insight**: Assess whether there is adequate physical-chemical insight corresponding to the properties of the
modifier and the reaction.
   3. **Complexity**: Ensure some rules consider combinations of sub-structures (functional groups) rather than a single
functional group.
   4. **Coverage**: Verify that at least 2 data points support a rule. More support corresponds to a higher score.
   5. **Balance**: Consider the balance between complexity and coverage. Although complex rules with detailed sub-
structures are valuable, sometimes simpler rules with higher coverage are also effective. Try to achieve a balanced approach.
--------------------------------------------------------------------------------------------

**Comment Format**
 **Start of Comments
   - **Comment 1**:...
   - **Comment 2**:...
   - ...
   **End of Comments
--------------------------------------------------------------------------------------------------------------

 **Your Target**
   Scan the rules one by one and score them based on the four criteria above. Sum the scores for each one rule and provide
your comments on the rules.
   If you believe the rules are good enough, conclude your answer with "**TRUE**", if not, DO NOT add anything in the end.
```

Figure S6. Prompt of Rule Commenter.

The generated rules are then reviewed by a “Rule Commenter”, who assesses them based on:

(1) Clarity: whether the rule unambiguously predicts high or low target values.

(2) Property Insight: the extent of physical-chemical reasoning in generating the rule.

(3) Complexity: whether the rule incorporates multiple sub-structures (functional groups).

(4) Coverage: ensuring each rule is supported by at least two data points.

(5) Balance: evaluating trade-offs between rule complexity and dataset coverage.

Prompt for Rule Advisor

```
**System Prompt**
 You are tasked with reading the discussion on the current rules.
 Based on the reaction data, provide practical advice for the rule generator to create improved rules.
--------------------------------------------------------------------------------------------------

**Reaction Background**
   {reaction_background}
   --------------------------------------------------------------------------------------------------

   **Current Rules**
   {current_rules}
   --------------------------------------------------------------------------------------------------

   **DataSet**
   {train_set}
   --------------------------------------------------------------------------------------------------

   **Discussions**
   {input_discussion}     # take needed information to give advice
   --------------------------------------------------------------------------------------------------

  ** Advice Format **
   ** Start of Advice **
   - ** Advice 1 **: ...
   - ** Advice 2 **: ...
   - ...
   ** End of Advice **


   --------------------------------------------------------------------------------------------------

   **Your Target**
   Your advice must be directly practical on how to improve the SMILES based rules set or directly give new rules.
   If you believe the rules are good enough in this stage, conclude your answer with "**TRUE**". If not, DO NOT add
anything at the end.
   Note that advice from Project Manager is important, take them on priority. That means current rule need to be optimized.
```

Figure S7. Prompt of Rule Advisor.

A "Rule Advisor" takes in the outputs from the "Rule Commenter" and provides revision guidance to the "Rule Generator". The iterative process continues until the "Rule Advisor" deems a rule sufficiently refined or determines it should be discarded. While both the "Rule Generator" and "Rule Commenter" focus only on the current round of the rule revision, the "Rule Advisor" can oversee the history of the whole discussion to make a more literal judgment. Once finalized, the rules proceed to the matrix generation stage.

Prompt for Matrix Generator

```
**System Prompt**
You are a coding assistant with expertise in RDkit. Your task is to generate Python code that takes a list of SMILES strings as input. The code should follow the provided natural language rules to convert these SMILES strings into a feature matrix using RDkit. The output matrix should be a DataFrame where each column corresponds to one rule, and each row corresponds to one SMILES string from the list. There should be number_of_SMILES rows and number_of_rules columns.
   Generate a feature matrix with the following criteria:
   - A value of 0 if the structural description of the rule does not match the SMILES.
   - A value of 1 if the structural description of the rule matches the SMILES and predicts a high target value.
   - A value of -1 if the structural description of the rule matches the SMILES and predicts a low target value.
--------------------------------------------------------------------------------------------

**Examples for SMARTS**
   {smarts_intro}  # Collected SMARTS examples for various function groups
   {group_egs}    # Human-designed examples for groups with physical-chemical properties, like electron-donating groups
--------------------------------------------------------------------------------------------

**Current Rules**
   {current_rules}
--------------------------------------------------------------------------------------------

**Suggestions from Matrix Checker**
   {suggestions}
--------------------------------------------------------------------------------------------

   Please generate Python code that follows these rules.
   Your code should be structured in the following format:

   {{
      "prefix": "<Description of the problem and approach>",
      "imports": "<Code block containing import statements>",
      "code": "<Code block not including import statements>"
   }}

   Example for "code":
   {{
   "prefix": "This code converts a list of SMILES strings into a feature matrix using RDkit.",
   "imports": "import pandas as pd\\nfrom rdkit import Chem\\nfrom rdkit.Chem import AllChem",
   "code": {code_example}
   \\n
   }}


   Note:
   Name the function as rule2matrix, Define the function without any example to run that function.
   Using SMARTS for better substructure search.
   Consider appropriate logic (and, or, not/exclude) of SMARTS patterns to describe a rule.
   Handle possible error: when there is any error for one rule apply to one SMILES, return 0 instead.
```

Figure S8. Prompt of Matrix Generator.

Rules are converted into numerical feature matrices for machine learning evaluation. A "Matrix Generator" assigns matrix elements as follows:

(1) **1**: The rule applies and predicts a positive effect on the target (e.g., yield).

(2) **-1**: The rule applies and predicts a negative effect.

(3) **0**: The rule is irrelevant to the molecule.

Two approaches are used for this assignment:

(1) Linguistic mode: direct evaluation based on linguistic rules using LLM knowledge

(2) Code mode: conversion of rules into a Python function for implementation

In the “code” mode, the “Matrix Generator” writes a tailored “rule2matrix(rule, smiles)” function for applying a rule to a sample. The written function contains proper annotations, package imports, and error handling. The way of representing a rule by code is quite flexible. Packages like the chemical informatics module “rdkit” are implemented to examine complex chemical structure relationships or calculate some chemical properties of a molecule for judgment. In addition, SMARTS (SMiles ARbitrary Target Specification) representations are adopted, which use placeholders and logical operators to allow dynamic adaptation to complex and variable structural motifs. Empirical testing showed that the “code” mode achieved higher accuracy in matrix generation compared to the “linguistic” mode.

Prompt for Matrix Checker

```
**System Prompt**
Your task is to check whether the transformation from language rules to a numeric feature matrix is effective. The matrix is generated by a code  written by Matrix Generator. If the matrix transformation is not effective, please give suggestions to improve current code.
The feature matrix is generated with the following criteria:
- A value of 0 if the structural description of the rule does not match the modifier.
- A value of 1 if the structural description of the rule matches the modifier and predicts a high target value.
- A value of -1 if the structural description of the rule matches the modifier and predicts a low target value.
-------------------------------------------------------------------------------------------

**Current Rules**
{current_rules}
-------------------------------------------------------------------------------------------

**SMILES Set**
{whole_SMILES}
-------------------------------------------------------------------------------------------

**Feature Matrix**
{data_matrix}
-------------------------------------------------------------------------------------------

**Examples for SMARTS**
{smarts_intro}
-------------------------------------------------------------------------------------------

**Current code**
{rule_code}
-------------------------------------------------------------------------------------------

**Your Target**
Check if the feature matrix fits the rules. Please check this carefully for each rule and each modifier.
Criteria:
- Check the matrix rule by rule, SMILES by SMILES.
- If there are many 0s in the feature matrix, verify if the rule truly does not match, especially for those 0s at the end of each rule feature list.
- Ensure that the 1s/-1s accurately correspond to high/low target values according to each rule.
- Take your time for thorough evaluation, but avoid making unfounded assumptions.

Give suggestions for how to improve rule code write by Matrix Generator:
- Carefully check the SMARTS write in the code based on if it describes the natural language rules correctly.
- Give suggestions to improve the generated code
If you find that the transformation is not successful, provide practical suggestions for your collaborator to regenerate the code generating rule matrix as SPECIFIC as possible.
-------------------------------------------------------------------------------------------------------------------------------

**Format of suggestions**
** Start of Suggestions **
- ** Suggestion 1 **: ...
- ** Suggestion 2 **: ...
- ...
** End of Suggestions **
-------------------------------------------------------------------------------------------------------------------------------

If the transformation from language rules to numeric feature matrix is effective, add '**TRUE**' at the end of your answer. Otherwise, do not add anything at the end.
```

Figure S9. Prompt of Matrix Checker.

A “Matrix Checker” validates matrix assignment and ensures the overall matrix structure is reasonable. Feedback is provided to refine rule implementation.

Prompt for Metric Commenter

```
**System Prompt**
You are collaborating with other agents on a research program focused on a catalytic problem.
Please provide comments on the performance of the current rules based on the current rule metrics.
You should focus more on confidence and lift.
--------------------------------------------------------------------

**Explanation on rule metrics**
- **Support**: displays the proportion of records for which the entire rule, rule condition(s), and rule prediction(s), are true. For example, if 20% of the training data contains both bread and cheese, then rule support for the rule bread -> cheese is 20%.
- **Support**: displays the ratio of rule support to rule condition support. This indicates the proportion of records with the specified rule condistion(s) for which the rule prediction(s) is/are also true. For example, if 50% of the training data contains bread (indicating rule condition support) but only 20% contains both bread and cheese (indicating rule support), then the prediction for the rule bread -> cheese would be Rule Support / Rule Condition Support or, in this case, 40%.
- **Lift**: displays the ratio of confidence for the rule to the prior probability of having the rule prediction. For example, if 10% of the entire population buys bread, then a rule that predicts whether people will buy bread with 20% confidence will have a lift of 20/10 = 2. If another rule tells you that people will buy bread with 11% confidence, then the rule has a lift of close to 1, meaning that having the rule condition(s) does not make a lot of difference in the probability of having the rule prediction. In general, rules with lift different from 1 will be more interesting than rules with lift close to 1.
- **Leverage**: displays the difference between the observed support for a rule and the expected support if the items were independent. Leverage measures the additional support the rule receives over what would be expected by chance. For example, if 5% of the training data contains both bread and cheese (indicating rule support), and if bread and cheese were independent with bread appearing in 20% and cheese in 10% of the data respectively, the expected support would be 20% * 10% = 2%. The leverage for the rule bread -> cheese would then be Rule Support - Expected Support or, in this case, 5% - 2% = 3%. Positive leverage values indicate a stronger association between the items than expected by chance, while negative leverage values indicate a weaker association. Leverage tends to highlight rules involving more frequent items in the dataset.
--------------------------------------------------------------------

**Reference rule metrics**
{reference_metrics}
--------------------------------------------------------------

**Current Rules**
{current_rules}
--------------------------------------------------------------

**Current Metrics**
{current_metric}
--------------------------------------------------------------------

Please provide comments on the performance of the current rules based on the current metrics. You are encouraged to use reference metric data as a reference.
The previous metric should serve as a baseline reference. The metrics during the current iteration should be used to determine if a local minimum has been reached and if there have been enough iterations.
Comment on how to improve the current rules after your  detailed analysis.
```

Figure S10. Prompt of Metric Commenter.

Prompt for ML Commenter

System Prompt
You are collaborating with other agents on a research program focused on a catalytic problem.
Please provide comments on the accuracies of the validation set and train set.
Give analysis based on the SHAP value calcualted on the validation.
Thinking about how to improve current rules which are used to generate feature matrix.

Reference Accuracis and SHAP values
{reference_trad_metrics}

Current Accuracies and SHAP values
{current_trad_metric}

Current Rules
{current_rules}

Guidance for Analysis
-1. Evaluate Effectiveness:
-Are the current rules effective enough?
-2. Assess Overfitting/Underfitting:
-Are the iterative rules overfitting or underfitting to the current training and validation sets?
-3. Analyze Discrepancies:
-If the predicted loading is high while the experimental value is low for a molecule, the rule with the most positive SHAP value for high loading is likely problematic.
-Conversely, if the predicted loading is low while the experimental value is high for a molecule, the rule with the most negative SHAP value for high loading is likely problematic.
-4. Interpret Accuracy Improvements:
-How do the accuracy metrics suggest the probability of improvement for the current rules?
-5. Derive Insights from SHAP Analysis:
-How does the SHAP analysis indicate opportunities for improving the current rules?
-Note: You cannot directly use SHAP analysis results to modify the rules, but you should consider ways to improve the current rules based on these results.

Performance Evaluation of Current Rules
Please provide comments on the performance of current rules based on the latest training results. Use reference metric data as a baseline for your analysis.
The previous metrics should serve as a baseline reference, while the current iteration metrics should be used to determine if a local minimum has been reached and if there have been enough iterations to refine the rules.

Recommendations for Improvement
After your detailed analysis, please provide comments on how to improve the current rules. Consider the following points in your suggestions:
Refine rules associated with high positive SHAP values for overestimated loadings.
Adjust rules with high negative SHAP values for underestimated loadings.
Improve generalization to address overfitting or underfitting issues.
Use insights from SHAP analysis to iteratively refine and evaluate rule effectiveness.

Figure S11. Prompt of ML Commenter.

Prompt for Program Manager

System Prompt
You are the manager of a target aimed at extracting rules from SMILES to describe the catalytic action of a modified catalyst. Please give your suggestions to improve current rules.

** Reaction Background **
{reaction_background}

** Rule Metric Log **
{rule_metric_log}

** Accuracy and SHAP Log **
{trad_metric_log}

** Training Set **
{train_set}

** Current Rules **
{current_rules}

** Current Rule Metric **
{current_rule_metric}

** Current Accuracy and SHAP **
{current_trad_metric}

** Rule Discussions **
{rule_discussions}

** Your Target **
1. Summarize the discussions above and provide directions to optimize the current rules. Please give your suggestions to improve current rules.
2. Judge if there is any possibility for further optimization of the current rules without overfitting.
3. If you think the rules should be further optimized, write '**Please Optimize Rules**' at the end of your answer. If you think there is enough optimization, do not add anything and let the program shutdown.

Figure S12. Prompt of Project Manager.

10. Outputs of the iteration of the selected rule

Output Message

Rule Generator Message:

Start of Rules

- **Rule 1**: **Aliphatic Amino Acids without Aromatic Rings Yield High**
  Modifiers that are aliphatic amino acids containing amino and carboxylic acid groups without any aromatic rings result in high yield.
  Examples:
  - (2S)-2-aminopentanedioic acid (glutamic acid) – High Yield
  - (2S)-2-aminobutanedioic acid (aspartic acid) – High Yield
  - 5-aminopentanoic acid – High Yield

- **Rule 2**: **Para-Substituted Benzoic Acids with Electron-Withdrawing Groups Yield High**
  Modifiers that are benzoic acid derivatives with electron-withdrawing substituents at the para position (e.g., nitro, bromo) result in high yield. Conversely, those with electron-donating groups or aldehyde groups at the para position yield low.
  Examples:
  - High Yield:
    - 4-(bromomethyl)benzoic acid – High Yield
    - 4-nitrobenzoic acid – High Yield
  - Low Yield:
    - 4-methylbenzoic acid – Low Yield
    - 4-formylbenzoic acid – Low Yield

- **Rule 3**: **Heterocyclic Carboxylic Acids Yield High**
  Modifiers that are heterocyclic carboxylic acids, such as pyridine carboxylic acids and pyrazole carboxylic acids, result in high yield.
  Examples:
  - pyridine-3-carboxylic acid – High Yield
  - 1H-pyrazole-4-carboxylic acid – High Yield
  - 2-oxo-1H-pyridine-4-carboxylic acid – High Yield

- **Rule 4**: **Long-Chain Aliphatic Carboxylic Acids without Polar Groups Yield High**
  Modifiers that are long-chain (C8 and above) aliphatic carboxylic acids without additional polar functional groups result in high yield. The presence of amino groups in the chain decreases the yield.
  Examples:
  - High Yield:
    - octanoic acid – High Yield
  - Low Yield:
    - 8-aminooctanoic acid – Low Yield
    - 7-aminoheptanoic acid – Low Yield

- **Rule 5**: **Modifiers with Multiple Hydroxyl Groups on Aliphatic Chains Yield High**
  Modifiers containing multiple hydroxyl groups attached to aliphatic chains result in high yield due to increased solubility and hydrogen-bonding interactions.
  Examples:
  - (2R,3S,4R,5R)-2,3,4,5,6-pentahydroxyhexanoic acid (gluconic acid) – High Yield
  - 2-[[1,3-dihydroxy-2-(hydroxymethyl)propan-2-yl]oxy]acetic acid – High Yield

- **Rule 6**: **Modifiers with Aldehyde (Formyl) Groups Yield Low**
  Modifiers containing aldehyde groups tend to result in low yield, possibly due to side reactions like Schiff base formation.
  Examples:
  - 4-formylbenzoic acid – Low Yield
  - 5-formyl-2-hydroxybenzoic acid – Low Yield

- **Rule 7**: **Highly Fluorinated Aliphatic Chains Yield Low**
  Modifiers with highly fluorinated aliphatic chains result in low yield, likely due to their hydrophobic nature and poor interaction with catalytic sites.
  Example:
  - 2,2,3,3,4,4,5,5,5-nonafluoropentanoic acid – Low Yield

- **Rule 8**: **Thiol Groups on Aliphatic Chains Yield High; on Aromatic Rings Yield Low**
  Modifiers containing sulfanyl (thiol) groups attached to aliphatic chains result in high yield, whereas thiol groups on aromatic rings result in low yield due to potential catalyst poisoning.
  Examples:
  - High Yield:
    - 3-sulfanylpropanoic acid – High Yield
  - Low Yield:
    - 4-sulfanylbenzoic acid – Low Yield

- **Rule 9**: **Modifiers with Bulky Aromatic Amino Acids Yield Variable Results**
  Modifiers that are aromatic amino acids without additional functional groups tend to yield low, but those with extended aromatic systems can yield high due to π-π interactions.
  Examples:
  - Low Yield:
    - (2S)-2-amino-3-phenylpropanoic acid (phenylalanine) – Low Yield
  - High Yield:
    - (2R)-2-amino-3-(1H-indol-3-yl)propanoic acid (tryptophan) – High Yield

- **Rule 10**: **Modifiers with Polyether Chains Yield Low**
  Modifiers containing methoxyethoxy (polyether) chains result in low yield, possibly due to steric hindrance or unfavorable solvation effects.
  Example:
  - 2-[2-(2-methoxyethoxy)ethoxy]acetic acid – Low Yield

- **Rule 11**: **Hydroxyphenyl Groups with Longer Aliphatic Chains Yield High**
  Modifiers with hydroxyphenyl groups attached via longer aliphatic chains (five or more carbons) result in high yield, while shorter chains result in low yield. Longer chains may facilitate better interaction with the catalyst.
  Examples:
  - High Yield:
    - 5-(4-hydroxyphenyl)pentanoic acid – High Yield
  - Low Yield:
    - 3-(4-hydroxyphenyl)propanoic acid – Low Yield

- **Rule 12**: **Modifiers with Ketone Groups Adjacent to Carboxylic Acids Yield Low**
  Modifiers containing ketone groups adjacent to carboxylic acids tend to result in low yield due to potential enolization or chelation with metal centers.
  Example:
  - 4-oxoheptanedioic acid – Low Yield

- **Rule 13**: **Modifiers with Acetamido Groups on Long Aliphatic Chains Yield Low**
  Modifiers that have acetamido groups attached to long aliphatic chains result in low yield, possibly due to intramolecular interactions that hinder reactivity.
  Example:
  - (2S)-6-acetamido-2-aminohexanoic acid – Low Yield

- **Rule 14**: **Extended Aromatic Systems with Carboxylic Acids Yield High**
  Modifiers containing carboxylic acids directly attached to extended aromatic systems facilitate π-π interactions and result in high yield.
  Example:
  - 1,3-dioxo-2-benzofuran-5-carboxylic acid – High Yield

- **Rule 15**: **Modifiers with Bulky Aromatic Substituents Yield Low**
  Modifiers that have bulky aromatic groups, particularly when these groups are not directly conjugated with the carboxylic acid, result in low yield due to steric hindrance.
  Example:
  - 4-pyridin-4-ylbenzoic acid – Low Yield

End of Rules

Figure S13. The first generation of rules.

Output Message

Rule Commenter Message:
Start of Comments

- **Comment 1**: Rule 1 is clear and supported by multiple data points (modifiers 0, 2, 3). It provides good property insight by linking aliphatic amino acids without aromatic rings to high yield. The rule balances complexity and coverage well.

- **Comment 2**: Rule 2 is clear and well-supported by data points (modifiers 1, 20, 21, 23). It offers good property insight by associating electron-withdrawing para substituents with high yield and electron-donating or aldehyde groups with low yield. The rule effectively balances complexity and coverage.

- **Comment 3**: Rule 3 is clear and supported by data points (modifiers 8, 16, 22). The rule provides property insight by linking heterocyclic carboxylic acids to high yield, possibly due to their coordination abilities. The complexity is appropriate, and the coverage is adequate.

- **Comment 4**: Rule 4 is clear and supported by data points (modifiers 5, 31, 34). It provides property insight by highlighting that long-chain aliphatic acids yield high unless polar groups (like amino groups) are present. The rule balances complexity and coverage effectively.

- **Comment 5**: Rule 5 is clear and supported by data points (modifiers 6, 9). It provides property insight by associating multiple hydroxyl groups with high yield, possibly due to enhanced solubility and hydrogen bonding. The rule is moderately complex with adequate coverage.

- **Comment 6**: Rule 6 is clear and supported by data points (modifiers 19, 20). It links the presence of aldehyde groups to low yield, providing property insight into possible side reactions. The rule has appropriate complexity and sufficient coverage.

- **Comment 7**: Rule 7 is clear and provides property insight by associating highly fluorinated chains with low yield due to hydrophobicity. However, the rule is only supported by one data point (modifier 14), so coverage is insufficient according to the criteria.

- **Comment 8**: Rule 8 is clear and supported by data points (modifiers 10, 18). It offers property insight by distinguishing between aliphatic and aromatic thiol groups' effects on yield. The complexity is appropriate, and coverage meets the minimum requirement.

- **Comment 9**: Rule 9 is somewhat less clear due to the term "variable results" but effectively distinguishes between different aromatic amino acids. It is supported by data points (modifiers 13, 15). The property insight is moderate, explaining the role of extended aromatic systems. Complexity is higher, and coverage is minimal.

- **Comment 10**: Rule 10 is clear and provides property insight about polyether chains leading to low yield. However, it is only supported by one data point (modifier 11), making the coverage insufficient.

- **Comment 11**: Rule 11 is clear and supported by data points (modifiers 7, 30). It provides property insight into the effect of chain length on yield with hydroxyphenyl groups. The rule balances complexity and coverage effectively.

- **Comment 12**: Rule 12 is clear and provides property insight linking adjacent ketone and carboxylic acid groups to low yield. However, it is only supported by one data point (modifier 28), making coverage insufficient.

- **Comment 13**: Rule 13 is clear and offers property insight regarding acetamido groups on long aliphatic chains leading to low yield. However, only one data point (modifier 33) supports this rule, so coverage is insufficient.

- **Comment 14**: Rule 14 is clear and provides property insight by associating extended aromatic systems with high yield. However, with only one data point (modifier 17), coverage is insufficient.

- **Comment 15**: Rule 15 is somewhat unclear due to conflicting data—modifier 12 yields low while modifier 25 yields high. The rule may lack clarity and is supported by only one data point, leading to insufficient coverage.

End of Comments

Figure S14. Comments on the initial rule set.

Output Message

Rule Advisor Message:

Start of Advice

- **Advice 1**: **Combine or Remove Rules with Insufficient Coverage**

Rules that are only supported by one data point (Rules 7, 10, 12, 13, 14) have insufficient coverage. Consider combining them with similar rules to increase their coverage or remove them if they cannot be supported with additional data. For example, merge Rule 7 (highly fluorinated aliphatic chains yield low) with other rules that relate to hydrophobic modifiers yielding low yield.

- **Advice 2**: **Clarify and Refine Rules with Ambiguities**

Rules that lack clarity or have conflicting data (Rules 9 and 15) need to be refined. For Rule 9, avoid ambiguous terms like "variable results" and clearly specify the structural features that lead to high or low yield. For Rule 15, resolve any conflicting data points and ensure the rule accurately reflects the trends observed.

- **Advice 3**: **Increase Coverage by Generalizing or Merging Similar Rules**

To improve coverage, consider generalizing rules or merging similar ones. For instance, combine Rules 6 and 12, both of which involve carbonyl groups (aldehyde and ketone) leading to low yield, into a single rule about modifiers with carbonyl groups adjacent to carboxylic acids resulting in low yield.

- **Advice 4**: **Expand Existing Rules to Include Additional Data Points**

Review the dataset to identify additional modifiers that fit within existing rules but are not currently included. This can help strengthen the rules by increasing their coverage. For example, see if other modifiers with hydroxyl groups (Rule 5) or heterocyclic carboxylic acids (Rule 3) can be incorporated.

- **Advice 5**: **Introduce New Rules Based on Unaddressed Data Patterns**

Analyze the dataset for patterns not covered by existing rules and consider formulating new rules. For instance, modifiers with amino groups on aromatic rings may influence yield; if this pattern exists, create a rule to capture it.

- **Advice 6**: **Ensure Each Rule Provides Clear Property Insight**

Revisit each rule to ensure it offers a clear and specific property insight that explains the observed effect on yield. Avoid overly broad or vague statements. This will enhance the usefulness and applicability of the rules.

- **Advice 7**: **Validate Rules Against the Full Dataset**

Cross-check each rule against the entire dataset to confirm that it consistently predicts high or low yield for relevant modifiers. This helps to identify exceptions or necessary refinements to the rules.

End of Advice

Figure S15. Advice on the initial rule set.

Output Message

Rule Generator Message:

Start of Rules

- **Rule 1**: **Short-Chain Aliphatic Amino Acids Yield High; Long-Chain Amino Acids Yield Low**
  Modifiers that are aliphatic amino acids with carbon chain lengths of 3 to 5 carbons and no aromatic rings result in high yield. Those with longer aliphatic chains (6 carbons or more) yield low.
  Examples:
  - High Yield:
    - (2S)-2-aminobutanedioic acid (aspartic acid) – High Yield
    - (2S)-2-aminopentanedioic acid (glutamic acid) – High Yield
    - 5-aminopentanoic acid – High Yield
  - Low Yield:
    - 7-aminoheptanoic acid – Low Yield
    - 8-aminooctanoic acid – Low Yield
    - (2S)-6-acetamido-2-aminohexanoic acid – Low Yield

- **Rule 2**: **Para-Substituted Benzoic Acids with Electron-Withdrawing Groups Yield High; Electron-Donating or Bulky Groups Yield Low**
  Modifiers that are benzoic acid derivatives with electron-withdrawing substituents at the para position (e.g., nitro, bromo, amino) result in high yield. Those with electron-donating substituents (e.g., methyl, methoxy) or bulky aromatic substituents yield low.
  Examples:
  - High Yield:
    - 4-nitrobenzoic acid – High Yield
    - 4-(bromomethyl)benzoic acid – High Yield
    - 4-aminobenzoic acid – High Yield
  - Low Yield:
    - 4-methylbenzoic acid – Low Yield
    - 4-formylbenzoic acid – Low Yield
    - 4-pyridin-4-ylbenzoic acid – Low Yield

- **Rule 3**: **Heterocyclic Carboxylic Acids Yield High**
  Modifiers that are heterocyclic carboxylic acids, such as pyridine carboxylic acids and pyrazole carboxylic acids, result in high yield due to their ability to coordinate with metal centers and facilitate electron transfer.
  Examples:
  - pyridine-3-carboxylic acid – High Yield
  - 1H-pyrazole-4-carboxylic acid – High Yield
  - 2-oxo-1H-pyridine-4-carboxylic acid – High Yield

- **Rule 4**: **Long-Chain Aliphatic Carboxylic Acids Yield High; Amino-Substituted Analogs Yield Low**
  Modifiers that are long-chain (6 to 8 carbons) aliphatic carboxylic acids without additional polar functional groups result in high yield. Introducing amino groups into the chain decreases the yield, possibly due to increased polarity or coordination that interferes with the catalyst.
  Examples:
  - High Yield:
    - octanoic acid – High Yield
  - Low Yield:
    - 7-aminoheptanoic acid – Low Yield
    - 8-aminooctanoic acid – Low Yield

- **Rule 5**: **Modifiers with Multiple Hydroxyl Groups Yield High**
  Modifiers containing multiple hydroxyl groups, especially on aliphatic chains, result in high yield due to enhanced solubility and increased hydrogen-bonding interactions with the catalyst.
  Examples:
  - (2R,3S,4R,5R)-2,3,4,5,6-pentahydroxyhexanoic acid (gluconic acid) – High Yield
  - 2-[[1,3-dihydroxy-2-(hydroxymethyl)propan-2-yl]oxy]acetic acid – High Yield

- **Rule 6**: **Modifiers with Aldehyde Groups Yield Low**
  Modifiers containing aldehyde (formyl) groups tend to result in low yield, possibly due to side reactions like Schiff base formation or catalyst poisoning.
  Examples:
  - 4-formylbenzoic acid – Low Yield
  - 5-formyl-2-hydroxybenzoic acid – Low Yield

- **Rule 7**: **Thiol Groups on Aliphatic Chains Yield High; On Aromatic Rings Yield Low**
  Modifiers with sulfanyl (thiol) groups attached to aliphatic chains result in high yield, possibly enhancing interaction with the metal center. Thiol groups on aromatic rings result in low yield due to potential catalyst deactivation.
  Examples:
  - High Yield:
    - 3-sulfanylpropanoic acid – High Yield
  - Low Yield:
    - 4-sulfanylbenzoic acid – Low Yield

- **Rule 8**: **Modifiers with Highly Fluorinated Aliphatic Chains Yield Low**
  Modifiers that are highly fluorinated aliphatic carboxylic acids result in low yield, likely due to their hydrophobic nature and poor interaction with the catalytic sites.
  Example:
  - 2,2,3,3,4,4,5,5,5-nonafluoropentanoic acid – Low Yield

- **Rule 9**: **Modifiers with Extended Conjugated Aromatic Systems Yield High**
  Modifiers containing extended conjugated aromatic systems directly attached to carboxylic acid groups facilitate π-π stacking interactions with the catalyst, resulting in high yield.
  Examples:
  - 1,3-dioxo-2-benzofuran-5-carboxylic acid – High Yield
  - (2R)-2-amino-3-(1H-indol-3-yl)propanoic acid (tryptophan) – High Yield

- **Rule 10**: **Modifiers with Polyether Chains Yield Low**
  Modifiers containing polyether chains, such as methoxyethoxy groups, result in low yield, possibly due to steric hindrance or unfavorable solvation effects that interfere with catalyst interaction.
  Example:
  - 2-[2-(2-methoxyethoxy)ethoxy]acetic acid – Low Yield

- **Rule 11**: **Hydroxyphenyl Groups with Longer Aliphatic Linkers Yield High; Shorter Linkers Yield Low**
  Modifiers with hydroxyphenyl groups connected via longer aliphatic chains (five carbons or more) result in high yield, while those with shorter chains yield low. Longer linkers may enhance flexibility and interaction with the catalyst.
  Examples:
  - High Yield:
    - 5-(4-hydroxyphenyl)pentanoic acid – High Yield
  - Low Yield:
    - 3-(4-hydroxyphenyl)propanoic acid – Low Yield

- **Rule 12**: **Modifiers with Ketone Groups Near Carboxylic Acids Yield Low**
  Modifiers containing ketone groups adjacent to carboxylic acids tend to result in low yield, possibly due to chelation with metal centers or interference with catalytic activity.
  Example:
  - 4-oxoheptanedioic acid – Low Yield

- **Rule 13**: **Bulky Aromatic Substituents Not Conjugated with Carboxylic Acid Yield Low**
  Modifiers with bulky aromatic substituents that are not directly conjugated with the carboxylic acid group result in low yield, potentially due to steric hindrance or disruption of favorable interactions with the catalyst.
  Examples:
  - 4-pyridin-4-ylbenzoic acid – Low Yield
  - 2-[(4-hydroxybenzoyl)amino]acetic acid – Low Yield

End of Rules

Figure S16. The generation of the second rule set.

Output Message

Rule Commenter Message:

Start of Comments

- **Comment 1**: Rule 1 is clear in specifying that short-chain aliphatic amino acids (3-5 carbons, no aromatic rings) yield high, while longer chains (6+ carbons) yield low. The structural criteria are well-defined, and the examples in the dataset support the rule, with multiple data points on each side. The rule provides property insight by implicating chain length as a factor influencing yield, possibly due to steric or electronic effects. The complexity is appropriate; it considers both chain length and the absence of aromatic rings. Overall, the rule meets the criteria of clarity, property insight, complexity, and coverage.

- **Comment 2**: Rule 2 attempts to correlate para-substituent electronic effects with yield, stating that electron-withdrawing groups result in high yield, while electron-donating or bulky groups result in low yield. However, there are inconsistencies with this rule. For example, 4-aminobenzoic acid is listed as high yield, but the amino group is generally electron-donating. Similarly, 4-formylbenzoic acid, which contains an electron-withdrawing formyl group, is listed as low yield. This suggests a lack of clarity and property insight in the rule. The rule may need to be refined to accurately reflect the observed data. The complexity of the rule is acceptable, but the coverage is undermined by these inconsistencies.

- **Comment 3**: Rule 3 is clear in stating that heterocyclic carboxylic acids yield high, supported by examples in the dataset. The property insight is sound, as heterocyclic compounds can coordinate with metal centers, potentially facilitating electron transfer in the catalytic process. The complexity is appropriate, focusing on heterocyclic structures. The rule is well-supported by data points, meeting the coverage criterion.

- **Comment 4**: Rule 4 states that long-chain (6-8 carbons) aliphatic carboxylic acids yield high, while amino-substituted analogs yield low. The rule is clear and offers property insight by suggesting that the addition of amino groups decreases yield due to increased polarity or interference with the catalyst. However, the coverage is limited, with only one data point (octanoic acid) supporting high yield for the unmodified long-chain acids. For the amino-substituted analogs, there are two data points showing low yield, which partially supports the rule. The rule's complexity is acceptable, but the limited coverage reduces its robustness.

- **Comment 5**: Rule 5 states that modifiers with multiple hydroxyl groups yield high, attributing this to enhanced solubility and hydrogen-bonding interactions with the catalyst. The rule is clear and provides reasonable property insight. However, the coverage is limited, with only two data points supporting the rule and no contrasting low-yield examples. The complexity is acceptable. To strengthen the rule, additional data points would be beneficial.

- **Comment 6**: Rule 6 states that modifiers with aldehyde groups yield low due to potential side reactions like Schiff base formation or catalyst poisoning. The rule is clear and offers property insight. However, the coverage is minimal, with only two data points supporting the rule. The rule's complexity is low. To enhance the rule's robustness, more data points and perhaps contrasting high-yield examples without aldehyde groups would be helpful.

- **Comment 7**: Rule 7 differentiates between thiol groups on aliphatic chains (high yield) and aromatic rings (low yield). The rule is clear and offers plausible property insight. However, the coverage is very limited, with only one data point for each case. The rule's complexity is acceptable. To strengthen the rule, additional data points are needed to confirm the trend.

- **Comment 8**: Rule 8 states that modifiers with highly fluorinated aliphatic chains yield low due to hydrophobicity affecting interactions with catalytic sites. The rule is clear and offers reasonable property insight. However, the coverage is minimal, with only one data point supporting the rule. The complexity is low. Additional data points are needed to confirm this pattern.

- **Comment 9**: Rule 9 suggests that modifiers with extended conjugated aromatic systems yield high due to π-π stacking interactions. While the rule provides property insight, the term "extended conjugated aromatic systems" is somewhat vague and may lack clarity. The coverage is limited, with only two data points. The complexity is acceptable. To improve the rule, clearer definitions and more supporting data would be beneficial.

- **Comment 10**: Rule 10 states that modifiers with polyether chains yield low due to steric hindrance or unfavorable solvation effects. The rule is clear and offers property insight. However, the coverage is minimal, with only one data point. The complexity is acceptable. Additional examples are needed to support the rule.

- **Comment 11**: Rule 11 states that hydroxyphenyl groups with longer aliphatic linkers yield high, while those with shorter linkers yield low. The rule is clear and provides property insight. The complexity is appropriate, considering both chain length and functional groups. The coverage is minimal, with only one example for each case. More data points are needed to confirm this trend.

- **Comment 12**: Rule 12 suggests that modifiers with ketone groups near carboxylic acids yield low due to chelation with metal centers or interference with catalytic activity. The rule is clear and offers property insight. However, the coverage is minimal, with only one data point. The complexity is acceptable. More supporting data is needed.

- **Comment 13**: Rule 13 states that modifiers with bulky aromatic substituents not conjugated with the carboxylic acid yield low. While the rule provides property insight, it may lack clarity due to the complexity of defining "not conjugated" in this context. The coverage is minimal, with only two data points. The complexity of the rule is high. Clarification and additional data points would strengthen the rule.

End of Comments

Figure S17. Comments on the second rule set.

Output Message

Rule Advisor Message:

Start of Advice

- **Advice 1**: **Refine Rule 2 to Align with Observed Data**
The current Rule 2 suggests that electron-withdrawing groups at the para position lead to high yield, while electron-donating groups result in low yield. However, the data shows inconsistencies—for example, 4-aminobenzoic acid (an electron-donating amino group) yields high, and 4-formylbenzoic acid (an electron-withdrawing formyl group) yields low. Refine the rule to focus on the ability of the substituents to coordinate with the metal center or participate in specific interactions rather than solely on their electronic nature. Consider grouping substituents based on their coordination potential or steric effects.

- **Advice 2**: **Increase Coverage by Combining Similar Rules**
Many rules (e.g., Rules 4, 5, 6, 7, 8, 10, 11, 12, 13) have limited coverage with only one or two supporting data points. To enhance robustness, consider combining rules that share common structural features or mechanisms. For instance, merge Rules 4 and 5 to create a broader rule about aliphatic carboxylic acids with functional groups that enhance solubility or interact favorably with the catalyst.

- **Advice 3**: **Clarify Vague Terminology in Rules**
Terms like "extended conjugated aromatic systems" in Rule 9 are vague and may lead to misinterpretation. Provide specific structural criteria, such as the minimum number of fused rings or specific aromatic systems (e.g., indole, benzofuran). This will improve clarity and applicability.

- **Advice 4**: **Re-express Rules to Reflect Functional Group Context**
Recognize that the same functional group can have different effects depending on its context in the molecule. For example, amino groups in aliphatic chains might decrease yield (Rule 4), while the same groups in aromatic systems may coordinate with the catalyst and increase yield (e.g., 4-aminobenzoic acid). Adjust the rules to account for these contextual differences.

- **Advice 5**: **Incorporate Functional Group Combinations and Positions**
Develop rules that consider combinations of functional groups and their positions within the molecule. For example, the presence of both hydroxyl and carboxylic acid groups on an aliphatic chain may enhance yield (as in gluconic acid), while the same groups in other positions might not have the same effect.

- **Advice 6**: **Gather Additional Data to Support Low-Coverage Rules**
For rules with minimal data support, such as Rules 7 (thiol groups), 8 (fluorinated chains), and 10 (polyether chains), collect more experimental data. Additional examples will help confirm whether these patterns hold true and justify retaining or modifying the rules.

- **Advice 7**: **Clarify and Refine Rule 13 Regarding Bulky Aromatics**
The definition of "bulky aromatic substituents not conjugated with the carboxylic acid" is unclear. Provide explicit structural examples or criteria, such as specifying the size of the aromatic group or the nature of its linkage to the carboxylic acid. Re-evaluate the rule to focus on steric hindrance effects and gather more data to support it.

- **Advice 8**: **Emphasize Specific Interactions Over General Electronic Effects**
Instead of broadly categorizing substituents as electron-donating or -withdrawing, focus on their ability to participate in specific interactions (e.g., metal coordination, hydrogen bonding). This approach may provide better property insight and explain exceptions observed in the data.

- **Advice 9**: **Prioritize Rules with Strong Support and Clear Insights**
Focus on refining and retaining rules that have robust data support and offer clear property insights, such as Rules 1 and 3. Consider omitting or significantly revising rules that cannot be adequately supported, to improve the overall quality and reliability of the rule set.

- **Advice 10**: **Consider Steric and Electronic Effects Together**
In rules where electronic effects don't fully explain the observed yields (e.g., Rule 2), incorporate steric considerations. For example, bulky substituents may hinder catalyst interaction regardless of their electronic nature. Adjust the rules to reflect the combined influence of steric and electronic factors.

End of Advice

Figure S18. Advice on the second rule set.

Output Message

Rule Generator Message:

Start of Rules

- **Rule 1**: **Short-Chain Aliphatic Amino Acids Yield High; Long-Chain Amino Acids Yield Low**

Modifiers that are aliphatic amino acids with carbon chain lengths of 4 to 5 carbons (e.g., aspartic acid, glutamic acid) result in high yield. Those with longer aliphatic chains (6 carbons or more) yield low. This may be due to the optimal size and coordination ability of shorter chains with the catalyst.

Examples:

- **High Yield:**
  - (2S)-2-aminobutanedioic acid (Aspartic acid) – High Yield
  - (2S)-2-aminopentanedioic acid (Glutamic acid) – High Yield
  - 5-aminopentanoic acid – High Yield
- **Low Yield:**
  - 7-aminoheptanoic acid – Low Yield
  - 8-aminooctanoic acid – Low Yield
  - (2S)-6-acetamido-2-aminohexanoic acid – Low Yield

- **Rule 2**: **Para-Substituted Benzoic Acids with Metal-Coordinating Groups Yield High; Non-Coordinating Groups Yield Low**

Modifiers that are benzoic acid derivatives with substituents at the para position capable of coordinating with metal centers (e.g., amino, nitro, bromomethyl) result in high yield. Substituents that are non-coordinating or solely electron-donating (e.g., methyl) yield low. Coordination enhances interaction with the catalyst.

Examples:

- **High Yield:**
  - 4-aminobenzoic acid – High Yield
  - 4-nitrobenzoic acid – High Yield
  - 4-(bromomethyl)benzoic acid – High Yield
- **Low Yield:**
  - 4-methylbenzoic acid – Low Yield
  - 4-formylbenzoic acid – Low Yield

- **Rule 3**: **Heterocyclic Carboxylic Acids Yield High**

Modifiers that are heterocyclic carboxylic acids, such as pyridine carboxylic acids and pyrazole carboxylic acids, result in high yield. Their heteroaromatic rings can coordinate with metal centers and facilitate electron transfer, enhancing catalytic activity.

Examples:

- **High Yield:**
  - Pyridine-3-carboxylic acid – High Yield
  - 1H-pyrazole-4-carboxylic acid – High Yield
  - 2-oxo-1H-pyridine-4-carboxylic acid – High Yield

- **Rule 4**: **Long-Chain Aliphatic Carboxylic Acids Yield High; Amino-Substituted Analogs Yield Low**

Modifiers that are long-chain (6 carbons or more) aliphatic carboxylic acids without additional polar functional groups result in high yield. Introducing amino groups into the chain decreases the yield, possibly due to increased polarity or interference with catalyst coordination.

Examples:

- **High Yield:**
  - Octanoic acid – High Yield
- **Low Yield:**
  - 7-aminoheptanoic acid – Low Yield
  - 8-aminooctanoic acid – Low Yield

- **Rule 5**: **Modifiers with Multiple Hydroxyl Groups Yield High**

Modifiers containing multiple hydroxyl groups, especially on aliphatic chains, result in high yield due to enhanced solubility and hydrogen-bonding interactions with the catalyst. These interactions may facilitate better dispersion and accessibility of active sites.

Examples:

- **High Yield:**
  - (2R,3S,4R,5R)-2,3,4,5,6-pentahydroxyhexanoic acid (Gluconic acid) – High Yield
  - 2-[[1,3-dihydroxy-2-(hydroxymethyl)propan-2-yl]oxy]acetic acid – High Yield

- **Rule 6**: **Modifiers Containing Carbonyl Groups Adjacent to Carboxylic Acids Yield Low**

Modifiers with aldehyde or ketone groups adjacent to carboxylic acids tend to result in low yield. These carbonyl groups might chelate with metal centers or undergo side reactions, hindering the catalyst's activity.

Examples:

- **Low Yield:**
  - 4-formylbenzoic acid – Low Yield
  - 5-formyl-2-hydroxybenzoic acid – Low Yield
  - 4-oxoheptanedioic acid – Low Yield

- **Rule 7**: **Thiol Groups on Aliphatic Chains Yield High; Thiol Groups on Aromatic Rings Yield Low**

Modifiers with thiol (sulfanyl) groups attached to aliphatic chains result in high yield, likely due to their ability to enhance interactions with the metal center. Thiol groups on aromatic rings may lead to catalyst deactivation, resulting in low yield.

Examples:

- **High Yield:**
  - 3-sulfanylpropanoic acid – High Yield
- **Low Yield:**
  - 4-sulfanylbenzoic acid – Low Yield

- **Rule 8**: **Modifiers with Highly Fluorinated Aliphatic Chains Yield Low**

Highly fluorinated aliphatic carboxylic acids result in low yield. The strong electronegativity and hydrophobicity of fluorinated chains can reduce interactions with the catalyst and impede the reaction.

Example:

- **Low Yield:**
  - 2,2,3,3,4,4,5,5,5-nonafluoropentanoic acid – Low Yield

- **Rule 9**: **Modifiers with Extended Conjugated Aromatic Systems Yield High**

Modifiers containing extended conjugated aromatic systems directly attached to carboxylic acid groups facilitate π-π stacking and electronic interactions with the catalyst, resulting in high yield.

Examples:

- **High Yield:**
  - 1,3-dioxo-2-benzofuran-5-carboxylic acid – High Yield
  - (2R)-2-amino-3-(1H-indol-3-yl)propanoic acid (Tryptophan) – High Yield
- **Low Yield:**
  - (2S)-2-amino-3-phenylpropanoic acid (Phenylalanine) – Low Yield

- **Rule 10**: **Hydroxyphenyl Groups with Longer Aliphatic Linkers Yield High; Shorter Linkers Yield Low**

Modifiers with hydroxyphenyl groups connected via longer aliphatic chains (five carbons or more) result in high yield, while those with shorter chains yield low. Longer linkers may provide flexibility, enhancing interactions with the catalyst.

Examples:

- **High Yield:**
  - 5-(4-hydroxyphenyl)pentanoic acid – High Yield
- **Low Yield:**
  - 3-(4-hydroxyphenyl)propanoic acid – Low Yield

- **Rule 11**: **Modifiers with Polyether Chains Yield Low**

Modifiers containing polyether chains, such as methoxyethoxy groups, result in low yield. These chains may cause steric hindrance or unfavorable solvation effects, interfering with catalyst interaction.

Example:

- **Low Yield:**
  - 2-[2-(2-methoxyethoxy)ethoxy]acetic acid – Low Yield

- **Rule 12**: **Modifiers with Bulky Non-Conjugated Aromatic Substituents Yield Low**

Modifiers with bulky aromatic substituents that are not directly conjugated with the carboxylic acid group result in low yield. Steric hindrance or lack of effective electronic interaction with the catalyst may reduce effectiveness.

Examples:

- **Low Yield:**
  - 4-pyridin-4-ylbenzoic acid – Low Yield
  - 2-[(4-hydroxybenzoyl)amino]acetic acid – Low Yield

End of Rules

Figure S19. The generation of the third rule set.

Output Message

Rule Commenter Message:

Start of Comments

- **Comment 1**:

Rule 1: Short-Chain Aliphatic Amino Acids Yield High; Long-Chain Amino Acids Yield Low

- **Clarity**: The rule clearly distinguishes between short-chain (4-5 carbons) and long-chain (6 or more carbons) aliphatic amino acids, making it easy to predict the yield based on chain length.
- **Property Insight**: Provides adequate insight by linking chain length to optimal size and coordination ability with the catalyst, which is relevant to the reaction mechanism.
- **Complexity**: Considers both the chain length and functional group (amino acid), offering a combination of structural features.
- **Coverage**: Supported by multiple data points for both high yield (e.g., aspartic acid, glutamic acid, 5-aminopentanoic acid) and low yield (e.g., 7-aminoheptanoic acid, 8-aminooctanoic acid).
- **Balance**: Achieves a good balance between complexity and coverage, providing a useful rule for prediction.

- **Comment 2**:

Rule 2: Para-Substituted Benzoic Acids with Metal-Coordinating Groups Yield High; Non-Coordinating Groups Yield Low

- **Clarity**: Clearly specifies the position (para) and the nature of substituents (metal-coordinating vs. non-coordinating), allowing for straightforward application.
- **Property Insight**: Highlights the importance of metal coordination in enhancing catalyst interaction, aligning with chemical principles.
- **Complexity**: Incorporates both positional and functional group considerations, adding necessary complexity.
- **Coverage**: Well-supported by examples of high yield (e.g., 4-aminobenzoic acid, 4-nitrobenzoic acid) and low yield (e.g., 4-methylbenzoic acid, 4-formylbenzoic acid).
- **Balance**: Balanced in complexity and breadth, making it an effective predictive rule.

- **Comment 3**:

Rule 3: Heterocyclic Carboxylic Acids Yield High

- **Clarity**: The rule is straightforward and easy to apply to modifiers with heterocyclic rings.
- **Property Insight**: Links the presence of heteroaromatic rings to their ability to coordinate with metal centers and facilitate electron transfer, which is chemically insightful.
- **Complexity**: Focuses on a specific structural feature, providing simplicity without oversimplification.
- **Coverage**: Supported by several data points (e.g., pyridine-3-carboxylic acid, 1H-pyrazole-4-carboxylic acid) showing high yield.
- **Balance**: Maintains simplicity while being supported by sufficient data.

- **Comment 4**:

Rule 4: Long-Chain Aliphatic Carboxylic Acids Yield High; Amino-Substituted Analogs Yield Low

- **Clarity**: Clearly distinguishes between unmodified long-chain acids and their amino-substituted counterparts.
- **Property Insight**: Suggests that additional amino groups may interfere with catalyst coordination, providing a plausible explanation.
- **Complexity**: Combines chain length and functional group presence, adding necessary depth.
- **Coverage**: Well-supported by examples (e.g., octanoic acid yielding high, while 7-aminoheptanoic acid yields low).
- **Balance**: Offers a balanced rule with practical applicability.

- **Comment 5**:

Rule 5: Modifiers with Multiple Hydroxyl Groups Yield High

- **Clarity**: The presence of multiple hydroxyl groups is a clear and identifiable feature.
- **Property Insight**: Associates enhanced solubility and hydrogen-bonding interactions with improved catalytic activity, which is chemically reasonable.
- **Complexity**: Focuses on a functional group pattern without overcomplicating.
- **Coverage**: Supported by examples like gluconic acid, providing enough data points.
- **Balance**: Simple rule with sufficient coverage, making it useful for predictions.

- **Comment 6**:

  Rule 6: Modifiers Containing Carbonyl Groups Adjacent to Carboxylic Acids Yield Low

  - **Clarity**: Clear in specifying the position of carbonyl groups relative to carboxylic acids.
  - **Property Insight**: Provides a rationale involving possible chelation or side reactions that hinder activity.
  - **Complexity**: Addresses a specific structural scenario, offering a precise guideline.
  - **Coverage**: Supported by multiple low-yield examples (e.g., 4-formylbenzoic acid).
  - **Balance**: Effectively balances specificity with adequate data support.

- **Comment 7**:

  Rule 7: Thiol Groups on Aliphatic Chains Yield High; Thiol Groups on Aromatic Rings Yield Low

  - **Clarity**: Clearly differentiates based on the location of thiol groups.
  - **Property Insight**: Explains how thiol placement affects interaction with the metal center.
  - **Complexity**: Combines functional group identity and its position on the molecule.
  - **Coverage**: Examples support both outcomes (e.g., 3-sulfanylpropanoic acid yields high; 4-sulfanylbenzoic acid yields low).
  - **Balance**: Adequate complexity and coverage justify the rule's inclusion.

- **Comment 8**:

  Rule 8: Modifiers with Highly Fluorinated Aliphatic Chains Yield Low

  - **Clarity**: Clear identification of highly fluorinated chains.
  - **Property Insight**: Relates the effect of electronegativity and hydrophobicity on catalyst interaction.
  - **Complexity**: Simple and focused.
  - **Coverage**: Limited to a single data point (e.g., nonafluoropentanoic acid yielding low).
  - **Balance**: Coverage is minimal; additional data would strengthen the rule.

- **Comment 9**:

  Rule 9: Modifiers with Extended Conjugated Aromatic Systems Yield High

  - **Clarity**: Clearly defines the structural feature of extended conjugation.
  - **Property Insight**: Connects conjugation to enhanced π-π stacking and electronic interactions.
  - **Complexity**: Addresses specific structural complexity.
  - **Coverage**: Supported by examples yielding high (e.g., tryptophan) and contrasting low-yield cases (e.g., phenylalanine).
  - **Balance**: Well-balanced with sufficient complexity and data support.

- **Comment 10**:

  Rule 10: Hydroxyphenyl Groups with Longer Aliphatic Linkers Yield High; Shorter Linkers Yield Low

  - **Clarity**: Clearly specifies linker length as a determining factor.
  - **Property Insight**: Provides insight into how linker length affects flexibility and catalyst interaction.
  - **Complexity**: Combines functional groups and molecular architecture.
  - **Coverage**: Supported by data (e.g., 5-(4-hydroxyphenyl)pentanoic acid yields high; 3-(4-hydroxyphenyl)propanoic acid yields low).
  - **Balance**: Offers a practical rule with good predictive capability.

- **Comment 11**:

  Rule 11: Modifiers with Polyether Chains Yield Low

  - **Clarity**: Clearly identifies modifiers with polyether chains.
  - **Property Insight**: Suggests steric hindrance or solvation effects as reasons for low yield.
  - **Complexity**: Simple and to the point.
  - **Coverage**: Limited to one example (e.g., methoxyethoxyethoxy acetic acid yielding low).
  - **Balance**: Coverage is limited; more data would enhance the rule's reliability.

- **Comment 12**:

  Rule 12: Modifiers with Bulky Non-Conjugated Aromatic Substituents Yield Low

  - **Clarity**: Clearly defines the nature of bulky, non-conjugated substituents.
  - **Property Insight**: Addresses steric hindrance and lack of effective electronic interaction.
  - **Complexity**: Incorporates both size and electronic considerations.
  - **Coverage**: Supported by multiple data points (e.g., 4-pyridin-4-ylbenzoic acid yielding low).
  - **Balance**: Provides a complex, well-supported rule useful for screening.

End of Comments

TRUE

Figure S20. Comments on the third rule set.

Output Message

Matrix Generator Message:

Code for rules:

```
def rule2matrix(smiles_list):
    # Define the rules with their associated patterns and predictions
    rules = [
        {
            'number': 1,
            'description': 'Rule 1: Short-Chain Aliphatic Amino Acids Yield High; Long-Chain Amino Acids Yield Low',
            'high_patterns': [
                {'amine': Chem.MolFromSmarts('[NX3H2]')},  # Primary amine
                {'carboxylic_acid': Chem.MolFromSmarts('[CX3](=O)[OX1H0-,OX2H1]')},  # Carboxylic acid
            ],
            'low_patterns': [
                {'amine': Chem.MolFromSmarts('[NX3H2]')},  # Primary amine
                {'carboxylic_acid': Chem.MolFromSmarts('[CX3](=O)[OX1H0-,OX2H1]')},  # Carboxylic acid
            ],
            'prediction': None  # Determined by chain length
        },
        {
            'number': 2,
            'description': 'Rule 2: Para-Substituted Benzoic Acids with Metal-Coordinating Groups Yield High; Non-Coordinating Groups Yield
Low',
            'high_patterns': [
                {'para_substituted_benzoic_acid': Chem.MolFromSmarts('c1ccc(cc1)C(=O)[OH]')},
                {'coordinating_group': [
                    Chem.MolFromSmarts('c1ccc(cc1[NH2])C(=O)[OH]'),  # Amino group
                    Chem.MolFromSmarts('c1ccc(cc1[N+](=O)[O-])C(=O)[OH]'),  # Nitro group
                    Chem.MolFromSmarts('c1ccc(cc1CBr)C(=O)[OH]'),  # Bromomethyl group
                ]},
            ],
            'low_patterns': [
                {'para_substituted_benzoic_acid': Chem.MolFromSmarts('c1ccc(cc1)C(=O)[OH]')},
                {'non_coordinating_group': [
                    Chem.MolFromSmarts('c1ccc(cc1C)C(=O)[OH]'),  # Methyl group
                ]},
            ],
            'prediction': None  # Determined by substituent type
        },
        {
            'number': 3,
            'description': 'Rule 3: Heterocyclic Carboxylic Acids Yield High',
            'patterns': [
                {'heterocyclic_carboxylic_acid': Chem.MolFromSmarts('[cH,r5,r6][cH,r5,r6]C(=O)[OH]')},
            ],
            'prediction': 1
        },
        {
            'number': 4,
            'description': 'Rule 4: Long-Chain Aliphatic Carboxylic Acids Yield High; Amino-Substituted Analogs Yield Low',
            'high_patterns': [
                {'long_chain_carboxylic_acid': Chem.MolFromSmarts('C[C@H](CCCC)C(=O)[OH]')},  # Chain length >=6
            ],
            'low_patterns': [
                {'amino_substituted': Chem.MolFromSmarts('*N*')},  # Contains amino group
            ],
            'prediction': None  # Determined by amino substitution
        },
        # ... Additional rules here, following the same structure ...
    ]
```

```
# Initialize results list
    results = []
    # Process each SMILES string
    for smi in smiles_list:
        mol = Chem.MolFromSmiles(smi)
        if mol is None:
            # If the molecule cannot be parsed, append a row of zeros
            results.append([0]*len(rules))
            continue
        row = []
        for rule in rules:
            try:
                match = False
                # Rule-specific logic
                if rule['number'] == 1:
                    # Rule 1 implementation
                    amine = rule['high_patterns'][0]['amine']
                    carboxylic_acid = rule['high_patterns'][1]['carboxylic_acid']
                    amine_matches = mol.GetSubstructMatches(amine)
                    carboxylic_matches = mol.GetSubstructMatches(carboxylic_acid)
                    chain_lengths = []
                    for a in amine_matches:
                        for c in carboxylic_matches:
                            path = Chem.GetShortestPath(mol, a[0], c[0])
                            carbons_in_path = [atom_idx for atom_idx in path if mol.GetAtomWithIdx(atom_idx).GetAtomicNum() == 6]
                            chain_length = len(carbons_in_path)
                            chain_lengths.append(chain_length)
                    if chain_lengths:
                        min_chain_length = min(chain_lengths)
                        if 2 <= min_chain_length <= 3:
                            row.append(1)
                        elif min_chain_length >= 4:
                            row.append(-1)
                        else:
                            row.append(0)
                    else:
                        row.append(0)
                elif rule['number'] == 2:
                    # Rule 2 implementation
                    benzoic_acid = rule['high_patterns'][0]['para_substituted_benzoic_acid']
                    benzoic_matches = mol.GetSubstructMatches(benzoic_acid)
                    if benzoic_matches:
                        high = False
                        low = False
                        # Check for coordinating groups
                        for patt in rule['high_patterns'][1]['coordinating_group']:
                            if mol.HasSubstructMatch(patt):
                                high = True
                                break
                        # Check for non-coordinating groups
                        for patt in rule['low_patterns'][1]['non_coordinating_group']:
                            if mol.HasSubstructMatch(patt):
                                low = True
                                break
                        if high:
                            row.append(1)
                        elif low:
                            row.append(-1)
                        else:
                            row.append(0)
                    else:
                        row.append(0)
                elif rule['number'] == 3:
                    # Rule 3 implementation
                    for patt_dict in rule['patterns']:
                        for patt in patt_dict.values():
                            if mol.HasSubstructMatch(patt):
                                match = True
                                break
                    row.append(rule['prediction'] if match else 0)
                elif rule['number'] == 4:
                    # Rule 4 implementation
                    long_chain_acid = rule['high_patterns'][0]['long_chain_carboxylic_acid']
                    amino_substituted = rule['low_patterns'][0]['amino_substituted']
                    if mol.HasSubstructMatch(long_chain_acid):
                        if mol.HasSubstructMatch(amino_substituted):
                            row.append(-1)
                        else:
                            row.append(1)
                    else:
                        row.append(0)
                # ... Implement other rules similarly ...
                else:
                    # Default case
                    row.append(0)
            except Exception as e:
                # In case of any error, append 0
                row.append(0)
        results.append(row)
    # Create DataFrame with results
    df = pd.DataFrame(results, columns=[f'Rule {rule["number"]}' for rule in rules])
    return df
```

Figure S21. Code to translate SMILES into a feature matrix based on the third rule set.

Output Message

Matrix Checker Message:

** Start of Suggestions **

- ** Suggestion 1 **: Modify Rule 1 in the code to exclude aromatic systems. Currently, the code counts the chain length between the amine and carboxylic acid irrespective of whether it's aliphatic or aromatic. Since Rule 1 specifically refers to aliphatic amino acids, the code should ensure that the path between the amine and carboxylic acid consists only of aliphatic (non-aromatic) carbons.
- ** Suggestion 2 **: Adjust the SMARTS patterns in Rule 1 to explicitly match aliphatic chains. You can use the `[C;!R]` notation to specify non-aromatic carbons. For example, redefine the patterns to ensure that both the amine and carboxylic acid are connected via an aliphatic chain without traversing any aromatic atoms.
- ** Suggestion 3 **: For Rule 2, revise the SMARTS patterns to accurately detect para-substituted benzoic acids with coordinating groups. The current patterns may not correctly identify molecules like 4-aminobenzoic acid. Use explicit substitution patterns to ensure that the substituents are at the para position relative to the carboxylic acid group.
- ** Suggestion 4 **: Modify the `benzoic_acid` pattern in Rule 2 to specify the carboxylic acid at the para position relative to the substituent. For example, use a SMARTS like `c1cc(ccc1[X])C(=O)[OH]`, where `[X]` represents the para-substituent. This will help in distinguishing between para, meta, and ortho substitutions.
- ** Suggestion 5 **: In Rule 4, replace the specific SMILES `'C[C@H](CCCC)C(=O)[OH]'` with a more general SMARTS pattern that matches long-chain aliphatic carboxylic acids. For instance, use `'CCCCCCC(=O)[OH]'` to match chains with six or more carbons. Additionally, account for any stereochemistry by removing chirality specifications unless necessary.
- ** Suggestion 6 **: Update the `amino_substituted` pattern in Rule 4. The pattern `'*N*'` is too broad and matches any molecule containing nitrogen. Instead, use a SMARTS pattern that identifies amino groups attached to the aliphatic chain of the carboxylic acid, such as `'CCCNCC(=O)[OH]'` for a three-carbon chain with an amino group.
- ** Suggestion 7 **: Refine the SMARTS pattern in Rule 3 to accurately capture heterocyclic carboxylic acids. The current pattern `'[cH,r5,r6][cH,r5,r6]C(=O)[OH]'` might match unintended structures. Consider using a pattern like `'[n,o,s][#6]C(=O)[OH]'` to match heteroatoms in a ring directly attached to a carboxylic acid.
- ** Suggestion 8 **: Incorporate validation steps in the code to ensure that each rule applies exclusively to its intended molecular features. This may involve adding checks for ring sizes, heteroatoms, or specific functional group arrangements to prevent misclassification.

** End of Suggestions **

Figure S22. Comments on the first code provided by Matrix Checker.

Output Message

Matrix Generator Message:

Code for rules:

```
def rule2matrix(smiles_list):
    # Define the rules with their associated patterns and predictions
    rules = [
        {
            'number': 1,
            'description': 'Rule 1: Short-Chain Aliphatic Amino Acids Yield High; Long-Chain Amino Acids Yield Low',
            'predictions': {'high': 1, 'low': -1},
            'amine_pattern': Chem.MolFromSmarts('[NX3H2;!$(N[a])]'),  # Primary aliphatic amine
            'carboxylic_acid_pattern': Chem.MolFromSmarts('[CX3](=O)[OX1H0-,OX2H1]'),
            'chain_length_bounds': {'high': (4, 5), 'low': (6, None)},  # High: 4-5 carbons, Low: >=6 carbons
        },
        {
            'number': 2,
            'description': 'Rule 2: Para-Substituted Benzoic Acids with Metal-Coordinating Groups Yield High; Non-Coordinating Groups Yield
Low',
            'predictions': {'high': 1, 'low': -1},
            'benzoic_acid_pattern': Chem.MolFromSmarts('c1ccc(cc1)C(=O)[OH]'),
            'para_coordinating_patterns': [
                Chem.MolFromSmarts('c1ccc(cc1[NH2])C(=O)[OH]'),  # Para-amino
                Chem.MolFromSmarts('c1ccc(cc1[N+](=O)[O-])C(=O)[OH]'),  # Para-nitro
                Chem.MolFromSmarts('c1ccc(cc1CBr)C(=O)[OH]'),  # Para-bromomethyl
            ],
            'para_non_coordinating_patterns': [
                Chem.MolFromSmarts('c1ccc(cc1C)C(=O)[OH]'),  # Para-methyl
                Chem.MolFromSmarts('c1ccc(cc1C=O)C(=O)[OH]'),  # Para-formyl
            ],
        },
        {
            'number': 3,
            'description': 'Rule 3: Heterocyclic Carboxylic Acids Yield High',
            'pattern': Chem.MolFromSmarts('[#6][C](=O)[O;H,-]'),  # Carboxylic acid attached to any carbon
            'heterocycle_pattern': Chem.MolFromSmarts('[n,o,s]1cccc1'),  # Five-membered heteroaromatic ring
            'prediction': 1,
        },
        {
            'number': 4,
            'description': 'Rule 4: Long-Chain Aliphatic Carboxylic Acids Yield High; Amino-Substituted Analogs Yield Low',
            'predictions': {'high': 1, 'low': -1},
            'long_chain_acid_pattern': Chem.MolFromSmarts('CCCCCCC(=O)[OH]'),  # Aliphatic chain with 6 or more carbons
            'amino_substituted_pattern': Chem.MolFromSmarts('[#6][NX3H2]'),  # Amino group attached to carbon
        },
        # ... Additional rules can be added here ...
    ]

    # Initialize results list
    results = []

    # Process each SMILES string
    for smi in smiles_list:
        mol = Chem.MolFromSmiles(smi)
        if mol is None:
            # If the molecule cannot be parsed, append a row of zeros
            results.append([0]*len(rules))
            continue
        row = []
```

```
for rule in rules:
        try:
            if rule['number'] == 1:
                # Rule 1 implementation
                amine_matches = mol.GetSubstructMatches(rule['amine_pattern'])
                acid_matches = mol.GetSubstructMatches(rule['carboxylic_acid_pattern'])
                chain_lengths = []
                for amine_match in amine_matches:
                    for acid_match in acid_matches:
                        path = Chem.GetShortestPath(mol, amine_match[0], acid_match[0])
                        # Check if all atoms in path are aliphatic carbons (non-aromatic carbons)
                        aliphatic = True
                        carbon_count = 0
                        for atom_idx in path:
                            atom = mol.GetAtomWithIdx(atom_idx)
                            if atom.GetAtomicNum() == 6:  # Carbon
                                if atom.GetIsAromatic():
                                    aliphatic = False
                                    break
                                else:
                                    carbon_count += 1
                            elif atom.GetAtomicNum() in [7, 8, 1]:  # N, O, or H
                                continue
                            else:
                                aliphatic = False
                                break
                        if aliphatic:
                            # Exclude the N and C atoms from the chain length count
                            chain_length = carbon_count - 1  # Subtract one if we want bonds between carbons
                            chain_lengths.append(chain_length)
                if chain_lengths:
                    min_chain_length = min(chain_lengths)
                    if rule['chain_length_bounds']['high'][0] <= min_chain_length <= rule['chain_length_bounds']['high'][1]:
                        row.append(rule['predictions']['high'])
                    elif rule['chain_length_bounds']['low'][0] <= min_chain_length:
                        row.append(rule['predictions']['low'])
                    else:
                        row.append(0)
                else:
                    row.append(0)
```

```
elif rule['number'] == 2:
                # Rule 2 implementation
                benzoic_matches = mol.GetSubstructMatches(rule['benzoic_acid_pattern'])
                if benzoic_matches:
                    high = False
                    low = False
                    # Check for coordinating groups
                    for patt in rule['para_coordinating_patterns']:
                        if mol.HasSubstructMatch(patt):
                            high = True
                            break
                    # Check for non-coordinating groups
                    if not high:
                        for patt in rule['para_non_coordinating_patterns']:
                            if mol.HasSubstructMatch(patt):
                                low = True
                                break
                    if high:
                        row.append(rule['predictions']['high'])
                    elif low:
                        row.append(rule['predictions']['low'])
                    else:
                        row.append(0)
                else:
                    row.append(0)
            elif rule['number'] == 3:
                # Rule 3 implementation
                if mol.HasSubstructMatch(rule['pattern']):
                    # Check for heterocyclic ring
                    if mol.HasSubstructMatch(rule['heterocycle_pattern']):
                        row.append(rule['prediction'])
                    else:
                        row.append(0)
                else:
                    row.append(0)
            elif rule['number'] == 4:
                # Rule 4 implementation
                if mol.HasSubstructMatch(rule['long_chain_acid_pattern']):
                    if mol.HasSubstructMatch(rule['amino_substituted_pattern']):
                        row.append(rule['predictions']['low'])
                    else:
                        row.append(rule['predictions']['high'])
                else:
                    row.append(0)
            else:
                # Default case for other rules not implemented
                row.append(0)
        except Exception:
            # In case of any error, append 0
            row.append(0)
    results.append(row)

  # Create DataFrame with results
  df = pd.DataFrame(results, columns=[f'Rule {rule['number']}' for rule in rules])
  return df
```

Figure S23. The second generation of the code.

Output Message

Matrix Checker Message:

** Start of Suggestions **

- **Suggestion 1**: **Align the SMILES Set with the Feature Matrix**

There is a mismatch between the number of SMILES strings (24) and the number of rows in the feature matrix (36). Ensure that each SMILES string corresponds to one row in the feature matrix. This alignment is crucial for accurate mapping and evaluation.

- **Suggestion 2**: **Correct Chain Length Calculation in Rule 1**

In Rule 1, instead of counting the number of carbons in the shortest path between the amine and carboxylic acid groups, identify the side chain connected to the α-carbon of the amino acid. The chain length should be the number of carbons in the side chain (excluding the α-carbon). Modify the code to accurately identify and count the side chain carbons to distinguish between short and long-chain amino acids.

- **Suggestion 3**: **Generalize the SMARTS Patterns in Rule 2**

The SMARTS patterns for para-substituted benzoic acids with coordinating and non-coordinating groups are too specific. Generalize the patterns to match any para-substituted benzoic acid with metal-coordinating groups (e.g., `[NX3;H2,H1,H0]`, `[OX1-,OX2]`, `[SX2]`) and non-coordinating groups (e.g., alkyl, aldehyde). Use wildcard patterns or specify properties (like donor atoms) to capture a broader range of substituents.

- **Suggestion 4**: **Expand Heterocycle Patterns in Rule 3**

The heterocycle pattern in Rule 3 only matches five-membered rings, whereas the rule includes six-membered heterocycles like pyridine. Modify the SMARTS pattern to include both five- and six-membered heteroaromatic rings, such as `[n,o,s]1cccc1` and `[n,o,s]1ccccc1`, to accurately identify all relevant heterocyclic carboxylic acids.

- **Suggestion 5**: **Parameterize Chain Length in Rule 4**

In Rule 4, the `long_chain_acid_pattern` is hard-coded for a specific chain length. Replace it with a pattern that matches aliphatic carboxylic acids with chain lengths of six carbons or more, such as `CCCCCCC(=O)[OH]` and use recursive SMARTS or quantifiers to specify chain lengths (e.g., `C[C;R0][C;R0]{4,}`).

- **Suggestion 6**: **Refine Amino Substituent Detection in Rule 4**

The `amino_substituted_pattern` in Rule 4 may not detect all amino-substituted analogs. Update it to match amino groups anywhere on the carbon chain, not just directly attached to a carbon atom, using a pattern like `[$([NX3H2,NX3H1][C;!R])]`.

- **Suggestion 7**: **Implement Missing Rules and Update Code**

The code currently only implements Rules 1 to 4. Extend the code to include the remaining rules (Rules 5 to 12). Carefully translate each natural language rule into the corresponding SMARTS patterns, ensuring they accurately reflect the described chemical features.

- **Suggestion 8**: **Validate SMARTS Patterns Against Examples**

For each rule, test the SMARTS patterns against the provided examples to confirm that they match high-yield and low-yield modifiers appropriately. Adjust the patterns as needed based on the validation results.

- **Suggestion 9**: **Improve Error Handling and Molecule Parsing**

Enhance the code's robustness by adding error handling for cases where the SMILES strings may not parse correctly or where substructure searches might fail due to different tautomeric forms or resonance structures. Use standardized molecule representations (e.g., Kekulization, tautomer normalization) to improve matching accuracy.

- **Suggestion 10**: **Add Comments and Documentation to Code**

Include detailed comments and documentation within the code to explain the purpose of each SMARTS pattern, the logic behind each rule implementation, and any assumptions made. This will aid in future code maintenance and facilitate collaboration.

** End of Suggestions **

- **Advice 5**: **Simplify and Generalize the Rules**

Revise the rules to reduce complexity and overlap. Consolidate similar rules and focus on the most impactful features. For example:

- Combine Rules 1 and 4 by creating a rule that specifies the optimal carbon chain length for aliphatic carboxylic acids and amino acids.
- Merge Rules 2 and 9 by focusing on the presence of metal-coordinating groups and conjugated systems, quantified by specific descriptors.

- **Advice 6**: **Incorporate Machine-Learnable Features**

Modify the rules to include features that machine learning algorithms can easily interpret:

- **Binary Indicators**: Presence or absence of specific functional groups (1 or 0).
- **Counts**: Number of times a particular feature appears (e.g., number of hydroxyl groups).
- **Distances**: Number of bonds between functional groups (e.g., distance from carboxyl group to amino group).

- **Advice 7**: **Use Hierarchical Classification of Functional Groups**

Classify functional groups hierarchically to capture both general and specific features:

- **Level 1**: Broad categories (e.g., aliphatic, aromatic, heterocyclic).
- **Level 2**: Specific functional groups (e.g., carboxylic acid, hydroxyl group).
- **Level 3**: Substituent positions and patterns (e.g., para-substituted aromatic ring).

- **Advice 8**: **Annotate Modifiers with SMARTS Patterns**

Utilize SMILES Arbitrary Target Specification (SMARTS) patterns to precisely define structural features:

- Create SMARTS patterns for high-yielding motifs.
- Use these patterns to identify and annotate modifiers programmatically.

- **Advice 9**: **Perform Statistical Analysis to Identify Key Features**

Analyze the existing dataset statistically to determine which features most strongly correlate with high yield:

- Use methods like correlation coefficients or feature importance from machine learning models.
- Focus the rules on these key features to improve predictive power.

- **Advice 10**: **Validate and Iterate the Rules**

After revising the rules, validate them against the dataset to ensure they accurately predict the yield outcomes:

- Check for consistency between the rules and experimental results.
- Refine the rules based on discrepancies to improve accuracy.

** End of Advice **

Figure S24. Comments on the second version of the code.

Output Message

Rule Generator Message:

Start of Rules

- **Rule 1**: **Short-Chain Aliphatic Amino Acids Yield High; Longer-Chain Analogs Yield Low**

Modifiers that are aliphatic amino acids with a carbon chain length of 4 or 5 carbons and an amino group at the α-position result in high yield. Longer-chain amino acids (6 carbons or more) yield low. This may be due to optimal coordination and minimal steric hindrance with the catalyst for shorter chains.

Examples:

- **High Yield:**
  - (2S)-2-aminobutanedioic acid (Aspartic acid)
  - (2S)-2-aminopentanedioic acid (Glutamic acid)
  - 5-aminopentanoic acid
- **Low Yield:**
  - (2S)-6-acetamido-2-aminohexanoic acid
  - 7-aminoheptanoic acid
  - 8-aminooctanoic acid

- **Rule 2**: **Para-Substituted Benzoic Acids with Electron-Withdrawing, Metal-Coordinating Groups Yield High; Electron-Donating or Non-Coordinating Groups Yield Low**

Modifiers that are benzoic acid derivatives with electron-withdrawing substituents at the para position capable of coordinating with metal centers (e.g., amino, nitro, bromomethyl) result in high yield. Those with electron-donating groups (e.g., methyl, methoxy) or non-coordinating substituents yield low. Coordination enhances interaction with the catalyst.

Examples:

- **High Yield:**
  - 4-aminobenzoic acid
  - 4-nitrobenzoic acid
  - 4-(bromomethyl)benzoic acid
- **Low Yield:**
  - 4-methylbenzoic acid
  - 4-formylbenzoic acid
  - 4-sulfanylbenzoic acid

- **Rule 3**: **Nitrogen-Containing Heterocyclic Carboxylic Acids Yield High**

Modifiers that are heterocyclic carboxylic acids with nitrogen-containing aromatic rings, such as pyridine and pyrazole carboxylic acids, result in high yield. Their ability to coordinate with metal centers enhances catalytic activity.

Examples:

- **High Yield:**
  - Pyridine-3-carboxylic acid (Niacin)
  - 1H-pyrazole-4-carboxylic acid
  - 2-oxo-1H-pyridine-4-carboxylic acid

- **Rule 4**: **Straight-Chain Aliphatic Carboxylic Acids Yield High; Addition of Polar Functional Groups Lowers Yield**

Modifiers that are straight-chain aliphatic carboxylic acids without additional functional groups result in high yield. Introducing polar groups like hydroxyl or amino decreases the yield, potentially due to altered solubility or interference with catalyst coordination.

Examples:

- **High Yield:**
  - Octanoic acid
- **Low Yield:**
  - 7-hydroxyheptanoic acid
  - 7-aminoheptanoic acid
  - 8-aminooctanoic acid

- **Rule 5**: **Modifiers with Multiple Hydroxyl Groups on Short Chains Yield High; Single Hydroxyl on Long Chains Yields Low**

Modifiers containing multiple hydroxyl groups on short aliphatic chains enhance solubility and interaction with the catalyst, resulting in high yield. A single hydroxyl group on a longer chain does not have the same effect and yields low.

Examples:

- **High Yield:**
  - (2R,3S,4R,5R)-2,3,4,5,6-pentahydroxyhexanoic acid (Gluconic acid)
- **Low Yield:**
  - 7-hydroxyheptanoic acid

- **Rule 6**: **Aliphatic Thiol Groups Yield High; Aromatic Thiol Groups Yield Low**

Modifiers with thiol (–SH) groups on aliphatic chains result in high yield, likely due to effective coordination with the metal catalyst. Thiol groups attached to aromatic rings may lead to catalyst poisoning or deactivation, yielding low.

Examples:

- **High Yield:**
  - 3-sulfanylpropanoic acid
- **Low Yield:**
  - 4-sulfanylbenzoic acid

- **Rule 7**: **Highly Fluorinated Aliphatic Chains Yield Low**

Modifiers with multiple fluorine atoms in aliphatic chains result in low yield. The strong electronegativity and hydrophobic nature of fluorinated chains can reduce interactions with the catalyst and hinder the reaction.

Example:

- **Low Yield:**
  - 2,2,3,3,4,4,5,5,5-nonafluoropentanoic acid

- **Rule 8**: **Modifiers with Carbonyl Groups Adjacent to Carboxylic Acids Yield Low**

Modifiers possessing aldehyde or ketone groups adjacent to carboxylic acids tend to yield low. These carbonyl groups might chelate with the metal catalyst or participate in side reactions, reducing catalytic efficiency.

Examples:

- **Low Yield:**
  - 4-formylbenzoic acid
  - 5-formyl-2-hydroxybenzoic acid
  - 4-oxoheptanedioic acid

- **Rule 9**: **Modifiers with Extended Conjugation Involving Heterocycles Yield High**

Modifiers containing extended conjugated systems with oxygen or nitrogen heterocycles enhance electron delocalization and coordination with the catalyst, resulting in high yield.

Example:

- **High Yield:**
  - 1,3-dioxo-2-benzofuran-5-carboxylic acid

- **Rule 10**: **Hydroxyphenyl Groups Connected by Long Aliphatic Chains Yield High; Short Chains Yield Low**

Modifiers with hydroxyphenyl groups linked by aliphatic chains of five or more carbons to a carboxylic acid yield high, possibly due to increased flexibility and optimal interaction with the catalyst. Shorter chains may not provide this advantage.

Examples:

- **High Yield:**
  - 5-(4-hydroxyphenyl)pentanoic acid
- **Low Yield:**
  - 3-(4-hydroxyphenyl)propanoic acid

- **Rule 11**: **Modifiers with Polyether Chains Yield Low**

Modifiers containing polyether chains, such as methoxyethoxy units, result in low yield. These moieties may introduce steric hindrance or unfavorable solvation effects, impeding effective catalyst interaction.

Example:

- **Low Yield:**
  - 2-[2-(2-methoxyethoxy)ethoxy]acetic acid

- **Rule 12**: **Bulky Non-Coordinating Substituents Yield Low**

Modifiers with bulky substituents that cannot effectively coordinate with the metal catalyst lead to low yield. Steric hindrance may prevent proper interaction with the active sites.

Examples:

- **Low Yield:**
  - 4-pyridin-4-ylbenzoic acid
  - 2-[[(2S)-2-amino-4-methylsulfanylbutanoyl]amino]acetic acid

- **Rule 13**: **Aromatic Rings with Amino Groups Adjacent to Carboxylic Acids Yield High**

Modifiers where an amino group is directly attached to an aromatic ring adjacent to a carboxylic acid enhance metal coordination, resulting in high yield.

Example:

- **High Yield:**
  - 4-aminobenzoic acid

- **Rule 14**: **Modifiers with Methoxy Groups Without Coordinating Substituents Yield Low**

Modifiers featuring methoxy groups attached to aromatic rings or aliphatic chains yield low if they lack other coordinating groups. Methoxy groups are electron-donating but do not coordinate effectively with metal catalysts.

Examples:

- **Low Yield:**
  - 2-[(4-hydroxybenzoyl)amino]acetic acid
  - 4-(3,4-dimethoxyphenyl)-4-oxobutanoic acid (Note: Despite containing methoxy groups, this compound yields high, possibly due to other contributing factors.)

- **Rule 15**: **Modifiers with Fused Nitrogen Heterocyclic Aromatic Systems Yield High**

Modifiers containing fused aromatic systems with nitrogen heterocycles (e.g., indole ring in tryptophan) can delocalize electrons and coordinate with metal centers effectively, yielding high.

Example:

- **High Yield:**
  - (2R)-2-amino-3-(1H-indol-3-yl)propanoic acid (Tryptophan)

End of Rules

Figure S25. The generation of the 4th rule set.

Output Message

Rule Commenter Message:

Start of Comments

- **Comment 1**: Rule 1 is clear and allows us to determine the yield based on the carbon chain length and the position of the amino group in aliphatic amino acids. It provides strong physical-chemical insight, attributing high yields to optimal coordination and minimal steric hindrance in shorter chains. The rule considers both chain length and functional groups, offering a good balance between complexity and coverage with multiple supporting data points.

- **Comment 2**: Rule 2 is well-defined, focusing on para-substituted benzoic acids with electron-withdrawing, metal-coordinating groups yielding high, and those with electron-donating or non-coordinating groups yielding low. It offers solid physical-chemical insight into how electron-withdrawing substituents enhance metal coordination. The rule effectively balances complexity and coverage, supported by multiple data points that validate its applicability.

- **Comment 3**: Rule 3 provides clear guidance by identifying nitrogen-containing heterocyclic carboxylic acids as yielding high. It offers adequate physical-chemical insight into the role of nitrogen heterocycles in metal coordination. While the rule is clear and considers specific substructures, its coverage could be strengthened with additional data points, although it currently meets the minimum requirement.

- **Comment 4**: Rule 4 is straightforward and clear, stating that straight-chain aliphatic carboxylic acids yield high, while the addition of polar functional groups lowers the yield. The rule provides reasonable physical-chemical insight into how polar groups may alter solubility or interfere with catalyst coordination. It effectively balances complexity by considering both the absence and presence of functional groups and is well-supported by multiple data points.

- **Comment 5**: Rule 5 lacks sufficient coverage, with limited data points supporting the high yield for modifiers with multiple hydroxyl groups on short chains. While it offers some physical-chemical insight into enhanced solubility and catalyst interaction, the rule's complexity may not justify its limited applicability. Additional data is needed to strengthen the rule's reliability.

- **Comment 6**: Rule 6 is clear in distinguishing between aliphatic and aromatic thiol groups' effects on yield. However, the rule's coverage is minimal, relying on a single high-yield and one low-yield example. Despite providing physical-chemical insight into metal coordination and potential catalyst poisoning, the limited data reduces confidence in the rule's general applicability.

- **Comment 7**: Rule 7 identifies that highly fluorinated aliphatic chains yield low, attributing this to reduced catalyst interaction due to electronegativity and hydrophobicity. The rule is clear but has very limited coverage with only one supporting data point. This minimal coverage hampers its effectiveness and reliability for broader application.

- **Comment 8**: Rule 8 is clear and supported by several data points showing that modifiers with carbonyl groups adjacent to carboxylic acids yield low. It offers adequate physical-chemical insight, suggesting possible chelation or side reactions with the catalyst. The rule balances complexity by focusing on specific functional group arrangements and has sufficient coverage to support its validity.

- **Comment 9**: Rule 9 has low coverage, supported by only one data point. While it introduces the concept that extended conjugation involving heterocycles yields high, the rule lacks clarity due to its vague description. The physical-chemical insight is plausible but not sufficiently detailed, and more data points are needed to validate this rule.

- **Comment 10**: Rule 10 discusses the impact of chain length in hydroxyphenyl-substituted aliphatic acids on yield. Despite offering some physical-chemical insight, the rule is based on minimal data, with only one high-yield and one low-yield example. The limited coverage and specificity reduce its reliability for predicting outcomes with new modifiers.

- **Comment 11**: Rule 11 addresses modifiers with polyether chains yielding low, suggesting steric hindrance or unfavorable solvation effects. The rule is clear but has insufficient coverage, supported by a single data point. This minimal support limits its applicability and confidence in the rule's generalizability.

- **Comment 12**: Rule 12 is clear in stating that bulky non-coordinating substituents yield low. It provides reasonable physical-chemical insight into how steric hindrance may prevent proper catalyst interaction. The rule considers combinations of substructures and is supported by at least two data points, offering a balance between complexity and coverage.

- **Comment 13**: Rule 13 overlaps with Rule 2, focusing on aromatic rings with amino groups adjacent to carboxylic acids yielding high. While it provides adequate physical-chemical insight into enhanced metal coordination, the rule's coverage is limited to a single data point. The redundancy and minimal support reduce its added value beyond what is already covered in Rule 2.

- **Comment 14**: Rule 14 is unclear and contains contradictory information. The rule states that modifiers with methoxy groups without coordinating substituents yield low, yet provides an example where such a compound yields high. This inconsistency undermines the rule's clarity and reliability, and the lack of sufficient supporting data further weakens its validity.

- **Comment 15**: Rule 15 introduces the idea that modifiers with fused nitrogen heterocyclic aromatic systems yield high, exemplified by tryptophan. While the rule offers physical-chemical insight into electron delocalization and metal coordination, it is supported by only one data point. The limited coverage necessitates more data to confirm the rule's applicability and strengthen its predictive power.

End of Comments

Figure S26. Comments on the 4th rule set.

Output Message

Rule Advisor Message:

** Start of Advice **

- **Advice 1**: **Clarify and Correct Rule 2 Regarding Metal Coordination**

Issue: Rule 2 currently states that para-substituted benzoic acids with electron-withdrawing, metal-coordinating groups yield high, while electron-donating or non-coordinating groups yield low. However, there is inconsistency because 4-aminobenzoic acid (an electron-donating group) yields high.

Suggestion:

- **Focus on Metal-Coordinating Ability**: Redefine the rule to emphasize that substituents capable of coordinating with metal centers (regardless of their electron-donating or -withdrawing nature) lead to high yield.
- **Remove Conflicting Information**: Eliminate references to electron-donating or -withdrawing properties that do not align with the observed data.
- **Practical Implementation**: Adjust the rule to state:

> **Rule 2 (Revised)**: **Para-Substituted Benzoic Acids with Metal-Coordinating Groups Yield High; Non-Coordinating Groups Yield Low**

This focuses on the presence of functional groups (e.g., amino, nitro, bromomethyl) that can coordinate with the metal catalyst, leading to high yield.

- **Advice 2**: **Combine Overlapping Rules Related to Heterocyclic and Conjugated Systems**

Issue: Rules 3, 9, and 15 all deal with heterocyclic or conjugated aromatic systems yielding high due to enhanced coordination but are fragmented.

Suggestion:

- **Create a Unified Rule**: Merge these rules into a single rule that covers modifiers with heterocyclic aromatic systems containing nitrogen or oxygen.
- **Enhance Clarity and Coverage**: Provide a broader definition that can be applied more generally.

Practical Implementation:

> **Rule (Combined)**: **Modifiers with Nitrogen or Oxygen-Containing Heterocyclic Aromatic Systems Yield High**

This consolidated rule simplifies the rule set and encompasses more compounds, improving usability.

- **Advice 3**: **Strengthen Rules with Limited Data by Including More Examples or Revising**

Issue: Rules 5, 6, 7, 9, 10, 11, and 15 have limited supporting data, reducing their reliability.

Suggestion:

- **Collect Additional Data**: Search for more compounds that fit these rules to validate them.
- **Revise or Eliminate Weak Rules**: If additional data is not available, consider combining these rules with others or removing them to prevent confusion.

Practical Implementation:

- For Rule 5 (Modifiers with Multiple Hydroxyl Groups Yield High), find more examples or integrate it with Rule 1 or Rule 4 if appropriate.
- For Rule 6 (Thiol Groups), assess whether the effect is significant across more data points.
- Focus on rules with strong support and practical significance.

- **Advice 4**: **Address Contradictions in Rules Involving Methoxy Groups**

Issue: Rule 14 states that modifiers with methoxy groups without coordinating substituents yield low, yet an example provided yields high.

Suggestion:

- **Reevaluate the Rule**: Examine the data to understand why exceptions occur.
- **Clarify Exceptions**: If other functional groups contribute to high yield despite the presence of methoxy groups, note this in the rule.

Practical Implementation:

- Modify the rule to:

> **Rule (Revised)**: **Modifiers with Methoxy Groups Yield Low Unless Accompanied by Strong Metal-Coordinating Functionalities**

- Provide explanations for exceptions to guide proper application.

- **Advice 5**: **Enhance Physical-Chemical Insights in Rules**

Issue: Some rules lack detailed explanations, which can limit understanding and application.

Suggestion:

- **Include Mechanistic Rationale**: Add explanations on how specific groups influence the reaction, such as electronic effects, steric hindrance, or solubility factors.
- **Link to Catalyst Interaction**: Describe how modifiers affect the coordination environment of the metal center.

Practical Implementation:

- For each rule, include a brief discussion of the underlying chemistry.
- For example, in the revised Rule 2, explain that metal-coordinating groups can form coordination bonds with the Fe center, enhancing catalytic activity.

- **Advice 6**: **Prioritize Rules Based on Data Strength and Predictive Power**

Issue: Not all rules are equally robust or valuable for predictions.

Suggestion:

- **Focus on High-Confidence Rules**: Emphasize rules with strong data support and clear trends.
- **Simplify the Rule Set**: Remove or consolidate less reliable rules to make the set more user-friendly.

Practical Implementation:

- Create a tiered rule set where primary rules have strong support, and secondary rules are used with caution.
- Ensure that the rules included are those that consistently predict the yield outcome based on the dataset.

- **Advice 7**: **Include Structural Features in SMILES-Based Rules**

Issue: To practically apply the rules, specific structural identifiers need to be translated into SMILES patterns.

Suggestion:

- **Define SMILES Substructures**: For each rule, specify the SMILES patterns corresponding to the functional groups or motifs described.
- **Use SMARTS Notation for Substructures**: Incorporate SMARTS patterns to identify functional groups within SMILES strings.

Practical Implementation:

- For example, in the revised Rule 2:

  - Metal-coordinating groups can be identified by SMARTS patterns for amino (`[NH2]`), nitro (`[N+](=O)[O-]`), or bromomethyl (`[CH2]Br`) substituents at the para position.

- Provide these patterns alongside the rules to facilitate computational screening.

- **Advice 8**: **Ensure Consistent Terminology and Definitions**

Issue: Inconsistent use of terms (e.g., 'long-chain' vs. specific carbon numbers) can cause confusion.

Suggestion:

- **Standardize Definitions**: Use consistent terminology for chain lengths, functional group positions, and substituent types.
- **Specify Carbon Counts**: When referring to chain lengths, provide exact numbers (e.g., C4-C5 for short chains, C6+ for long chains).

Practical Implementation:

- Update rules to include precise definitions.
- For example, revise Rule 1 to specify "carbon chain lengths of exactly 4 or 5 carbons".

- **Advice 9**: **Incorporate Negative Rules Where Appropriate**

Issue: Some compounds consistently yield low, and identifying these can prevent ineffective modifications.

Suggestion:

- **Explicitly State Negative Predictors**: Define structures or functional groups that reliably result in low yields.
- **Balance Positive and Negative Rules**: This will improve the overall predictive power of the rule set.

Practical Implementation:

- For example, create a rule such as:

  > **Rule**: **Modifiers with Polyether Chains Yield Low**

  - Clearly list such structures to avoid in future designs.

- **Advice 10**: **Validate and Update Rules with Ongoing Data**

Issue: As new data becomes available, rules may need adjustments.

Suggestion:

- **Implement Continuous Validation**: Regularly test rules against new compounds.
- **Be Open to Refinement**: Update rules to reflect new findings and improve accuracy.

Practical Implementation:

- Establish a feedback mechanism where experimental results are used to validate and refine the rules over time.
- Encourage collaboration between experimentalists and modelers to maintain an up-to-date rule set.

** End of Advice **

Figure S27. Advice on the 4th rule set.

Output Message

Matrx Generator Message:

Code for rules:

```
def rule2matrix(smiles_list):
    # Number of rules
    num_rules = 15  # There are 15 rules as provided
    # Initialize results list
    results = []
    # Define SMARTS patterns and logic for each rule
    # Rule 2 patterns for high yield substituents
    amino_group = Chem.MolFromSmarts('[NX3;H2,H1]')  # Primary or secondary amine
    nitro_group = Chem.MolFromSmarts('[NX3](=O)=O')  # Nitro group
    bromomethyl_group = Chem.MolFromSmarts('CBr')     # Bromomethyl group
    # Rule 4 patterns
    straight_chain_carboxylic_acid = Chem.MolFromSmarts('C(=O)[O;H1,-]')
    polar_groups = [
        Chem.MolFromSmarts('[OX2H]'),  # Hydroxyl group
        Chem.MolFromSmarts('[NX3;H2,H1]'),  # Amine group
    ]
    # Rule 6 patterns
    aliphatic_thiol = Chem.MolFromSmarts('[#6][SX2H]')
    aromatic_thiol = Chem.MolFromSmarts('[c][SX2H]')
    # Rule 7 pattern
    fluorinated_chain = Chem.MolFromSmarts('[CX4](F)(F)(F)')  # Trifluoromethyl as an example
    # Rule 13 pattern
    amino_adjacent_to_carboxy_aromatic = Chem.MolFromSmarts('[c][NX3;H2,H1][c][C](=O)[O;H1,-]')
# Process each SMILES string
    for smi in smiles_list:
        mol = Chem.MolFromSmiles(smi)
        if mol is None:
            # If the molecule cannot be parsed, append a row of zeros
            results.append([0]*num_rules)
            continue
        row = []
        # Rule 1
        try:
            value = check_rule1(mol)
            row.append(value)
        except Exception:
            row.append(0)
        # Rule 2
        try:
            value = check_rule2(mol, amino_group, nitro_group, bromomethyl_group)
            row.append(value)
        except Exception:
            row.append(0)
        # Rule 3
        try:
            value = check_rule3(mol)
            row.append(value)
        except Exception:
            row.append(0)
      # Rule 4
        try:
            value = check_rule4(mol, straight_chain_carboxylic_acid, polar_groups)
            row.append(value)
        except Exception:
            row.append(0)
        # Rule 5
        try:
            value = check_rule5(mol)
            row.append(value)
        except Exception:
            row.append(0)
        # ... Continue for Rules 6 to 15
```

```
# Rule 6
        try:
            value = check_rule6(mol, aliphatic_thiol, aromatic_thiol)
            row.append(value)
        except Exception:
            row.append(0)
        # Rule 7
        try:
            value = check_rule7(mol, fluorinated_chain)
            row.append(value)
        except Exception:
            row.append(0)
        # Rule 13
        try:
            value = check_rule13(mol, amino_adjacent_to_carboxy_aromatic)
            row.append(value)
        except Exception:
            row.append(0)
        # For remaining rules, append 0 or implement similar logic
        # Fill remaining rules with zeros for now
        while len(row) < num_rules:
            row.append(0)
        results.append(row)
    # Create DataFrame with results
    columns = [f'Rule {i+1}' for i in range(num_rules)]
    df = pd.DataFrame(results, columns=columns)
    return df

# Define the functions for each rule

def check_rule1(mol):
    # Rule 1: Short-Chain Aliphatic Amino Acids Yield High; Longer-Chain Analogs Yield Low
    # Aliphatic amino acids with chain length of 4 or 5 carbons and an amino group at the α-position result in high yield.
    # Longer-chain amino acids (6 carbons or more) yield low.
    # SMARTS pattern for alpha-amino acid
    amino_acid_smarts = '[NX3H2][CX4H]([*])[CX3](=O)[OX2H1]'
    amino_acid_mol = Chem.MolFromSmarts(amino_acid_smarts)
    matches = mol.GetSubstructMatches(amino_acid_mol)
    if not matches:
        return 0  # Not an alpha-amino acid
    for match in matches:
        alpha_c_idx = match[1]
        chain_length = get_chain_length_from_alpha(mol, alpha_c_idx)
        if chain_length in [4, 5]:
            return 1  # High yield
        elif chain_length >= 6:
            return -1  # Low yield
    return 0

def get_chain_length_from_alpha(mol, alpha_c_idx):
    # Calculate the number of carbons from the alpha carbon to the terminal carbon
    visited = set()
    max_chain_length = 0
    def dfs(atom_idx, length):
        nonlocal max_chain_length
        visited.add(atom_idx)
        atom = mol.GetAtomWithIdx(atom_idx)
        if atom.GetSymbol() != 'C':
            return
        max_chain_length = max(max_chain_length, length)
        for neighbor in atom.GetNeighbors():
            nbr_idx = neighbor.GetIdx()
            if nbr_idx not in visited and neighbor.GetSymbol() == 'C':
                dfs(nbr_idx, length + 1)
    dfs(alpha_c_idx, 1)
    return max_chain_length
```

```
def check_rule2(mol, amino_group, nitro_group, bromomethyl_group):
    # Rule 2: Para-Substituted Benzoic Acids with EW and Coordinating Groups Yield High
    # Electron-Withdrawing, Metal-Coordinating Groups at para position result in high yield
    # Electron-Donating or Non-Coordinating Groups yield low
    # Identify benzoic acid core
    benzoic_acid = Chem.MolFromSmarts('c1ccc(cc1)C(=O)[O;H1,-]')
    if not mol.HasSubstructMatch(benzoic_acid):
        return 0
    # Identify para-substituted benzoic acids
    para_substituted = Chem.MolFromSmarts('c1cc([#6,#7,#8,#9,#16,#17,#35,#53])ccc1C(=O)[O;H1,-]')
    if not mol.HasSubstructMatch(para_substituted):
        return 0
    # Now check if substituent is EWG capable of coordination (amino, nitro, bromomethyl)
    if mol.HasSubstructMatch(amino_group) or mol.HasSubstructMatch(nitro_group) or mol.HasSubstructMatch(bromomethyl_group):
        return 1  # High yield
    else:
        return -1  # Low yield

def check_rule3(mol):
    # Rule 3: Nitrogen-Containing Heterocyclic Carboxylic Acids Yield High
    # Identify nitrogen-containing aromatic rings and carboxylic acid groups
    nitrogen_heterocycle = Chem.MolFromSmarts('[n]1cccc1')  # Example: pyridine ring
    carboxylic_acid = Chem.MolFromSmarts('C(=O)[O;H1,-]')
    if mol.HasSubstructMatch(nitrogen_heterocycle) and mol.HasSubstructMatch(carboxylic_acid):
        return 1
    else:
        return 0

def check_rule4(mol, straight_chain_carboxylic_acid, polar_groups):
    # Rule 4: Straight-Chain Aliphatic Carboxylic Acids Yield High; Addition of Polar Groups Lowers Yield
    if mol.HasSubstructMatch(straight_chain_carboxylic_acid):
        # Check for polar groups
        for group in polar_groups:
            if mol.HasSubstructMatch(group):
                return -1  # Low yield due to polar group
        return 1  # High yield
    else:
        return 0

def check_rule5(mol):
    # Rule 5: Modifiers with Multiple Hydroxyl Groups on Short Chains Yield High; Single Hydroxyl on Long Chains Yields Low
    hydroxyl_group = Chem.MolFromSmarts('[OX2H]')
    matches = mol.GetSubstructMatches(hydroxyl_group)
    if len(matches) >= 2:
        # Multiple hydroxyl groups
        aliphatic_chain = Chem.MolFromSmarts('[C][C][C]')  # Simplified pattern for short chain
        if mol.HasSubstructMatch(aliphatic_chain):
            return 1  # High yield
    elif len(matches) == 1:
        # Single hydroxyl on long chain
        long_chain = Chem.MolFromSmarts('[C][C][C][C][C]')
        if mol.HasSubstructMatch(long_chain):
            return -1  # Low yield
    return 0

def check_rule6(mol, aliphatic_thiol, aromatic_thiol):
    # Rule 6: Aliphatic Thiol Groups Yield High; Aromatic Thiol Groups Yield Low
    if mol.HasSubstructMatch(aliphatic_thiol):
        return 1  # High yield
    elif mol.HasSubstructMatch(aromatic_thiol):
        return -1  # Low yield
    else:
        return 0

def check_rule7(mol, fluorinated_chain):
    # Rule 7: Highly Fluorinated Aliphatic Chains Yield Low
    if mol.HasSubstructMatch(fluorinated_chain):
        return -1  # Low yield
    else:
        return 0

def check_rule13(mol, amino_adjacent_to_carboxy_aromatic):
    # Rule 13: Aromatic Rings with Amino Groups Adjacent to Carboxylic Acids Yield High
    if mol.HasSubstructMatch(amino_adjacent_to_carboxy_aromatic):
        return 1  # High yield
    else:
        return 0
```

Figure S28. The generation of the third version of code.

Output Message

Matrix Checker Message:

** Start of Suggestions **

- **Suggestion 1**: **Refine the SMARTS pattern for Rule 7 to accurately represent highly fluorinated aliphatic chains**. The current pattern `'[CX4](F)(F)(F)'` matches any trifluoromethyl group, including those attached to non-aliphatic carbons (e.g., attached directly to heteroatoms or aromatic rings). Rule 7 specifies that modifiers with multiple fluorine atoms in *aliphatic chains* yield low. Update the SMARTS pattern to capture fluorinated aliphatic chains with multiple fluorinated carbons, such as `'[*][C;R0;X4](F)(F)[C;R0;X4](F)(F)[*]'`, which looks for consecutive aliphatic carbons each bearing fluorines.

- **Suggestion 2**: **Adjust the code for Rule 4 to accurately identify straight-chain aliphatic carboxylic acids and the presence of additional polar functional groups**. The current code may incorrectly assign high or low yields without properly checking for chain branching or ring structures. Modify the SMARTS for straight-chain carboxylic acids to exclude rings and branches, e.g., `'C(-C)(-C)-C(=O)[O;H1,-]'`. Additionally, ensure the code checks for the absence of any other functional groups besides the terminal carboxylic acid.

- **Suggestion 3**: **Improve the chain length calculation in `check_rule1` for Rule 1**. The current function `get_chain_length_from_alpha` may not accurately calculate the chain length from the alpha carbon to the terminal carbon, potentially including side chains or overestimating the chain length. Refine the function to trace the longest linear carbon chain starting from the alpha carbon, excluding branches. Utilize breadth-first search (BFS) limiting traversal to acyclic, aliphatic carbon atoms.

- **Suggestion 4**: **Enhance the SMARTS patterns in Rule 2 to ensure correct identification of para-substituted benzoic acids with coordinating groups**. The current code may not specifically check that the substituents are at the para position relative to the carboxylic acid group. Update the SMARTS to `'c1cc([$(N),$(N(=O)=O),$(CBr)])ccc1C(=O)[O;H1,-]'`, where `[$(N),$(N(=O)=O),$(CBr)]` represents the amino, nitro, or bromomethyl groups at the para position.

- **Suggestion 5**: **Refine the handling of Rule 5 to distinguish between multiple hydroxyl groups on short chains and single hydroxyl groups on long chains**. The current code might not accurately differentiate chain lengths or the number of hydroxyl groups. Implement more specific SMARTS patterns to check for short aliphatic chains (e.g., 5 carbons or fewer) with two or more hydroxyl groups, such as `'[C&R0]-[C&R0]-[C&R0]-[C&R0]-C(=O)[O;H1,-]'` with multiple `-OH` groups attached.

- **Suggestion 6**: **Implement the remaining rules (8 to 15) in the code to provide a complete feature matrix**. This will enable a thorough evaluation of the transformation effectiveness. Each rule should have a corresponding function, similar to `check_rule1` to `check_rule7`, with appropriate SMARTS patterns and logic based on the natural language descriptions.

- **Suggestion 7**: **Update the SMARTS pattern for Rule 13 to accurately identify aromatic rings with amino groups adjacent to carboxylic acids**. The current pattern `'[c][NX3;H2,H1][c][C](=O)[O;H1,-]'` may not correctly capture all relevant molecules. Modify it to `'c1ccc([NH2])cc1C(=O)[O;H1,-]'` to ensure the amino group is directly attached to the aromatic ring and adjacent (ortho or para) to the carboxylic acid.

- **Suggestion 8**: **Ensure that the context and connectivity of functional groups are correctly considered in all SMARTS patterns**. For example, in Rule 7, the fluorine atoms should be attached to carbons within an aliphatic chain, not to carbons of functional groups like carbonyls or aromatic systems. Adjust patterns to check for fluorine substitutions along linear aliphatic chains.

- **Suggestion 9**: **Validate and test the SMARTS patterns against known positive and negative examples**. For each rule, create a set of test molecules that should match or not match the SMARTS patterns. This will help verify that the patterns are accurately capturing the intended structures and can be adjusted based on test results.

- **Suggestion 10**: **Consider advanced features like recursive SMARTS or molecular fingerprints for complex rules**. For rules involving extended conjugation (Rule 9) or fused aromatic heterocycles (Rule 15), simple SMARTS patterns may be insufficient. Use recursive SMARTS or substructure searches that can capture these more complex molecular features.

** End of Suggestions **

Figure S29. Suggestions on the third version of code.

Output Message

Metric Calculator Message:

Train support: 0.07047619047619047
Train confidence: 0.5919047619047618
Train lift: 1.184967320261438
Train leverage: 0.009523809523809524

Figure S30. Rule metrics calculated on the feature matrix.

Output Message

Metric Commenter Message:

Analysis of Current Rule Performance Based on Metrics

After reviewing the current rule metrics in comparison with the reference metrics from previous iterations, we can make the following observations and evaluations focusing on **confidence** and **lift** as key indicators of rule performance.

Current Metrics vs. Reference Metrics

Support:

- **Current Train Support:** **0.0705**
- **Previous Train Support:** **0.1667**, **0.1845**

Interpretation:

- The **current train support** is significantly lower than the previous values.
- **Lower support** indicates the current rules apply to a **smaller portion** of the training data.
- This suggests that the rules have become **more specific**, potentially limiting their applicability.

Confidence:

- **Current Train Confidence:** **0.5919**
- **Previous Train Confidence:** **0.6352**, **0.5396**

Interpretation:

- The current confidence is **slightly below** the first previous value and **above** the second.
- This indicates that when the rule conditions are met, there is approximately a **59.19% chance** of correctly predicting the outcome.
- The confidence level remains **moderate** but has not significantly improved.

Lift:

- **Current Train Lift:** **1.1849**
- **Previous Train Lift:** **1.2048**, **1.0559**

Interpretation:

- The current lift is **slightly below** the first previous value and **above** the second.
- A lift value **greater than 1** signifies that the rule is **better than random chance** at predicting the outcome.
- The **current lift** reflects a **moderate association**, similar to previous iterations.

Leverage:

- **Current Train Leverage:** **0.0095**
- **Previous Train Leverage:** **0.0289**, **-0.0041**

Interpretation:

- The current leverage is **lower** than the first previous value but **higher** than the second, which was negative.
- **Positive leverage** indicates the rule has predictive power **above chance**, but the small value suggests the association is **weak**.

Overall Performance Assessment

- **Support Decrease:** The drop in support suggests the rules are covering **fewer data instances**, potentially affecting the rules' **generalizability** and **practical usefulness**.
- **Confidence Stability:** Confidence remains at a **moderate level**, indicating **no significant improvement** in the accuracy of the predictions when the rule conditions are satisfied.
- **Lift Consistency:** The lift values have remained relatively **stable**, showing **no substantial enhancement** in the rules' ability to predict the outcome compared to the prior probability.
- **Leverage Weakness:** The low leverage value points to a **limited increase** in the rule's predictive power over random chance.

Local Minimum Consideration

- The metrics suggest that the current iteration has **not achieved significant improvements** over previous iterations.
- The **stagnation** in confidence and lift, coupled with the **reduced support**, indicates that we may have reached a **local minimum** in rule performance.
- **Further iterations** using the current approach may yield **diminishing returns**, suggesting a need to **re-evaluate** the strategy.

Recommendations for Rule Improvement

To enhance the current rules' performance, the following strategies are recommended:

1. **Expand Rule Coverage (Increase Support):**
   - **Generalize Rule Conditions:**
     - Broaden specific conditions within the rules to **encompass a wider range** of data points.
     - **Combine similar rules** to reduce redundancy and increase overall support.
   - **Example Action:**
     - For rules that specify exact chain lengths or positions, consider using **ranges** or **patterns** to include more compounds.

2. **Enhance Predictive Accuracy (Improve Confidence and Lift):**
   - **Identify Strong Predictors:**
     - Utilize statistical techniques (e.g., **correlation analysis, feature importance metrics**) to pinpoint variables with the **strongest influence** on the outcome.
     - Focus on these variables when formulating or modifying rules.
   - **Optimize Rule Conditions:**
     - Refine existing rules to **eliminate ambiguity** and enhance specificity where it contributes to higher confidence.

3. **Mitigate Overfitting Risks:**
   - **Simplify Rules:**
     - Avoid overly complex rules that may not generalize well to new data.
     - Ensure each rule has a balance between **complexity and generalizability**.
   - **Cross-Validation:**
     - Employ cross-validation techniques to test the rules on multiple subsets of data, ensuring they perform consistently.

4. **Integrate Additional Data or Features:**
   - **Expand Dataset:**
     - Incorporate more data points, possibly from **external datasets**, to provide a richer basis for rule generation.
   - **Add Relevant Features:**
     - Consider additional chemical descriptors or properties (e.g., **lipophilicity, electronic effects**) that may influence catalytic activity.

5. **Explore Alternative Rule-Induction Methods:**
  - **Machine Learning Algorithms:**
    - Use algorithms such as **decision trees, random forests, or association rule mining** to uncover patterns not evident through manual rule creation.
  - **Unsupervised Learning:**
    - Apply clustering methods to identify **natural groupings** in the data that may inform new rules.

6. **Re-evaluate Current Rules:**
  - **Rule Testing and Validation:**
    - Systematically test each rule against the data to assess its individual performance.
    - **Eliminate or revise** rules that contribute minimally or negatively to overall metrics.
  - **Incorporate Feedback:**
    - Engage domain experts to review the rules for **chemical plausibility** and **practical relevance**.

Specific Suggestions Based on Current Rules

- **Rule Consolidation:**
  - **Merge overlapping rules** to reduce complexity and improve support.
  - For example, Rules 1, 4, and 5 all deal with **chain length and functional group presence**; consider combining them into a comprehensive rule regarding aliphatic chain modifiers.

- **Adjust Rule Specificity:**
  - **Broaden overly specific conditions** to include more compounds.
  - For instance, Rules 7 and 11 focus on specific substituents (fluorination, polyethers) yielding low. Generalizing to "Modifications that increase steric hindrance or electronegativity reduce yield" could cover more cases.

- **Reconsider Underperforming Rules:**
  - **Eliminate or revise rules** that contribute to low support without significantly improving confidence or lift.
  - Rules with **limited examples** or **exceptions** (e.g., Rule 14) may need refinement.

- **Incorporate Positive Interactions:**
  - Identify combinations of features that **synergistically enhance** catalytic performance.
  - For example, modifiers with **both electron-donating groups and metal-coordinating abilities**.

Conclusion

The analysis indicates that the current set of rules has **not substantially improved** in terms of confidence and lift when compared to previous iterations, and the **decrease in support** suggests a **narrowing applicability**. To progress beyond this **local minimum**, it is essential to:

- **Reassess and broaden the rules** to cover more data points without sacrificing predictive power.
- **Leverage advanced analytical methods** and incorporate additional data to uncover new patterns.
- **Balance specificity and generality** to enhance both the practical utility and the predictive accuracy of the rules.

By implementing these recommendations, we can aim to develop a set of rules that not only predict high yields with greater confidence and reliability but also apply to a broader range of modifiers, thereby enhancing the overall effectiveness of the catalytic research program.

Next Steps:

- **Collaborate with team members** to discuss these findings and decide on the most feasible strategies to implement.
- **Prioritize actions** that are likely to yield the most significant improvements in rule performance.
- **Schedule iterative reviews** after implementing changes to continually assess progress and adjust the approach as needed.

Figure S31. Comments on the rules based on rule metrics.

Output Message

Traditional Calculator Message:

< 5 Fold Validation Performance: >

An accuracy of 0.45714285714285713 was obtained using classification model.

< SHAP Analysis Feedback >

For molecule C(CC(=O)O)[C@@H](C(=O)O)N, the predicted yield is low and the experimental value is high

Rule2 SHAP value for high loading: 0.037482648345899386; Rule5 SHAP value for high loading: -0.03748264834589954;

For molecule C1=CC(=CC=C1CBr)C(=O)O, the predicted yield is high and the experimental value is high

Rule2 SHAP value for high loading: 0.0657349348875376; Rule5 SHAP value for high loading: 0.07062870147609895;

For molecule C1=CC(=CC=C1C(=O)O)N, the predicted yield is high and the experimental value is high

Rule2 SHAP value for high loading: 0.0657349348875376; Rule5 SHAP value for high loading: 0.07062870147609895;

For molecule C([C@H]([C@H]([C@@H]([C@H](C(=O)O)O)O)O)O)O, the predicted yield is low and the experimental value is high

Rule2 SHAP value for high loading: 0.037482648345899386; Rule5 SHAP value for high loading: -0.03748264834589954;

For molecule C1=CC=C(C=C1)C[C@@H](C(=O)O)N, the predicted yield is high and the experimental value is low

Rule2 SHAP value for high loading: 0.0657349348875376; Rule5 SHAP value for high loading: 0.07062870147609895;

For molecule C1=CC(=CC=C1C(=O)NCC(=O)O)O, the predicted yield is high and the experimental value is low

Rule2 SHAP value for high loading: 0.0657349348875376; Rule5 SHAP value for high loading: 0.07062870147609895;

For molecule C(CCCC(=O)O)CCCN, the predicted yield is low and the experimental value is low

Rule2 SHAP value for high loading: 0.03071409950343756; Rule5 SHAP value for high loading: -0.13071409950343674;

For molecule C(CCN)CC(=O)O, the predicted yield is low and the experimental value is high

Rule2 SHAP value for high loading: 0.0008820679092235045; Rule5 SHAP value for high loading: -0.30088206790922123;

For molecule C1=CC2=C(C=C1C(=O)O)C(=O)OC2=O, the predicted yield is low and the experimental value is high

Rule2 SHAP value for high loading: -0.43682497714884944; Rule5 SHAP value for high loading: -0.06317502285114925;

For molecule C1=CC(=CC=C1C=O)C(=O)O, the predicted yield is low and the experimental value is low

Rule2 SHAP value for high loading: -0.43682497714884944; Rule5 SHAP value for high loading: -0.06317502285114925;

For molecule C1=CC(=CC=C1C(=O)O)[N+](=O)[O-], the predicted yield is low and the experimental value is high

Rule2 SHAP value for high loading: -0.43682497714884944; Rule5 SHAP value for high loading: -0.06317502285114925;

For molecule COC1=C(C=C(C=C1)C(=O)CCC(=O)O)OC, the predicted yield is low and the experimental value is high

Rule2 SHAP value for high loading: 0.02070923593349588; Rule5 SHAP value for high loading: -0.020709235933496002;

For molecule C(CC(=O)O)C(=O)CCC(=O)O, the predicted yield is high and the experimental value is low

Rule2 SHAP value for high loading: 0.03122284099170394; Rule5 SHAP value for high loading: 0.3021104923416311;

For molecule C1=CC(=CC=C1CCC(=O)O)O, the predicted yield is high and the experimental value is low

Rule2 SHAP value for high loading: 0.03122284099170394; Rule5 SHAP value for high loading: 0.3021104923416311;

For molecule C([C@@H](C(=O)O)N)C(=O)O, the predicted yield is high and the experimental value is high

Rule2 SHAP value for high loading: -0.01575858661686482; Rule5 SHAP value for high loading: 0.0871871580454371;

For molecule C1=CNC(=O)C=C1C(=O)O, the predicted yield is low and the experimental value is high

Rule2 SHAP value for high loading: -0.04548371025483702; Rule5 SHAP value for high loading: -0.05451628974516312;

For molecule C1=CC(=CC=C1C2=CC=NC=C2)C(=O)O, the predicted yield is low and the experimental value is low

Rule2 SHAP value for high loading: -0.06513470090281291; Rule5 SHAP value for high loading: -0.03486529909718768;

For molecule C(=O)(C(C(C(C(F)(F)F)(F)F)(F)F)(F)F)O, the predicted yield is low and the experimental value is low

Rule2 SHAP value for high loading: -0.020830584794126514; Rule5 SHAP value for high loading: 0.020830584794126184;

For molecule C1=CC=C2C(=C1)C(=CN2)C[C@H](C(=O)O)N, the predicted yield is low and the experimental value is high

Rule2 SHAP value for high loading: -0.04548371025483702; Rule5 SHAP value for high loading: -0.05451628974516312;

For molecule C1=CC(=CN=C1)C(=O)O, the predicted yield is low and the experimental value is high

Rule2 SHAP value for high loading: -0.04548371025483702; Rule5 SHAP value for high loading: -0.05451628974516312;

For molecule CC(=O)NCCCC[C@@H](C(=O)O)N, the predicted yield is low and the experimental value is low

Rule2 SHAP value for high loading: -0.020830584794126514; Rule5 SHAP value for high loading: 0.020830584794126184;

For molecule CCCCCCCC(=O)O, the predicted yield is low and the experimental value is high

Rule2 SHAP value for high loading: -0.025943377329192836; Rule5 SHAP value for high loading: -0.30977090838509214;

For molecule C(CS)C(=O)O, the predicted yield is high and the experimental value is high

Rule2 SHAP value for high loading: 0.00785588862914182; Rule5 SHAP value for high loading: 0.08142982565657256;

For molecule C1=CC(=C(C=C1C=O)C(=O)O)O, the predicted yield is high and the experimental value is low

Rule2 SHAP value for high loading: 0.00785588862914182; Rule5 SHAP value for high loading: 0.08142982565657256;

For molecule CC1=CC=C(C=C1)C(=O)O, the predicted yield is low and the experimental value is low

Rule2 SHAP value for high loading: -0.165072680666469; Rule5 SHAP value for high loading: 0.029358394952183797;

For molecule CSCC[C@@H](C(=O)NCC(=O)O)N, the predicted yield is high and the experimental value is low

Rule2 SHAP value for high loading: 0.00785588862914182; Rule5 SHAP value for high loading: 0.08142982565657256;

For molecule C1=CN=CC=C1CCCC(=O)O, the predicted yield is high and the experimental value is high

Rule2 SHAP value for high loading: 0.00785588862914182; Rule5 SHAP value for high loading: 0.08142982565657256;

For molecule C(COCCOCCN)C(=O)O, the predicted yield on catalyst is high and the experimental value is low

Rule2 SHAP value for high loading: 0.00785588862914182; Rule5 SHAP value for high loading: 0.08142982565657256;

For molecule C(C(=O)O)NC(CO)(CO)CO, the predicted yield is high and the experimental value is high

Rule2 SHAP value for high loading: -0.0021432815782388725; Rule5 SHAP value for high loading: 0.06642899586395318;

For molecule C1=CC(=CC=C1CCCCC(=O)O)O, the predicted yield is high and the experimental value is high

Rule2 SHAP value for high loading: -0.0021432815782388725; Rule5 SHAP value for high loading: 0.06642899586395318;

For molecule C1=CC(=CC=C1C(=O)O)S, the predicted yield is low and the experimental value is low

Rule2 SHAP value for high loading: -0.13668064854400283; Rule5 SHAP value for high loading: 0.0009663628297168683;

For molecule COCCOCCOCC(=O)O, the predicted yield is high and the experimental value is low

Rule2 SHAP value for high loading: -0.00950355074316629; Rule5 SHAP value for high loading: 0.019243810483426237;

For molecule C1=C(C=NN1)C(=O)O, the predicted yield is high and the experimental value is high

Rule2 SHAP value for high loading: -0.00950355074316629; Rule5 SHAP value for high loading: 0.019243810483426237;

For molecule C(CCCO)CCC(=O)O, the predicted yield is high and the experimental value is low

Rule2 SHAP value for high loading: -0.0021432815782388725; Rule5 SHAP value for high loading: 0.06642899586395318;

For molecule C(CCCN)CCC(=O)O, the predicted yield is low and the experimental value is low

Rule2 SHAP value for high loading: -0.01567118259371731; Rule5 SHAP value for high loading: -0.12004310312056754;

\---

Figure S32. ML feedback calculate on the feature matrix.

Output Message

ML Message:

Performance Evaluation of Current Rules

Upon reviewing the latest training results, several critical observations can be made regarding the performance of the current rules:

- **Significant Drop in Validation Accuracy**: The current 5-fold validation accuracy is **0.457**, which is substantially lower than the previous reference validation accuracies of **approximately 0.76**. Similarly, the test accuracy has decreased from values like **0.75** to **0.625** or even lower in previous runs.

- **Indication of Underfitting**: The marked decrease in validation accuracy suggests that the current rules are not capturing the underlying patterns necessary for accurate predictions. This underperformance implies that the model may be **underfitting**, struggling to generalize from the training data to unseen data.

- **Ineffectiveness of Current Rules**: The existing set of rules appears insufficient for the complexity of the dataset. The rules may be too simplistic, overly broad, or missing critical chemical features that influence the catalyst modifier loading.

SHAP Analysis and Detailed Insights

The SHAP (SHapley Additive exPlanations) values provide insights into how each rule contributes to the model's predictions for individual molecules. By analyzing the SHAP values, we can identify which rules are contributing to mispredictions and need refinement.

Analyzing Misclassifications

Underestimations (Predicted Low, Experimental High)

In these cases, the model predicts a **low loading**, but the experimental value shows a **high loading**. According to the guidance, the rule with the most **negative SHAP value** for high loading is likely attributing to the underestimation.

Examples:

1. **Molecule**: `C(CC(=O)O)[C@@H](C(=O)O)N`
  - **Rule2 SHAP**: +0.03748
  - **Rule5 SHAP**: **-0.03748**
  - **Analysis**: Rule 5 has a negative SHAP value, suggesting it's pushing the prediction towards low loading. This indicates that Rule 5 may not be adequately accounting for the features of this molecule.

2. **Molecule**: `C([C@H]([C@H]([C@@H]([C@H](C(=O)O)O)O)O)O)O`
  - **Rule2 SHAP**: +0.03748
  - **Rule5 SHAP**: **-0.03748**
  - **Analysis**: Similar to the first molecule, Rule 5 negatively impacts the prediction, leading to underestimation.

3. **Molecule**: `C1=CC(=C(C=C1C=O)C(=O)O)O`
  - **Rule2 SHAP**: -0.43682
  - **Rule5 SHAP**: -0.06317
  - **Analysis**: Both Rule 2 and Rule 5 have negative SHAP values, with Rule 2 having a more significant impact. This suggests issues with both rules for this molecule.

4. **Molecule**: `C1=CNC(=O)C=C1C(=O)O`
  - **Rule2 SHAP**: **-0.04548**
  - **Rule5 SHAP**: -0.05451
  - **Analysis**: Rule 2 has a notable negative SHAP value, indicating it may be incorrectly influencing the prediction towards low loading.

Overestimations (Predicted High, Experimental Low)

In these cases, the model predicts a **high loading**, but the experimental value shows a **low loading**. The rule with the most **positive SHAP value** for high loading is likely causing the overestimation.

Examples:

1. **Molecule**: `C(CCCO)CCC(=O)O`
   - **Rule2 SHAP**: -0.00214
   - **Rule5 SHAP**: **+0.06642**
   - **Analysis**: Rule 5 has a positive SHAP value, pushing the prediction towards high loading when it should be low, indicating a potential issue with Rule 5.

2. **Molecule**: `C(CC(=O)O)C(=O)CCC(=O)O`
   - **Rule2 SHAP**: +0.03122
   - **Rule5 SHAP**: **+0.30211**
   - **Analysis**: Rule 5 is significantly contributing to the overestimation, suggesting it may need refinement.

3. **Molecule**: `C(CCCO)CC(=O)O`
   - **Rule2 SHAP**: -0.01567
   - **Rule5 SHAP**: **+0.08718**
   - **Analysis**: Positive SHAP value from Rule 5 leads to overestimation.

Patterns Identified

- **Rule 5**:
  - Frequently exhibits **negative SHAP values** in underestimations and **positive SHAP values** in overestimations.
  - Suggests that Rule 5 is contributing inconsistently to the predictions, leading to misclassifications.

- **Rule 2**:
  - Shows significant **negative SHAP values** in several underestimations.
  - Indicates that Rule 2 might be suppressing the prediction of high loading when it is actually high.

Overfitting/Underfitting Assessment

The substantial decrease in validation accuracy without evidence of high training accuracy suggests that the model is **underfitting**. This means:

- The model lacks complexity to capture the underlying data patterns.
- The current rules do not encompass all necessary features influencing the target variable.
- Additional rules or features may be required to improve predictive performance.

Recommendations for Improvement

Based on the SHAP analysis and performance evaluation, the following recommendations are proposed to enhance the current rules:

1. Refine Rule 5

Current Rule 5:

- **Description**: Modifiers with multiple hydroxyl groups on short chains yield high; single hydroxyl on long chains yields low.
- **Issue**: Rule 5 contributes to both overestimations and underestimations, indicating that it may be too general or misclassified.

Suggested Refinements:

- **Specify Chain Length and Hydroxyl Positions**:
  - Precisely define what constitutes a "short chain" (e.g., chains with 3-5 carbons).
  - Detail the positions of hydroxyl groups that promote high loading.
  - Example: *Modifiers with multiple hydroxyl groups on chains of 3-5 carbons, with hydroxyls at adjacent carbons, yield high loading.*

- **Introduce Exceptions**:
  - Identify molecules that do not conform to Rule 5 and adjust the rule to exclude them.
  - For instance, molecules with multiple hydroxyl groups but additional functional groups that interfere with loading should be excluded.

- **Consider Steric Effects**:
  - Assess how steric hindrance from bulky groups may affect the applicability of Rule 5.
  - Modify the rule to account for steric factors that could lower loading despite multiple hydroxyl groups.

2. Adjust Rule 2

Current Rule 2:

- **Description**: Para-substituted benzoic acids with electron-withdrawing, metal-coordinating groups yield high; electron-donating or non-coordinating groups yield low.
- **Issue**: Rule 2 sometimes incorrectly contributes to low loading predictions when the experimental value is high.

Suggested Refinements:

- **Clarify Electron-Withdrawing Groups**:
  - Provide a specific list of substituents considered electron-withdrawing and capable of metal coordination.
  - Example: *Nitro, amino, and halogen groups at the para position yield high loading.*

- **Differentiate between Substituents**:
  - Recognize that not all electron-withdrawing groups equally impact loading.
  - Adjust the rule to reflect the relative strength of different substituents.

- **Incorporate Ortho and Meta Positions**:
  - Consider the effect of substituents at positions other than para.
  - Expand the rule to include molecules where the position of the substituent influences loading.
3. Introduce Additional Rules

Identify Missing Chemical Features:

- **Heterocyclic Compounds**: Some underestimations involve heterocyclic carboxylic acids. Develop rules that better capture the behavior of such compounds.
  - **New Rule Suggestion**: *Modifiers that are nitrogen-containing heterocyclic carboxylic acids with ring systems capable of resonance stabilization yield high loading.*

Address Long-Chain Aliphatic Acids:

- The molecule `CCCCCCCC(=O)O` (octanoic acid) is underestimated. Consider adding or refining a rule to capture the effect of longer aliphatic chains.

  - **New Rule Suggestion**: *Long-chain aliphatic carboxylic acids (7 carbons or more) without polar functional groups yield high loading due to increased hydrophobic interactions with the catalyst.*

4. Improve Generalization

Avoid Overfitting Specific Cases:

- Ensure that rules are not overly tailored to specific molecules at the expense of general applicability.

Increase Rule Complexity Where Necessary:

- Introduce quantitative descriptors or thresholds (e.g., electronegativity values, pKa ranges) to make rules more precise.

Consider Multivariate Interactions:

- Explore rules that account for interactions between multiple functional groups within a molecule.

5. Utilize SHAP Analysis for Rule Refinement

Iterative Refinement Process:

- Continue using SHAP values to identify problematic rules in future iterations.
- Focus on rules that consistently contribute to mispredictions.

Feedback Loop Between Model and Rules:

- Use model performance and SHAP insights to inform rule adjustments.
- After modifying rules, retrain the model and reassess SHAP contributions.

6. Re-Evaluate Molecules with High Misclassification Rates

Investigate Outliers:

- Examine molecules that are frequently misclassified to identify unique features not captured by current rules.

Potentially Exclude or Specially Treat Outliers:

- If certain molecules do not conform to any rule patterns, consider treating them as exceptions or developing specific rules.

Conclusion

The current set of rules demonstrates limitations in accurately predicting the catalyst modifier loading, as evidenced by the decreased validation accuracy and SHAP analysis. To address these issues:

- **Refine existing rules** like Rule 5 and Rule 2 to be more precise and better reflect the chemical behavior influencing loading.
- **Introduce new rules** to capture missing features and improve the model's ability to generalize.
- **Use SHAP analysis** systematically to guide rule adjustments and understand the impact of each rule.
- **Adopt an iterative approach** to model training and rule refinement, ensuring continuous improvement in predictive performance.

By implementing these recommendations, it is anticipated that the model's accuracy will improve, moving closer to or surpassing previous validation accuracies. Continuously refining the rules based on empirical evidence and model feedback will enhance the robustness and reliability of the predictive framework for catalyst modifier loading.

Figure S33. Comments to the rules based on the ML feedback.

Output Message

Project Manager Message:

Summary of Discussions and Directions for Rule Optimization

1. Performance Evaluation of Current Rules

The current set of rules for predicting the yield of the radical-mediated remote δ-C(sp^3)–H bond functionalization reaction shows suboptimal performance, as evidenced by the metrics and analyses provided:

- **Significant Drop in Validation Accuracy**: The 5-fold validation accuracy has decreased to **45.71%**, a notable decline from previous accuracies of approximately **76%**. This indicates that the model's predictive capability has diminished.

- **SHAP Analysis Highlights Misclassifications**: The SHAP (SHapley Additive exPlanations) analysis reveals that certain rules, particularly **Rule 2** and **Rule 5**, are contributing to both overestimations and underestimations of the modifier loading on the catalyst. This inconsistency suggests that these rules may not be accurately capturing the underlying chemical behavior.

- **Underfitting Indication**: The low validation accuracy, coupled with no evidence of overfitting (i.e., training accuracy is not significantly higher than validation accuracy), suggests that the model is underfitting. It lacks the complexity necessary to model the data adequately.

2. Analysis of Specific Rules

- **Rule 2 (Para-Substituted Benzoic Acids)**:
  - **Issue**: Rule 2 sometimes incorrectly predicts low loading for molecules that experimentally have high loading. The SHAP values indicate that Rule 2 contributes negatively in these cases.
  - **Possible Cause**: The current SMARTS patterns may not be accurately identifying all relevant functional groups or their positions, particularly the para-substitution pattern and metal-coordinating capabilities.

- **Rule 5 (Modifiers with Multiple Hydroxyl Groups)**:
  - **Issue**: Rule 5 contributes to misclassifications by both overestimating and underestimating the yield. It seems to inconsistently predict the effect of hydroxyl groups on different chain lengths.
  - **Possible Cause**: The rule may be too general or not adequately specifying the effect of chain length and the number or position of hydroxyl groups.

3. Suggestions to Improve Current Rules

A. Refine Rule 2

- **Clarify Substituent Positions**: Ensure that the SMARTS patterns specifically identify para-substituted benzoic acids. Modify the pattern to target substituents at the para position relative to the carboxylic acid group.

- **Update Metal-Coordinating Groups**: Expand or refine the list of electron-withdrawing, metal-coordinating substituents. Explicitly include groups known to coordinate with metals (e.g., amino, nitro, halogens like bromine).

- **Consider Other Positions**: Examine whether substitutions at ortho or meta positions affect yield and update the rule accordingly.

B. Refine Rule 5

- **Specify Chain Length and Hydroxyl Group Count**: Clearly define what constitutes a "short chain" (e.g., 3-5 carbons) and stipulate the minimum number of hydroxyl groups required to impact yield positively.

- **Detail Hydroxyl Group Positions**: Indicate that hydroxyl groups should be on adjacent carbons or in specific configurations that favor interaction with the catalyst.

- **Exclude Confounding Functional Groups**: Modify the rule to exclude molecules where additional functional groups negate the positive effect of multiple hydroxyl groups.

C. Introduce New Rules or Modify Existing Ones

- **Rule for Heterocyclic Compounds with Metal-Coordinating Ability**:
  - **Suggestion**: Develop a rule that captures the behavior of nitrogen-containing heterocyclic carboxylic acids, as they have shown high yield due to their ability to coordinate with metal centers.

- **Rule for Long-Chain Aliphatic Carboxylic Acids**:
  - **Suggestion**: Create or adjust a rule that addresses long-chain aliphatic acids without polar functional groups, which may yield high due to hydrophobic interactions enhancing catalyst interaction.

D. Improve SMARTS Patterns Across Rules

- **Ensure Accurate Representation**: Review and update all SMARTS patterns to precisely match the chemical structures described in the rules. This includes correctly capturing chain lengths, functional group positions, and connectivity.

- **Test SMARTS Patterns**: Validate the updated SMARTS patterns against a set of known molecules to ensure they match the intended structures and exclude incorrect ones.

E. Incorporate Additional Chemical Descriptors

- **Consider Steric and Electronic Effects**: Introduce descriptors or thresholds for steric hindrance (e.g., branching, bulky groups) and electronic properties (e.g., electronegativity, resonance effects) to enhance rule specificity.

- **Utilize Quantitative Structure-Property Relationships (QSPR)**: Integrate quantitative data where possible to refine rules based on measurable properties.

F. Re-evaluate and Simplify Rules

- **Consolidate Overlapping Rules**: Combine rules that cover similar chemical features to increase support and reduce complexity.

- **Eliminate Redundant or Ineffective Rules**: Remove rules that do not contribute significantly to improving predictive performance or that have low support and confidence.

4. Potential for Further Optimization Without Overfitting

There is potential for further optimization of the current rules without overfitting:

- **Addressing Underfitting**: Since the model is underfitting, increasing its complexity by refining the rules should improve performance. This involves making the rules more precise and better aligned with the underlying chemical principles.

- **Balancing Specificity and Generality**: By carefully refining the rules to be neither too broad nor too narrow, we can improve the model's ability to generalize without overfitting.

- **Iterative Testing and Validation**: Applying changes iteratively and validating the model's performance at each step helps prevent overfitting.

- **Incorporating Domain Expertise**: Collaborating with chemists to ensure that the rules make chemical sense can improve their effectiveness and applicability.

Conclusion

The current analysis indicates that the rules can be further optimized to enhance the model's predictive capabilities. By refining the existing rules, introducing new ones where necessary, and improving the specificity of SMARTS patterns, we can address the underfitting issue and improve both the accuracy and reliability of the predictions.

Please Optimize Rules

Figure S34. Summarization of this iteration given by Project Manager.

Output Message

Rule Advisor Message:

** Start of Advice **

- **Advice 1**: **Refine Rule 2 for Para-Substituted Benzoic Acids**

  - **Update SMARTS Patterns**: Modify the SMARTS patterns in Rule 2 to specifically identify para-substituted benzoic acids with electron-withdrawing, metal-coordinating groups at the para position relative to the carboxylic acid.
    - **Include Metal-Coordinating Groups**: Ensure the patterns include substituents like amino (`-NH2`), nitro (`-NO2`), halogens (e.g., bromo), and bromomethyl groups.
    - **Exclude Electron-Donating Groups**: Exclude modifiers with electron-donating groups (e.g., methyl, methoxy) or non-coordinating substituents.
  - **Example SMARTS Pattern for High Yield**:
    - `C1=CC([NX3;H2,H1;!$(N-C=O),OX1,O,NX2])=CC=C1C(=O)O`
      - This pattern matches benzoic acids with a metal-coordinating group (N or O donor) at the para position.
  - **Validation**: Test the updated SMARTS patterns against known high and low yield modifiers to ensure accurate classification.

- **Advice 2**: **Refine Rule 5 for Multiple Hydroxyl Groups on Short Chains**

  - **Specify Chain Length and Hydroxyl Count**: Define "short aliphatic chains" as chains with 5 or fewer carbons and specify that high yield requires multiple (e.g., 3 or more) hydroxyl groups.
  - **Detail Hydroxyl Group Positions**: Indicate that hydroxyl groups should be on adjacent carbons to enhance interaction with the catalyst.
  - **Update SMARTS Pattern**:
    - High Yield: `O[C@@H]([C@@H](O)[C@@H](O)[C@H](O)CO)C(=O)O`
      - Matches modifiers like gluconic acid with multiple hydroxyls on a short chain.
    - Low Yield Exclusion: Ensure that compounds with a single hydroxyl on long chains (e.g., 7-hydroxyheptanoic acid) do not match the high-yield pattern.
  - **Exclude Confounding Functional Groups**: Modify the rule to exclude modifiers with additional polar groups that may interfere with catalyst coordination.

- **Advice 3**: **Improve SMARTS Patterns Across All Rules**

  - **Accurate Representation**: Review and update all SMARTS patterns to precisely match the chemical structures described in the rules.
  - **Testing SMARTS Patterns**: Validate each pattern against known modifiers to confirm they match intended structures and exclude incorrect ones.
  - **Example**: For Rule 1 (Short-Chain Aliphatic Amino Acids):
    - High Yield Pattern: `N[C@@H](C(=O)O)C`
      - Matches short-chain amino acids like alanine and aspartic acid.
    - Exclude longer chains by limiting the pattern to a specific number of carbons.

- **Advice 4**: **Introduce a New Rule for Nitrogen-Containing Heterocyclic Carboxylic Acids**

  - **Rule Statement**: Modifiers that are nitrogen-containing heterocyclic carboxylic acids (e.g., pyridine, pyrazole carboxylic acids) yield high due to effective metal coordination.
  - **SMARTS Pattern**:
    - High Yield: `[nX2]1cccc1C(=O)O`
      - Matches pyridine carboxylic acids.
    - `[nH]1nccc1C(=O)O`
      - Matches pyrazole carboxylic acids.
  - **Validation**: Ensure these patterns accurately identify known high-yield modifiers like niacin and exclude non-relevant compounds.

- **Advice 5**: **Adjust Rule 4 for Straight-Chain Aliphatic Carboxylic Acids**

  - **Emphasize Chain Length and Lack of Polar Groups**: Specify that high yield is associated with unbranched aliphatic carboxylic acids without additional polar functional groups.
  - **Update SMARTS Pattern**:
    - High Yield: `CCCCCCCC(=O)O`
      - Matches octanoic acid and similar long-chain acids.
    - Exclude Low Yield Modifiers: Modify the pattern to exclude acids with hydroxyl or amino groups (e.g., `C[CX4][CX4][CX4][CX4][CX4]C(=O)O`).
  - **Clarify Rule Scope**: Clearly state that the presence of polar groups on the aliphatic chain lowers yield due to interference with catalytic activity.

- **Advice 6**: **Re-evaluate and Simplify Rules**

  - **Consolidate Overlapping Rules**: Identify rules covering similar chemical features (e.g., Rules 9 and 15 regarding heterocycles) and merge them to increase clarity and support.
  - **Remove Ineffective Rules**: Analyze the impact of each rule on the model's performance and eliminate those with low contribution or redundant scope.
  - **Prioritize High-Impact Rules**: Focus on rules that have strong support from both the data and chemical reasoning.

- **Advice 7**: **Incorporate Additional Chemical Descriptors**

  - **Steric Hindrance**: Introduce descriptors for steric bulk (e.g., branching, bulky substituents) to account for modifiers that may hinder catalyst access.
  - **Electronic Effects**: Include parameters like electronegativity or resonance effects to quantify the impact of substituents on metal coordination.
  - **Implement QSPR Models**: Use Quantitative Structure-Property Relationship models to provide a quantitative basis for rule refinement.

- **Advice 8**: **Iteratively Test and Validate Model Improvements**

  - **Retrain the Model**: After refining rules and SMARTS patterns, retrain the model to assess improvements in predictive accuracy.
  - **Cross-Validation**: Use k-fold cross-validation to evaluate model performance and ensure changes address underfitting without overfitting.
  - **SHAP Analysis**: Re-run SHAP analysis to identify any remaining misclassifications and refine rules accordingly.

- **Advice 9**: **Collaborate with Chemists for Expert Input**

  - **Expert Review**: Engage with chemists to ensure that all rules and patterns align with chemical intuition and experimental observations.
  - **Incorporate Feedback**: Use their insights to adjust rules, especially in cases where data is limited or ambiguous.
  - **Continuous Improvement**: Establish a feedback loop for ongoing rule refinement as new data becomes available.

- **Advice 10**: **Document All Changes and Rationales**

  - **Change Log**: Maintain detailed records of all rule modifications, including changes to SMARTS patterns and the reasons behind them.
  - **Version Control**: Use version control systems to track changes and enable rollback if needed.
  - **Transparency**: Ensure documentation is clear and accessible to all team members for collaboration and future reference.

** End of Advice **

Figure S35. Advice based on the latest iteration to start the next iteration.

11. Traditional and LLM results on Fe/Hf

Traditional descriptors achieved a test accuracy of 0.81 via LOO process. SHAP analysis of the selected features reveals that a small standard deviation in the atomic PEOE charge corresponds to a high Fe/Hf ratio, while a large standard deviation is associated with a low Fe/Hf ratio (Figure S36). However, the remaining four features do not exhibit a clear trend.

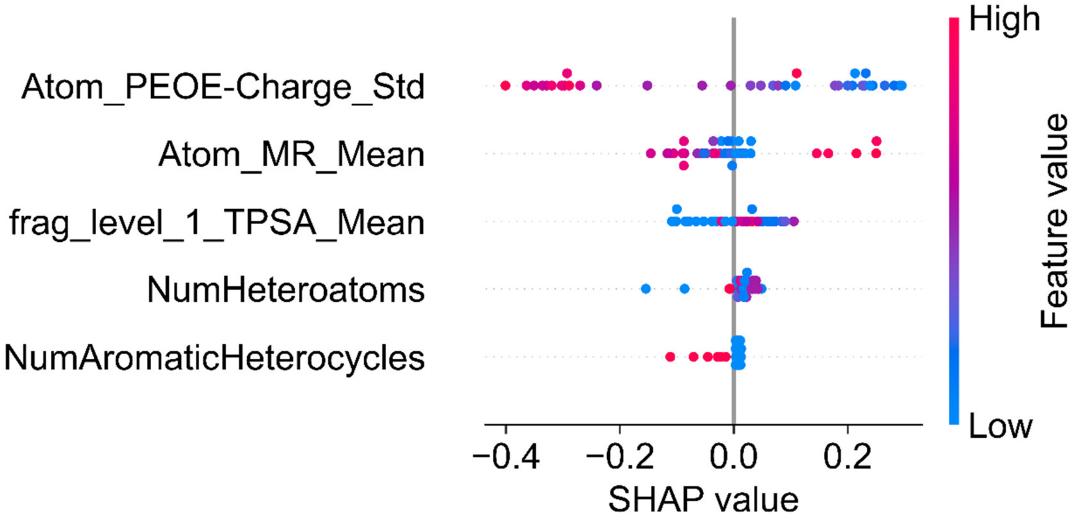

Figure S36. SHAP analysis of transitional descriptors targeting Fe/Hf.

We also applied the LLM pipeline and traditional featurization method to predict the amount of Fe loading, characterized by Fe/Hf. The LLM pipeline achieved an LOO accuracy of 0.75. The six rules with the most significant SHAP values are presented in 错误!未找到引用源。.

Table S6. SHAP value of top LLM rules for Fe/Hf.

Rule	SHAP value
Modifiers containing sulfur function groups, such as thiol (–SH) or thioether (–S–) groups, lead to high Fe/Hf values.	-0.035
Modifiers with hydroxyl groups on flexible aliphatic chains of five or more carbon atoms result in a high Fe/Hf value.	0.033
Para-substituted benzoic acids with electron-withdrawing groups (e.g., nitro, halogen, sulfanyl, pyridyl) result in a high Fe/Hf ratio.	-0.030
Modifiers containing multiple hydroxyl (-OH) groups in close proximity (e.g., polyols or sugars) or phenolic hydroxyl groups on aromatic rings lead to low Fe/Hf values.	0.029
Modifiers with multiple adjacent hydroxyl groups (polyols) decrease Fe/Hf (Low Fe/Hf).	0.029
Modifiers containing carbonyl groups adjacent to aromatic rings (e.g., keto	0.027

groups, lactones) predict a high Fe/Hf value due to potential chelation with Fe ions.	

The most significant SHAP value is -0.035, and the least significant value is 0.001.

These rules illustrate how specific functional groups correlate with the Fe/Hf ratio in the dataset. However, the random forest model trained on a subset of these rules achieves an LOO accuracy of only 0.67 on the test set.

12. Two distinctive fingerprints from MACCS for Fe/Hf

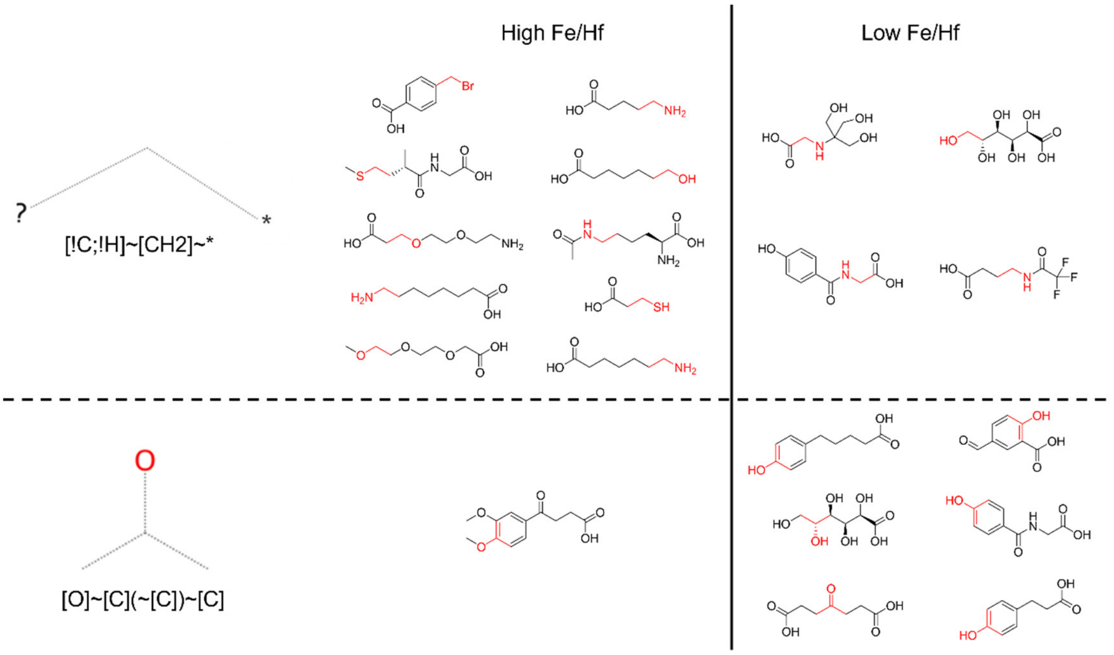

Figure S37. Distinctive patterns differentiating modifiers with high and low Fe loading values.

Two SMARTS patterns highlight the distinction between modifiers with high and low Fe/Hf values. Modifiers containing coordinating groups such as amino, hydroxyl, and sulfhydryl groups exhibit high Fe/Hf values due to their ability to stabilize chelated Fe ions on the TPY site through strong coordinating interactions. These groups also provide additional coordination sites for Fe, enabling the capture of more Fe ions. However, despite the strong coordinating interaction between phenolic hydroxyl groups and Fe ions, modifiers with phenolic hydroxyl groups demonstrate relatively low Fe/Hf values. This suggests that the Fe ion loading is significantly influenced by steric effects, which hinder efficient coordination in these cases.

13. Dataset

Table S7. Carboxylic acid molecules used as modifiers.

Index	Carboxylic acid molecule	CAS	Molecular formula	Source/Brand
1	Perfluoropentanoic acid	2706-90-3	$C_5HF_9O_2$	Bidepharm
2	L-Cysteic Acid	498-40-8	$C_3H_7NO_5S$	Bidepharm
3	D-Tryptophan	153-94-6	$C_{11}H_{12}N_2O_2$	Bidepharm
4	L-Glutamic acid	56-86-0	$C_5H_9NO_4$	Bidepharm
5	4-Pyrazolecarboxylic acid	37718-11-9	$C_4H_4N_2O_2$	Bidepharm
6	4-Mercaptobenzoic acid	1074-36-8	$C_7H_6O_2S$	Bidepharm
7	2-(2-(2-Methoxyethoxy)ethoxy)acetic acid	16024-58-1	$C_7H_{14}O_5$	Bidepharm
8	4-(4-Pyridyl)benzoic acid	4385-76-6	$C_{12}H_9NO_2$	Bidepharm
9	4-Bromomethylbenzoic acid	6232-88-8	$C_8H_7BrO_2$	Bidepharm
10	L-Aspartic acid	56-84-8	$C_4H_7NO_4$	Bidepharm
11	L-Phenylalanine	63-91-2	$C_9H_{11}NO_2$	Bidepharm
12	5-Aminovaleric acid	660-88-8	$C_5H_{11}NO_2$	Bidepharm
13	4-Aminobenzoic acid	150-13-0	$C_7H_7NO_2$	Bidepharm
14	Octanoic acid	124-07-2	$C_8H_{16}O_2$	Bidepharm
15	Tricine	5704-04-1	$C_6H_{13}NO_5$	Bidepharm

Index	Carboxylic acid molecule	CAS	Molecular formula	Source/Brand
16	5-(4-Hydroxyphenyl)pentanoic acid	4654-08-4	$C_{11}H_{14}O_3$	Bidepharm
17	2-Hydroxyisonicotinic acid	22282-72-0	$C_6H_5NO_3$	Bidepharm
18	Gluconic acid	526-95-4	$C_6H_{12}O_7$	Bidepharm
19	Trimellitic Anhydride	552-30-7	$C_9H_4O_5$	Bidepharm
20	3-Mercaptopropionic acid	107-96-0	$C_3H_6O_2S$	Bidepharm
21	5-Formylsalicylic acid	616-76-2	$C_8H_6O_4$	Bidepharm
22	4-Formylbenzoic acid	619-66-9	$C_8H_6O_3$	Acmec
23	4-Nitrobenzoic acid	62-23-7	$C_7H_5NO_4$	Acmec
24	p-Toluic acid	99-94-5	$C_8H_8O_2$	Acmec
25	Nicotinic acid	59-67-6	$C_6H_5NO_2$	Acmec
26	8-Aminooctanoic acid	1002-57-9	$C_8H_{17}NO_2$	Acmec
27	7-Aminoheptanoic acid	929-17-9	$C_7H_{15}NO_2$	Acmec
28	L-Methionylglycine	14486-03-4	$C_7H_{14}N_2O_3S$	Acmec
29	4-Pyridin-4-yl-butyric acid	102878-73-9	$C_9H_{11}NO_2$	Acmec
30	3-(3,4-Dimethoxybenzoyl) propionic acid	5333-34-6	$C_{12}H_{14}O_5$	Acmec
31	4-Hydroxyhippuric acid	2482-25-9	$C_9H_9NO_4$	Energy Chemical
32	Amino-PEG2-acid	791028-27-8	$C_7H_{15}NO_4$	Energy Chemical
33	Heptanedioic acid	502-50-1	$C_7H_{10}O_5$	Energy Chemical

Index	Carboxylic acid molecule	CAS	Molecular formula	Source/Brand
34	4-(2,2,2-TrifluoroacetaMido)butanoic acid	50632-83-2	$C_6H_8F_3NO_3$	Energy Chemical
35	7-Hydroxyheptanoic acid	3710-42-7	$C_7H_{14}O_3$	Energy Chemical
36	N^{ε} -Acetyl-L-lysine	692-04-6	$C_8H_{16}N_2O_3$	Energy Chemical
37	3-(4-Hydroxyphenyl) propionic acid	501-97-3	$C_9H_{10}O_3$	Energy Chemical

Table S8. ICP-OES of the catalyst and SBU modified carboxylic acid molecular ratio.

Index	Carboxylic acid molecule	The amount of Fe added	Modification ratio (molecular /SBUs)
1	Perfluoropentanoic acid	0.37 μmol	-
2	L-Cysteic Acid	0.27 μmol	0.7
3	D-Tryptophan	0.36 μmol	2.4
4	L-Glutamic acid	0.83 μmol	2.0
5	4-Pyrazolecarboxylic acid	1.35 μmol	1.1
6	4-Mercaptobenzoic acid	0.87 μmol	3.0
7	2-(2-(2-Methoxyethoxy)ethoxy)acetic acid	0.36 μmol	1.6
8	4-(4-Pyridyl)benzoic acid	0.43 μmol	0.7

Index	Carboxylic acid molecule	The amount of Fe added	Modification ratio (molecular /SBUs)
9	4-Bromomethylbenzoic acid	0.29 μmol	0.3
10	L-Aspartic acid	0.41 μmol	1.8
11	L-Phenylalanine	0.42 μmol	0.8
12	5-Aminovaleric acid	0.30 μmol	0.4
13	4-Aminobenzoic acid	0.22 μmol	1.2
14	Octanoic acid	0.60 μmol	1.6
15	Tricine	0.66 μmol	0.3
16	5-(4-Hydroxyphenyl)pentanoic acid	0.54 μmol	1.2
17	2-Hydroxyisonicotinic acid	0.65 μmol	3.0
18	Gluconic acid	0.65 μmol	1.7
19	Trimellitic Anhydride	0.59 μmol	1.6
20	3-Mercaptopropionic acid	0.80 μmol	2.4
21	5-Formylsalicylic acid	0.38 μmol	0.6
22	4-Formylbenzoic acid	0.36 μmol	1.8
23	4-Nitrobenzoic acid	0.86 μmol	2.3
24	p-Toluic acid	0.44 μmol	2.0
25	Nicotinic acid	0.60 μmol	2.6
26	8-Aminooctanoic acid	0.30 μmol	1.0

Index	Carboxylic acid molecule	The amount of Fe added	Modification ratio (molecular /SBUs)
27	7-Aminoheptanoic acid	0.29 μmol	0.6
28	L-Methionylglycine	0.29 μmol	0.4
29	4-Pyridin-4-yl-butyric acid	0.59 μmol	2.5
30	3-(3,4-Dimethoxybenzoyl) propionic acid	0.44 μmol	1.4
31	2-[(4-Hydroxyphenyl)carbonyl amino]ethanoic acid	0.20 μmol	1.5
32	Amino-PEG2-acid	0.26 μmol	0.6
33	Heptanedioic acid	0.16 μmol	0.8
34	4-(2,2,2-TrifluoroacetaMido)butanoic acid	0.18 μmol	1.9
35	7-Hydroxyheptanoic acid	0.26 μmol	2.0
36	N^{ε}-Acetyl-L-lysine	0.28 μmol	0.8
37	3-(4-Hydroxyphenyl) propionic acid	0.15 μmol	2.1

14. ^{1}H-NMR and GC-MS data

Procedure: 3 mg of the catalyst was weighed and washed with acetone more than three times, then dried in a vacuum drying oven. Following this, 50 μL of a K_3PO_4/D_2O solution was added to the solid catalyst, causing it to dissolve. Afterward, deuterated reagents (D_2O or DMSO-d_6) were introduced, and mesitylene was used as the internal standard for quantification. The mixture was

then filtered through a membrane and prepared for ^{1}H-NMR analysis.

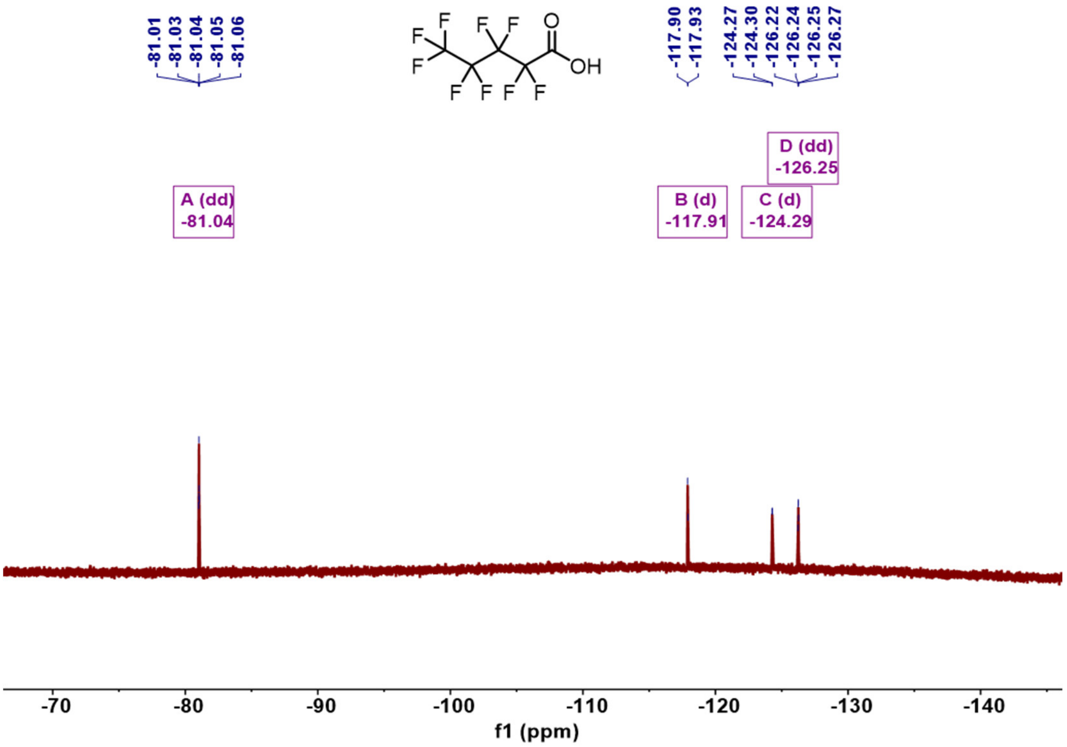

Figure S38. ^{19}F-NMR analysis after digestion of Hf-TPY-MOL-perfluoropentanoic acid (CAS: 2706-90-3).

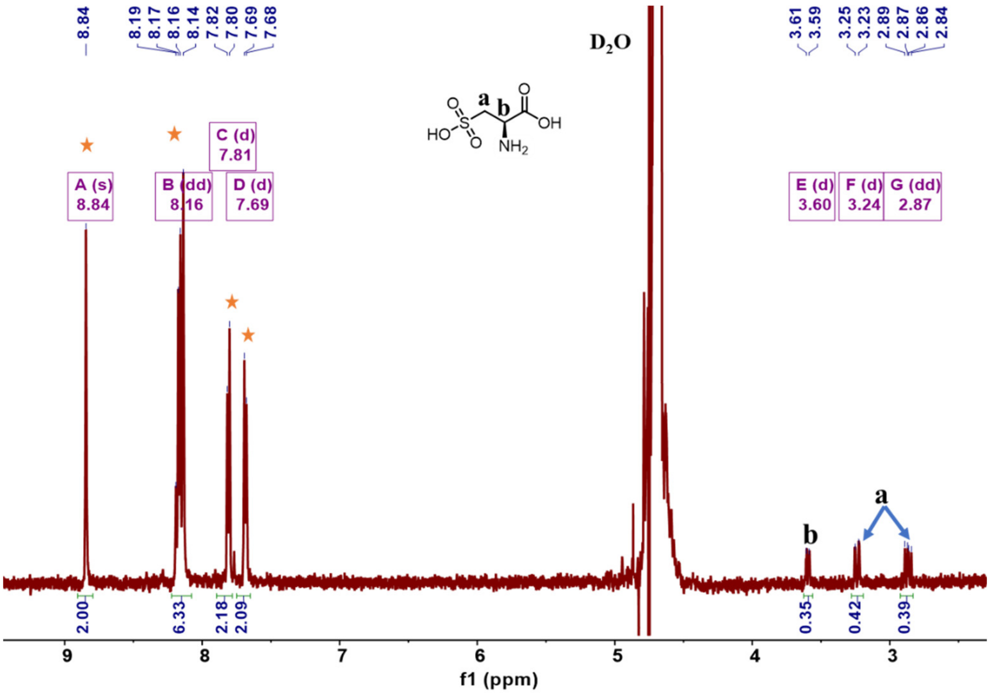

Figure S39. ^{1}H-NMR analysis after digestion of Hf-TPY-MOL-L-cysteic acid (CAS: 498-40-8).
★: ^{1}H-NMR peaks for H_3TPY, a-b indicate peaks of corresponding Hs on the molecule.

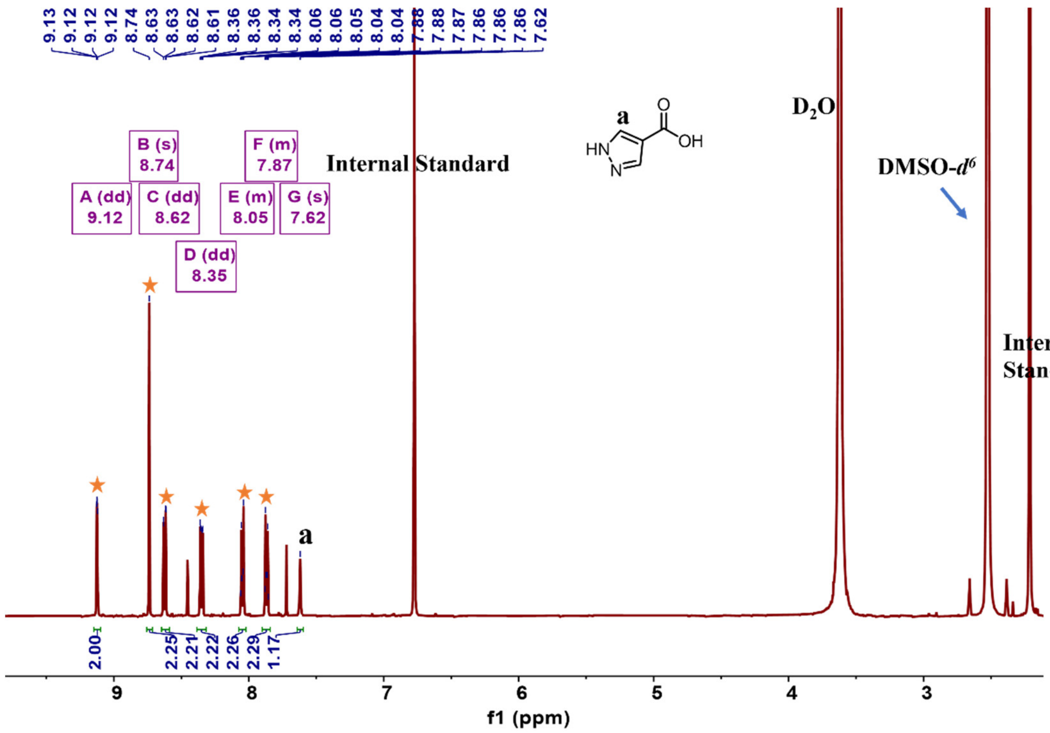

Figure S40. ^{1}H-NMR analysis after digestion of Hf-TPY-MOL-4-pyrazolecarboxylic acid (CAS: 37718-11-9). ★: ^{1}H-NMR for H_3TPY, a indicates the peak of the corresponding H on the molecule.

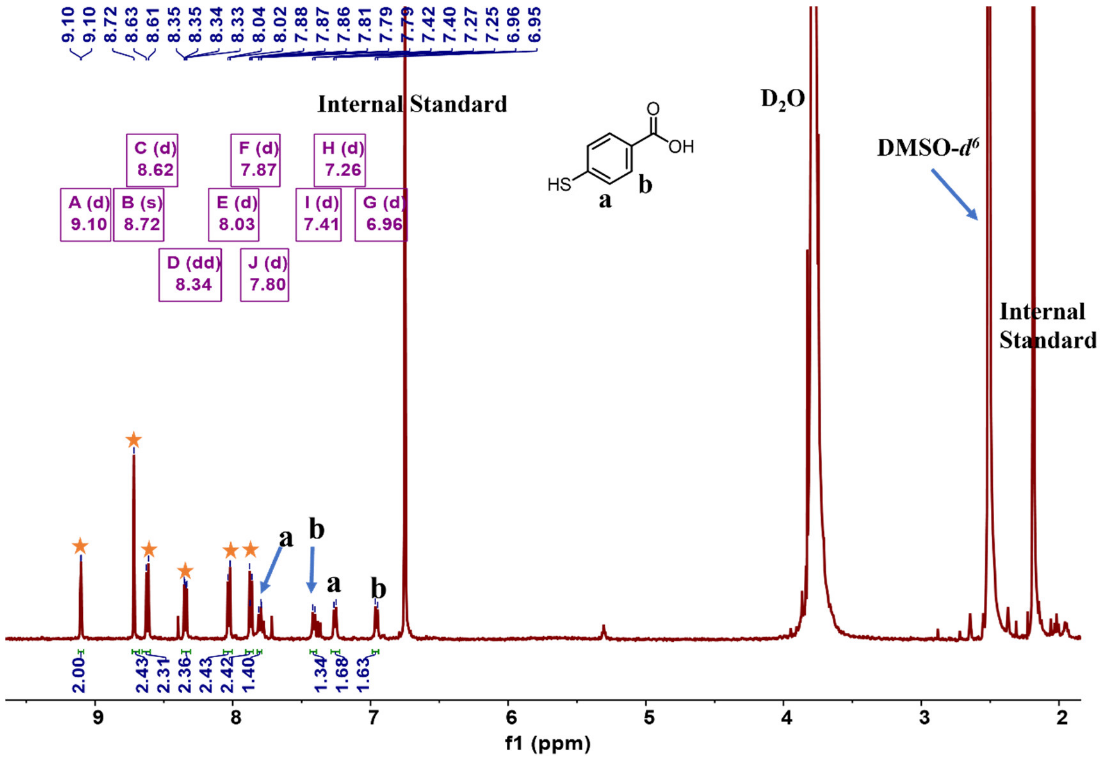

Figure S41. ^{1}H-NMR analysis after digestion of Hf-TPY-MOL-4-mercaptobenzoic acid (CAS:

1074-36-8). ★: ^{1}H-NMR peaks for H_3TPY, a-b indicate peaks of corresponding Hs on the molecule.

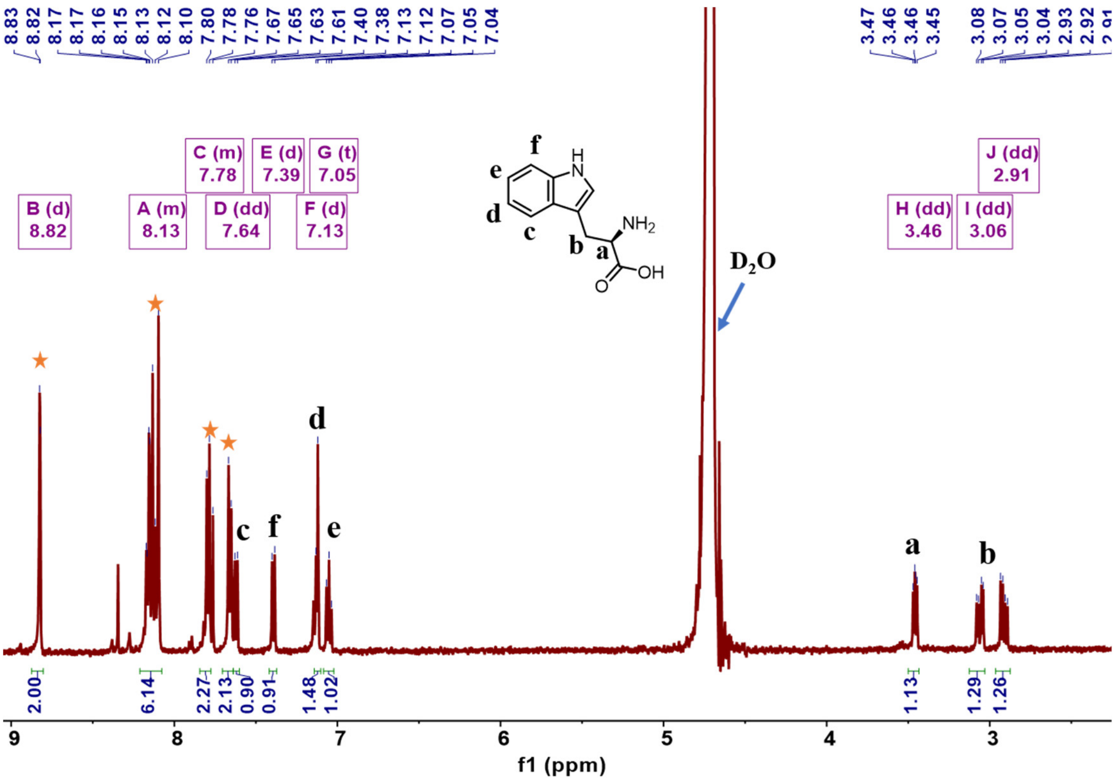

Figure S42. ^{1}H-NMR analysis after digestion of Hf-TPY-MOL-D-tryptophan (CAS: 153-94-6). ★ : ^{1}H-NMR peaks for H_3TPY, a-f indicate peaks of corresponding Hs on the molecule.

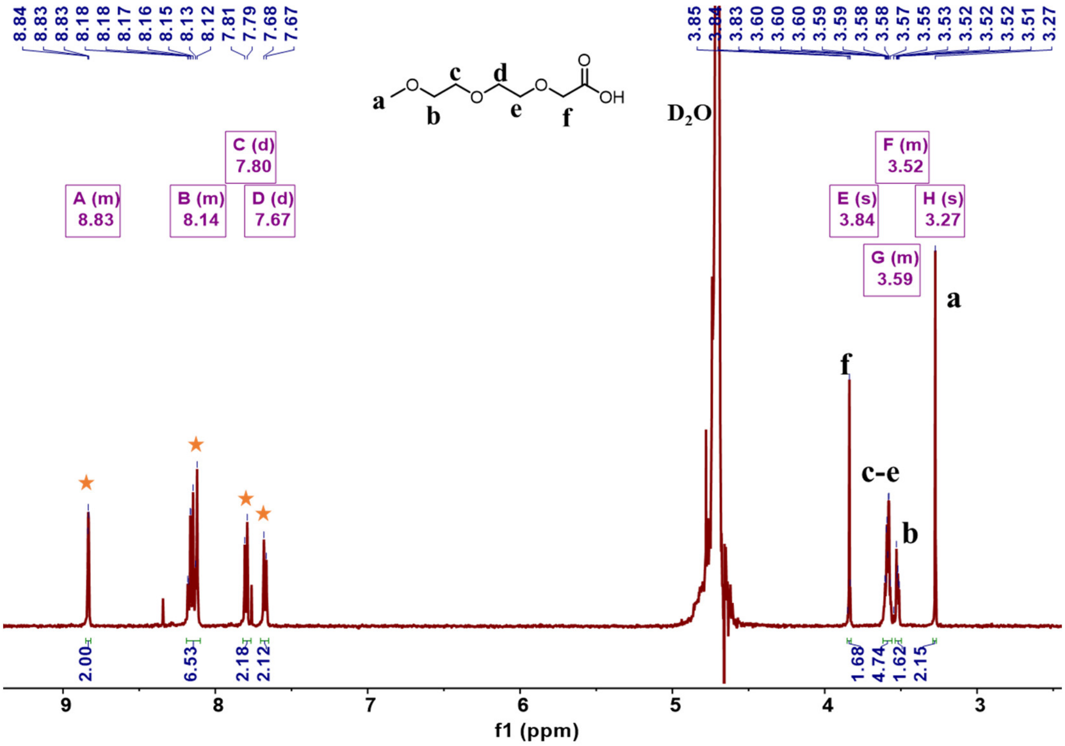

Figure S43. ^{1}H-NMR analysis after digestion of Hf-TPY-MOL-2-(2-(2-methoxyethoxy) ethoxy)

acetic acid (CAS: 16024-58-1). ★ : ^{1}H-NMR peaks for H_3TPY, a-f indicate peaks of corresponding Hs on the molecule.

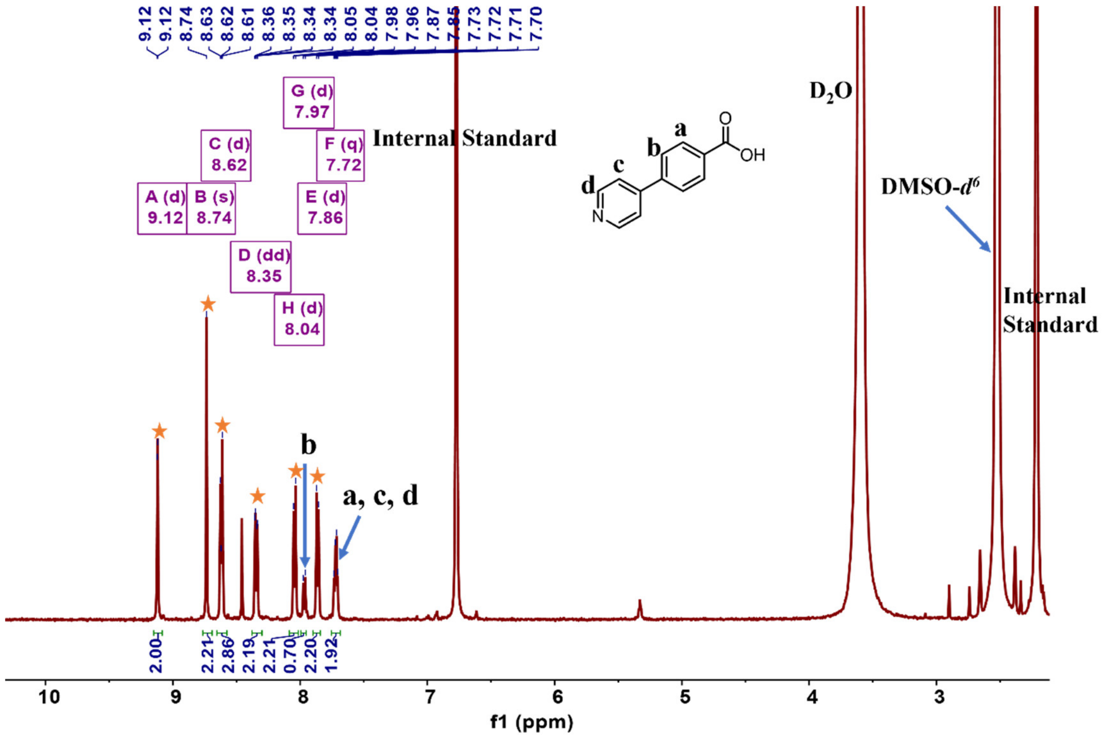

Figure S44. ^{1}H-NMR analysis after digestion of Hf-TPY-MOL-4-(4-pyridyl)benzoic acid (CAS: 4385-76-6). ★ : ^{1}H-NMR peaks for H_3TPY, a-d indicate peaks of corresponding Hs on the molecule.

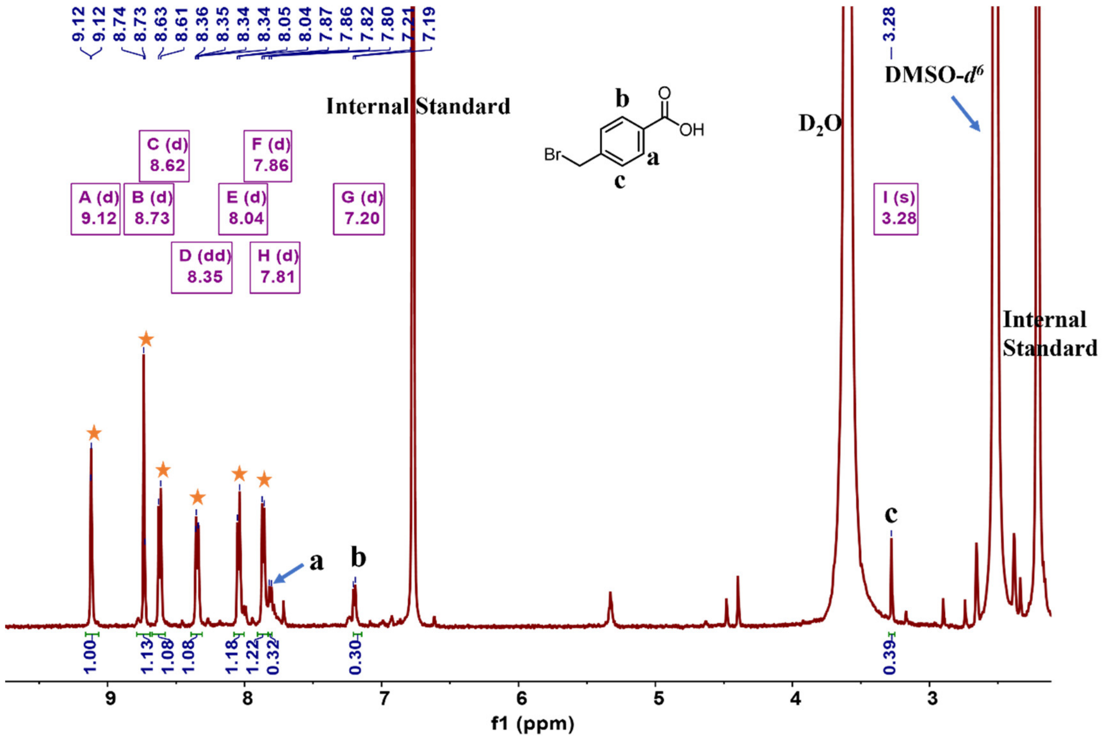

Figure S45. ^{1}H-NMR analysis after digestion of Hf-TPY-MOL-4-bromomethylbenzoic acid (CAS: 6232-88-8). ★: ^{1}H-NMR peaks for H_3TPY, a-c indicate peaks of corresponding Hs on the molecule.

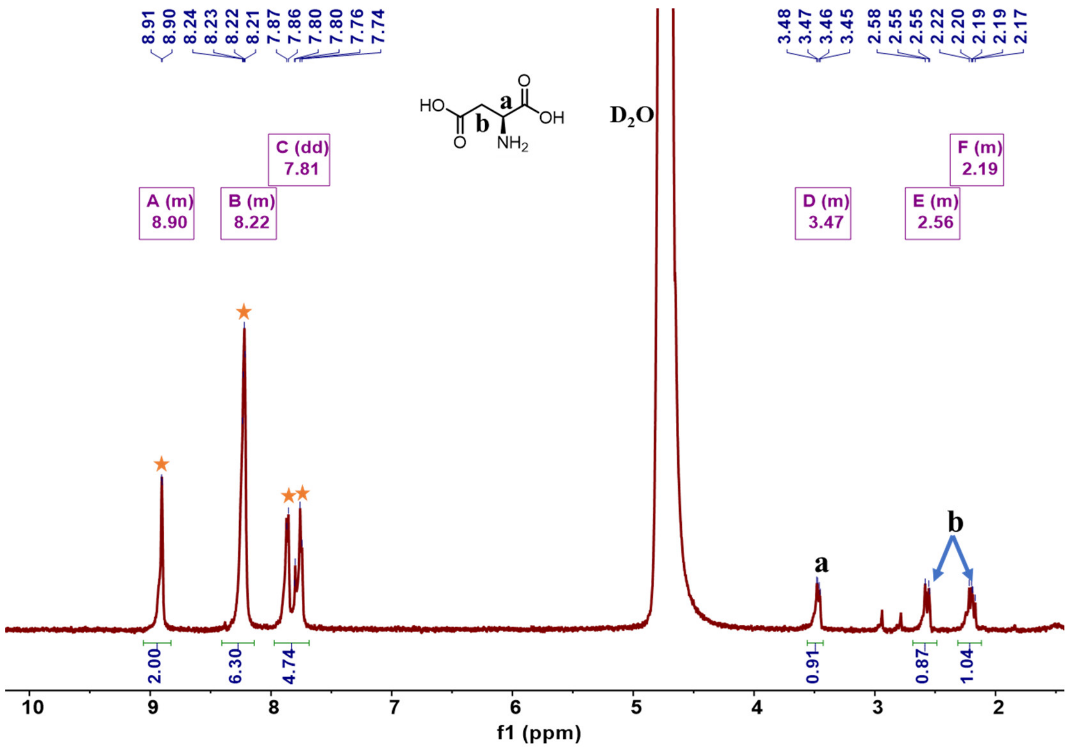

Figure S46. ^{1}H-NMR analysis after digestion of Hf-TPY-MOL-L-aspartic acid (CAS: 56-84-8). ★: ^{1}H-NMR peaks for H_3TPY, a-b indicate peaks of corresponding Hs on the molecule.

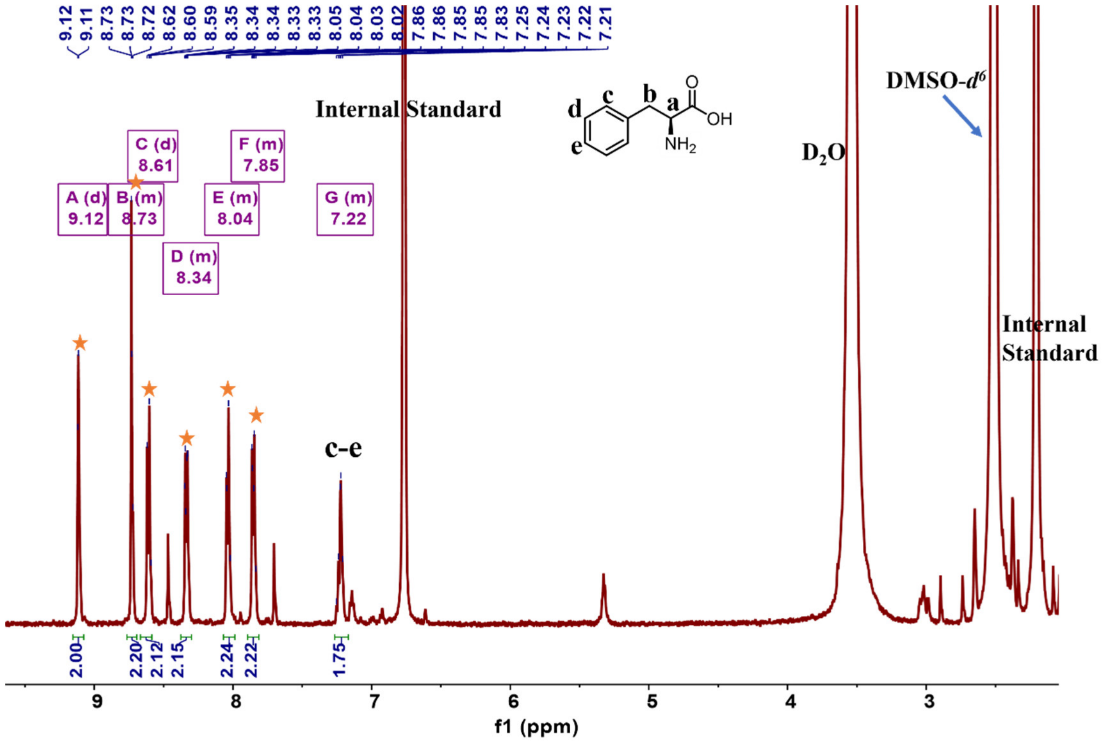

Figure S47. ^{1}H-NMR analysis after digestion of Hf-TPY-MOL-L-phenylalanine (CAS: 63-91-2). ★ : ^{1}H-NMR peaks for H_3TPY, a-d indicate peaks of corresponding Hs on the molecule.

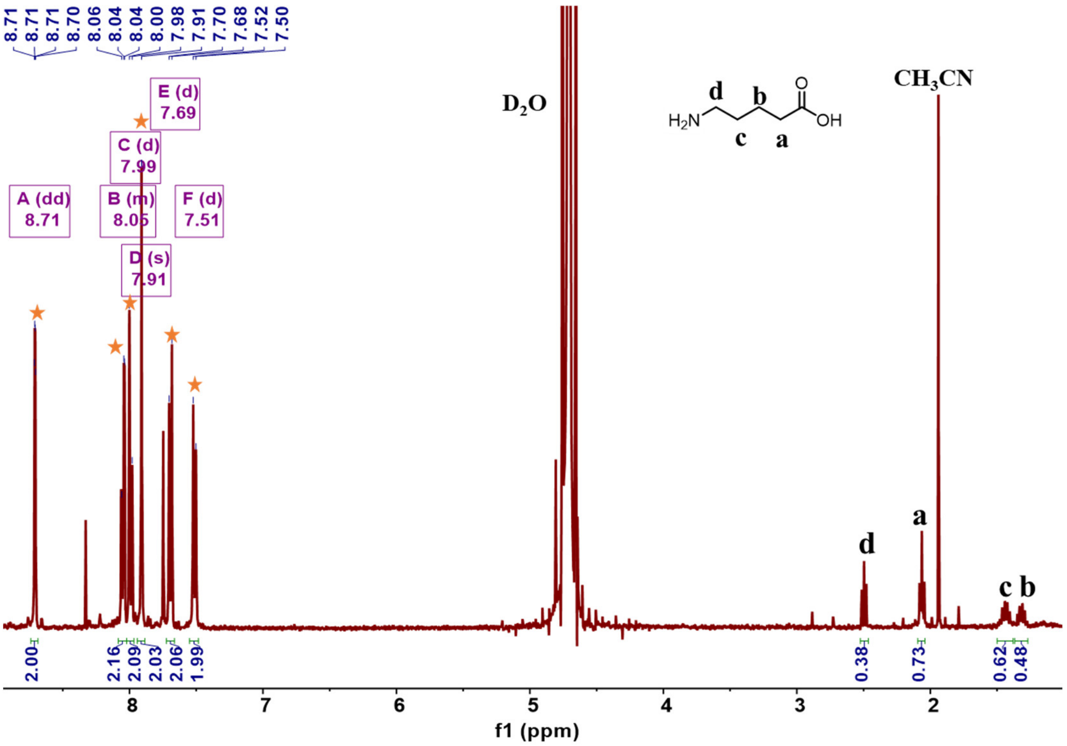

Figure S48. ^{1}H-NMR analysis after digestion of Hf-TPY-MOL-5-aminovaleric acid (CAS: 660-88-8). ★ : ^{1}H-NMR peaks for H_3TPY, a-d indicate peaks of corresponding Hs on the molecule.

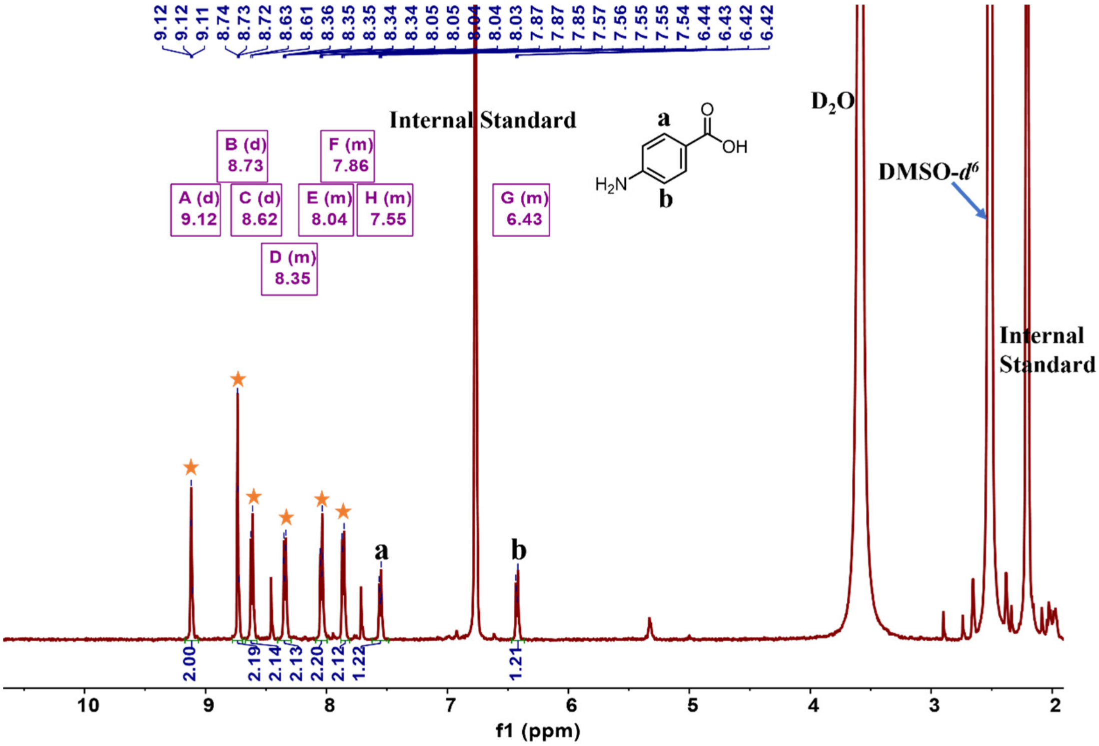

Figure S49. ^{1}H-NMR analysis after digestion of Hf-TPY-MOL-4-aminobenzoic acid (CAS:

150-13-0). ★ : ^{1}H-NMR peaks for H_3TPY, a-b indicate peaks of corresponding Hs on the molecule.

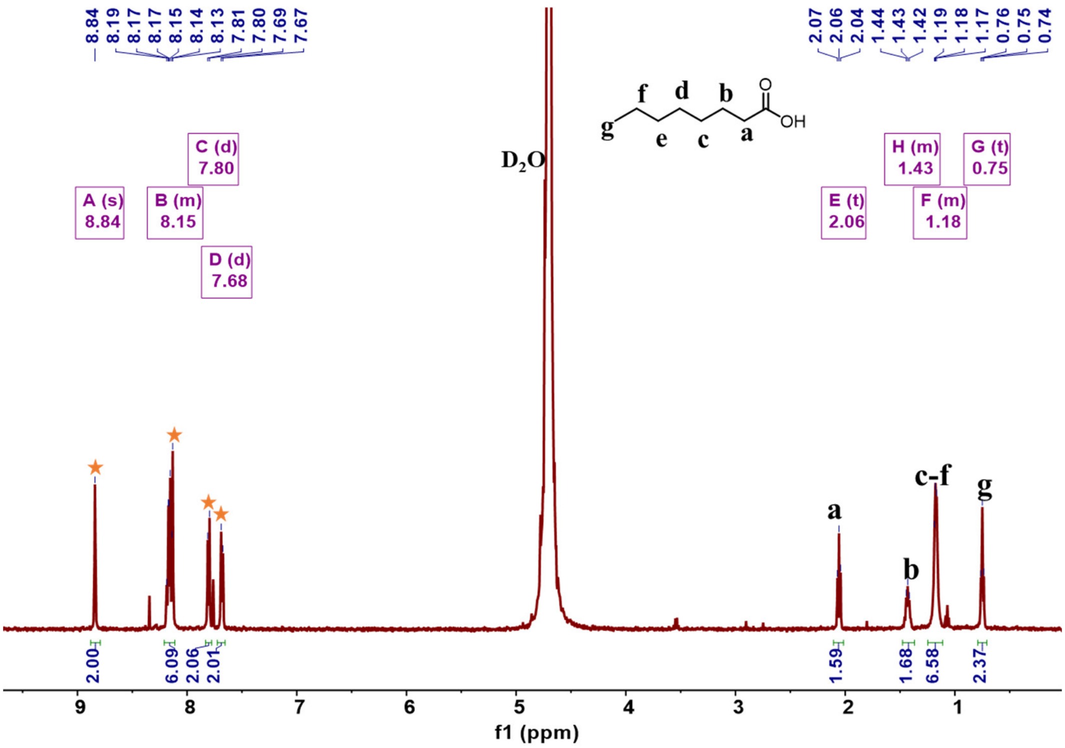

Figure S50. ^{1}H-NMR analysis after digestion of Hf-TPY-MOL-octanoic acid (CAS: 124-07-2). ★ : ^{1}H-NMR peaks for H_3TPY, a-g indicate peaks of corresponding Hs on the molecule.

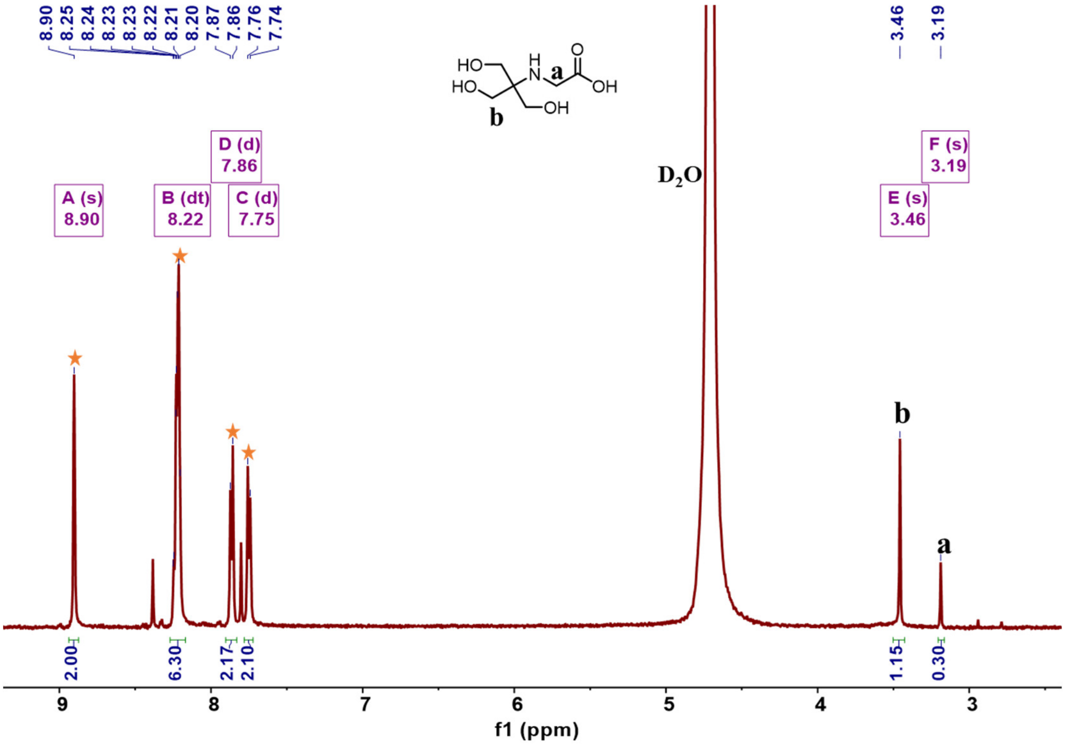

Figure S51. ^{1}H-NMR analysis after digestion of Hf-TPY-MOL-tricine (CAS: 5704-04-1). ★ : ^{1}H-NMR peaks for H_3TPY, a-b indicate peaks of corresponding Hs on the molecule.

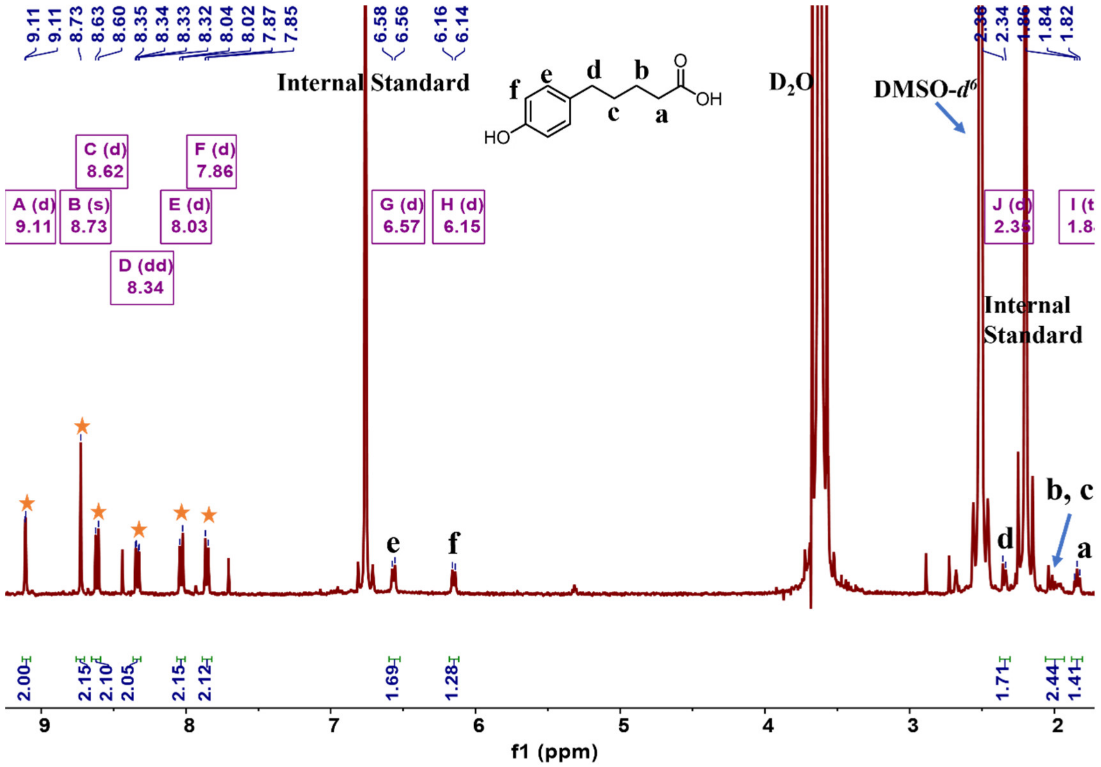

Figure S52. ^{1}H-NMR analysis after digestion of Hf-TPY-MOL-5-(4-hydroxyphenyl) pentanoic acid (CAS: 4654-08-4). ★ : ^{1}H-NMR peaks for H_3TPY, a-f indicate peaks of corresponding Hs on the molecule.

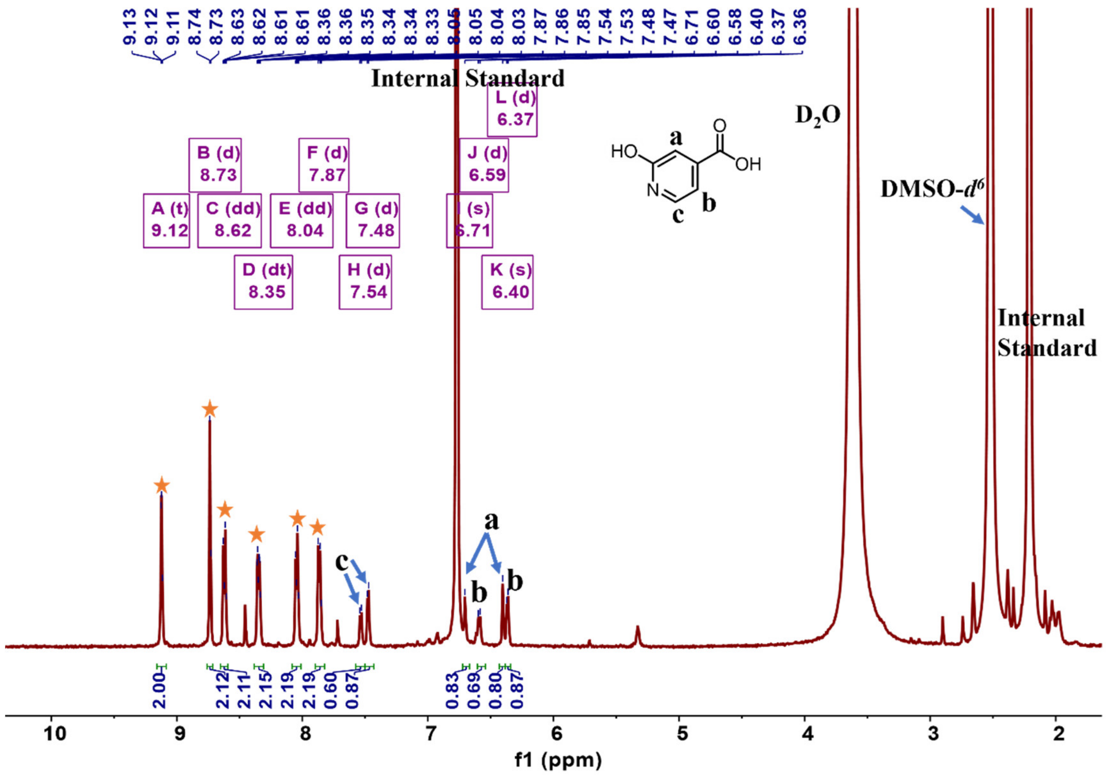

Figure S53. ^{1}H-NMR analysis after digestion of Hf-TPY-MOL-2-hydroxyisonicotinic acid (CAS: 22282-72-0). ★ : ^{1}H-NMR peaks for H_3TPY, a-c indicate peaks of corresponding Hs on the molecule.

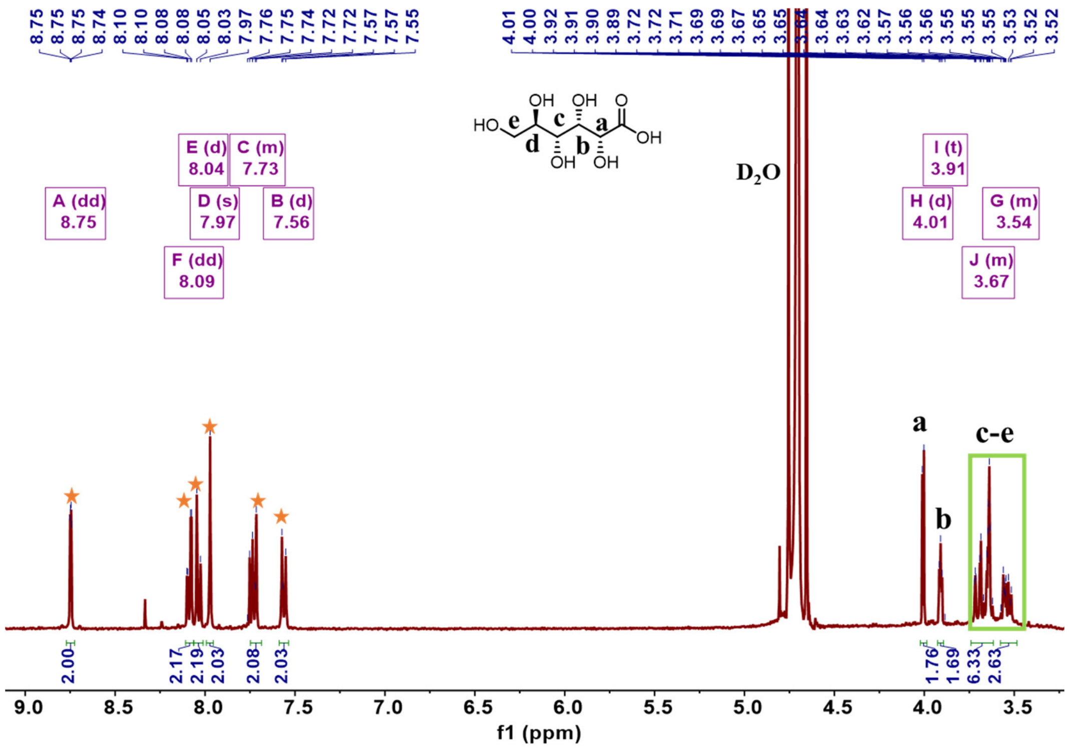

Figure S54. ^{1}H-NMR analysis after digestion of Hf-TPY-MOL-gluconic acid (CAS: 526-95-4). ★: ^{1}H-NMR peaks for H_3TPY, a-e indicate peaks of corresponding Hs on the molecule.

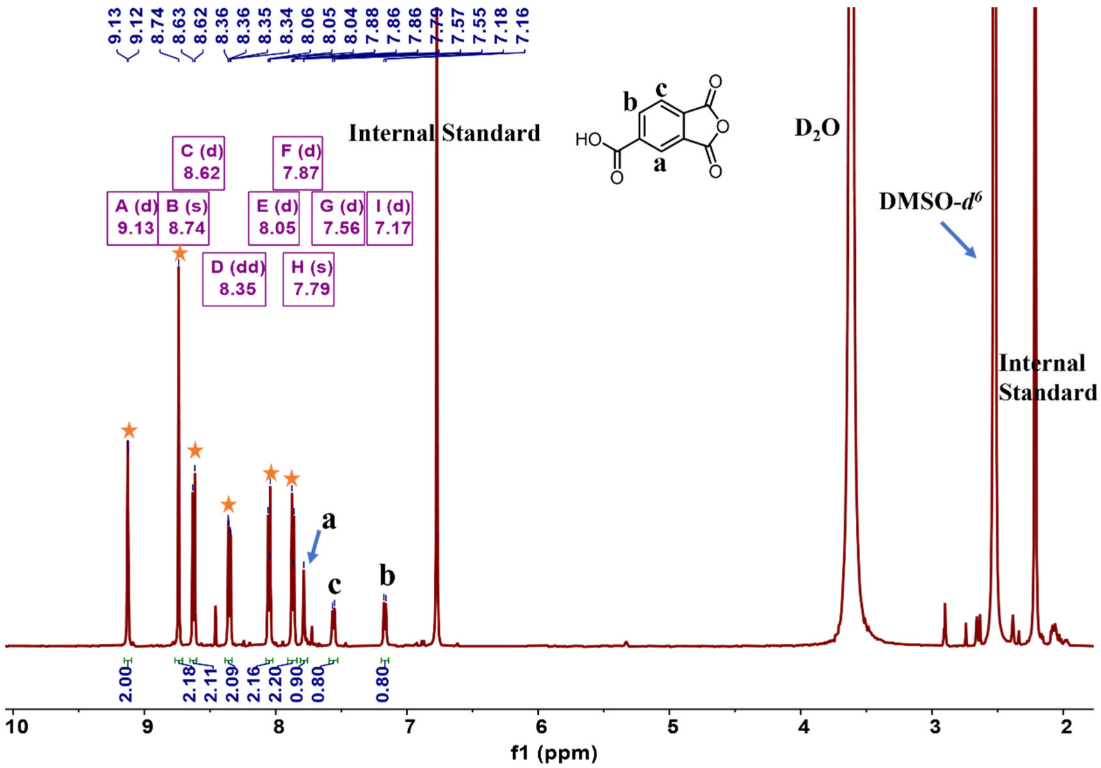

Figure S55. ^{1}H-NMR analysis after digestion of Hf-TPY-MOL-trimellitic anhydride (CAS: 552-30-7). ★: ^{1}H-NMR peaks for H_3TPY, a-c indicate peaks of corresponding Hs on the molecule.

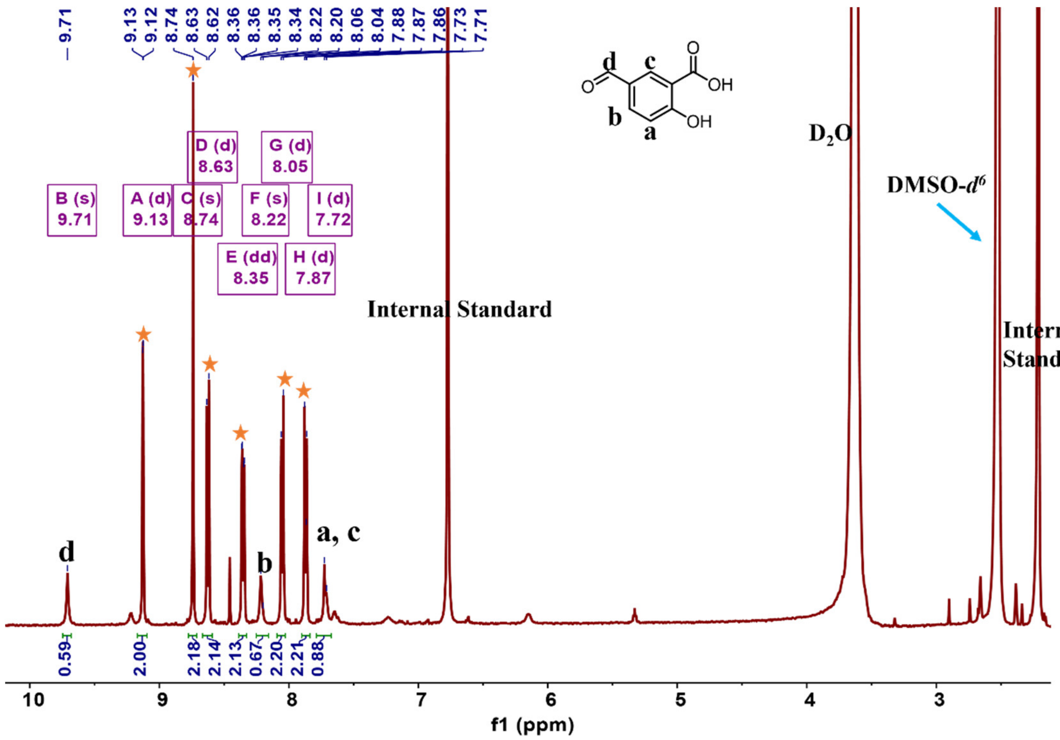

Figure S56. ^{1}H-NMR analysis after digestion of Hf-TPY-MOL-5-formylsalicylic acid (CAS: 616-76-2). ★ : ^{1}H-NMR peaks for H_3TPY, a-d indicate peaks of corresponding Hs on the molecule.

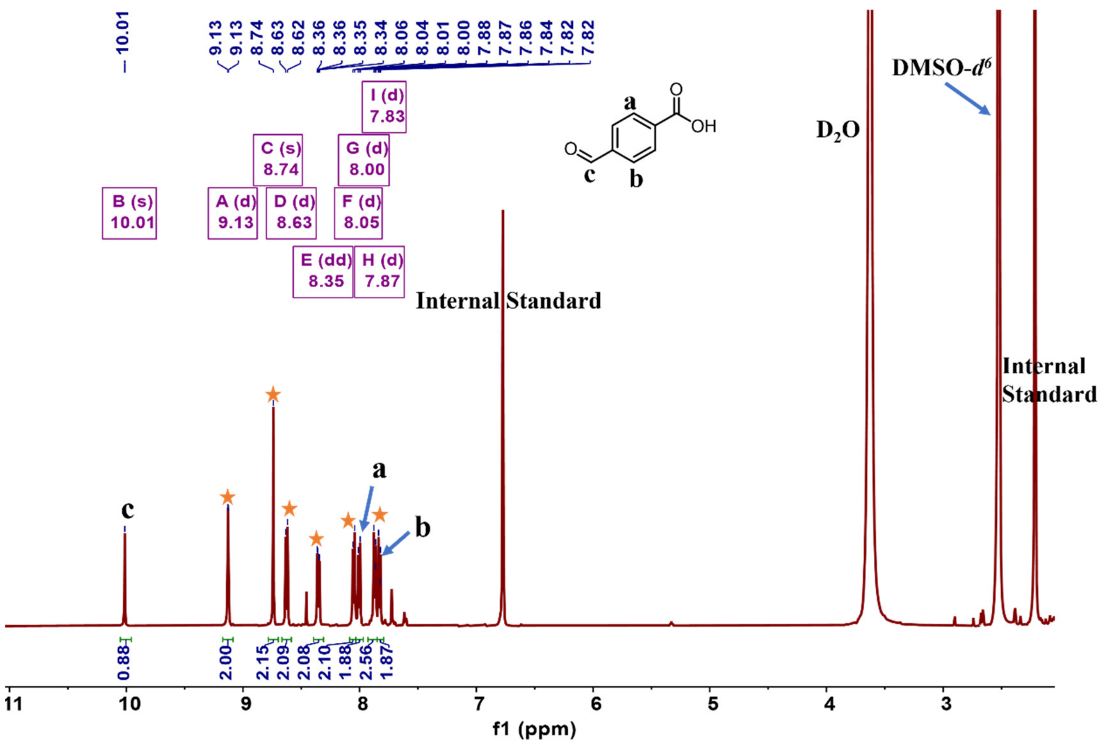

Figure S57. ^{1}H-NMR analysis after digestion of Hf-TPY-MOL-4-formylbenzoic acid (CAS: 619-66-9). ★: ^{1}H-NMR peaks for H_3TPY, a-c indicate peaks of corresponding Hs on the molecule.

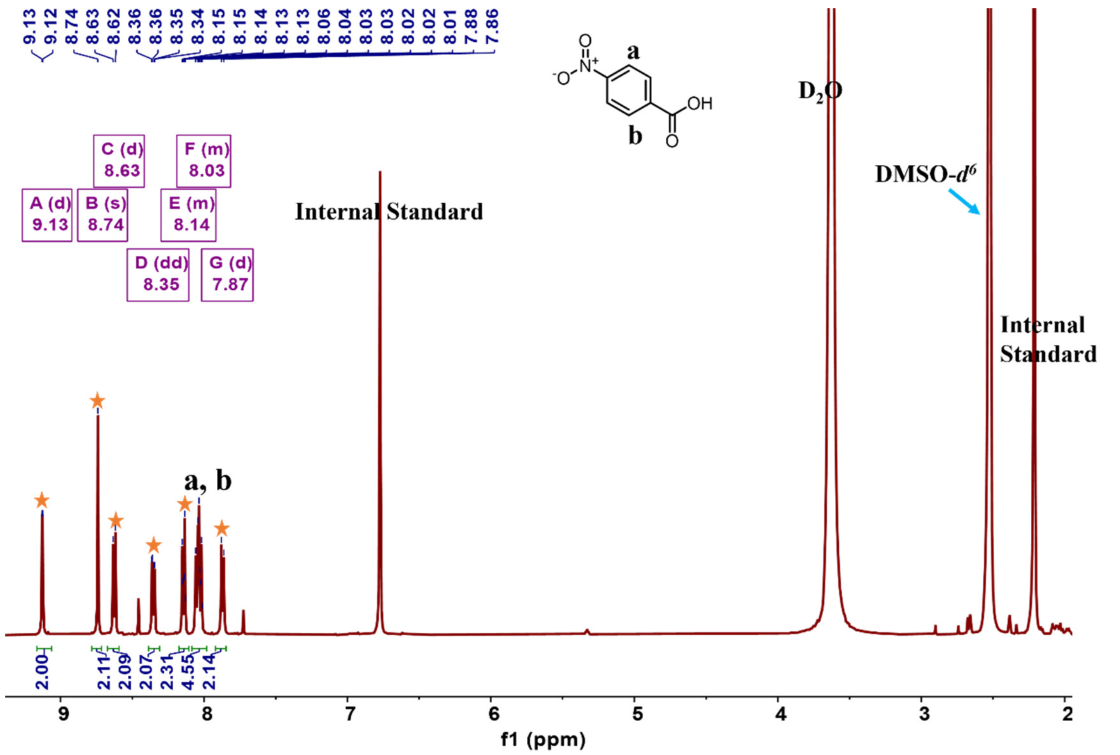

Figure S58. ^{1}H-NMR analysis after digestion of Hf-TPY-MOL-4-nitrobenzoic acid(CAS: 62-23-7). ★: ^{1}H-NMR peaks for H_3TPY, a-b indicate peaks of corresponding Hs on the molecule.

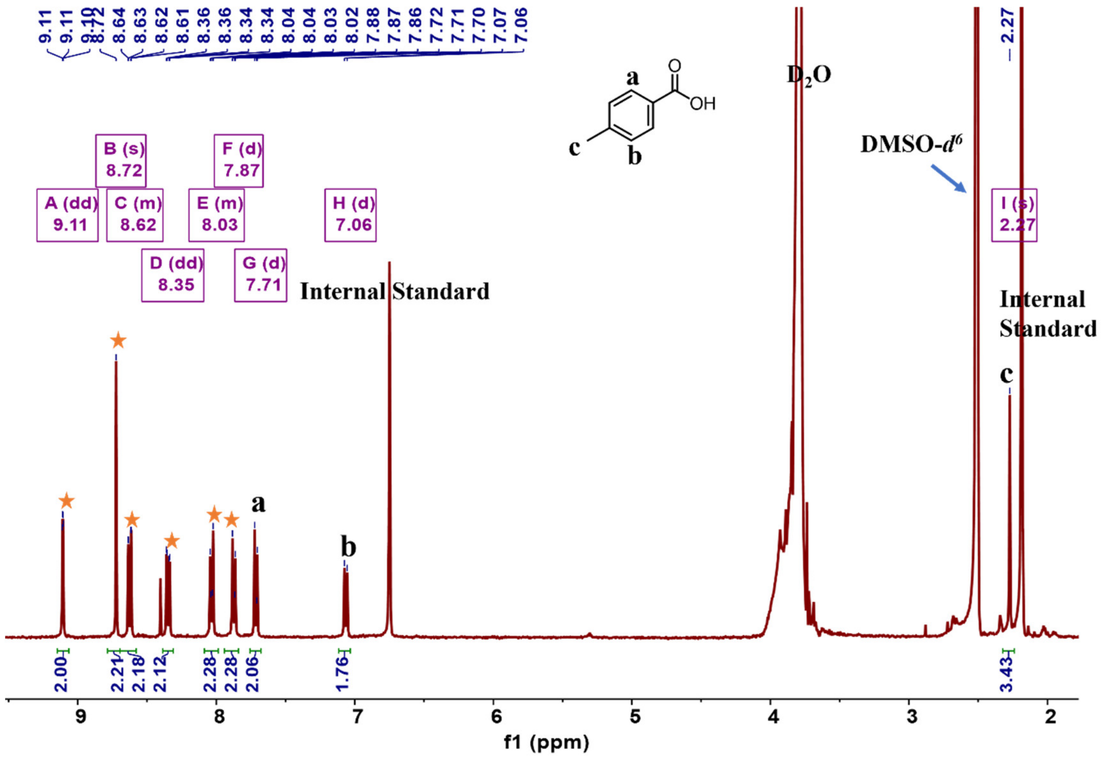

Figure S59. ^{1}H-NMR analysis after digestion of Hf-TPY-MOL-*p*-toluic acid (CAS: 99-94-5). ★: ^{1}H-NMR peaks for H_3TPY, a-b indicate peaks of corresponding Hs on the molecule.

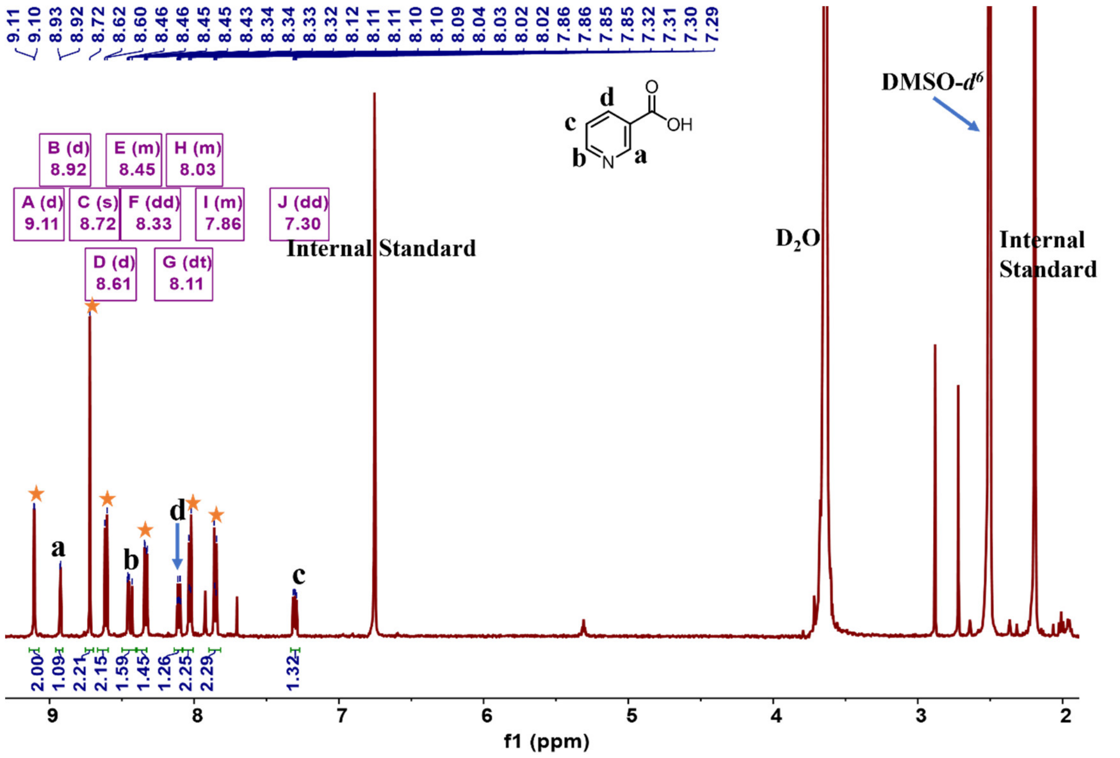

Figure S60. ^{1}H-NMR analysis after digestion of Hf-TPY-MOL-nicotinic acid (CAS: 59-67-6). ★: ^{1}H-NMR peaks for H_3TPY, a-d indicate peaks of corresponding Hs on the molecule.

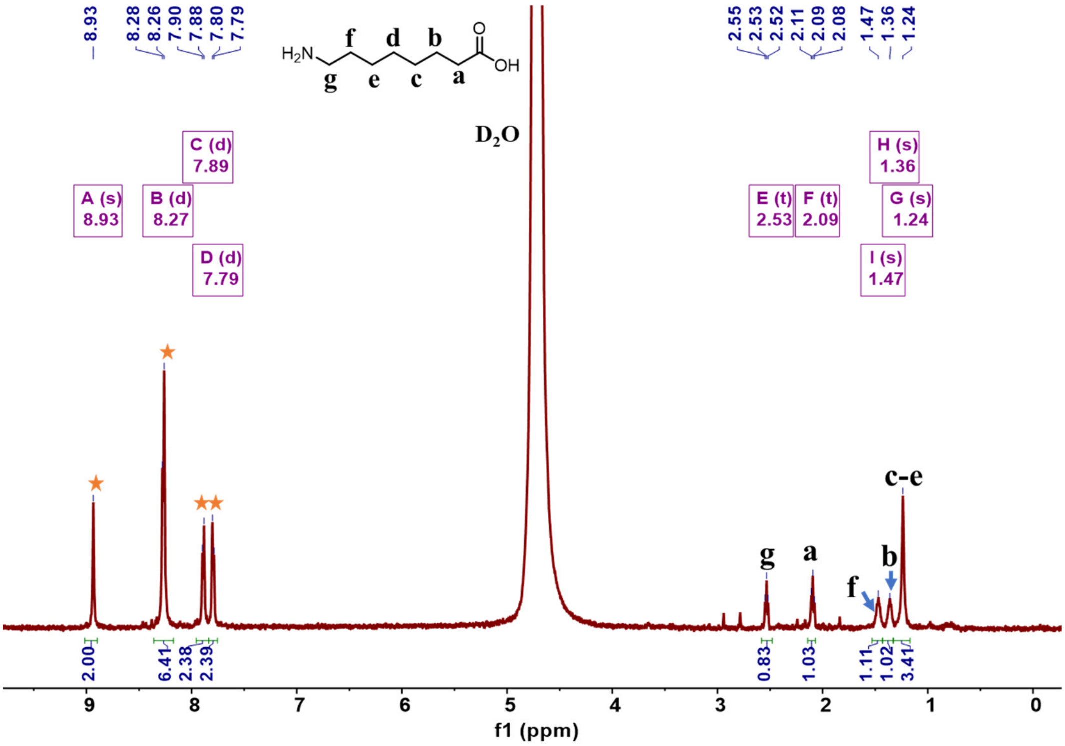

Figure S61. ^{1}H-NMR analysis after digestion of Hf-TPY-MOL-8-aminooctanoic acid (CAS: 1002-57-9). ★: ^{1}H-NMR peaks for H_3TPY, a-g indicate peaks of corresponding Hs on the molecule.

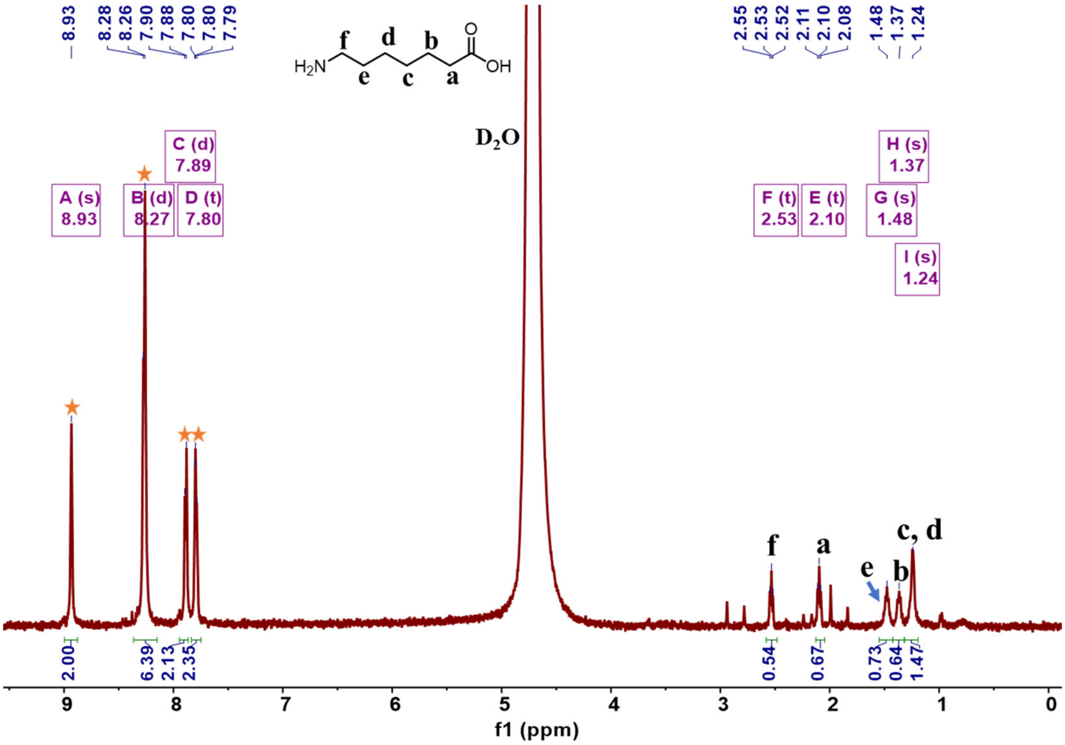

Figure S62. ^{1}H-NMR analysis after digestion of Hf-TPY-MOL-7-aminoheptanoic acid (CAS: 929-17-9). ★: ^{1}H-NMR peaks for H_3TPY, a-f indicate peaks of corresponding Hs on the molecule.

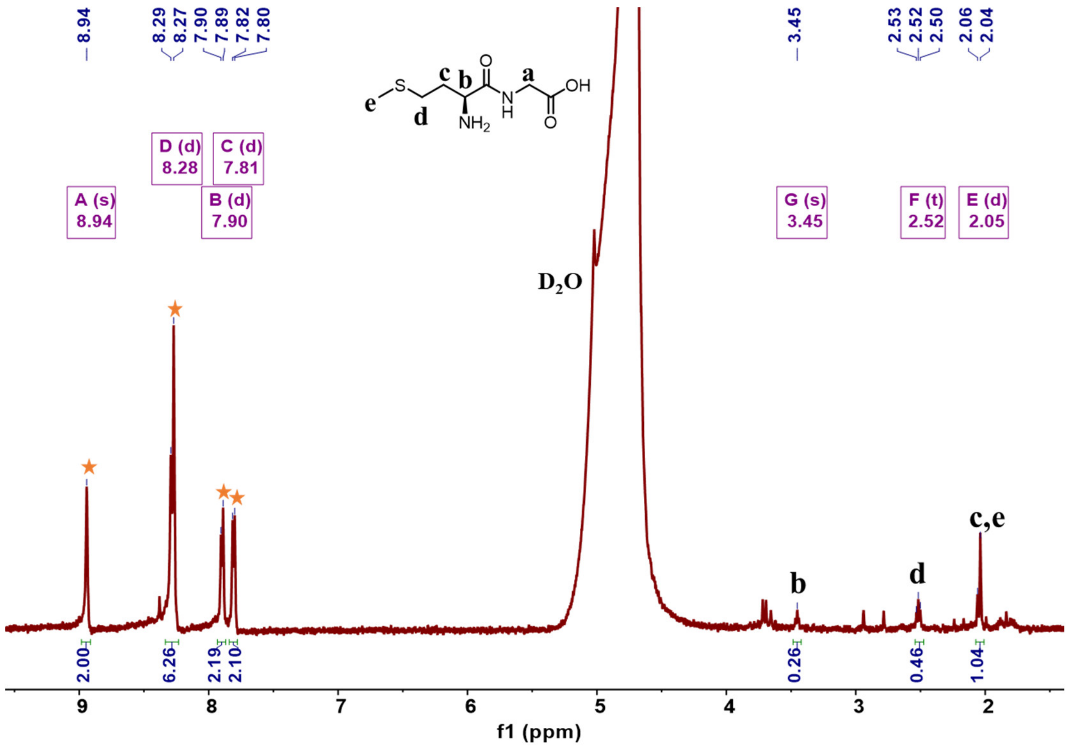

Figure S63. ¹H-NMR analysis after digestion of Hf-TPY-MOL-L-methionylglycine (CAS: 14486-03-4). ★: ¹H-NMR peaks for H_3TPY, a-e indicate peaks of corresponding Hs on the molecule.

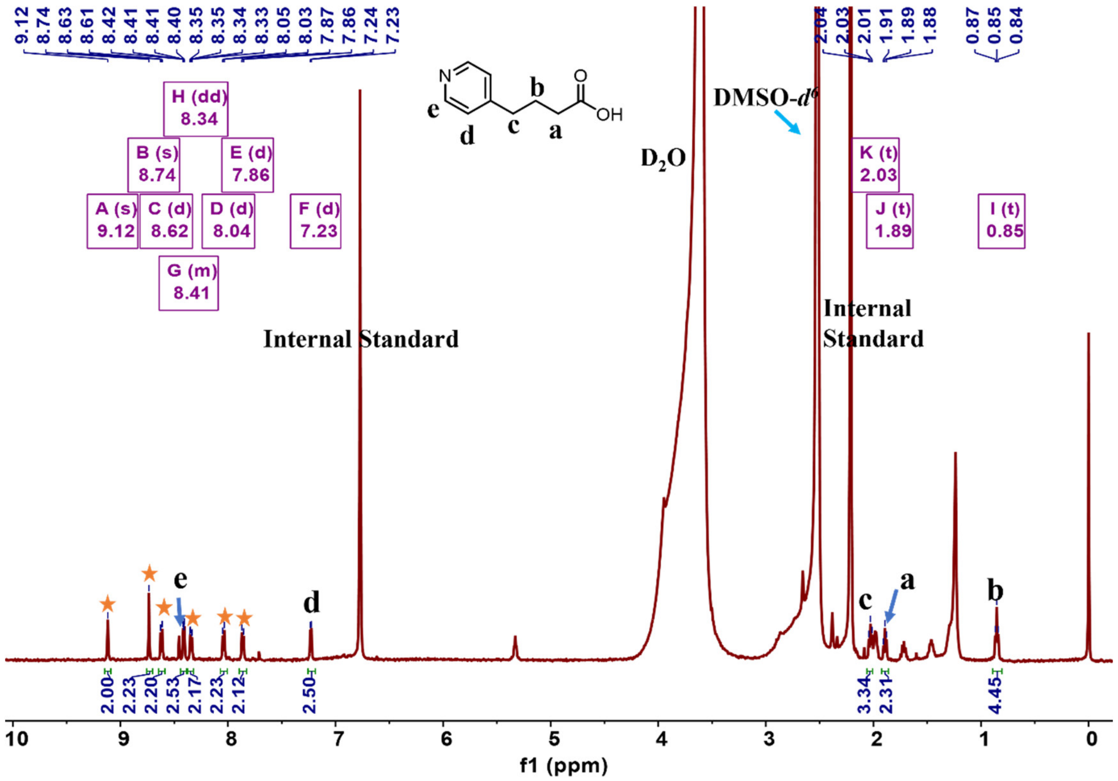

Figure S64. ¹H-NMR analysis after digestion of Hf-TPY-MOL-4-pyridin-4-yl-butyric acid (CAS: 102878-73-9). ★: ¹H-NMR peaks for H_3TPY, a-e indicate peaks of corresponding Hs on the molecule.

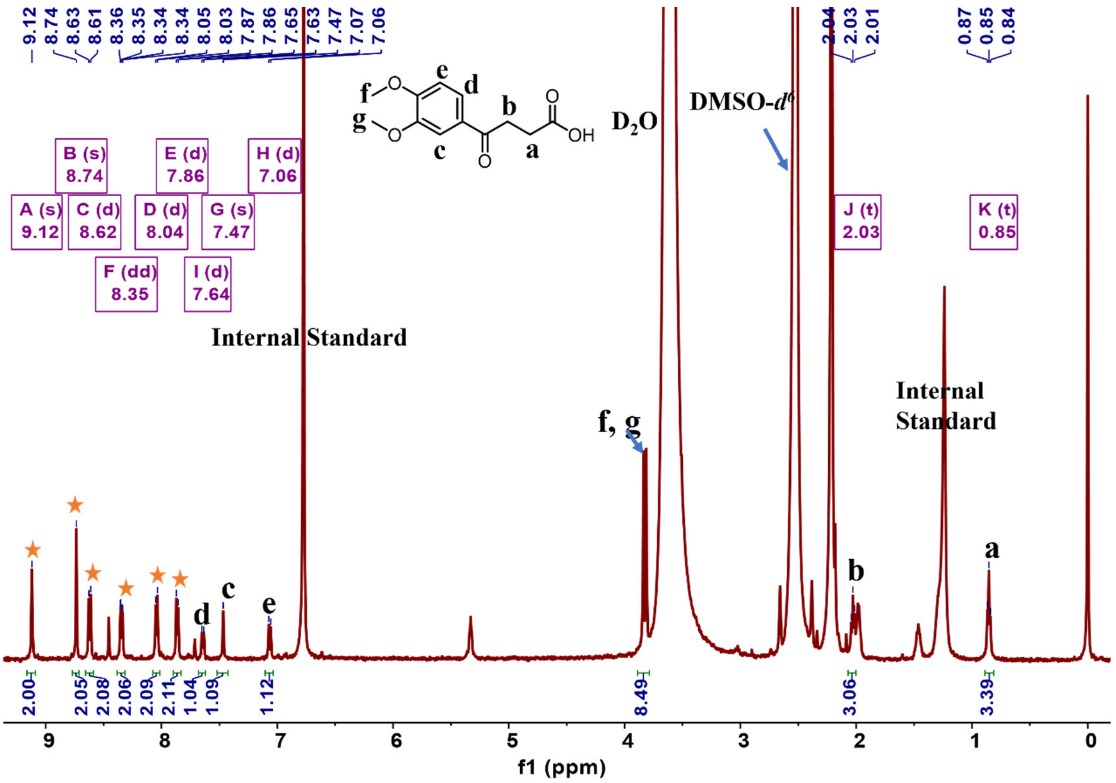

Figure S65. ^{1}**H-NMR analysis after digestion of Hf-TPY-MOL-3-(3,4-dimethoxybenzoyl) propionic acid (CAS: 5333-34-64).** ★: ^{1}H-NMR peaks for H_3TPY, a-g indicate peaks of corresponding Hs on the molecule.

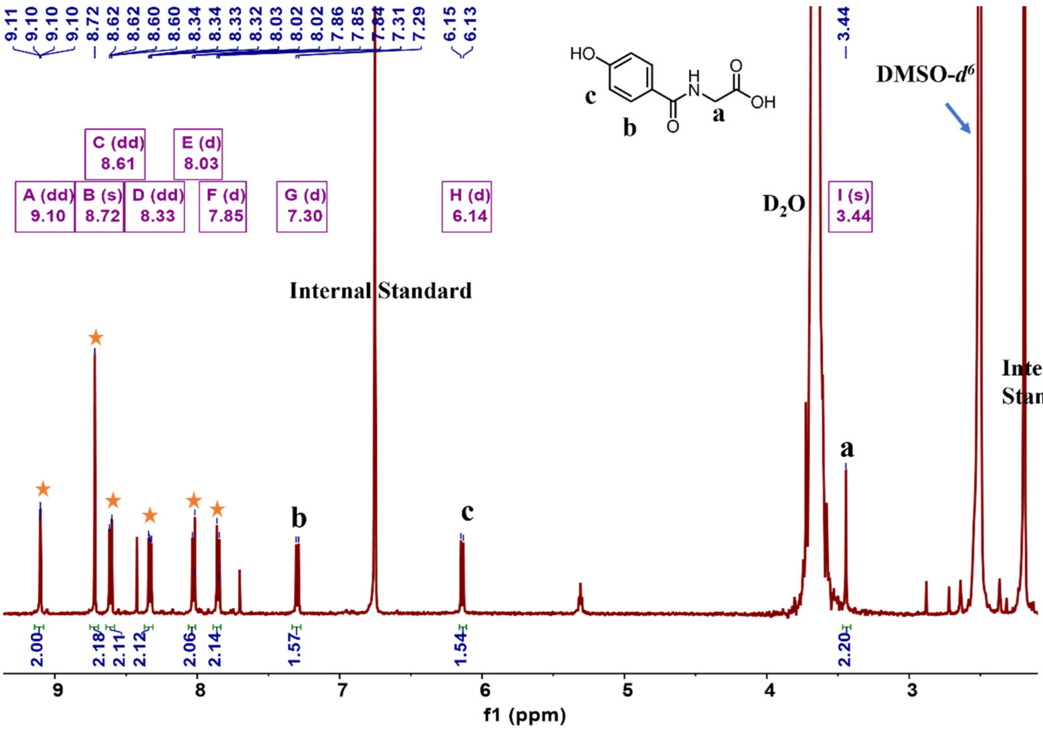

Figure S66. ^{1}**H-NMR analysis after digestion of Hf-TPY-MOL-2-[(4-hydroxyphenyl) carbonyl**

amino] ethanoic acid (CAS: 2482-25-9). ★ : ^{1}H-NMR peaks for H_3TPY, a-c indicate peaks of corresponding Hs on the molecule.

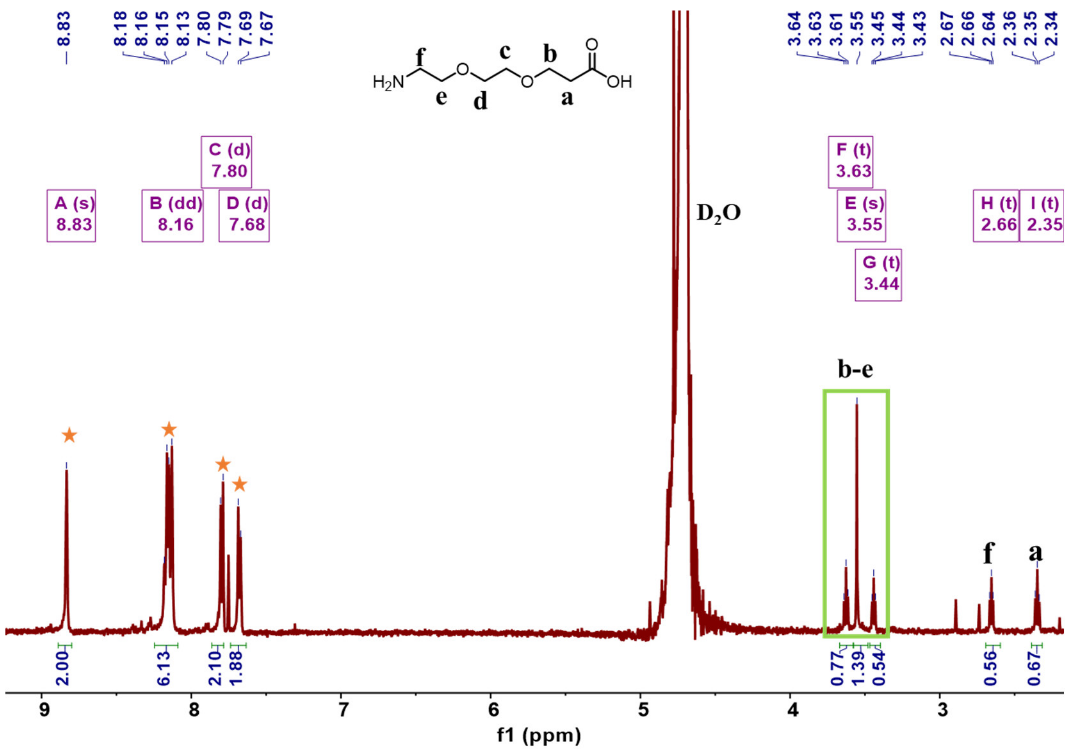

Figure S67. ^{1}H-NMR analysis after digestion of -amino-PEG2-acid (CAS: 791028-27-8). ★ : ^{1}H-NMR peaks for H_3TPY, a-f indicate peaks of corresponding Hs on the molecule.

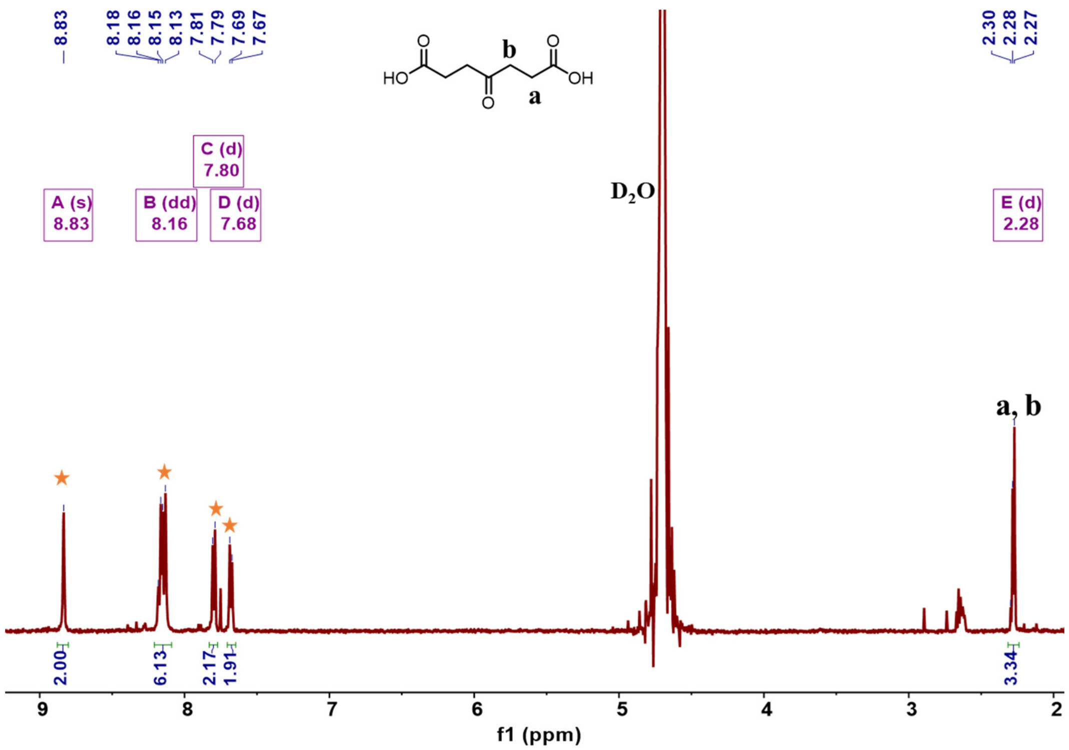

Figure S68. ^{1}H-NMR analysis after digestion of Hf-TPY-MOL-heptanedioic acid (CAS: 502-

50-1). ★ : ^{1}H-NMR peaks for H_3TPY, a-b indicate peaks of corresponding Hs on the molecule.

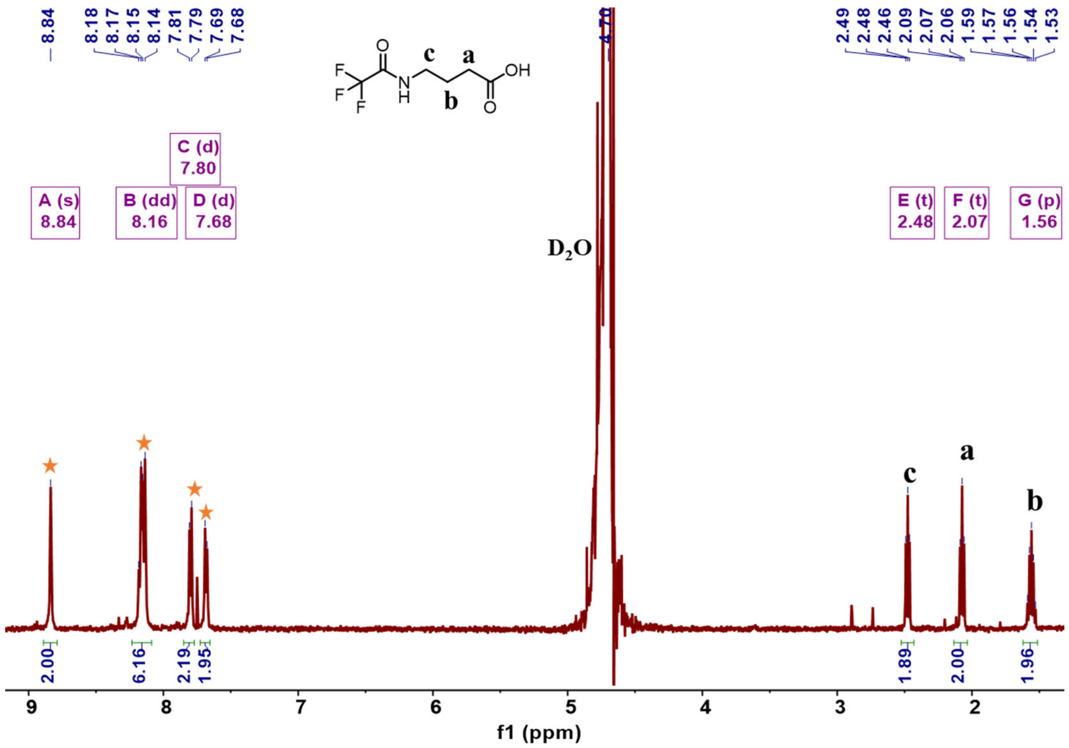

Figure S69. ^{1}H-NMR analysis after digestion of Hf-TPY-MOL-4-(2,2,2-trifluoroacetamido) butanoic acid (CAS： 50632-83-2). ★ : ^{1}H-NMR peaks for H_3TPY, a-c indicate peaks of corresponding Hs on the molecule.

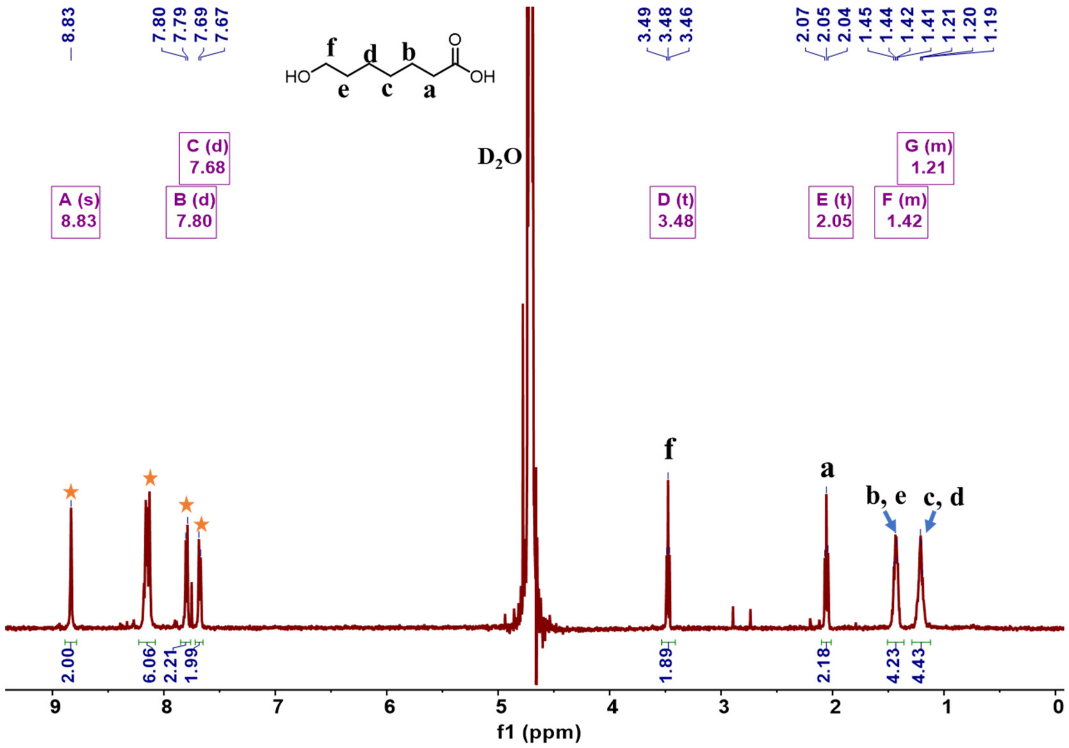

Figure S70. ^{1}H-NMR analysis after digestion of Hf-TPY-MOL-7-hydroxyheptanoic acid (CAS:

3710-42-7). ★ : ^{1}H-NMR peaks for H_3TPY, a-f indicate peaks of corresponding Hs on the molecule.

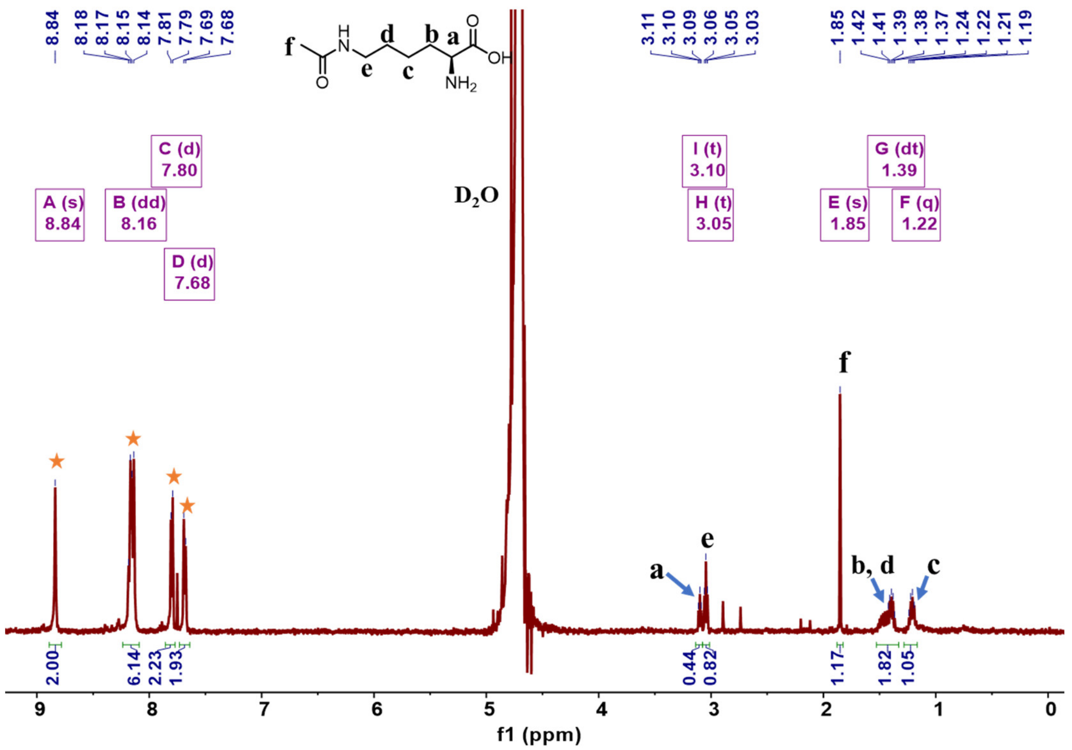

Figure S71. ^{1}H-NMR analysis after digestion of Hf-TPY-MOL-N-epsilon-acetyl-L-lysine (CAS: 692-04-6). ★ : ^{1}H-NMR peaks for H_3TPY, a-f indicate peaks of corresponding Hs on the molecule.

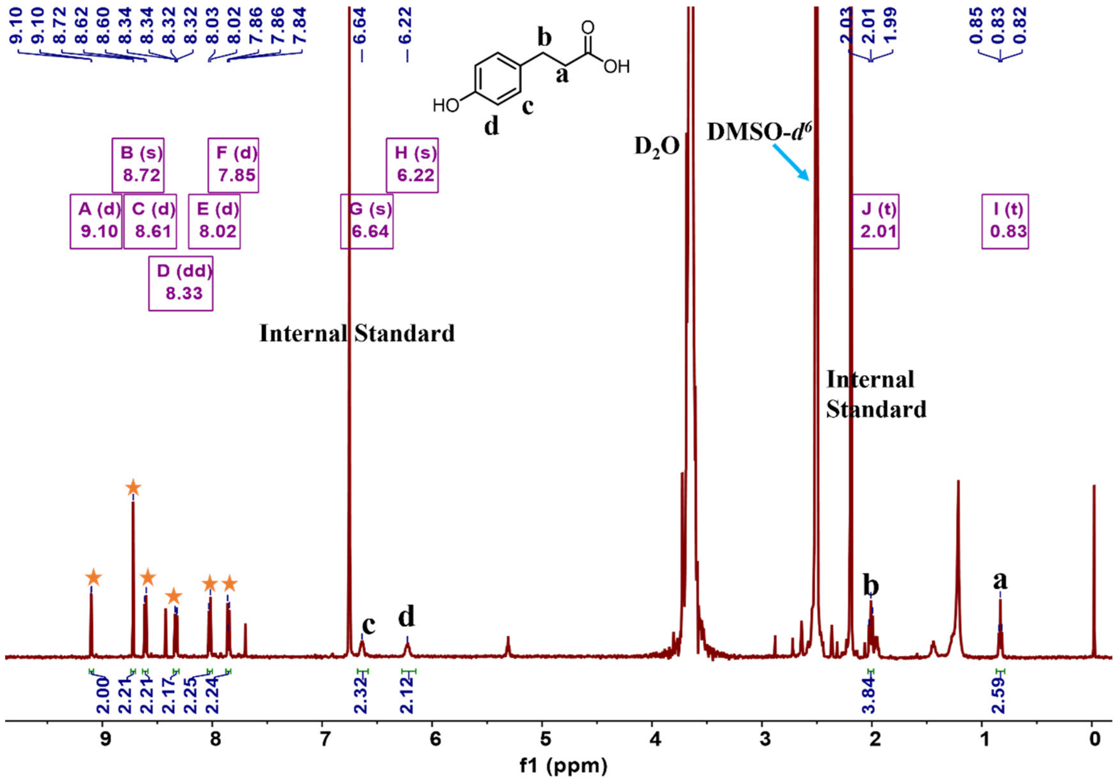

Figure S72. 1H-NMR analysis after digestion of Hf-TPY-MOL-3-(4-hydroxyphenyl) propionic

acid (CAS: 501-97-3). ★ : 1H-NMR peaks for H3TPY, a-d indicate peaks of corresponding Hs on the molecule.

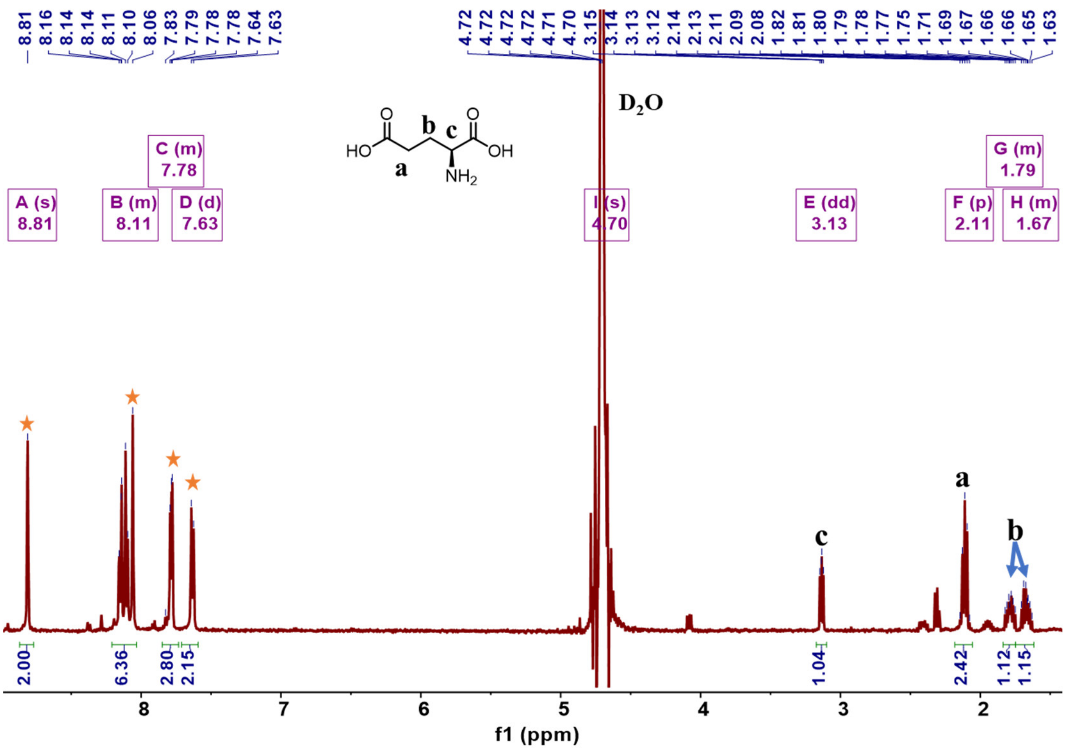

Figure S73. ^{1}H-NMR analysis after digestion of Hf-TPY-MOL-L-glutamic acid (CAS: 56-86-0). ★ : ^{1}H-NMR peaks for H_3TPY, a-c indicate peaks of corresponding Hs on the molecule.

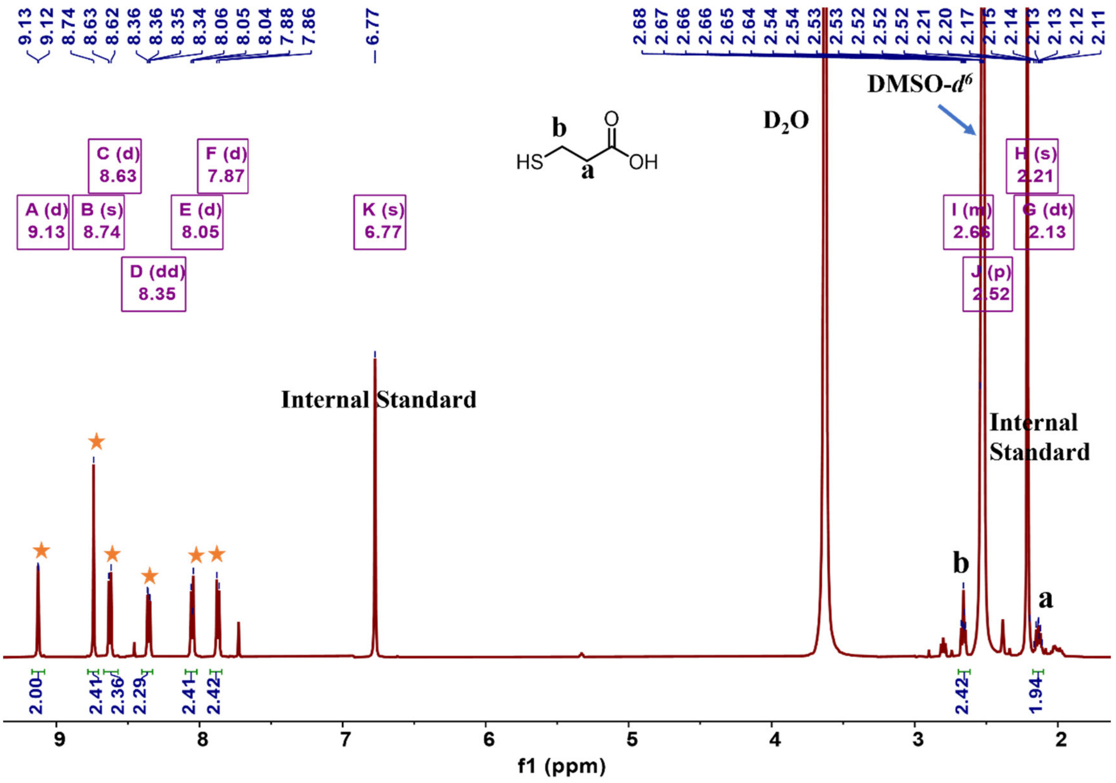

Figure S74. ^{1}H-NMR analysis after digestion of Hf-TPY-MOL-3-mercaptopropionic acid (CAS: 107-96-0). Label a-b indicate peaks of corresponding Hs on the molecule.

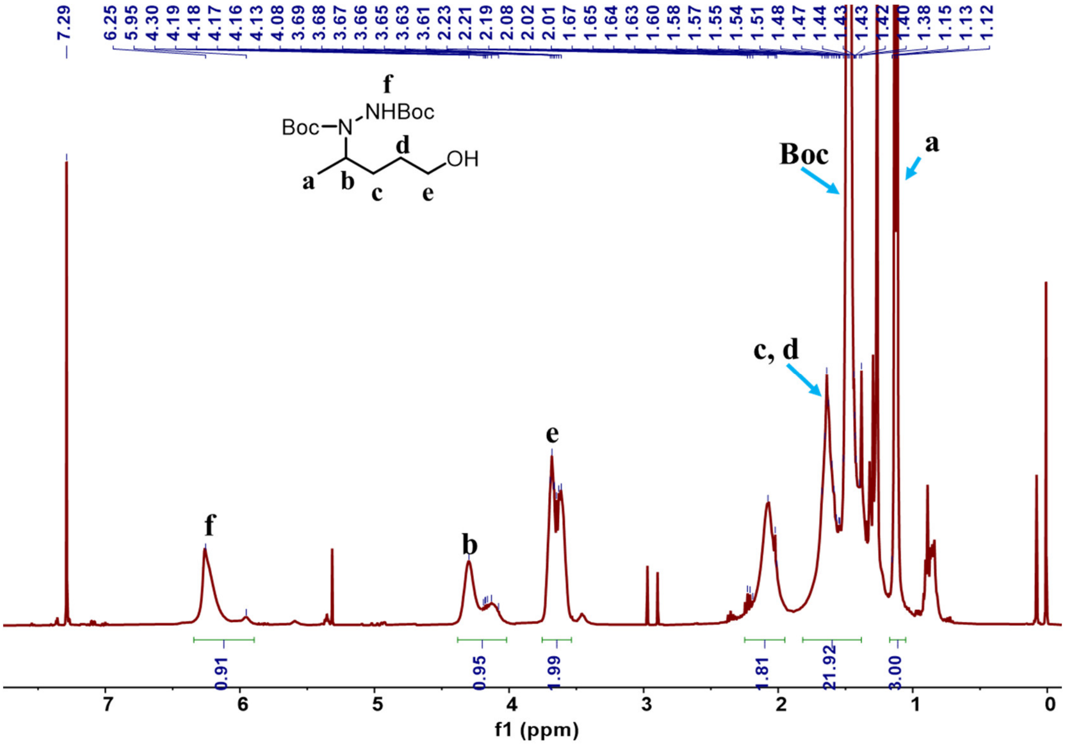

Figure S75. ^{1}H-NMR spectrum of the amination products of *n*-pentanol. Label a-f indicate peaks of corresponding Hs on the molecule.

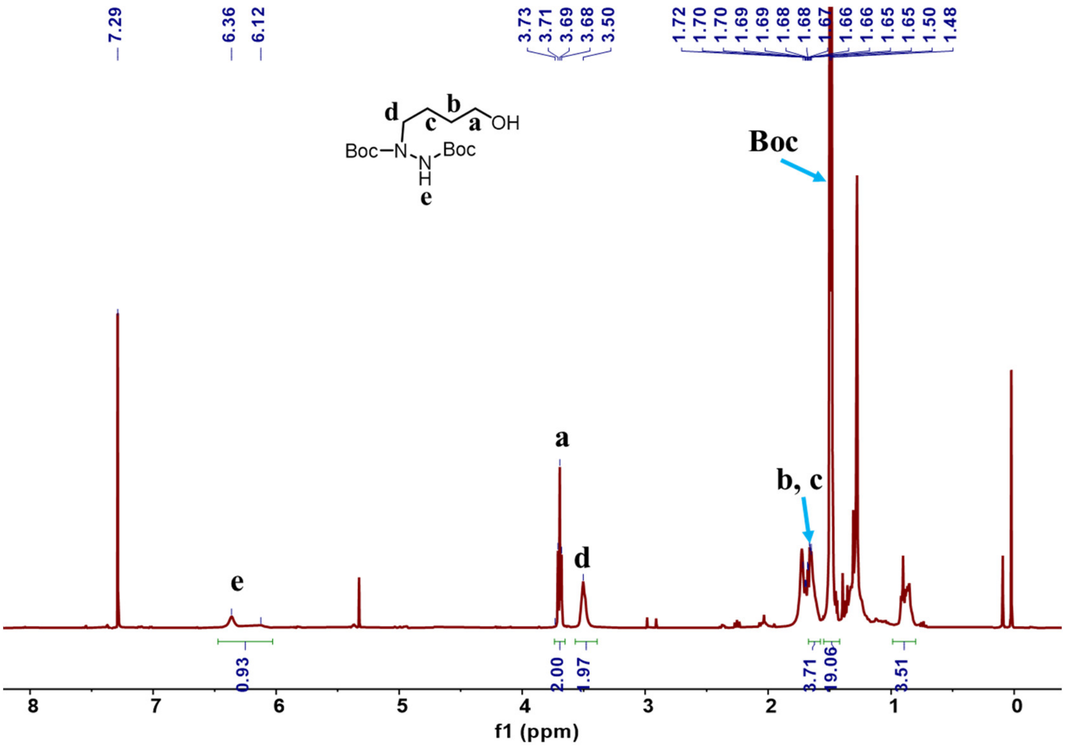

Figure S76. ^{1}H-NMR spectrum of the amination products of *n*-butanol. Label a-e indicate peaks of corresponding Hs on the molecule.

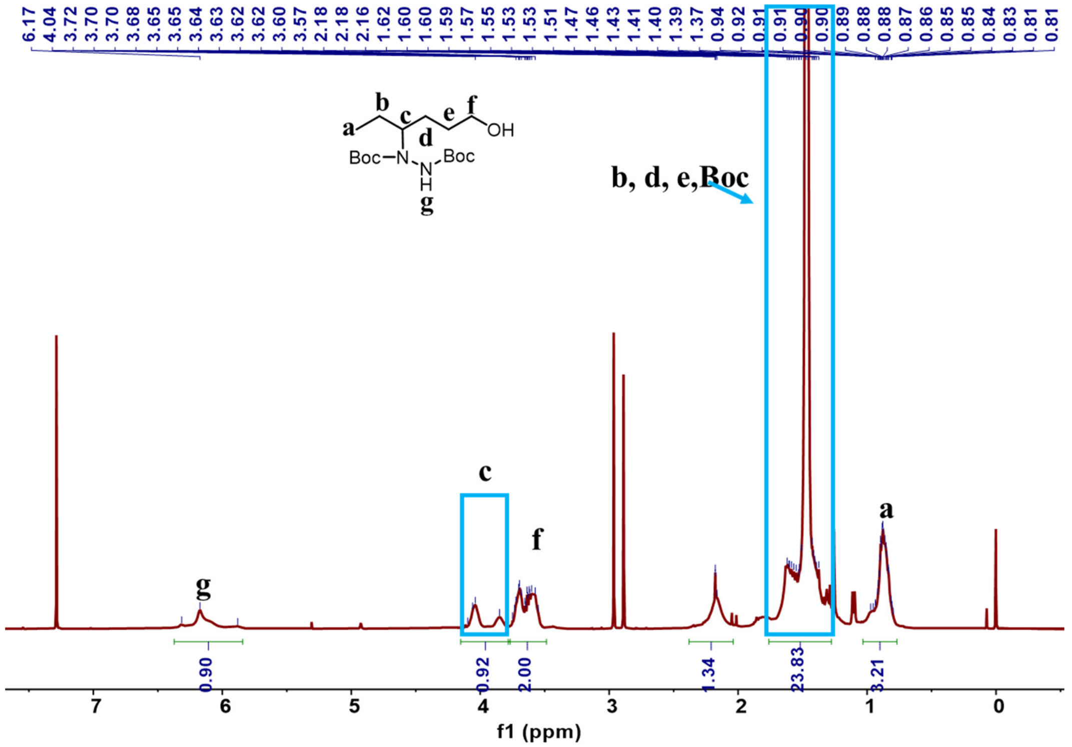

Figure S77. ^{1}H-NMR spectrum of the amination products of *n*-hexanol. Label a-e indicate peaks of corresponding Hs on the molecule.

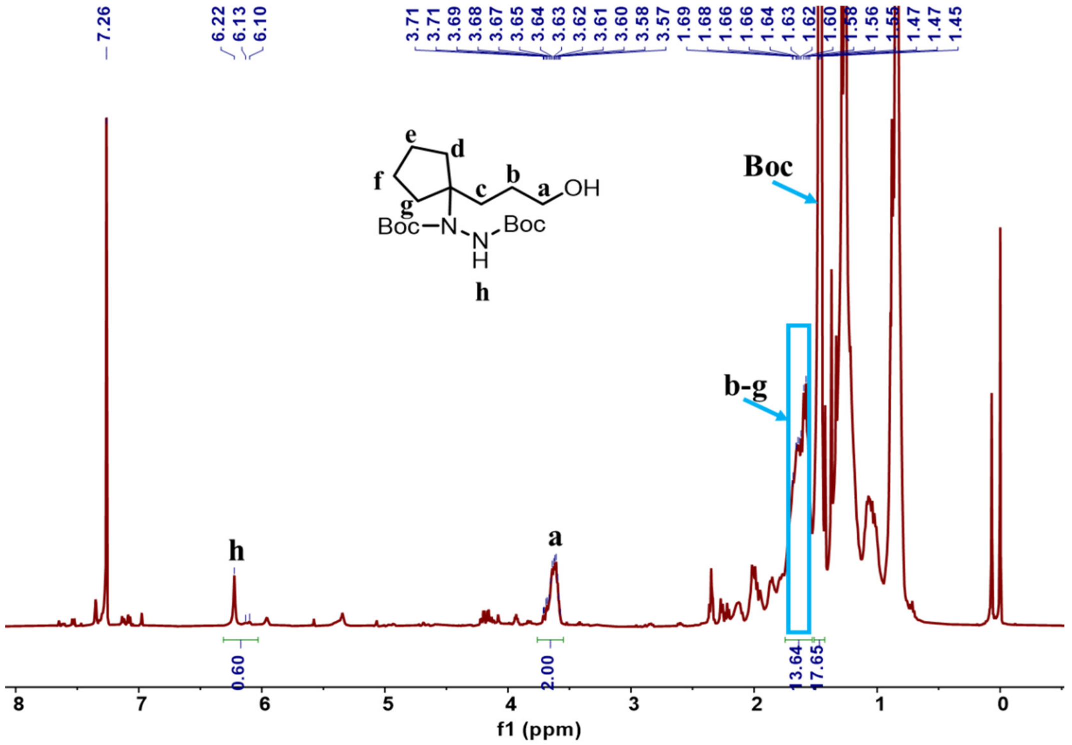

Figure S78. ^{1}H-NMR spectrum of the amination products of 3-cyclopentyl-1-propanol. Label a-g indicate peaks of corresponding Hs on the molecule.

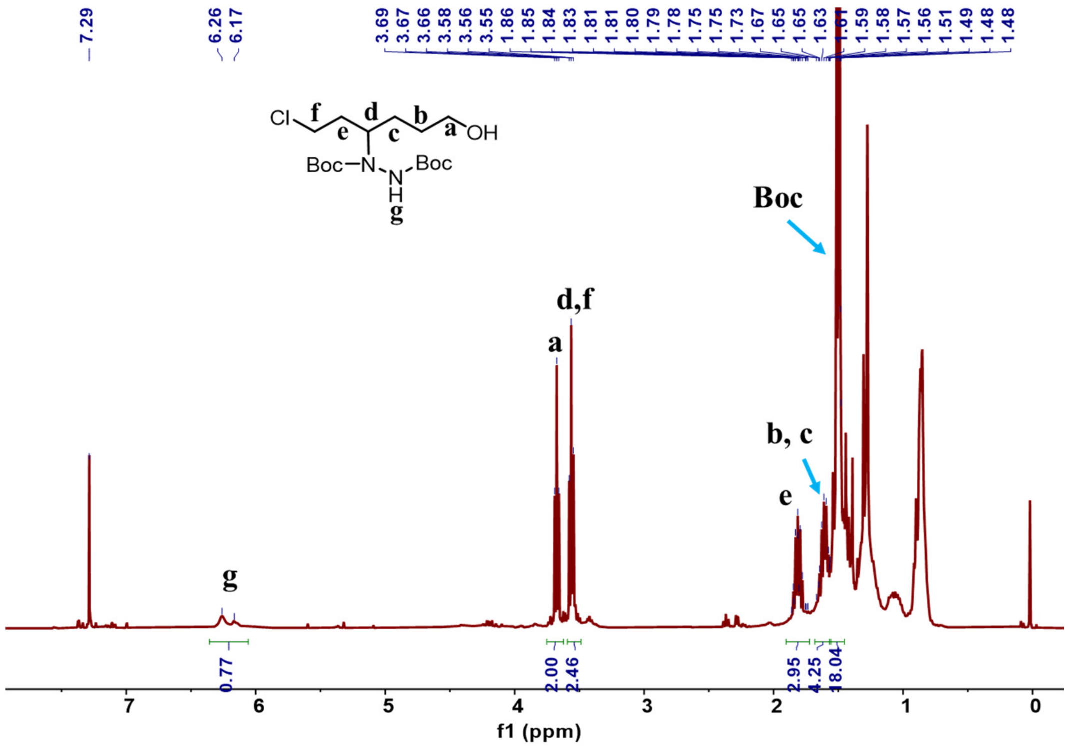

Figure S79. ¹H-NMR spectrum of the amination products of 6-chlorohexanol. Label a-f indicate peaks of corresponding Hs on the molecule.

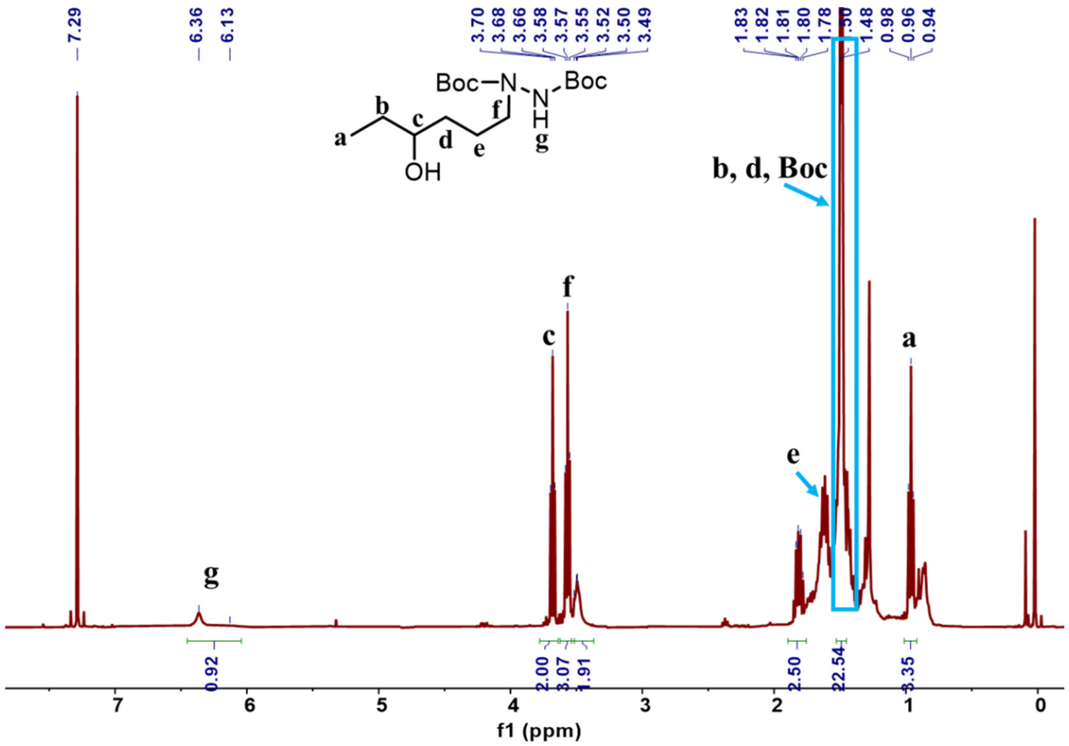

Figure S80. ¹H-NMR spectrum of the amination products of 3-hexanol. Label a-g indicate peaks of corresponding Hs on the molecule.

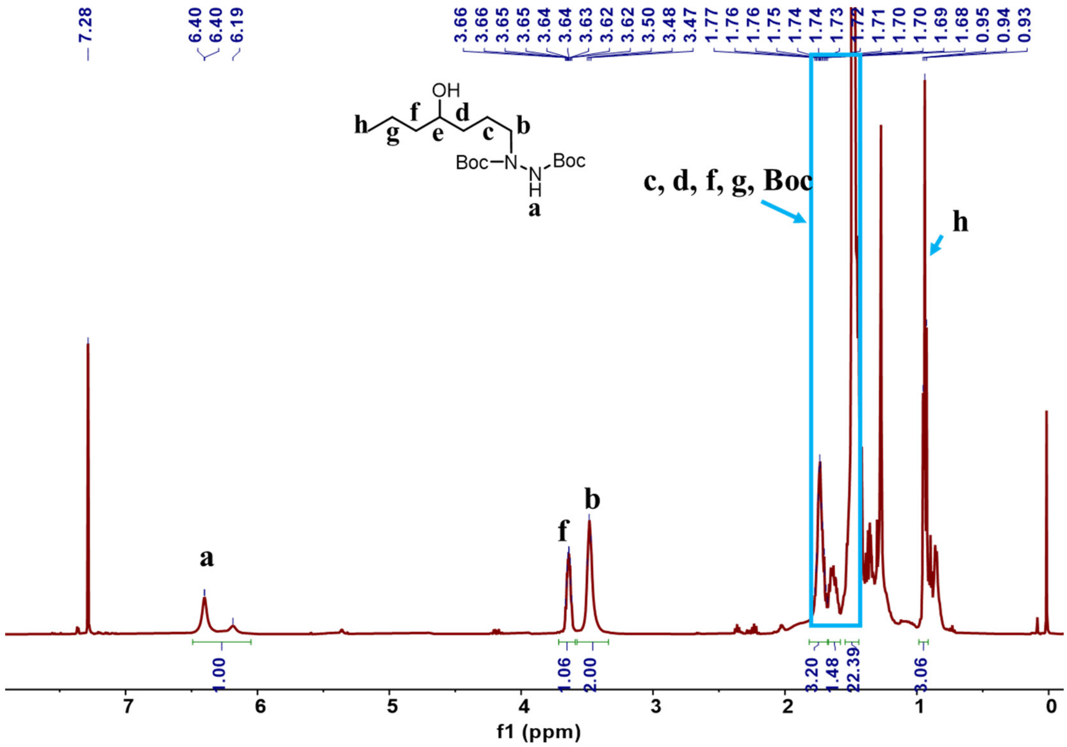

Figure S81. ^{1}H-NMR spectrum of the amination products of 4-heptanol. Label a-h indicate peaks of corresponding Hs on the molecule.

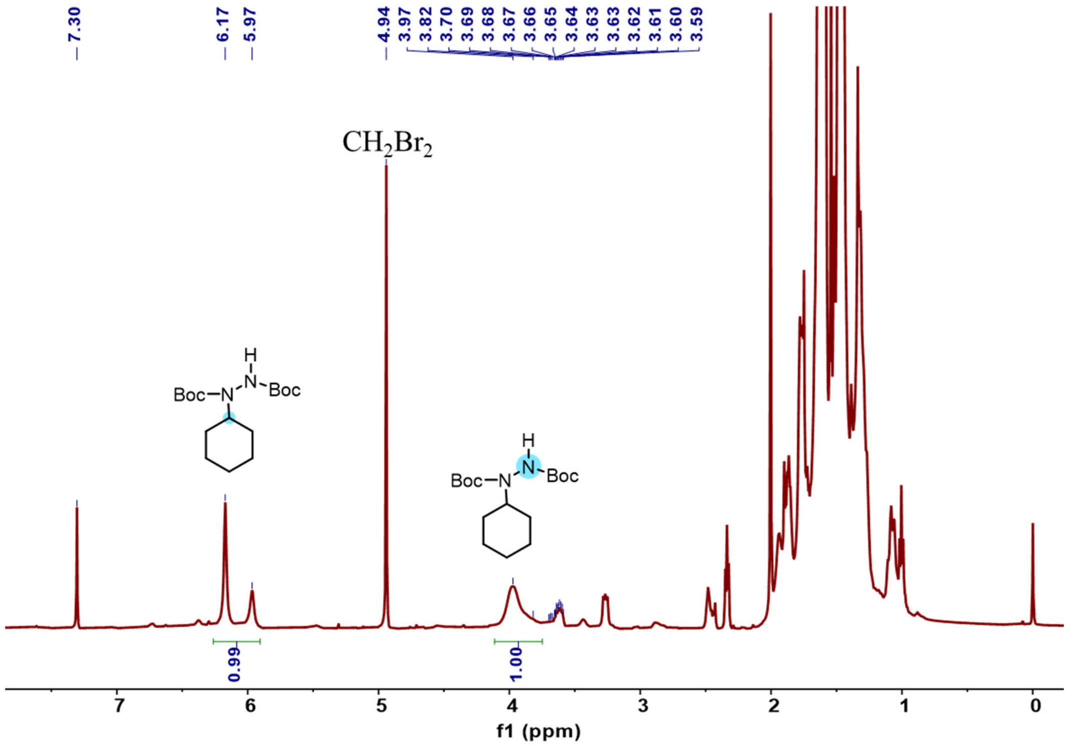

Figure S82. ^{1}H-NMR spectrum of the amination products of cyclohexane.

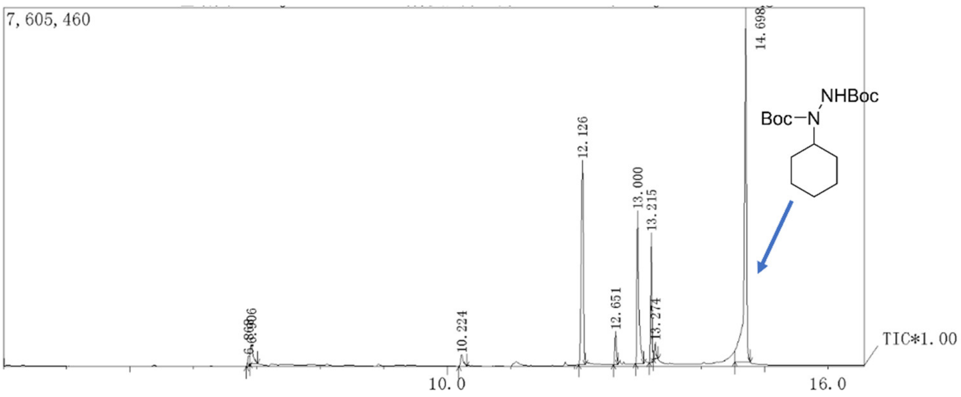

Index	Retention Time	Peak Area	Peak Area%	Peak Height	Area/Height	Mark
1	6.868	491614	1.13	223613	2.20	M1
2	6.906	1082146	2.49	398655	2.71	M1
3	10.224	670909	1.54	245025	2.74	M1
4	12..126	9606660	22.09	4327190	2.22	M1
5	12.651	1090026	2.51	706243	1.54	M1
6	13.000	7087395	16.30	3239927	2.19	M1
7	13.215	3817714	8.78	2751439	1.39	M1
8	13.274	539367	1.24	355425	1.52	M1
9	14.698	19103672	43.93	7500112	2.55	M1
		43489503	100.00	19747629		

Index: 9 Retention Time: 14.700 Mass Peak: 128 Base Peak: 57.10 (1900703)

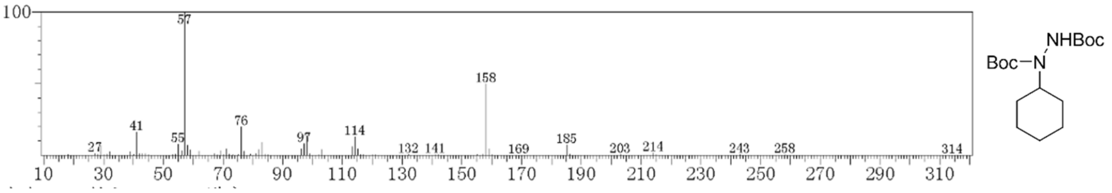

Figure S83. GC-MS trace of the amination products of cyclohexane and experimental MS (lower).

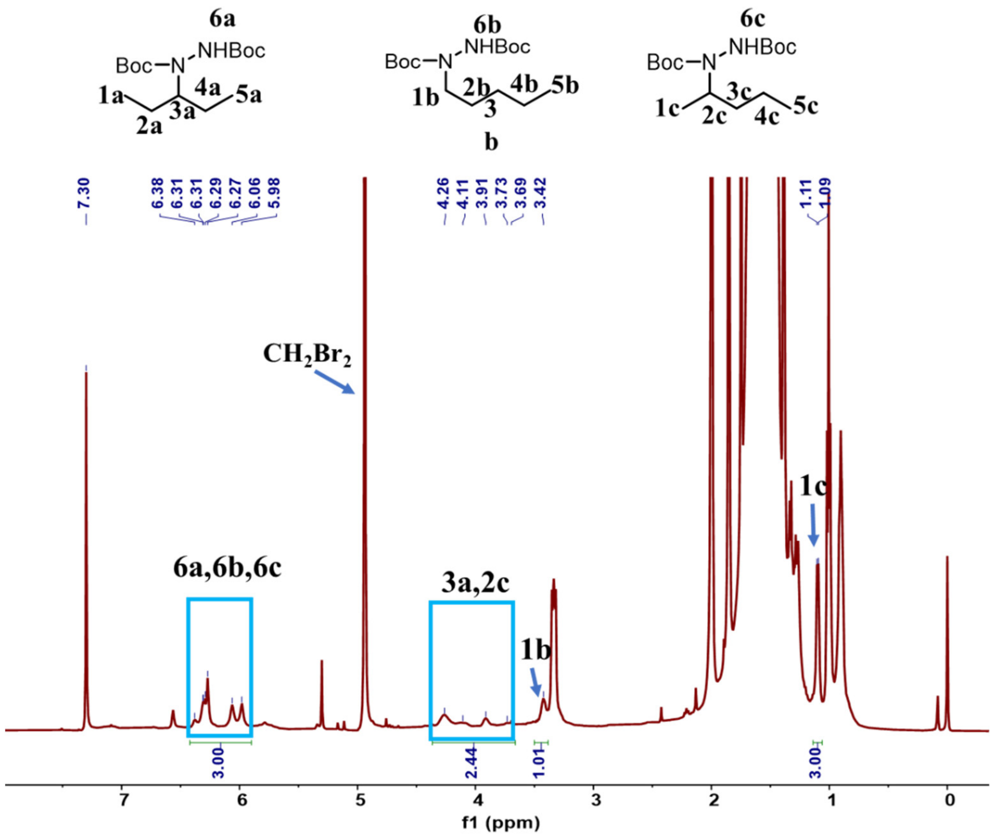

Figure S84. ^{1}H-NMR spectrum of the amination products of *n*-pentane. Label 1a-6c indicates peaks of corresponding Hs on the molecule.

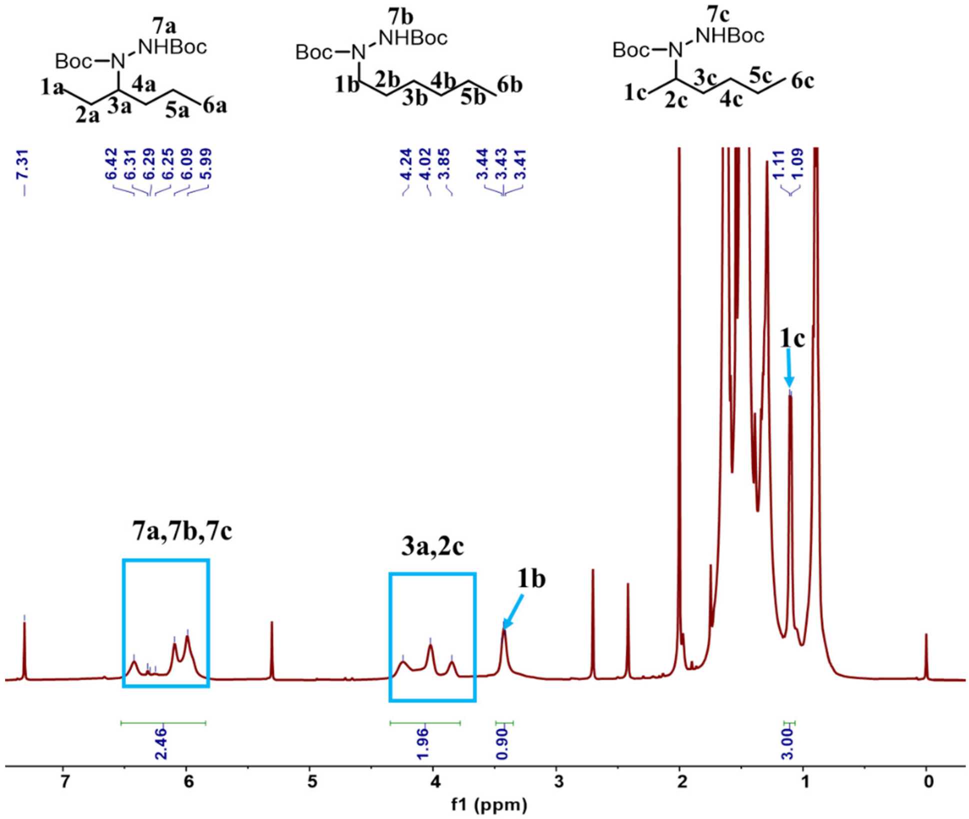

Figure S85. ^{1}H-NMR spectrum of the amination products of *n*-hexane. Label 1a-7c indicates

peaks of corresponding Hs on the molecule.

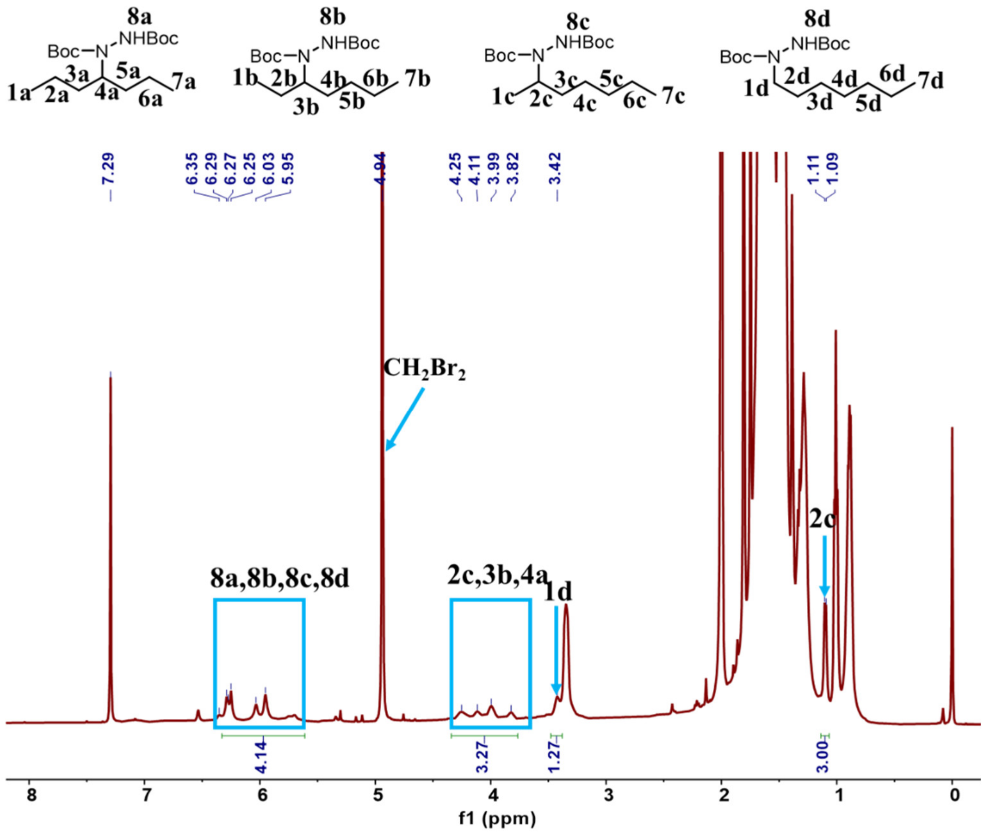

Figure S86. ^{1}H-NMR spectrum of the amination products of *n*-heptane. Label 1a-8d indicates peaks of corresponding Hs on the molecule.

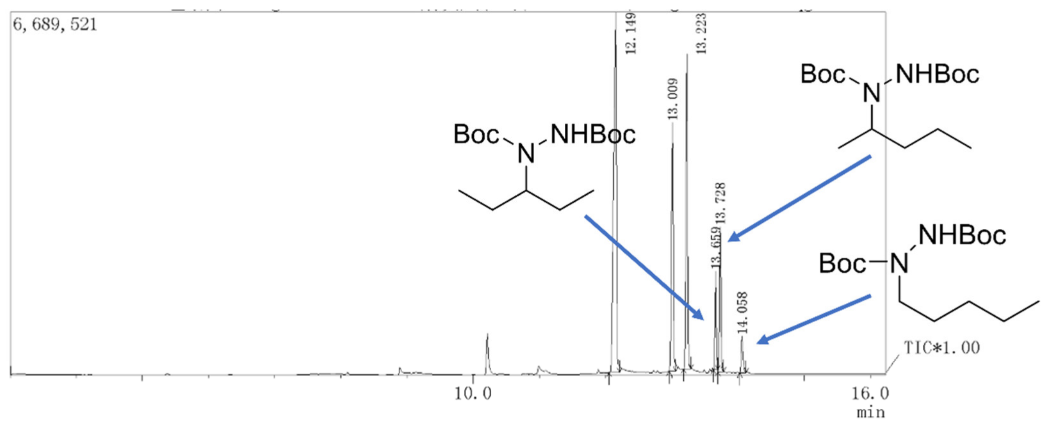

Index	Retention Time	Peak Area	Peak Area%	Peak Height	Area/Height	Mark
1	12.149	20004095	43.56	6633630	3.02	M1
2	13.009	8880898	19.34	4576905	1.94	M1
3	13.223	9362658	20.39	5804389	1.61	M1
4	13.659	2294455	5.00	1820105	1.26	M1
5	13.728	4017097	8.75	2667610	1.51	M1
6	14.058	1362990	2.97	678582	2.01	M1
		45922190	100.00	22181221		

Index: 4 Retention Time: 13.658 Mass Peak: 75 Base Peak: 57.10 (438246)

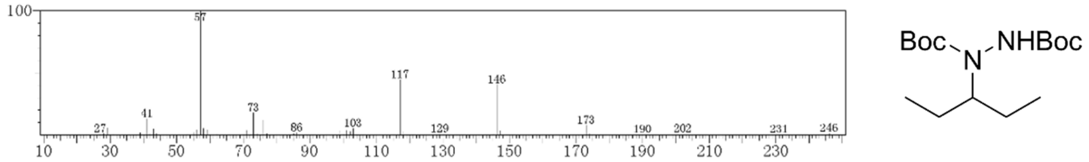

Index: 5 Retention Time: 13.725 Mass Peak: 85 Base Peak: 57.10 (710231)

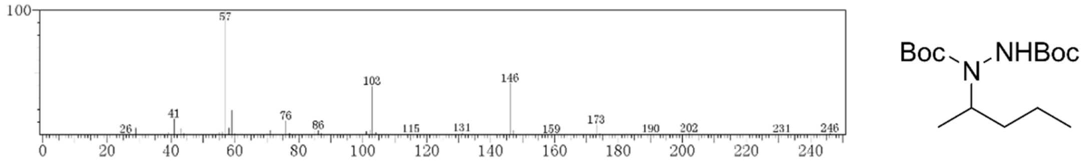

Index: 6 Retention Time: 14.058 Mass Peak: 69 Base Peak: 57.10 (226316)

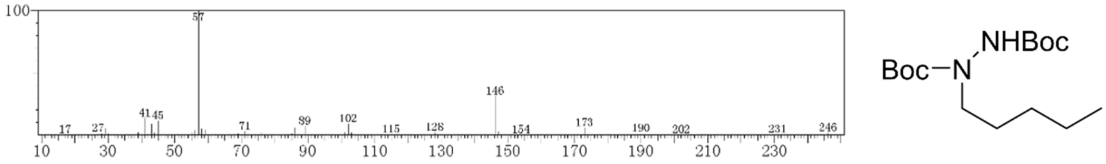

Figure S87. GC-MS trace of the amination products of *n*-pentane and experimental MS (lower).

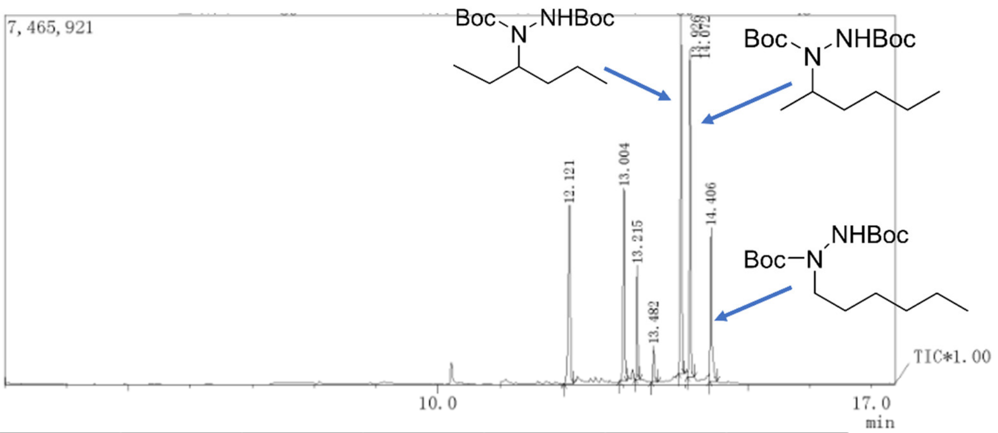

Index	Retention Time	Peak Area	Peak Area%	Peak Height	Area/Height	Mark
1	12.121	8215246	16.90	3606853	2.28	M1
2	13.004	7607108	15.65	3898453	1.95	M1
3	13.215	3145615	6.47	2300773	1.37	M1
4	13.482	1093295	2.25	732646	1.49	M1
5	13.926	11738864	24.15	7232435	1.62	M1
6	14.072	11030348	22.69	6599836	1.67	M1
7	14.406	5772403	11.88	3115540	1.85	M1
		48602879	100.00	27486536		

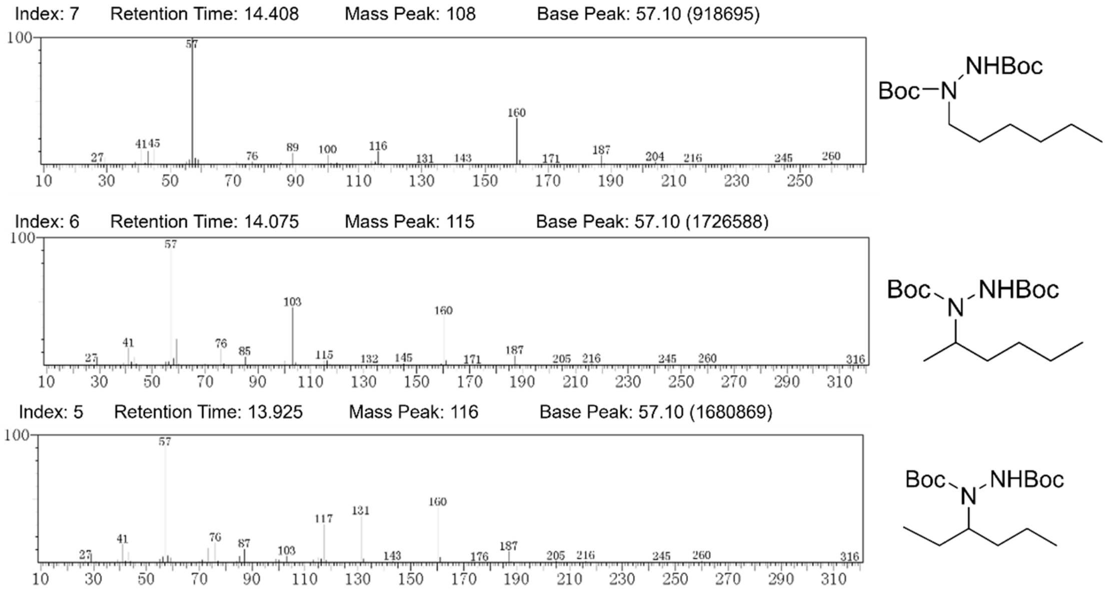

Figure S88. GC-MS trace of the amination products of *n*-hexane and experimental MS (lower).

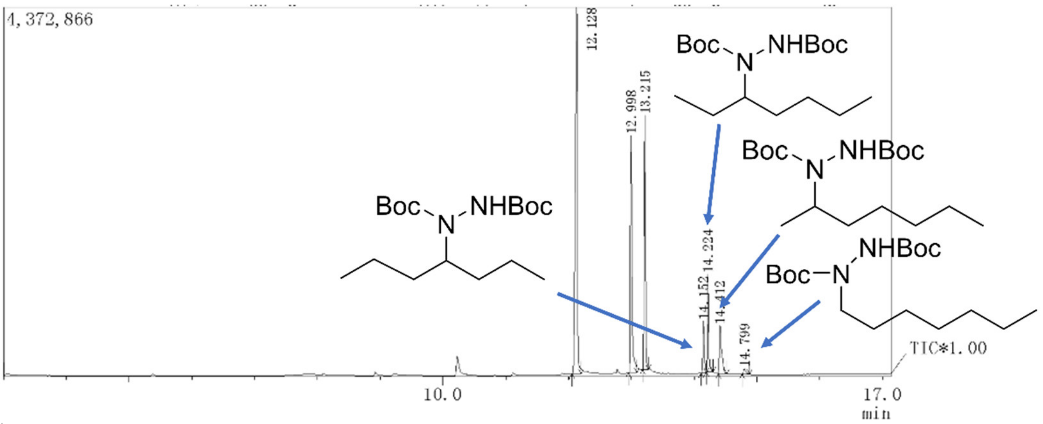

Index	Retention Time	Peak Area	Peak Area%	Peak Height	Area/Height	Mark
1	12.128	10549506	43.61	4337267	2.43	M1
2	12.998	5021575	20.76	2808961	1.79	M1
3	13.215	4280026	17.69	3011157	1.42	M1
4	14.152	886466	3.66	614603	1.44	M1
5	14.224	1734042	7.17	1156049	1.50	M1
6	14.412	1550299	6.41	569693	2.72	M1
7	14.799	167642	0.69	65962	2.54	M1
		24189556	100.00	12563692		

Index: 4 Retention Time: 14.150 Mass Peak: 66 Base Peak: 57.10 (143094)

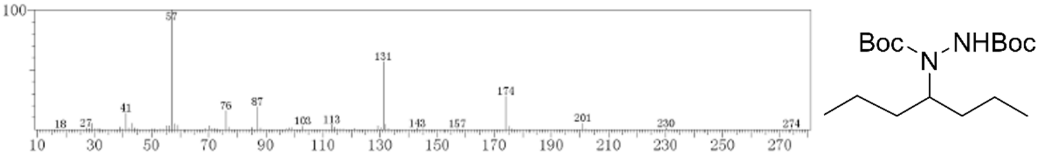

Index: 5 Retention Time: 14.225 Mass Peak: 83 Base Peak: 57.10 (295093)

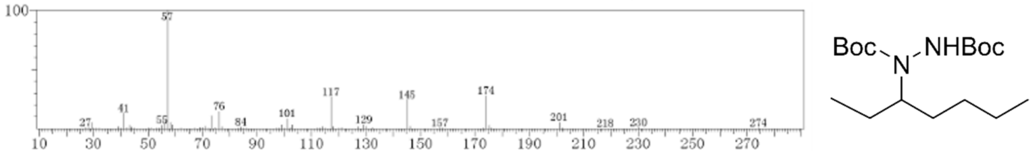

Index: 6 Retention Time: 14.408 Mass Peak: 64 Base Peak: 57.10 (168591)

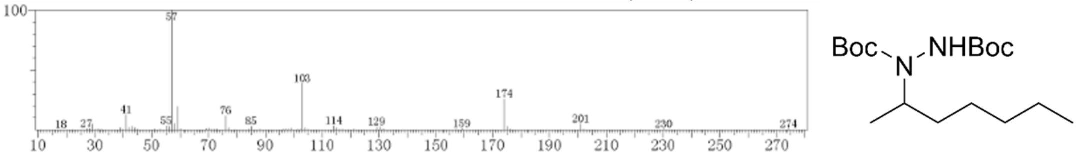

Index: 7 Retention Time: 14.800 Mass Peak: 31 Base Peak: 57.10 (23692)

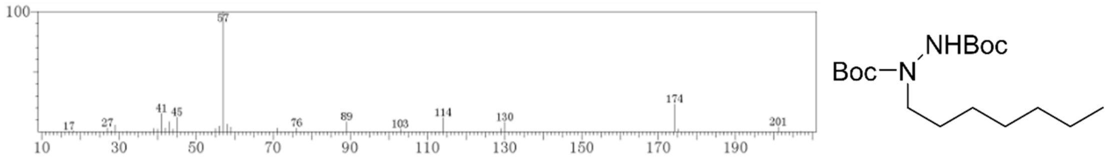

Figure S89. GC-MS trace of the amination products of *n*-heptane and experimental MS (lower).
