# Supplementary material for: Reasoning Language Model as Rule Finder: A Case Study on C–H Bond Activation Using 2D Metal–Organic Frameworks
Source: ACS Cent Sci. 2025 Jun 13;11(7):1135–46. doi: 10.1021/acscentsci.5c00561 (PMC12291106; doi:10.1021/acscentsci.5c00561)
Supplement: Supplementary file 2 [file oc5c00561_si_002.pdf]

oc-2025-005616.R1

Name: Peer Review Information for "Reasoning Language Model as a Rule Finder: on C–H Bond Activation Using 2D Metal-Organic Frameworks"

First Round of Reviewer Comments

Reviewer: 1

Comments to the Author

The article is well written and presents a clear and thoughtful exploration of the photocatalytic δ-C(sp^3)–H amination of 1-pentanol using a metal–organic layer (MOL) catalyst composed of Hf_6 clusters coordinated with terpyridine ligands (Hf-TPY-MOL), functionalized with Fe^{3+} ions. The catalyst (Hf-TPY-MOL-Fe) was synthesized following established procedures and confirmed by PXRD, TEM, UV-Vis, and ICP-OES.

One of the major strengths of the work lies in its emphasis on the use of reasoning large language models (LLMs) as rule finders to predict catalytic outcomes. The authors effectively demonstrate how LLMs can complement traditional descriptors by extracting interpretable rules that connect molecular structure to reactivity.

Importantly, the authors also acknowledge the limitations of both approaches. They point out that traditional models often rely on predefined features, which can reduce interpretability and require substantial domain expertise and feature engineering. At the same time, the manuscript clearly discusses that LLMs, while powerful, can generate outputs not grounded in the input data. By integrating agent-based refinement and systematic validation, the study showcases a balanced and rigorous methodology to mitigate these challenges.

I believe that the quality of the dataset, the depth of discussion, and the novelty of the approach position this work as a valuable contribution to the field. Once the authors address some of the points raised above—particularly clarifying product distributions, elaborating on site selectivity, and discussing the choice of $Fe(OTf)_3$—along with a few minor revisions, I would suggest that this manuscript could be suitable for publication in ACS Central Science.

Please see the comments, questions, and suggestions below

Could the authors clarify the choice of $Fe(OTf)_3$ instead of other common Fe^{3+} salts? What was the specific reason for selecting this counterion?

Also, could you better explain the following procedure?

“To confirm the heterogeneous nature of the catalysis and rule out the leaching of Fe^{3+} ions, UV-Vis absorption spectroscopy of the reaction supernatant revealed no detectable Fe^{3+} signals (Table 1). Cross-experiments were conducted using 1-pentanol as the substrate, followed by the addition of n-hexane to the supernatant after isolating the solid catalyst. Significant conversion of 1-pentanol, but no reaction with n-hexane, confirmed that the supernatant lacked catalytic activity.”

Did the authors intend to say something like:

In the presence of the catalyst, 1-pentanol underwent successful conversion. However, after catalyst removal by filtration, no further conversion was observed in the supernatant, suggesting no leaching of active species and supporting the heterogeneous nature of the catalysis.?

Additionally, why was the classical Sheldon leaching test not used [https://doi.org/10.1006/jcat.1998.1979]? This is a well-established method for confirming catalyst heterogeneity under actual reaction conditions and could have provided more definitive evidence. Please, say a few words about this in the eventual revised version.

The authors wrote:

"In comparison, the Hf-TPY-MOL-Fe has high activity (TON of 233, yield of 35% after irradiation of 12 h) for converting n-hexane to a mixture of substitution at C-1 (di-tert-butyl 1-hexylhydrazine-1,2-dicarboxylate), substitution at C-2, and substitution at C-3 (2.0: 1.9: 1.0)."

This statement appears to be misleading. According to the supplementary information (Figure S3), the actual product distribution seems to be the opposite: C1:C2:C3 = 1.0:1.9:2.0, which I believe is the correct ratio.

On the same subject, the authors also wrote:

"Additionally, a range of different alcohol substrates showed selective C–H conversion at the δ-position with respect to the hydroxyl group, while C–H conversion of alkanes without the hydroxyl group showed a distribution of site selectivity."

Did the authors take into account that each carbon position has a different number of C–H bonds? I suggest re-evaluating the selectivity using the method described by Shulpin (10.2174/157019309788167). For example, in compound 2bc (Figure S3), it is evident that abstraction from the primary carbon (C1)—despite having six hydrogens—is disfavored, as significantly less product is formed at that site. In contrast, when normalized by the number of hydrogens, product distribution across the other positions appears nearly equivalent. There are two C2 positions (totaling 4 H), two C3 positions (4 H), and one C4 position (2 H).

You may also find it helpful to consult Section 6.2 "Selectivity Parameters" in the reference above mentioned, which offers useful insights into quantitative approaches for evaluating site-selective C–H functionalization.

Reviewer: 2

Comments to the Author

This paper presents an integration of reasoning LLMs as rule finders for catalyst design, using Fe-loaded 2D metal-organic frameworks (MOFs). The authors combine LLM-derived interpretable rules with traditional machine learning descriptors to uncover key structure-activity relationships, notably identifying para-substituted benzoates with electron-withdrawing or coordinating groups as performance boosters. The iterative multi-agent framework is particularly interesting, demonstrating not just predictive capability but also mechanistic insight. This work contributes to the emerging field of AI-assisted catalyst design, offering a scalable and interpretable approach. I am happy to recommend for publication after addressing some minor suggestions:

1. Comment on how the LLM-rule extraction framework may generalize to other catalytic systems or chemical reactions.

2. A brief discussion on the current limitations of LLMs and the iterative process would strengthen the paper.

3. Consider refining the graphical summary that more clearly contrasts traditional ML vs. LLM-based approaches for readers less familiar with AI methods.

4. Ensure consistent use of terms like “modifier,” “additive,” and “substituent” throughout the text to avoid confusion.

5. Clarify whether the electronic effects from the para-substituents on benzoates are primarily transmitted through the Hf–carboxylate framework or whether any secondary coordination plays a role.

6. The paper mentions Cl• radical formation via Fe–Cl LMCT. A brief discussion of how the "electronic effect" of the modifiers enhance or inhibit this HAT step would enrich the chemical understanding.

Reviewer: 3

Comments to the Author

This is a timely and innovative study that bridges catalysis and machine learning by introducing a reasoning language model (LLM)-driven framework to identify chemically meaningful rules for C(sp^3)–H bond activation catalyzed by 2D MOFs. The authors have effectively integrated experimental data, domain knowledge, and a multi-agent AI framework to generate interpretable rules that improve prediction accuracy and mechanistic understanding.

The manuscript is well-organized, and the experiments are carefully designed and validated. Particularly commendable is the iterative multi-agent rule refinement strategy, which demonstrates how LLMs can be applied not merely for data fitting but as hypothesis generators and interpretable rule extractors. The inclusion of control experiments, such as the use of Hf-BTB-MOL and functional group-dependent activity analysis, strengthens the mechanistic conclusions.

The combination of LLM-driven reasoning with traditional machine learning descriptors and SHAP analysis provides a compelling case for hybrid AI–chemistry workflows. The finding that para-substituted benzoic acids with electron-withdrawing or coordinating groups enhance catalytic activity is both intuitive and well supported by experimental and computational results.

Overall, this work represents a significant step forward in AI-augmented catalyst design. I strongly support publication and believe this study will inspire future efforts in integrating explainable AI methods into chemical research and materials discovery.

Minor Revisions:

1. It may help to briefly clarify the distinction between "code mode" and "linguistic mode" in the main text when first introduced, for readers less familiar with LLM pipeline implementation.

2. In Figure 5, improving the font size of axis labels and legends would enhance readability, especially when comparing multiple models. Also, please ensure consistent use of LLM names (e.g., “GPT-4o,” “o1”) throughout the figure and captions.

3. While the small dataset is acknowledged, a brief discussion on how the model's generalizability might scale with larger datasets would strengthen the impact and help position the work in broader contexts.

4. There are a few minor typographical issues (e.g., “Matirx” instead of “Matrix” on page 18 "Matirx generator encounter an error

when running code for rule2matrix") that should be corrected during final proofreading.

Author's Response to Peer Review Comments:

Dear Editor,

We have revised the paper according to the suggestions from the reviewers. We have also attached a letter of response to the comments from the reviewers. We hope our revised paper is worthy of consideration for publication in ACS Central Science.

Sincerely Yours,

Cheng Wang

Reviewer 1: The article is well written and presents a clear and thoughtful exploration of the photocatalytic δ-C(sp^3)–H amination of 1-pentanol using a metal–organic layer (MOL) catalyst composed of Hf_6 clusters coordinated with terpyridine ligands (Hf-TPY-MOL), functionalized with Fe^{3+} ions. The catalyst (Hf-TPY-MOL-Fe) was synthesized following established procedures and confirmed by PXRD, TEM, UV-Vis, and ICP-OES.

One of the major strengths of the work lies in its emphasis on the use of reasoning large language models (LLMs) as rule finders to predict catalytic outcomes. The authors effectively demonstrate how LLMs can complement traditional descriptors by extracting interpretable rules that connect molecular structure to reactivity.

Importantly, the authors also acknowledge the limitations of both approaches. They point out that traditional models often rely on predefined features, which can reduce interpretability and require substantial domain expertise and feature engineering. At the same time, the manuscript clearly discusses that LLMs, while powerful, can generate outputs not grounded in the input data. By integrating agent-based refinement and systematic validation, the study showcases a balanced and rigorous methodology to mitigate these challenges.

I believe that the quality of the dataset, the depth of discussion, and the novelty of the approach position this work as a valuable contribution to the field. Once the authors address some of the points raised above—particularly clarifying product distributions, elaborating on site selectivity, and discussing the choice of $Fe(OTf)_3$—along with a few minor revisions, I would suggest that this manuscript could be suitable for publication in ACS Central Science.

Response:

We highly appreciate the reviewers' comments, and we believe the revised manuscript has addressed the points raised by the reviewer.

1. Could the authors clarify the choice of $Fe(OTf)_3$ instead of other common Fe^{3+} salts? What was the specific reason for selecting this counterion?

Response:

We sincerely thank the reviewer for the insightful comment regarding the choice of $Fe(OTf)_3$.

The selection of $Fe(OTf)_3$ as the Fe^{3+} source was based on prior studies reported in Reference 20. In that work, the authors systematically evaluated several Fe^{3+} and Fe^{2+} salts, including $FeCl_3$ (99.99%), $FeCl_3 \cdot 6H_2O$, $Fe_2(SO_4)_3$, $Fe(acac)_3$, $Fe(OTf)_3$, $Fe(NO_3)_3 \cdot 9H_2O$, $FeCl_2$, and $Fe(OAc)_2$. Among these, $FeCl_3$ (99.99%) and $Fe(OTf)_3$ were shown to provide higher catalytic activity, and their performances were nearly equivalent in terms of conversion.

However, due to the potential involvement of Cl^- in ligand-to-metal charge-transfer (LMCT) processes, we sought to decouple the roles of Fe^{3+} and Cl^- by using $Fe(OTf)_3$ as the iron source while separately introducing Cl^- through TBACl. Control experiments (Table 1, Entry 4)

confirmed that Cl^- is indeed essential for the reaction, as no product formation was observed in the absence of TBACl. The inertness of oxidation and coordination of OTf^- also makes $Fe(OTf)_3$ a good choice. Thus, $Fe(OTf)_3$ was selected as the reagent.

We have added the discussion on Fe salts to the Supporting Information, see:

"The selection of $Fe(OTf)_3$ as the Fe^{3+} source was based on the prior study, which compared various Fe^{3+} and Fe^{2+} salts. $FeCl_3$ (99.99%) and $Fe(OTf)_3$ showed the highest catalytic activity with nearly equivalent performance. However, $Fe(OTf)_3$ was chosen to avoid potential interference from Cl^- in ligand-to-metal charge-transfer (LMCT) processes, allowing the separate study of Fe^{3+} and

Cl^- roles (with Cl^- introduced via TBACl)."

2. Also, could you better explain the following procedure?

"To confirm the heterogeneous nature of the catalysis and rule out the leaching of Fe^{3+} ions, UV-Vis absorption spectroscopy of the reaction supernatant revealed no detectable Fe^{3+} signals (Table 1). Cross-experiments were conducted using 1-pentanol as the substrate, followed by the addition of nhexane to the supernatant after isolating the solid catalyst. Significant conversion of 1-pentanol, but no reaction with n-hexane, confirmed that the supernatant lacked catalytic activity."

Did the authors intend to say something like:

In the presence of the catalyst, 1-pentanol underwent successful conversion. However, after catalyst removal by filtration, no further conversion was observed in the supernatant, suggesting no leaching of active species and supporting the heterogeneous nature of the catalysis.?

Response:

We apologize for the confusion. We meant to demonstrate the heterogeneous nature of the catalysis from two aspects: (1) the lack of leached Fe^{3+} signal on UV-Vis spectroscopy after the

reaction, and (2) the lack of catalytic activity of the supernatant after 12 hours of reaction. Specifically, control experiments were carried out by first conducting the catalytic reaction using 1-pentanol as the substrate, showing catalytic performance. After removing the solid catalyst, the resulting supernatant was used in a second reaction with n-hexane as the substrate. The lack of conversion in this second reaction confirmed that the supernatant possessed no catalytic activity. The relevant content of the manuscript was modified for improved clarity, see:

"To confirm the heterogeneous nature of the catalysis and rule out the contribution of leached Fe^{3+} ions, we supplemented our analysis of heterogeneity through multiple substrates in the same process using the same catalyst. After 12 hours of successful reaction on 1-pentanol, the supernatant was extracted and tested by UV-Vis absorption spectroscopy. Then the activity of the supernatant was tested with the reaction to n-hexane. The UV-Vis spectroscopy showed no detectable Fe^{3+} signals (Figure S1), and the result showed no conversion of n-hexane, confirming that the supernatant possessed no catalytic activity, supporting the conclusion that the catalysis is heterogeneous." Additionally, why was the classical Sheldon leaching test not used [https://doi.org/10.1006/jcat.1998.1979]? This is a well-established method for confirming catalyst heterogeneity under actual reaction conditions and could have provided more definitive evidence.

Please, say a few words about this in the eventual revised version.

Response:

Regarding the classical Sheldon leaching test, we acknowledge its established utility for probing catalyst heterogeneity. However, in our system, the precision of yield measurements was limited (a measurement error of ~10% for this heterogeneous reaction). To ensure reliability, we conducted three repeats of experiments for each catalyst and reported the mean yield of the two closest replicates.

This precision makes the classical Sheldon leaching test less reliable. Thus, we supplemented our analysis of heterogeneity through **multiple substrates in the same process using the same catalyst**. After 12 hours of successful reaction on 1-pentanol, the supernatant was extracted and tested by UV-Vis absorption spectroscopy. Then the activity of the supernatant was tested with the reaction to n-hexane. The UV-Vis spectroscopy showed no detectable Fe^{3+} signals (Figure S1), and the result showing no conversion of n-hexane confirmed that the supernatant possessed no catalytic activity, supporting the conclusion that the catalysis is heterogeneous.

3. The authors wrote:

"In comparison, the Hf-TPY-MOL-Fe has high activity (TON of 233, yield of 35% after irradiation of 12 h) for converting n-hexane to a mixture of substitution at C-1 (di-tert-butyl 1-hexylhydrazine-
1,2-dicarboxylate), substitution at C-2, and substitution at C-3 (2.0: 1.9: 1.0)."

This statement appears to be misleading. According to the supplementary information (Figure S3), the actual product distribution seems to be the opposite: C1:C2:C3 = 1.0:1.9:2.0, which I believe is the correct ratio.

Response:

We sincerely appreciate the reviewer's careful examination of our data and figuring out our mistake. You are right—there was an error in the reported product distribution ratio in the manuscript. As shown in Figure S3, the correct ratio for the n-hexane conversion products is indeed C1:C2:C3 = 1.0:1.9:2.0, not 2.0:1.9:1.0. We have corrected this mistake in the revised manuscript and doublechecked other data presented in the text. When normalized with respect to the number of hydrogens on different carbon atoms (as suggested by the reviewer below), the ratio is C1:C2:C3 = 1:2.8:3.0. The relevant content has been corrected, see:

"In comparison, the heterogeneous catalyst Hf-TPY-MOL-Fe has high activity (TON of 233, yield of 35% after irradiation of 12 h) for converting n-hexane to a mixture of substitution at C-1 (ditert-butyl 1-hexylhydrazine-1,2-dicarboxylate), substitution at C-2, and substitution at C-3 (1.0: 2.8:

3.0, normalized with respect to the number of Hs in C1, C2 and C3)."

4. Did the authors take into account that each carbon position has a different number of C–H bonds?

I suggest re-evaluating the selectivity using the method described by Shulpin (10.2174/157019309788167). For example, in compound 2bc (Figure S3), it is evident that abstraction from the primary carbon (C1)—despite having six hydrogens—is disfavored, as significantly less product is formed at that site. In contrast, when normalized by the number of hydrogens, product distribution across the other positions appears nearly equivalent. There are two C2 positions (totaling 4 H), two C3 positions (4 H), and one C4 position (2 H).

You may also find it helpful to consult Section 6.2 "Selectivity Parameters" in the reference above mentioned, which offers useful insights into quantitative approaches for evaluating site-selective C– H functionalization.

Response:

We sincerely appreciate the reviewer's insightful suggestion regarding the normalization of product selectivity by the number of equivalent C–H bonds at each site. The reviewer is correct that this analysis provides a more meaningful evaluation of the intrinsic site selectivity in our C–H functionalization reaction. The relevant content has been added to the manuscript, and we have cited the mentioned paper in the manuscript as Reference 22.

We have now also incorporated this important correction in the revised Supporting Information, where all selectivity data (in Figure S3 and the text) have been properly scaled according to the number of available hydrogens at each carbon site. This re-analysis confirms the reviewer's observation that for n-heptane (C1:C2:C3:C4 = 1.0:14.0:15.4:15.9), the functionalization at the primary C1 position is indeed strongly disfavored relative to secondary positions, while the normalized product distribution across C2, C3, and C4 positions reveals more subtle differences in intrinsic reactivity.

Reviewer 2: This paper presents an integration of reasoning LLMs as rule finders for catalyst design, using Fe-loaded 2D metal-organic frameworks (MOFs). The authors combine LLM-derived interpretable rules with traditional machine learning descriptors to uncover key structure-activity relationships, notably identifying para-substituted benzoates with electron-withdrawing or coordinating groups as performance boosters. The iterative multi-agent framework is particularly interesting, demonstrating not just predictive capability but also mechanistic insight. This work contributes to the emerging field of AI-assisted catalyst design, offering a scalable and interpretable approach. I am happy to recommend for publication after addressing some minor suggestions:

Response:

We appreciate the reviewer's positive comments and acknowledge the suggestions from the reviewer to improve the quality of the manuscript.

1. Comment on how the LLM-rule extraction framework may generalize to other catalytic systems or chemical reactions.

Response:

The LLM-based rule extraction framework demonstrates strong generalizability across catalytic systems and chemical reactions, as it relies primarily on SMILES strings as input. This adaptability allows it to be readily applied to other systems where molecular structures play a key role. The only adjustments required are (1) modifying the prompt to describe the new reaction system and (2) incorporating relevant domain knowledge. These prompts can be efficiently generated using existing LLMs after describing the specific system to be studied. This approach is particularly valuable in cases where a limited number of experimental dataset exists (~100 samples), and the traditional structure-activity relationships remain unclear. Applying machine learning and fingerprints arbitrarily can bring ambiguity in explaining the system. In contrast, by identifying hidden patterns and generating chemically interpretable rules, LLMs can guide further research, bridging data-driven hypotheses with mechanistic understanding. This relevant discussion is added to the manuscript, see:

"Considering the transferability of our LLM-based framework, the approach demonstrates notable versatility across diverse catalytic systems due to its foundation on SMILES

representations. The system's adaptability primarily requires two modifications: (1) customization of prompts to reflect new reaction systems, and (2) incorporation of domain-specific details about the target reaction process. This flexibility is particularly advantageous for research scenarios involving moderate-sized datasets (~100 samples) where conventional structure-activity relationships can be elusive.”

2. A brief discussion on the current limitations of LLMs and the iterative process would strengthen the paper.

Response:

We appreciate the reviewer’s suggestion. There are limitations of the LLMs and the iterative process, indeed. First, the refinement of LLM-generated rules lacks a rigorous mathematical foundation, unlike gradient-based methods such as gradient descent. As a result, iterative updates do not consistently lead to improvement. Second, most LLMs rely on textual input and struggle with heterogeneous data formats, limiting their ability to process complex experimental datasets. This limitation is further compounded by token constraints, which restrict the amount of data or contextual information that can be handled in a single query. While the development of multimodal models or AI agents equipped with specialized tools offers a promising path forward, further research and validation are needed. We have added this discussion in the Conclusion section of the revised manuscript, see:

“Despite these strengths, several limitations remain. First, the refinement of LLM-generated rules lacks a rigorous mathematical foundation, unlike gradient-based methods such as gradient

descent. As a result, iterative updates do not consistently lead to improvement. Second, most LLMs rely on textual input and struggle with heterogeneous data formats, limiting their ability to process complex experimental datasets. This limitation is further compounded by token constraints (e.g., 200,000 for o1), which restrict the amount of data or contextual information that can be handled in the iterative process. While the development of multimodal models or AI agents equipped with specialized tools offers a promising path forward, further research and validation are needed."

3. Consider refining the graphical summary that more clearly contrasts traditional ML vs. LLM-based approaches for readers less familiar with AI methods.

Response:

The TOC of the paper has been revised to clarify the contrasts between traditional ML vs. LLMbased approaches for general readers.

4. Ensure consistent use of terms like "modifier," "additive," and "substituent" throughout the text to avoid confusion.

Response:

We appreciate the reviewer's attention to terminological precision. To ensure conceptual clarity throughout the manuscript, we have systematically standardized our terminology in the revised manuscript as follows:

1. "Modifier" is now exclusively used for carboxylate molecules coordinating to secondary building units (SBUs).
2. "Additive" is reserved for TBACl added to the reaction flask.

3. “Substituent” is consistently applied to functional groups on aromatic rings when describingthe molecular structures of the modifiers.

5. Clarify whether the electronic effects from the para-substituents on benzoates are primarily transmitted through the Hf–carboxylate framework or whether any secondary coordination plays a role.

Response:

We sincerely appreciate this insightful question regarding the electronic transmission mechanism. While we cannot completely exclude some degree of secondary coordination between the modifiers and Fe^{3+} centers, we believe its contribution is limited by three key factors:

1. Thermodynamic preference: The Hf^{4+} nodes, being harder Lewis acids than Fe^{3+}, exhibit a stronger affinity for carboxylate modifiers, favoring their coordination to Hf sites.

2. Experimental procedure: Our sequential preparation method—first modifying the Hf-TPYMOL framework with carboxylates before introducing Fe^{3+}—ensures modifiers are predominantly pre-positioned on Hf^{4+} nodes.

3. Steric constraints: The terpyridine (TPY) plane creates considerable steric hindrance that prevents most benzoate modifiers from accessing additional coordination sites on the Fe^{3+} centers beyond their primary binding to SBUs.

We have added this discussion in the revised manuscript, see:

“At the same time, the thermodynamic preference of Hf^{4+} for carboxylate binding, the sequential experimental preparation method ensuring pre-coordination to Hf nodes, and steric

hindrance from the terpyridine plane collectively reduce the possibility of secondary coordination with Fe^{3+} centers. These factors indicate that the electronic effects from para-substituents on benzoates are primarily transmitted through the Hf-carboxylate framework, other than any secondary coordination."

6. The paper mentions Cl• radical formation via Fe–Cl LMCT. A brief discussion of how the "electronic effect" of the modifiers enhances or inhibits this HAT step would enrich the chemical understanding.

Response:

We sincerely appreciate the reviewer's insightful suggestion regarding the mechanistic role of electronic effects in the HAT step.

The electronic effects of para-substituents on the modifiers govern the Fe–Cl LMCT process and subsequent HAT efficiency by tuning the electron density at the Fe^{3+} center through conjugation via the modified-MOL framework. Electron-withdrawing groups (EWGs) enhance catalytic activity by (1) increasing Fe^{3+} electron deficiency, thereby lowering the LMCT energy barrier and promoting Cl• radical generation, and (2) polarizing the Fe–Cl bond to facilitate homolytic cleavage. Conversely, electron-donating groups (EDGs) diminish Fe^{3+} electron deficiency, slowing LMCT kinetics and reducing Cl• availability. Metal-coordinating groups (e.g., amines) can change into EWGs if they get protonated or coordinate to dissociative Fe^{3+}. Crucially, the para-substitution ensures electronic coupling through π-conjugation while avoiding steric conflicts, as the modifiers' carboxylates and TPY ligands coordinate to Hf^{4+} nodes, physically

isolating substituents from the Fe center. This design confirms that electronic modulation dictates activity, directly linking substituent electronic properties to Cl• radical generation and HAT efficiency in sp^3 C–H activation.

We have significantly expanded the discussion in the analysis part of the manuscript to provide a more complete chemical understanding derived from our LLM extracted rule, see:

"The electronic effects of para-substituents on the modifiers exert precise control over the hydrogen atom transfer (HAT) efficiency by modulating the Fe-Cl ligand-to-metal charge transfer (LMCT) process. Our studies reveal a clear structure-activity relationship: para-substituted benzoates with electron-withdrawing groups (EWGs) or metal-coordinating groups consistently yield superior activity, while electron-donating groups (EDGs) or non-coordinating substituents underperform. This trend originates from the substituents' ability to tune the electron density at the Fe^{3+} center through the conjugated Hf-carboxylate framework.

EWGs enhance catalytic performance through two synergistic mechanisms: (1) they increase the Fe^{3+} center's electron deficiency, lowering the LMCT energy barrier and promoting Cl• radical generation, and (2) they strengthen Fe-Cl bond polarization, facilitating homolytic cleavage. Conversely, EDGs diminish these effects, resulting in slower LMCT kinetics. Notably, metalcoordinating groups. Notably, the metal-coordinating group, like amines, can coordinate to dissociative Fe^{3+} or get protonated, thereby transforming into electron-withdrawing groups.

Structural analysis confirms this electronic property: the para-substituents positioning ensures optimal electronic coupling through π-conjugation while avoiding steric conflicts with the active site. Crucially, the modifiers' carboxylates and TPY ligands coordinate to shared Hf^{4+}

nodes, creating an efficient pathway for electronic effects while physically separating substituents from the Fe center (Figure 6). This arrangement excludes direct interaction between substituents and reaction intermediates/transition states, confirming the primacy of electronic over steric effects in governing activity.

As a result, these analyses emphasized the importance of the LMCT process between Fe^{3+} and Cl^{-}, providing guidance for improving the yield of the original sp^3 C–H bond activation."

Reviewer 3: This is a timely and innovative study that bridges catalysis and machine learning by introducing a reasoning language model (LLM)-driven framework to identify chemically meaningful rules for C(sp^3)–H bond activation catalyzed by 2D MOFs. The authors have effectively integrated experimental data, domain knowledge, and a multi-agent AI framework to generate interpretable rules that improve prediction accuracy and mechanistic understanding.

The manuscript is well-organized, and the experiments are carefully designed and validated. Particularly commendable is the iterative multi-agent rule refinement strategy, which demonstrates how LLMs can be applied not merely for data fitting but as hypothesis generators and interpretable rule extractors. The inclusion of control experiments, such as the use of Hf-BTB-MOL and functional group-dependent activity analysis, strengthens the mechanistic conclusions. The combination of LLM-driven reasoning with traditional machine learning descriptors and SHAP analysis provides a compelling case for hybrid AI-chemistry workflows. The finding that parasubstituted benzoic acids with electron-withdrawing or coordinating groups enhance catalytic activity is both intuitive and well supported by experimental and computational results.

Overall, this work represents a significant step forward in AI-augmented catalyst design. I strongly support publication and believe this study will inspire future efforts in integrating explainable AI methods into chemical research and materials discovery.

Response:

We appreciate the reviewer's positive comments on our contributions to AI-augmented catalyst design.

1. It may help to briefly clarify the distinction between "code mode" and "linguistic mode" in the main text when first introduced, for readers less familiar with LLM pipeline implementation.

Response:

We appreciate the reviewer's suggestion to clarify the distinction between the two operational modes of the LLM pipeline. We have added a more detailed explanation in the manuscript, see:

> *"Linguistic mode: In this approach, the LLM directly processes chemical structures (provided as SMILES strings) and proposed rules using its pre-trained knowledge, generating a feature matrix through natural language processing. This mode leverages the model's inherent chemical understanding without explicit programming steps, producing an output matrix of dimensions (number of SMILES) × (number of rules).*
>
> *Code mode: Here, the LLM first translates generated rules into executable Python functions. These programmatic rules are then systematically applied to the SMILES data through automated script execution, generating the feature matrix computationally. This mode provides more transparent and reproducible rule implementation while maintaining the LLM's interpretative advantages."*

2. In Figure 5, improving the font size of axis labels and legends would enhance readability, especially when comparing multiple models. Also, please ensure consistent use of LLM names (e.g., "GPT-4o," "o1") throughout the figure and captions.

Response:

We appreciate the reviewer's helpful suggestions. We have now ensured consistent naming conventions for all LLMs (e.g., "GPT-4o" and "o1") across the figure, captions, and text. Additionally, we have improved readability by increasing the font size of axis labels and legends by 110% in the revised version of Figure 5.

3. While the small dataset is acknowledged, a brief discussion on how the model's generalizability might scale with larger datasets would strengthen the impact and help position the work in broader contexts.

Response:

We thank the reviewer for highlighting this important point regarding model generalizability. In response, we have revised the Conclusion section to discuss the potential for scaling to larger datasets. While our method demonstrates strong transferability across diverse chemical systems through prompt-based adaptation, its current implementation is most efficient for medium-sized datasets. This is primarily due to limitations in handling heterogeneous data formats and the token constraints inherent to LLMs. Nonetheless, we believe that with future developments, such as integration with multimodal models or tool-augmented agents, the approach can be extended to larger-scale applications.

We have clarified this perspective in the revised manuscript, see:

"Considering the transferability of our LLM-based framework, the approach demonstrates notable versatility across diverse catalytic systems due to its foundation on SMILES representations. The system's adaptability primarily requires two modifications: (1) customization

of prompts to reflect new reaction systems, and (2) incorporation of domain-specific details about the target reaction process. This flexibility is particularly advantageous for research scenarios involving moderate-sized datasets (~100 samples) where conventional structure-activity relationships can be elusive.

Despite these strengths, several limitations remain. First, the refinement of LLM-generated rules lacks a rigorous mathematical foundation, unlike gradient-based methods such as gradient descent. As a result, iterative updates do not consistently lead to improvement. Second, most LLMs rely on textual input and struggle with heterogeneous data formats, limiting their ability to process complex experimental datasets. This limitation is further compounded by token constraints (e.g., 200,000 for o1), which restrict the amount of data or contextual information that can be handled in the iterative process. While the development of multimodal models or AI agents equipped with specialized tools offers a promising path forward, further research and validation are needed."

4. There are a few minor typographical issues (e.g., "Matirx" instead of "Matrix" on page 18 "Matirx generator encounter an error when running code for rule2matrix") that should be corrected during final proofreading.

Response:

We sincerely appreciate the careful review of our manuscript and the identification of these typographical errors. We have thoroughly proofread the entire document and corrected all instances of misspellings (including "Matirx" to "Matrix" on page 18) and other minor grammatical issues.

Formatting Needs:

Abstract: Please make sure the word count of your Abstract does not exceed 200 words.

The abstract is less than 200 words now, see:

*“**Abstract:** Unraveling the structure-activity relationship in catalysis requires interpretable models that can extract governing principles from complex datasets. This study explores reasoning large language models (LLMs) as rule-finders for predicting C(sp^3)–H activation outcomes catalyzed by 2D Fe-terpyridine MOFs. Surface modifications with molecular modifiers systematically modulate the catalytic microenvironment, but linking modifier structure to activity remains challenging. While traditional descriptors offer high predictive accuracy, LLM-derived rules provide interpretable insights. Integrating LLM reasoning with experimental features (e.g., Fe-loading, modifier ratios) identified para-substituted benzoates with electron-withdrawing or coordinating groups as performance boosters. Validated by machine learning, this rule achieved 82.6% prediction accuracy. Notably, the coordinating group can become electron-withdrawing upon Fe^{3+} coordination or protonation. The LLM revealed that modifiers tune the catalyst's electronic state rather than directly interacting with intermediates/transition states, bridging data-driven predictions with mechanistic understanding. This highlights LLM's potential to derive chemically meaningful rules in catalysis.”*

Graphics: If a figure has parts labeled (i.e., a, b, etc.), each part must be mentioned in the figure caption. (See Figure 4.)

We use Figure 4a, b to cite the figure instead, see:

"The way of representing a rule by code is quite flexible (Figure 4a, b)."

Major Objects: Please cite each major object (figures, tables, equations and schemes) in the manuscript text. If a figure is not cited as a whole, at least one part must be cited (e.g. Figure 1 or Figure 1a). Figure 6 does not appear to be cited.

We have cited Figure 6 in the manuscript now, see

"Crucially, the modifiers' carboxylates and TPY ligands coordinate to shared Hf^{4+} *nodes, creating an efficient pathway for electronic effects while physically separating substituents from the Fe center (Figure 6)."*

Supporting Information: If the manuscript is accompanied by any Supporting Information for Publication, a brief description of the supplementary material is required in the manuscript before the reference list. The appropriate format is: Supporting Information. Brief statement in non-sentence format listing the contents of the material supplied as Supporting Information. Please list each supporting item individually.

*Examples of sufficient descriptions: "Supporting Information: 1H NMR spectra for all compounds" or "Additional experimental details, materials, and methods, including photographs of experimental setup."

*Examples of insufficient descriptions: "Supporting Information: Figures S1-S3" or "Additional figures as mentioned in the text."

Yes, the description has been added now, see

*“**Supporting Information***

Additional experimental details, materials, feature sets, machine learning results, and H^1 NMR spectra for all the experiments.”

Supporting Information: Provide authors’ names and affiliations in the Supporting Information file, matching those in the manuscript file.

Yes, the relevant information is added.

Supporting Information: Please number all pages in the following format: S1, S2, S3, etc.

Yes, all the pages are numbered.

Synopsis: ACS Central Science requires a brief synopsis. The synopsis should be no more than 200 characters (including spaces) and should reasonably correlate with the Table of Contents (TOC) graphic. The synopsis is intended to explain the importance of the article to a broader readership across the sciences. Please place your synopsis in the manuscript file after the TOC graphic and label as “Synopsis.”

A synopsis is added below the TOC graph in the manuscript, see

*“**Synopsis:** Reasoning large-language models combined with machine learning uncover structureactivity rules for Fe^{3+}-loaded metal-organic frameworks, enabling interpretable $C(sp^3)$–H bond activation insights and AI-driven catalyst design.”*

TOC Graphic: Please label your TOC Graphic as "TOC Graphic" in the manuscript file.

Yes, it is labelled as "TOC Graphic" now.
